# Boradigermaallyl: inhibition of CH bond activation by borane CO adduct formation followed by CO insertion†

**DOI:** 10.1039/d5sc00881f

**Published:** 2025-03-27

**Authors:** Ralf H. Kern, Noemi Hiller, Klaus Eichele, Hartmut Schubert, Christina Tönshoff, Holger F. Bettinger, Lars Wesemann

**Affiliations:** a Institut für Anorganische Chemie, Eberhard Karls Universität Tübingen Auf der Morgenstelle 18 72076 Tübingen Germany lars.wesemann@uni-tuebingen.de; b Institut für Organische Chemie, Eberhard Karls Universität Tübingen Auf der Morgenstelle 18 72076 Tübingen Germany

## Abstract

Boradigermaallyl, valence-isoelectronic to an allyl cation, stabilized by Ge-bound C_6_H_3_-2,6-(Trip)_2_ (Trip = 2,4,6-C_6_H_2_iPr_3_) groups shows triple insertion of ethylene into the Ge–B bonds (2) or an addition of styrene at the Ge atoms (3) followed by CH addition of a Trip methyl group at a GeB unit. Phenylacetylene forms two addition products (4, 5) with the GeB unit or both Ge atoms, which are also followed by a CH insertion of a methyl group. Under CO atmosphere the CH addition was prevented in the case of the phenylacetylene addition, and a CO adduct of this cycloaddition product (6) was characterized. Subsequently this CO adduct exhibits a CO insertion into the B–C bond and an α,β-unsaturated acylboron compound (7) was characterized. In the case of the anthracene addition to boradigermaallyl the observed CH addition was also suppressed by CO adduct formation (8). Biphenylene reacts with boradigermaallyl at room temperature under insertion of a boron atom into a phenyl moiety (9).

## Abstract 2

Multifaceted reactions of boradigermaallyl with unsaturated organic molecules are presented. CH activation is suppressed by CO borane adduct formation and subsequent CO insertion results in an α,β-unsaturated acylboron compound.

## Graphical abstract

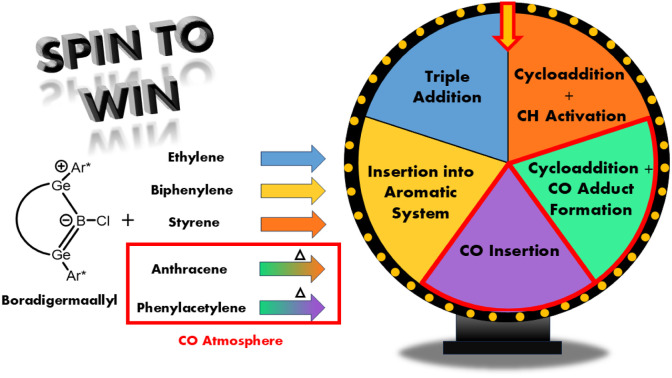

## Introduction

As subvalent boron compounds, borylenes represent a class of highly reactive intermediates.^1–3^ This was convincingly demonstrated by fluoroborylene, synthesized under matrix isolation conditions from boron and elemental fluorine, which is able to cleave the triple bond of nitrogen.^4^ Phenylborylene, characterized by matrix experiments, undergoes insertion into an *ortho*-CH bond of the phenyl ring at 10 K upon photolysis (*λ* > 350 nm).^5^ Boron monofluoride and chloride, synthesized by low pressure reaction of halides BX_3_ (X = F, Cl) with boron at 2000 °C, react with alkynes and alkenes.^1,6–8^ The formation of transient borylenes was postulated after isolation of trapping products resulting in the formation of borirenes,^9–11^ borylene (R_2_NB, Ar′B) insertion,^12,13^ H_2_ and CH addition at boron.^14–17^ Stabilization of borylenes in Lewis base adducts or as ligands in transition metal complexes is a highly active field of research.^3,18^ In transition metal chemistry borylenes are known as terminal and bridging ligands with a variety of metals.^18,19^ Mono as well as bis(Lewis-base) adducts of borylenes were characterized and their diverse reactivity is the starting point of versatile chemistry.^20,21^

**Scheme 1:**
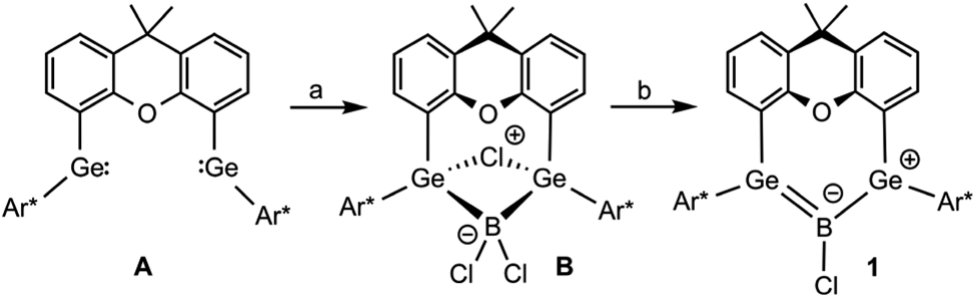
Synthesis of boradigermaallyl 1. (a) Me_2_S·BCl_3_, (b) {(^Mes^Nacnac)Mg}_2_, [^Mes^Nacnac = {[(Mes)NC(Me)]_2_CH}^−^, Ar* = C_6_H_3_-2,6-(Trip)_2_, Trip = 2,4,6-C_6_H_2_iPr_3_].^22–24^

We introduced a chelating germylene A (Scheme 1) as a novel means of stabilizing chloroborylene.^22^ The GeBGe linkage extends the reactivity beyond that of typical borylene chemistry and it is thus an attractive system for the exploration of novel chemical space. Addition of boron trichloride to a bis(germylene) moiety followed by reduction of the bridging Ge(BCl_2_)Ge B unit results in a delocalized low valent boron compound with chloroborylene coordinated by two germylenes 1 (Scheme 1). Due to delocalisation of the boron electron pair into the empty p-orbitals of the germylenes a boradigermaallyl results. The electronic situation of this species is comparable with the allyl-cation and can also be regarded as a bis(germylene) adduct of chloroborylene exhibiting a high amount of π-back bonding from boron to the germanium atoms.^22^ High reactivity of the boradigermaallyl is documented by insertion into a C–C bond of benzene at room temperature.

In this publication we present reactions of boradigermaallyl with the unsaturated molecules ethylene, styrene, phenylacetylene, anthracene, and biphenylene. For the phenylacetylene and anthracene reactions the influence of carbon monoxide is investigated. The coordination of CO at boron was found to suppress subsequent CH addition reactions. Furthermore, we present a rare example of isolation and structural characterization of both a borane CO adduct and the resulting CO insertion product.

## Results and discussion

**Scheme 2:**
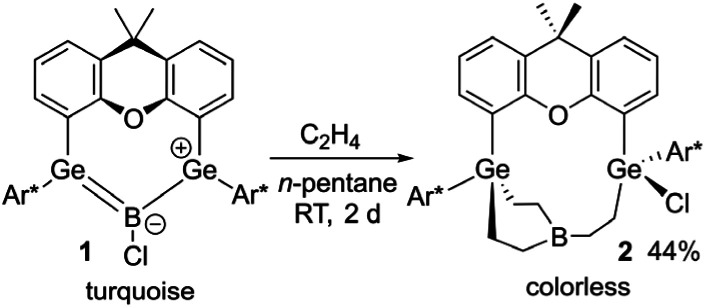
Triple addition of ethylene to boradigermaallyl.

**Fig. 1:**
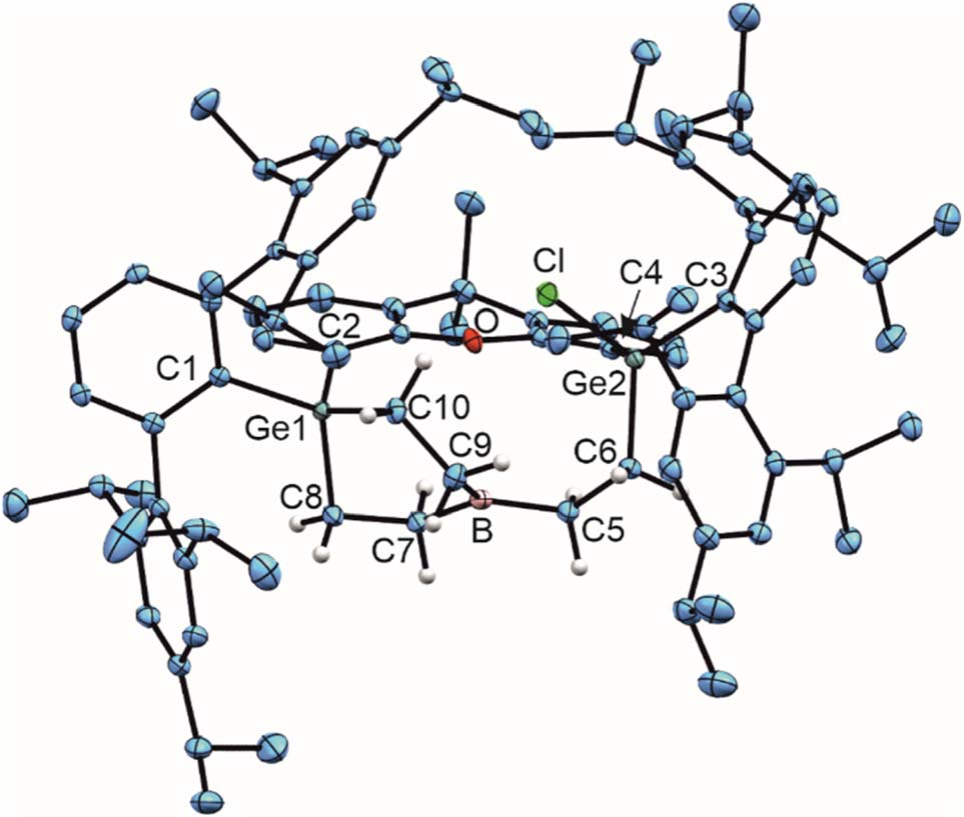
ORTEP of the molecular structure of 2. Thermal ellipsoids are shown at 50% probability level. Hydrogen atoms except ethylene H-atoms have been omitted. Selected interatomic distances [Å] and angles [°]: B–C5 1.573(2), B–C7 1.578(3), B–C9 1.573(3), C5–C6 1.538(2), C7–C8 1.542(2), C9–C10 1.550(2), Ge2–C6 1.9556(17), Ge1–C8 1.9447(17), Ge1–C10 1.9659(16), C9–B–C5 122.2(2), C9–B–C7 119.2(2), C5–B–C7 118.2(2).

In reaction with an excess of ethylene at room temperature in *n*-pentane boradigermaallyl (1) shows a triple (2 + 2) cycloaddition accompanied by a chloride transfer from the boron atom to a germanium atom (Scheme 2). The molecular structure of compound 2 (Fig. 1) exhibits an almost trigonal planar arrangement of the boron atom [Σ angles at B: 359.6(1)°]. The B–C bond lengths [1.573(2)–1.578(3) Å] can be compared with a single bond between these elements, similar to the bond lengths in the molecular structure of BEt_3_.^25^ In solution a ^11^B NMR signal could not be obtained, which is probably due to the trigonal planar arrangement at the boron atom causing a very broad ^11^B NMR signal.

**Scheme 3:**
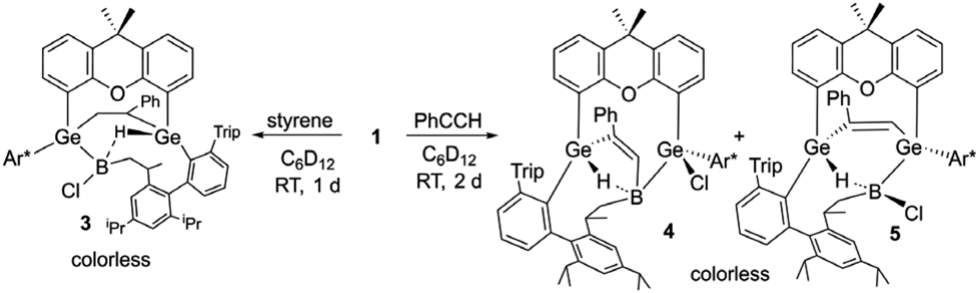
Styrene and phenylacetylene reaction of 1.

Multiple addition of ethylene has been observed for a variety of main group element compounds: disilyne,^26,27^ digermyne,^28–30^ distannyne,^31^ silylsilylene,^32^ germylene, and gallylgermylene.^33^ In the cases of the digermynes and distannynes this addition is a reversible reaction. In comparison to the small ethylene molecule, an excess of the more sterically demanding styrene in reaction with boradigermaallyl (1) leads to product 3, which was isolated by crystallization at room temperature (Scheme 3, left). Compound 3 is the product of a single olefin addition at the germanium atoms of 1 followed by a CH bond activation of a methyl group of the Trip moiety by a GeB unit (see ESI† for molecular structure of 3). The resulting GeH unit shows in the solid-state structure an interaction with the boron atom to give a bridging Ge–H–B moiety. In the solution ^11^B NMR spectrum of 3 a signal was not observed.

The reactivity of the boradigermaallyl towards a C≡C triple bond was also evaluated in the reaction with phenylacetylene (Scheme 3, right). Two different types of colorless crystals were found after crystallization of the reaction mixture between 1 and phenylacetylene. By single crystal diffraction two products (4, 5) were characterized (see ESI† for molecular structures), but a further separation of crystals was not possible. Therefore, NMR characterization of 4 and 5 in the product mixture is not possible. Compound 4 is the product of an addition reaction between a GeB unit and the C≡C triple bond. In the case of compound 5 the alkyne shows a twofold bond formation with the Ge atoms of 1. Like in compound 3, in both cases 4 and 5 a reaction of a CH unit of a Trip methyl group with a GeB unit was found to give a BCH_2_ and a GeH moiety. This GeH unit exhibits an interaction with the boron atom in the molecular structures of 4 and 5.

Based on the molecular structures of the reaction products of the styrene 3 and phenylacetylene reactions 4, 5 we assume that after addition of unsaturated organic molecules to 1 a triply-coordinate electrophilic boron species, having a Ge–B bond, is initially formed, which subsequently reacts with a Trip methyl CH unit to give the isolated products 3, 4 and 5. To prevent this undesired CH addition we added a weak Lewis base to the reaction mixtures to coordinate at the intermediately formed electrophilic boron atom of the presumed olefin or alkyne addition products. A strong Lewis base was not used because boradigermaallyl (1) was found to add ^Me^NHC to the germanium atom.^22^

**Scheme 4:**
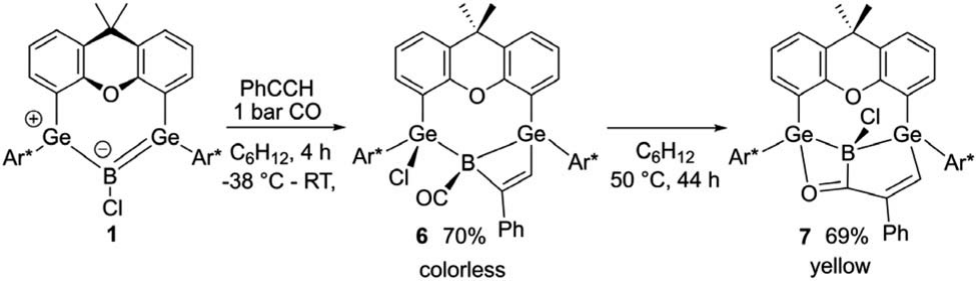
Phenylacetylene addition under an atmosphere of CO.

**Fig. 2:**
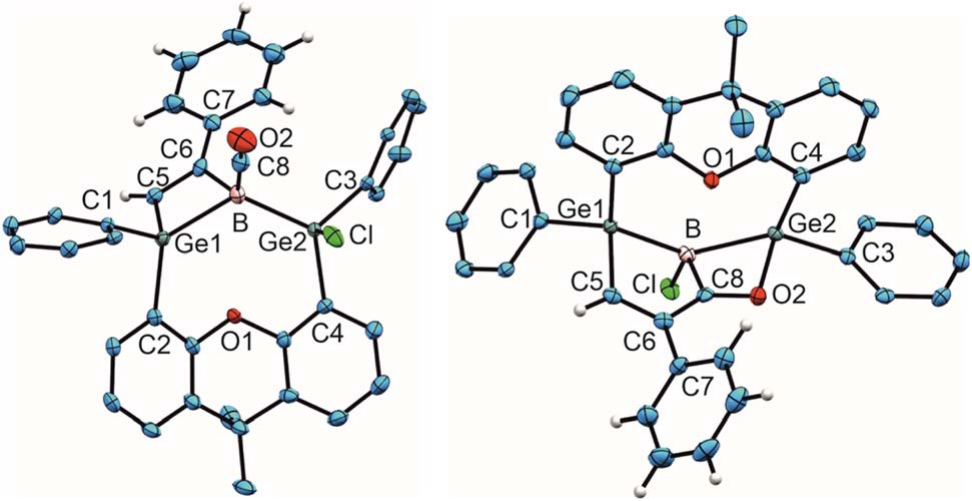
ORTEP of the molecular structure of 6 (left) and 7 (right). Thermal ellipsoids are shown at 50% probability level. Hydrogen atoms and Trip groups except phenylacetylene H-atoms have been omitted. Selected interatomic distances [Å] and angles [°] 6: B–C8 1.492(5), C8–O2 1.139(4), Ge1–B 2.194(3), Ge2–B 2.109(3), B–C6 1.660(5), C6–C5 1.342(4), C5–Ge1 1.932(3), Ge1–B–Ge2 124.5(2), B–C8–O2 176.0(3), B–C6–C5 104.7(2), C6–C5–Ge 101.8(2), C5–Ge–B 70.3(1), Ge1–B–C6 82.3(2); 7: Ge1–B 2.133(2), Ge2–B 2.159(2), B–C8 1.605(3), B–Cl 1.8396(19), C8–O2 1.275(2), C8–C6 1.466(2), C5–C6 1.350(3), C5–Ge1 1.9802(19), O2–Ge2 2.0098(13), Ge1–B–Ge2 136.6(1), Cl–B–C8 116.8(1), B–C8–O2 114.5(2), C8–O2–Ge 93.9(1), O2–Ge2–B 71.2(1), Ge2–B–C8 79.9(1).

Thus, boradigermaallyl was reacted with the unsaturated organic molecules styrene or phenylacetylene under an atmosphere of carbon monoxide, which should react as a weak Lewis base and is known to form adducts with boranes (Scheme 4 and Table 1C–I), but not with 1.^7,34–40^ Deduced from NMR spectroscopy, the styrene/CO reaction mixture with 1 resulted in a product other than 3, which however, could not be isolated. However, in the case of phenylacetylene we characterized a CO adduct 6 of the alkyne addition product (Scheme 4 and Fig. 2).

**Table 1:** Analytical data of borane carbonyl adducts^a^^,^^b^

	^11^B (ppm)	^13^C (ppm)	IR (cm^−1^)	B–C (Å)	C–O (Å)
C				1.534(10)	1.135(10)
D	—	—	2162	1.522(5)	1.117(3)
E	−20.7	—	2176	1.544(15)	1.091(14)
F	−16	166.4	2199	1.609(3)	1.115(3)
G	−17.9	159.8	2252	1.69(2)	1.11(2)
H	−16.4	160.3	2252	1.618(2)	1.109(2)
I	−18.8	169.5	2128	1.56(2)	1.10(2)
6	−23.2	181.6	2077	1.492(5)	1.139(4)
8	−21.4	173.0	2115	1.538(3)	1.125(2)

a H_3_B(CO) C,^34^ (F_2_B)_3_B(CO) D,^7^ (Cl_2_B)_3_B(CO) E,^35^ perfluoropentaphenyl-borole(CO) F,^36^ (CF_3_)_3_B(CO) G,^37^ (C_2_F_5_)_3_B(CO) H,^38^ [borole(^Me^NHC)(CO)]^+^I [Ph* = 3,5-*t*Bu_2_(C_6_H_3_)].^39^

b
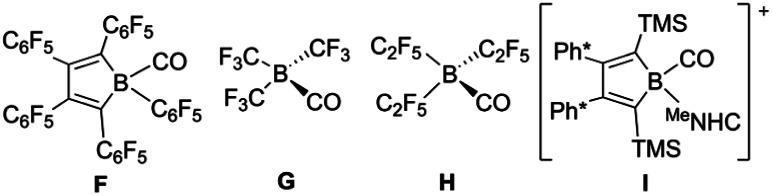

The best preparative results were obtained by first freezing the boradigermaallyl and phenylacetylene at −38 °C in cyclohexane and only allowing the mixture to warm up to room temperature after replacing the argon atmosphere by CO. Freezing prevents the premature, undesired reaction of both reactants with each other. The CO adduct 6 was isolated as colorless crystals in a yield of 70%. Phenylacetylene addition at a Ge–B bond was observed and the CH addition reaction was prevented. Compound 6 shows at room temperature a subsequent slow insertion reaction of the CO molecule into a B–C bond (*vide infra*). To avoid the CO insertion reaction, 6 must be stored at −38 °C. CO adducts of boranes are rather rare substances, and analytical data of these adducts are listed in Table 1.^7,35–39,41,42^

In the ^11^B NMR spectrum a signal at −23.2 ppm was observed for the tetracoordinate boron atom in 6. The literature examples of B–CO adducts were found to show ^11^B NMR signals at a comparably low frequency (Table 1). The stretching frequency for the CO group of 6 was observed in the IR spectrum at 2077 cm^−1^ (Table 1). The observed ^13^C NMR signal of the CO unit of 6 at 181.6 ppm lies at a slightly higher frequency in comparison to the published examples. In the molecular structure of 6 shown in Fig. 2 the carbonyl group exhibits the shortest B–C and longest C–O bond in the series of adducts, consistent with the smallest IR stretching frequency for 6 underpinning the tendency found for 6 to show the highest amount of π-backbonding to the CO molecule in the presented series (Table 1). Based on DFT calculations and NBO analysis the π-backbonding observed in the B–CO unit of 6 can be rationalized as an effect of hyperconjugation of the two Ge–B bonds into a π*-orbital.^43–57^

The bond lengths of the unsaturated GeBC_2_ ring (6, Fig. 2) can be compared with an intramolecular germylene/borane Lewis pair {[*t*BuC(NCy)_2_]GeC(Ph)C(C_6_F_5_)B(C_6_F_5_)_2_} featuring also a GeBC_2_-ring with a C–C double bond and only slightly different bond lengths [Ge–B 2.160(2), C–C 1.359(3), B–C 1.659(3), Ge–C 1.935(2) Å].^58^ The Ge–B interatomic distances in 6 are within the range of Ge–B single bonds found in the literature.^59–62^

Since the ^1^H and ^11^B NMR spectra revealed the slow formation of a new reaction product at room temperature in solution, a solution of 6 was heated to 50 °C for 44 h to complete this subsequent reaction more quickly (Scheme 4). The color of the solution changed from colorless to yellow and finally orange. The signal in the ^11^B NMR spectrum also changed from −23.2 to 10.1 ppm. Single crystals of 7 were obtained from *n*-pentane overnight, the molecular structure of which is shown in Fig. 2. The CO group is inserted into the B–C(vinyl) bond to give an α,β-unsaturated acylboron compound 7 while the chloride ligand migrated simultaneously from the germanium to the boron atom. The bond lengths in the α,β-unsaturated acylboron moiety [C5–C6 1.350(3), C6–C8 1.466(2), C8–B 1.605(3), C8–O2 1.275(2) Å] are close to the values documented for examples found in the literature.^63,64^ The oxygen atom of the acyl group exhibits coordination to the germanium atom O2-Ge(2) 2.0098(13) Å, which is comparable with acetamide [2.0145(14) Å] and carboxylate coordination [2.045(1), 2.043(1) Å] at germanium atoms.^65–67^ The Ge–B bond lengths in 7 are similar to single bonds between these elements.^59–62^ For a rare example of a borane–CO adduct and a subsequent carbon monoxide insertion, we can present the molecular structures and analytical data for both compounds, the adduct and insertion product. The observed reaction sequence (Scheme 4) can be compared with the findings of Piers *et al.* who described the reaction of pentaphenylborole with carbon monoxide to give a CO adduct at −78 °C, followed by room temperature ring expansion insertion of the CO group into a B–C bond.^36^ Carbonylations of organoboranes have been known for a long time and are important reactions in organic synthesis.^68–70^ These reactions were postulated to proceed by alkyl group migration from the boron atom to the carbon atom of the coordinated CO group.^68,70^ CO insertion into B–C bonds without detection of a CO coordination intermediate were reported for α-borylated phosphorus ylides, and zwitterionic titanium and platinum borate complexes.^71–74^ Using frustrated Lewis pairs (FLP) reactions of [HB(C_6_F_5_)_2_] with CO give formylborane, presumably *via* CO insertion into a boron–hydrogen bond.^75–77^ Aldridge and coworkers employed a trapping strategy to isolate a CO adduct of a BNB bis-borane FLP in reaction with tri(*tert*butyl)phosphine. This approach revealed the reversible uptake of CO and the reversible migration of an aryl group (C_6_F_5_) between boron and the carbon monoxide carbon atom.^78^ Acylboranes represent an important target in organic synthesis and a variety of synthetic pathways have been established.^79–81^ However, coordination of carbon monoxide followed by insertion of the CO moiety into a B–C bond has not been developed as a preparative route for the synthesis of acylboranes.

Interestingly, computational analysis of the relative stabilities of 6 and 7 suggests that the observed reactivity is driven by the two bulky terphenyl substituents, and not the consequence of the inherent reactivity of the GeC_2_B(CO) moiety. Replacing the two bulky C_6_H_3_-2,6-(Trip)_2_ groups by hydrogen or phenyl results in 7 being higher in energy than 6 by 8 and 6–10 kcal mol^−1^, respectively, using different density functional methods without and with correction for London dispersion as well as *ab initio* methods (see ESI†).

**Scheme 5:**
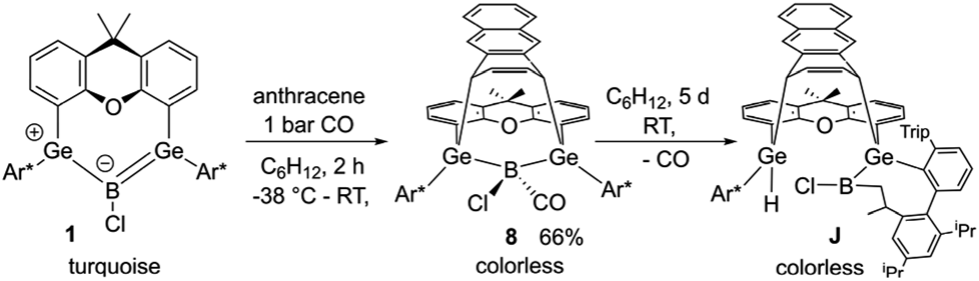
Anthracene reaction under an atmosphere of CO.

**Fig. 3:**
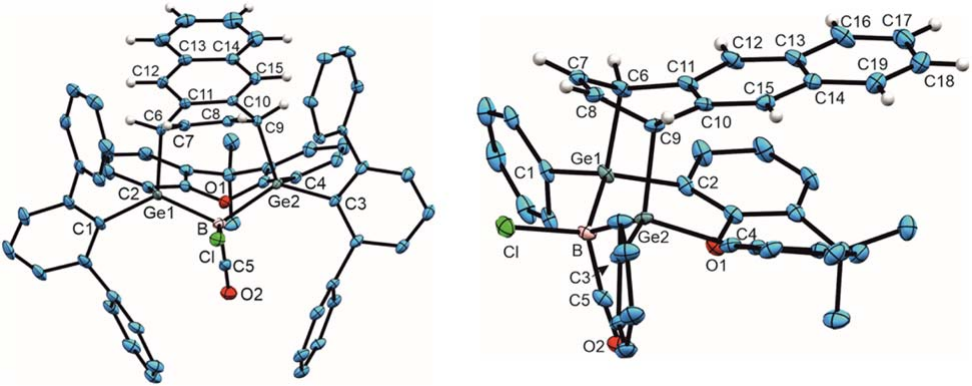
ORTEP of the molecular structure of 8. Thermal ellipsoids are shown at 50% probability level. Hydrogen atoms and iPr groups (left picture) or Trip groups (right picture) except anthracene H-atoms have been omitted. Selected interatomic distances [Å] and angles [°]: B–Ge1 2.145(2), B–Ge2 2.137(2), B–Cl 1.842(2), B–C5 1.538(3), C5–O2 1.125(2), C6–Ge1 2.0247(19), C9–Ge2 2.0243(18), C1–Ge1 2.0066(19), C2–Ge1 1.970(2), C3–Ge2 2.0084(18), C4–Ge2 1.968(2), C7–C8 1.335(3), C6–C7 1.494(3), C8–C9 1.496(3), C6–C11 1.509(3), C9–C10 1.507(3), C10–C11 1.428(3), C10–C15 1.378(3), C11–C12 1.373(3), C12–C13 1.416(3), C13–C14 1.419(3), C14–C15 1.415(3), Ge1–B–Ge2 115.4(1), B–C5–O2 173.8(2), C8–C9–C10 111.5(2), C7–C6–C11 111.5(2).

Coordination of CO to preclude follow-up reactions can also be employed in other reactions of 1. Recently, we discovered that treating boradigermaallyl with anthracene results in a (4 + 3) cycloaddition followed by a CH addition reaction involving a GeB unit to give compound J (Scheme 5).^22^ In the presence of a CO atmosphere (Scheme 5), the (4 + 3) cycloaddition product of 1 with anthracene was isolated as a CO adduct 8 (Fig. 3) and the CH addition reaction was prevented. Storing compound 8 at room temperature gradually leads to the formation of product J after decarbonylation as determined by NMR spectroscopy. The ^11^B NMR signal at −21.4 ppm and the ^13^C NMR signal at 173.0 ppm found for 8 are close to the signals of 6 (Table 1). Together with the B–CO interatomic distances (Table 1) in comparison to 6 a reduced π-backbonding was observed for 8. The Ge–C distances with the anthracene molecule [C6–Ge1 2.0247(19), C9–Ge2 2.0243(18) Å] are only slightly longer than the distances between the germanium atoms and the other aryl-groups [C1–Ge1 2.0066(19), C2–Ge1 1.970(2), C3–Ge2 2.0084(18), C4–Ge2 1.968(2) Å].

**Scheme 6:**
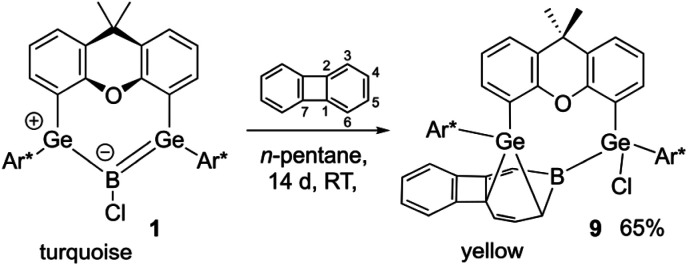
Biphenylene reaction with boradigermaallyl.

We were also interested in the reactivity of boradigermaallyl towards biphenylene, as biphenylene has already been used for comparative reactivity studies against low-valent main group element compounds.^82–87^ Kinjo *et al.* reported an oxidative addition of a cyclic Al(i) anion into the weakest C–C bond, a bridging C1–C7 bond in the four membered cycle, of biphenylene (see Scheme 6 for numbering).^83^ The same group published the reaction of a dianionic dialane with biphenylene to yield a reduction product of biphenylene featuring a C–C bond split and formation of two Al–C bonds.^84^ Crimmin *et al.* found chemoselective C–C bond activation treating an aluminium(i) compound [{HC(CMeNAr)_2_}Al] (Ar = 2,6-iPr_2_C_6_H_3_)^85^ with biphenylene.^86^ In the first step of this reaction at room temperature a (1 + 4) cycloaddition intermediate was formed over 7 days, which was thermolyzed for 3 h at 100 °C to yield a mixture of two double aluminium insertion products into the less reactive phenyl moiety of biphenylene.^86^ Liu and coworkers reported an Al(i)-insertion reaction into a phenyl moiety of biphenylene at 100 °C treating an NHC-adduct of an aluminylene compound.^87^

**Fig. 4:**
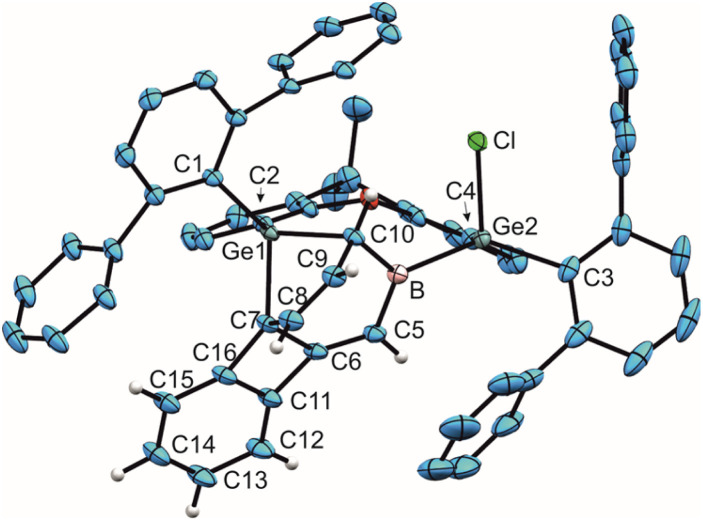
ORTEP of the molecular structure of 9. Thermal ellipsoids are shown at 50% probability level. Hydrogen atoms and iPr groups except biphenylene H-atoms have been omitted. Selected interatomic distances [Å] and angles [°]: B–C5 1.533(3), B–C10 1.574(3), B–Ge2 2.081(2), Ge1–C7 2.0099(18), Ge1–C10 2.0105(19), C5–C6 1.339(3), C6–C7 1.551(2), C7–C8 1.508(3), C8–C9 1.339(3), C9–C10 1.522(2), C5–B–Ge2 115.4(1), C5–B–C10 121.0(2), C10–B–Ge2 122.7(1).

When the boradigermaallyl was treated with one equivalent of biphenylene at room temperature a slow change in color was observed, shifting from turquoise to green and finally orange over the course of two weeks. Crystallization from *n*-pentane gave the insertion product in 65% yield. Boradigermaallyl does not show a reaction with the weak bonds of the four membered ring in biphenylene. In contrast to the low valent aluminium species (*vide supra*), which inserts at 100 °C into the phenyl moiety, the low valent boron compound 1 reacts with biphenylene at ambient temperature. Like in the aluminium insertion reported by Liu *et al.* boron splits the C3–C4 bond of biphenylene (Scheme 6 and Fig. 4).^87^

Compound 9 is reminiscent of the benzene reaction product of 1, exhibiting also insertion of the boron atom into the phenyl ring followed by (2 + 4) cycloaddition of a germylene moiety.^22^ The bond lengths inside the seven membered ring of 9 are comparable with the distances found in the boradigermaallyl–benzene reaction product.^22^ The signal in the ^11^B NMR spectrum was observed in the solid state at 71 ppm, very close to the signal for the benzene reaction product with 1, which was found in the solid state at 69 ppm.^22^

## Conclusions

Boradigermaallyl with two stabilizing bulky C_6_H_3_-2,6-(Trip)_2_ (Trip = 2,4,6-C_6_H_2_iPr_3_) groups exhibits high reactivity towards unsaturated organic molecules featuring C=C double or C≡C triple bonds. While ethylene reacts *via* a threefold (2 + 2) cycloaddition reaction with the GeB units of the unsaturated low valent [Ge=B–Ge] boron compound, styrene and phenylacetylene show addition of only one molecule. In the styrene case, addition at the germanium atoms was observed followed by CH insertion of a methyl group of the terphenyl substituent at a GeB unit. A mixture of two products was observed in reaction with phenylacetylene. (2 + 2) cycloaddition reactions at the germanium atoms and B=Ge unit were found to give products of *cis* substituted olefins. These addition reactions were also accompanied by an undesired CH addition of a methyl group of the terphenyl ligand with a GeB unit, which was suppressed by running the phenylacetylene addition reaction under an atmosphere of carbon monoxide. Instead, an interesting series of reactions was found, formation of a carbon monoxide borane adduct of the (2 + 2) cycloaddition product in a reasonable yield of 70% followed by selective insertion of carbon monoxide into the B-vinyl bond to give an acyl borane. Also, in the case of the anthracene addition product with boradigermaallyl carbon monoxide coordination at boron prevents the CH addition at the GeB unit. The reversibility of the CO coordination has been verified, as upon CO release the CH addition at the GeB unit commences. Toward biphenylene, boradigermaallyl shows high reactivity and selectively inserts at room temperature into a C–C bond of a phenyl moiety. The high reactivity of boradigermaallyl observed toward unsaturated organic molecules makes further studies worthwhile, for example with organic heterocycles.

## Data availability

Full experimental and computational details are provided as part of the ESI.†

## Author contributions

Investigations, writing, review R. H. K.; preparation of 3–6 N. H.; special NMR experiments K. E.; X-ray measurements and structure determinations H. S.; computational investigations C. T.; supervision, funding acquisition, computational investigations, manuscript writing and review H. F. B.; supervision, funding acquisition, computational investigations, manuscript writing and review L. W.

## Conflicts of interest

There are no conflicts to declare.

## Supplementary Material

SC-016-D5SC00881F-s001

SC-016-D5SC00881F-s002
