# Supplementary material for: Boradigermaallyl: inhibition of CH bond activation by borane CO adduct formation followed by CO insertion
Source: Chem Sci. 2025 Mar 27;16(18):7759–65. doi: 10.1039/d5sc00881f (PMC11966537; doi:10.1039/d5sc00881f)
Supplement: SC-016-D5SC00881F-s001 [file SC-016-D5SC00881F-s001.pdf]

Supplementary Information (SI) for Chemical Science.
This journal is © The Royal Society of Chemistry 2025



Supporting Information

# Boradigermaallyl : inhibition of CH bond activation by borane CO adduct formation followed by CO Insertion

Ralf H. Kern,[a] Noemi Hiller,[a] Klaus Eichele,[a] Hartmut Schubert,[a] Christina Tönshoff,[b] Holger F. Bettinger,[b] Lars Wesemann*[a]

[a] R. H. Kern, N. Hiller, Dr. K. Eichele, Dr. H. Schubert, Prof. Dr. L. Wesemann
Institut für Anorganische Chemie
Eberhard Karls Universität Tübingen
Auf der Morgenstelle 18, 72076 Tübingen, Germany
E-mail: lars.wesemann@uni-tuebingen.de

[b] Dr. C. Tönshoff, Prof. Dr. H. F. Bettinger
Institut für Organische Chemie
Eberhard Karls Universität Tübingen
Auf der Morgenstelle 18, 72076 Tübingen, Germany

## Content

Experimental....2
Crystal structure determination....15
NMR spectroscopy....20
  NMR spectra of compound **2**....20
  NMR spectra of compound **3**....27
  NMR spectra of compound **6**....34
  NMR spectra of compound **7**....43
  NMR spectra of compound **8**....51
  NMR spectra of compound **9**....60
IR-spectroscopy....71
UV-Vis spectroscopy....74
Computational Methods....75
References....120

# Experimental

**General procedures:** All manipulations were carried out under argon atmosphere using standard Schlenk techniques and gloveboxes. Benzene was dried with activated aluminium oxide, *n*-pentane and *n*-hexane were obtained from a MBraun solvent purification systems (SPS). All other solvents ($Et_2O$, THF, toluene, benzene-$d_6$, cyclohexane-$d_{12}$) were distilled from a sodium-potassium alloy and like the previous mentioned solvents subsequently degassed by three freeze–pump–thaw cycles. Starting material boradigermaallyl (**1**) was prepared according to a literature procedure.[1] For the reaction with $^{13}$C-labeled carbon monoxide, $^{13}$CO from Sigma-Aldrich with an isotopic distribution of 99.0 % $^{13}$C and 12 % $^{18}$O was commercially purchased. Further chemicals were purchased commercially and used as received.

**Elemental analysis:** Elemental analysis was performed at the Institute of Inorganic Chemistry, University of Tübingen using an *elementar* vario MICRO Cube.

**NMR spectroscopy:** NMR spectra were recorded with either a Bruker Avance III HD 300 NanoBay spectrometer equipped with a 5 mm BBFO probe head and operating at 300.13 ($^1$H) and 96.29 ($^{11}$B) MHz, a Bruker AVII+400 NMR spectrometer equipped with Brukers 5 mm QNP (quad nucleus probe) or a 5 mm BBFO probe head operating at 400.11 ($^1$H), 100.61 ($^{13}$C) and 128.37 ($^{11}$B) MHz, a Bruker Avance III HDX 600 spectrometer with a 5 mm Prodigy BBO cryo probe head operating at 600.13 ($^1$H) and 150.90 ($^{13}$C) or a Bruker Avance III HDX 700 NMR spectrometer equipped with a 5 mm TXI probe head operating at 700.29 ($^1$H) and 176.9 ($^{13}$C) MHz. Chemical shifts are reported in $\delta$ values in ppm relative to external $SiMe_4$ ($^1$H, $^{13}$C) or $BF_3 \cdot OEt_2$ ($^{11}$B) referenced in most cases on the solvent $^2$H resonance frequency as follows: Ξ = 25.145020 % for $^{13}$C, Ξ = 32.083 974 % for $^{11}$B. The multiplicity of the signals is indicated as s = singlet, d = doublet, t = triplet, sept = septet, m = multiplet or br = broad/unresolved. For the assignment of proton and carbon signals detailed analysis of $^1$H, $^{13}$C{$^1$H}, $^1$H–$^1$H COSY, $^1$H–$^{13}$C HSQC, $^1$H–$^{13}$C HMBC and $^{13}$C{$^1$H} DEPT 135 spectra was done.

Powdered sample of **9** was packed into 4 mm o.d. zirconia rotors under the inert atmosphere of a glove box. Solid-state NMR spectra were obtained on a Bruker Avance III HD 300 wide-bore NMR spectrometer ($B_0$ = 7.05 T) operating at 300.13 ($^1$H) and 96.29 ($^{11}$B) MHz. Spectra were acquired under high-power proton decoupling (hpdec) using a background suppression sequence (zgbsig) to eliminate the contributionof the stator material, employing a selective “90°” pulse on the central transition (half of solution 90° pulse). . Referencing against Ξ = 32.083974 % ($^{11}$B)[2] was achieved by the substitution method: an external sample of $CHCl_3$ in acetone in a zirconia rotor was spun at 1.5 kHz and the external magnetic field was adjusted such that the $^1$H chemical shift of $CHCl_3$ matched a predetermined chemical shift wrt. external 1% TMS in $CHCl_3$. The 4 mm double-bearing-double resonance probe head produced a strong $^{11}$B background signal (Fig. SI26) that was eliminated by acquiring and subtracting the spectrum of an empty rotor. Simulation of spectra was achieved using the sola module of Bruker TopSpin 4.1.4.

**Crystallography:** X-ray data were collected with a Bruker Smart APEX II diffractometer with graphite-monochromated Mo-$K_\alpha$ radiation. The programs used were Bruker’s APEX2 v2011.8-0, including SAINT for data reduction, SADABS for absorption correction, and SHELXS for structure solution, as well as the WinGX suite of programs version 1.70.01 or the GUI ShelXle, including SHELXL for structure refinement.[3-9]

**UV/Vis Spectroscopy:** Visible UV/Vis absorption spectra were recorded on PerkinElmer Lambda 35 spectrophotometer in gas tight 1 cm quartz cuvettes sealed with Teflon stoppers or Teflon lined screw caps.

**IR spectroscopy.** The IR spectra were recorded as potassium bromide pellets, which were prepared in an MBraun glovebox and measured with a Bruker VERTEX 70 IR spectrometer. In addition, IR spectra were measured with an ATR unit on the same device.

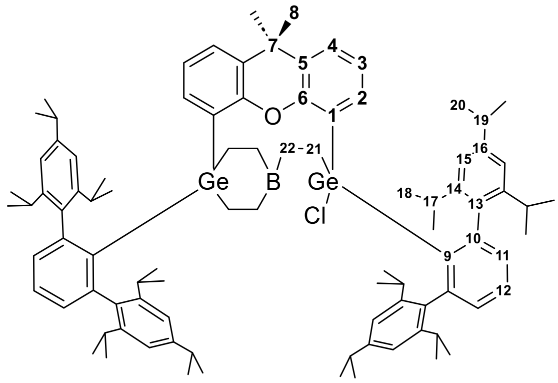


**Synthesis of compound 2**: A turquoise solution of boradigermaallyl **1** (80.0 mg, 58.7 µmol, 1.00 equiv.) in *n*-pentane (5.00 ml) was transferred to a Schlenk tube with a volume of approximately 50 ml and subsequently degassed with one “freeze-pump-thaw” cycle. The argon atmosphere was exchanged for 1 bar ethylene and the solution was then stirred for 2 days at room temperature. A decolorization of the reaction solution already occurred after 60 minutes, however, a complete turnover was not achieved until after 2 days. In the next step, small, suspended solids in the colorless to mint-green solution were filtered off and the solvent was removed under reduced pressure. The residue was dissolved in $Et_2O$ and the solution was concentrated by partial evaporation of the solvent under reduced pressure. Crystallization over a period of one week at —38 °C led to colorless crystals of the product **2** suitable for X-ray diffraction (37.3 mg, 25.8 µmol, 44 %).

**$^1$H-NMR** (700.21 MHz, tol-d$_8$, 255 K): $\delta$ [ppm] = —0.06 (d, 1H, $^2J_{HH}$ = 11.0 Hz, H-21), 0.39 – 0.46 (m, 2H, H-21 (1H) + H-22 (1H)), 0.53 (d, 3H, $^3J_{HH}$ = 6.5 Hz, H-18), 0.71 – 0.83 (m, 2H, H-22), 0.85 – 0.91 (m, 2H, H-21 (1H) + H-22 (1H)), 0.92 –0.96 (m, 4H, H-18 (3H) + H-22 (1H)), 0.97 (d, 3H, $^3J_{HH}$ = 6.7 Hz, H-18), 1.00 – 1.04 (m, 1H, H-22) 1.07 (d, 3H, $^3J_{HH}$ = 6.7 Hz, H-18), 1.10 – 1.13 (m, 6H, H-18), 1.16 (d, 3H, $^3J_{HH}$ = 6.7 Hz, H-18), 1.18 – 1.25 (m, 21H, H-18 (6H) + H-20 (15H)), 1.25 – 1.28 (m, 4H, H-18 (3H) + H-21 (1H)), 1.29 – 1.31 (m, 6H, H-8 (3H) + H-20 (3H)), 1.33 (d, 3H, $^3J_{HH}$ = 6.9 Hz, H-20), 1.34 – 1.37 (m, 6H, H-18 (3H) + H-20 (3H)), 1.37 – 1.41 (m, 6H, H-18), 1.44 – 1.47 (m, 6H, H-8 (3H) + H-18 (3H)), 1.58 (d, 3H, $^3J_{HH}$ = 6.9 Hz, H-18), 1.65 – 1.70 (m, 1H, H-21), 1.73 (d, 3H, $^3J_{HH}$ = 6.6 Hz, H-18), 1.80 – 1.84 (m, 1H, H-21), 2.49 – 2.59 (m, 1H, H-17), 2.73 – 2.84 (m, 4H, H-17 (1H) + H-19 (3H)), 2.92 – 3.00 (m, 2H, H-17 (1H) + H-19 (1H)), 3.09 (sept, 1H, $^3J_{HH}$ = 6.7 Hz, H-17), 3.20 – 3.30 (m, 2H, H-17), 3.30 – 3.38 (m, 1H, H-17), 3.43 (sept, 1H, $^3J_{HH}$ = 6.7 Hz, H-17), 6.38 (t, 1H, $^3J_{HH}$ = 7.4 Hz, H-3), 6.69 (t, 1H, $^3J_{HH}$ = 7.4 Hz, H-3), 6.72 – 6.77 (m, 2H, H-2 (1H) + H-15 (1H)), 6.89 (dd, 1H, $^3J_{HH}$ = 7.7 Hz, $^4J_{HH}$ = 1.2 Hz, H-4), 6.95 (s, br, 1H, H-15), 6.96 – 6.98 (m, 2H, H-11 (1H) + H-12 (1H), overlapped by solvent signal), 7.02 (dd, 1H, $^3J_{HH}$ = 7.7 Hz, $^4J_{HH}$ = 1.3 Hz, H-4, overlapped by solvent signal), 7.06 – 7.09 (m, 3H, H-15), 7.09 – 7.11 (m, 1H, H-2, overlapped by solvent signal), 7.13 (s, br, 1H, H-15), 7.16 – 7.19 (m, 2H, H-11 (1H) + H-12 (1H)), 7.20 (s, br, 1H, H-15), 7.23– 7.26 (m, 2H, H-11), 7.31 (s, br, 1H, H-15). **$^{13}$C{$^1$H}-NMR** (176.07 MHz, tol-d$_8$, 255 K): $\delta$ [ppm] = 7.3 (br, C-21), 11.7 (br, C-21), 18.0 (br, C-22), 18.3 (br, C-21), 21.6 (C-18), 22.5 (C-18), 22.7 (C-18), 22.8 (C-18), 23.0 (C-18), 23.1 (C-22), 23.6 (C-18), 23.8 (C-20), 23.9 (C-22), 24.2 (C-18), 24.5 (C-20), 24.5 (C-20), 24.5 (C-20), 24.6 (C-20), 24.6 (C-20), 24.7 (C-20), 24.7 (C-18), 25.1

(C-18), 25.2 (C-20), 25.5 (C-18), 26.5 (C-18), 26.6 (C-18), 26.7 (C-18), 26.9 (C-18), 27.0 (C-18), 27.4 (C-18), 31.0 (C-17), 31.1 (C-17), 31.2 (C-17), 31.3 (2 x C-17), 31.4 (C-17), 31.5 (C-17), 32.2 (C-17), 33.9 (C-8), 34.1 (C-8), 34.5 (C-7), 34.7 (C-19), 34.8 (2x C-19), 34.9 (C-19), 120.3 (C-15), 120.4 (C-15), 120.7 (2x C-15), 120.8 (C-15), 121.4 (C-15), 121.8 (C-3 + 2x C-15), 122.4 (C-1), 123.0 (C-3), 126.8 (C-4), 127.4 (C-12), 127.8 (C-4, overlapped by solvent signal), 128.0 (C-12, overlapped by solvent signal), 128.9 (C-1, overlapped by solvent signal), 129.3 (2x C-5), 130.4 (C-11), 131.2 (C-11), 131.4 (C-11), 132.4 (C-11), 133.8 (C-2), 136.9 (C-2), 138.1 (C-13), 138.8 (C-13), 138.9 (C-13), 139.7 (C-9), 139.7 (C-13), 141.4 (C-9), 146.1 (C-14), 146.3 (3x C-14), 146.7 (C-10), 146.9 (C-10), 147.2 (C-14), 147.3 (C-14), 147.5 (C-14 + C-16), 147.6 (C-14), 148.1 (C-10), 148.1 (C-10), 148.3 (C-16), 148.3 (C-16), 149.5 (C-16), 153.8 (C-6), 154.7 (C-6). **$^{11}$B{$^1$H}-NMR**: No boron NMR signal could be observed in solution. **Elemental analysis calcd (%)** for $C_{93}H_{122}BClGe_2O$ + $Et_2O$: C 76.57, H 8.74; found: C 76.62, H 8.54.

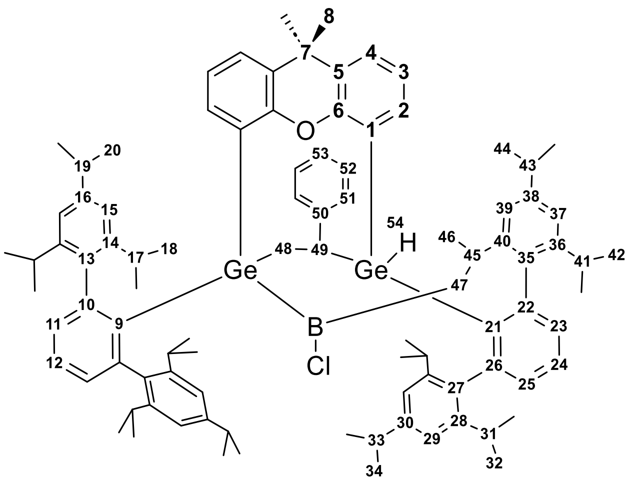


**Synthesis of compound 3**: Boradigermaallyl **1** (60.0 mg, 44.0 µmol, 1.00 equiv.) was dissolved in pentane (2.00 ml) and styrene (30.0 µl, 261 µmol, 5.92 equiv.) was added while stirring at room temperature. A color change from turquoise to brown-red was already visible within half an hour, however, the complete conversion of the reactants required one day of stirring at room temperature and resulted in a salmon-colored suspension. Volatile components were removed under reduced pressure and the colorless-pink residue was dissolved in $Et_2O$ (2.00 ml). By partially evaporating the solvent under reduced pressure, the concentration of the product in solution was increased and colorless crystals of product **3** suitable for X-ray structure analysis were obtained after three days of crystallization at room temperature (12.9 mg, 8.79 µmol, 20 %, 95 % purity). Due to the C-H bond activation, this compound was not the intended target of this work, and therefore no reaction optimization was carried out to increase yield or purity.

**$^1$H-NMR** (700.29 MHz, $C_6D_6$): $\delta$ [ppm] = −0.16 (d, 1H, $^2J_{HH}$ = 15.2 Hz, H-47), 0.28 (d, 3H, H-18), 0.46 – 0.51 (m, 1H, H-48), 0.82 – 0.85 (m, 6H, H-18 (3H) + H-32 (3H)), 0.89 – 0.92 (m, 6H, H-32 (3H) + H-20 (3H)), 0.92 – 0.95 (m, 9H, H-18 (3H) + H-42 (6H)), 0.97 (d, 3H, $^3J_{HH}$ = 7.0 Hz, H-20), 1.05 (d, 3H,

$^3J_{HH}$ = 6.8 Hz, H-18), 1.11 (d, 3H, $^3J_{HH}$ = 6.9 Hz, H-44), 1.15 (d, 3H, $^3J_{HH}$ = 6.8 Hz, H-44), 1.18 – 1.20 (m, 6H, H-8 (3H)) + H-18 (3H)), 1.22 – 1.24 (m, 1H, H-48), 1.31 (d, 3H, $^3J_{HH}$ = 7.0 Hz, H-46), 1.36 – 1.41 (m, 15H, H-18 (3H) + H-20 (3H) + H-32 (3H) + H-34 (6H)), 1.43 (d, 3H, $^3J_{HH}$ = 6.9 Hz, H-20), 1.45 (s, 3H, H-8), 1.64 (d, 3H, $^3J_{HH}$ = 6.9 Hz, H-18), 1.67 (d, 3H, $^3J_{HH}$ = 6.7 Hz, H-18), 1.72 – 1.76 (m, 1H, H-47), 1.78 (d, 3H, $^3J_{HH}$ = 6.9 Hz, H-18), 2.36 (sept, 1H, $^3J_{HH}$ = 6.7 Hz, H-41), 2.45 (sept, 1H, $^3J_{HH}$ = 7.0 Hz, H-19), 2.64 (sept, 1H, $^3J_{HH}$ = 6.9 Hz, H-43), 2.95 (sept, 1H, $^3J_{HH}$ = 7.0 Hz, H-33), 2.99 – 3.06 (m, 2 H, H-19, H-17), 3.10 (sept, 1H, $^3J_{HH}$ = 6.8 Hz, H-17), 3.18 (sept, 1H, $^3J_{HH}$ = 6.8 Hz, H-31), 3.27 (s, 1H, H-54), 3.29 – 3.40 (m, 4H, H-17 (2H) + H-45 (1H) + H-49 (1H)), 3.61 (sept, 1H, $^3J_{HH}$ = 6.7 Hz, H-31), 5.46 – 5.49 (m, 2H, H-51), 6.47 (dd, 1H, $^3J_{HH}$ = 7.2 Hz, $^4J_{HH}$ = 1.1 Hz, H-2), 6.52 – 6.55 (m, 2H, H-52), 6.55 – 6.58 (m, 2H, H-3 (1H) + H-37 (1H)), 6.62 (d, 1H, $^4J_{HH}$ = 1.6 Hz, H-15), 6.63 – 6.66 (m, 1H, H-53), 6.72 (t, 1H, $^3J_{HH}$ = 7.4 Hz, H-3), 6.81 (dd, 1H, $^3J_{HH}$ = 7.3 Hz, $^4J_{HH}$ = 1.1 Hz, H-2), 6.93 – 6.97 (m, 4H, H-4 (2H) + H-15 (1H) + H-29 (1H)), 6.97 – 7.00 (m, 2H, H-23 (1H) + H-24 (1H)), 7.01 – 7.03 (m, 1H, H-25), 7.08 – 7.11 (m, 1H, H-12), 7.13 (d, 1H, $^4J_{HH}$ = 1.5 Hz , H-39), 7.14 – 7.15 (m, 1H, H-11, overlapped by solvent signal), 7.22 (dd, 1H, $^3J_{HH}$ = 7.4 Hz, $^4J_{HH}$ = 1.5 Hz, H-11), 7.30 (d, 1H, $^4J_{HH}$ = 1.6 Hz , H-15), 7.35 (d, 1H, $^4J_{HH}$ = 1.6 Hz, H-15), 7.43 (d, 1H, $^4J_{HH}$ = 1.6 Hz, H-29). **$^{13}$C-NMR** (176.10 MHz, $C_6D_6$): $\delta$ [ppm] = 21.9 (C-32), 22.5 (C-18), 23.0 (C-42), 23.0 (C-44), 23.3 (C-18), 23.3 (C-18), 23.7 (C-20), 24.1 (C-34), 24.2 (C-20), 24.4 (C-20), 24.5 (C-34), 24.6 (C-20), 24.6 (C-18), 24.7 (C-44), 25.1 (C-32), 25.5 (C-8 + C-18), 25.7 (C-18), 26.3 (C-32), 26.4 (C-18), 26.8 (C-18), 27.0 (C-42), 27.2 (C-48), 27.2 (C-32), 28.2 (C-46), 30.6 (C-31 + C-41 + C-17), 31.0 (C-17), 31.3 (C-31), 31.3 (C-17), 31.5 (C-17), 33.3 (C-8), 33.5 (C-43), 34.0 (C-19), 34.1 (C-19), 34.2 (C-45), 34.5 (C-33), 35.3 (C-7), 36.2 (C-49), 36.7 (C-47), 120.1 (C-37), 120.5 (C-15), 120.8 (C-15), 121.1 (C-15), 121.8 (C-39), 122.0 (C-29), 122.1 (C-15 + C-29), 123.0 (C-3), 123.4 (C-3), 124.2 (C-53), 124.7 (C-4), 125.7 (C-4), 126.2 (C-1), 126.7 (C-12), 127.3 (2x C-52), 128.1 (C-24, overlapped by solvent signal), 128.2 (2x C-51, overlapped by solvent signal), 130.1 (C-11), 130.5 (C-5), 130.7 (C-5), 131.7 (C-25), 132.5 (C-11 + C-23), 132.8 (C-2), 134.0 (C-1), 135.3 (C-2), 137.7 (C-21), 138.0 (C-35), 139.5 (C-13), 139.9 (C-27), 141.6 (C-13), 142.9 (C-9), 144.0 (C-50), 145.6 (C 14), 146.4 (C-28), 146.6 (C-36), 147.4 (C-14), 147.4 (C-40), 147.7 (C-26), 147.9 (C-28), 148.1 (C-10), 148.1 (C-38), 148.2 (C-16), 148.2 (C-14), 148.3 (C-14), 148.5 (C-16), 148.8 (C-22), 148.9 (C-30), 149.3 (C-10), 153.8 (C-6), 154.1 (C-6). **$^{11}$B{$^1$H}-NMR**: No boron NMR signal could be observed in solution. **An elemental analysis was not carried out due to insufficient purity.**

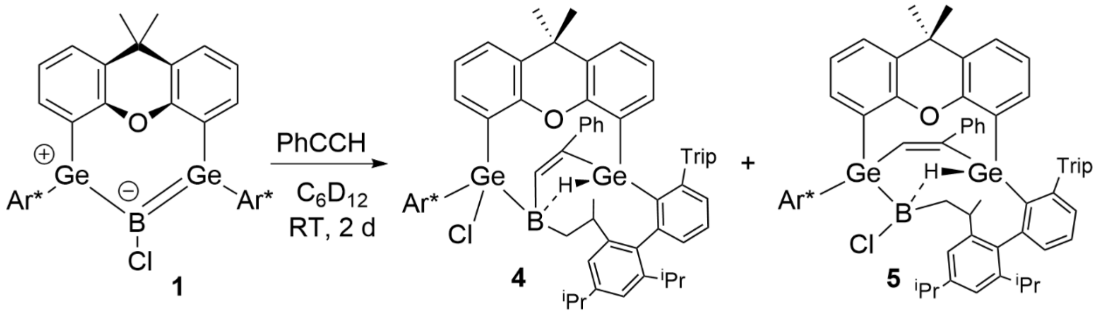


Scheme S1. Phenylalkyne reaction with **1**.

**Synthesis of a mixture of compounds 4 and 5**:Boradigermaallyl **1** (60.0 mg, 44.0 μmol, 1.00 equiv.) was dissolved in *n*-pentane (2.00 ml) and phenylacetylene (4.83 μl, 44.0 μmol, 1.00 equiv.)) was added while stirring at room temperature Over a period of three days, a color change from turquoise to pale yellow and a complete conversion of the reactants was observed. The solvent was then removed under reduced pressure and the residue was dissolved in $Et_2O$ (8.00 ml). The concentration was increased by partial evaporation of the solvent under reduced pressure and the products **4** and **5** were isolated over a period of three days using fractional crystallization at room temperature.

In the crystallization process, two types of colorless crystals, both suitable for X-ray structure analysis, were obtained in two fractions in different ratios. Due to the C-H bond activation, the compounds were not the intended targets of this work and therefore no reaction optimization was carried out. In addition, it was not possible to separate the two compounds, for which reason the yield and purity were not reported. Due to the extremely complex NMR spectra and the inability to separate the compounds from each other, no NMR characterization is provided.

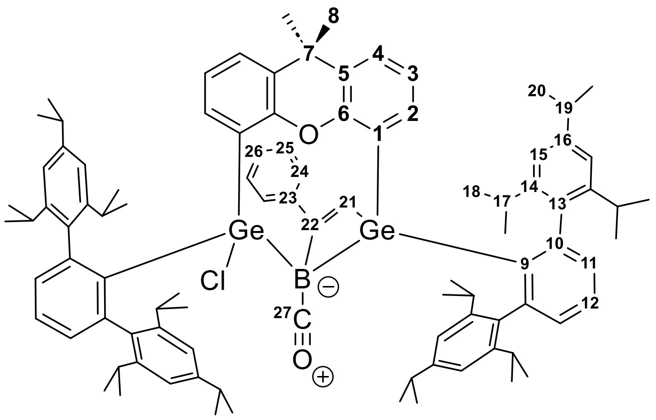


**Synthesis of compound 6**: Boradigermaallyl **1** (100 mg, 73.4 µmol, 1.00 equiv.) was dissolved in cyclohexane (2.00 ml) and then frozen at −38 °C inside a Schlenk tube with a volume of approximately 50 ml. Afterwards, phenylacetylene (8.06 µl, 73.4 µmol, 1.00 equiv.) that was already dissolved in cyclohexane (0.20 ml) was added to the Schlenk tube in such a way that the solution froze on the cold glass wall, while avoiding contact with the frozen turquoise solid of the boradigermaallyl. Subsequently, the argon atmosphere of the Schlenk tube was removed under reduced pressure and replaced with carbon monoxide. The entire frozen Schlenk tube was then brought to room temperature and the resulting turquoise solution was stirred for 4 h, resulting in a color change from turquoise to green to light yellow. The solvent was then removed under reduced pressure and the yellow residue was suspended in pentane. The resulting suspension had an orange-brown color from which product **6** precipitates at −38 °C as a colorless solid (76.1 mg, 51.0 µmol, 70 %). Colorless crystals suitable for X-ray diffraction were obtained from a concentrated $Et_2O$ solution of **6** at −38 °C after three days of crystallization. The compound continues to react at room temperature over the course of a few hours and should be stored at −38 °C.

**$^1$H-NMR** (700.29 MHz, $C_6D_6$, 283 K): $\delta$ [ppm] = − 0.21 (d, 3H, $^3J_{HH}$ = 6.8 Hz, H-18), 0.36 (d, 3H, $^3J_{HH}$ = 6.6 Hz, H-18), 0.86 – 0.89 (m, 3H, H-18), 0.92 (d, 3H, $^3J_{HH}$ = 6.7 Hz, H-18), 0.93 (d, 3H, $^3J_{HH}$ = 6.9 Hz, H-18), 0.97 (d, 3H, $^3J_{HH}$ = 6.7 Hz, H-18), 1.01 (d, 3H, $^3J_{HH}$ = 6.7 Hz, H-18), 1.02 – 1.05 (m, 6H, H-18 (3H), H-20 (3H)), 1.09 – 1.11 (m, 6H, H-18 (3H), H-20 (3H)), 1.15 (d, 3H, $^3J_{HH}$ = 6.9 Hz, H-18), 1.21 (d, 3H, $^3J_{HH}$ = 6.9 Hz, H-18), 1.30 (d, 3H, $^3J_{HH}$ = 6.9 Hz, H-20), 1.32 (d, 3H, $^3J_{HH}$ = 6.7 Hz, H-18), 1.33 – 1.36 (m, 6H, H-18 (3H), H-20 (3H)), 1.37 (s, 3H, H-8), 1.40 (d, 3H, $^3J_{HH}$ = 6.9 Hz, H-20), 1.44 – 1.47 (m, 9H, H-8 (3H), H-18 (3H), H-20 (3H)), 1.49 – 1.51 (m, 6H, H-18 (3H), H-20 (3H)), 1.59 (d, 3H, $^3J_{HH}$ = 6.9 Hz, H-20), 1.76 (d, 3H, $^3J_{HH}$ = 6.8 Hz, H-18), 2.31 (sept., 1H, $^3J_{HH}$ = 6.7 Hz, H-17), 2.45 – 2.58 (m, 4H, H-17 (3H), H-19 (1H)), 2.87 (sept., 1H, $^3J_{HH}$ = 6.9 Hz, H-19), 2.97 (sept., 1H, $^3J_{HH}$ = 6.9 Hz, H-19), 3.10 (sept., 1H, $^3J_{HH}$ = 6.9 Hz, H-19), 3.46 (sept., 1H, $^3J_{HH}$ = 6.9 Hz, H-17), 3.66 (sept., 1H, $^3J_{HH}$ = 6.8 Hz, H-17), 3.71 (sept., 1H, $^3J_{HH}$ = 6.7 Hz, H-17), 3.93 (sept., 1H, $^3J_{HH}$ = 6.8 Hz, H-17), 5.81 (s, 1H, H-21), 6.49 (d, 1H, $^4J_{HH}$ = 1.5 Hz, H-15), 6.54 – 6.56 (m, 1H, H-2), 6.57 – 6.60 (m, 1H, H-3), 6.69 – 6.71 (m, 2H, H-3, H-15), 6.76 – 6.78 (m, 1H, H-4), 6.80 (d, 1H, $^4J_{HH}$ = 1.5 Hz , H-15), 6.95 – 6.97 (m, 1H, H-11), 7.00 – 7.04 (m, 7H, H-4 (1H), H-12 (2H), H-15 (1H), H-25 (2H), H-26(1H)), 7.13 – 7.15 (m, 3H, H-11 (1H), H-24 (2H)), 7.19 (d, 1H, $^4J_{HH}$ = 1.5 Hz, H-15), 7.22 (dd, 1H,

$^{3}J_{HH}$ = 7.6 Hz, $^{4}J_{HH}$ = 1.3 Hz, H-11), 7.24 (d, 1H, $^{4}J_{HH}$ = 1.5 Hz, H-15), 7.30 (d, 1H, $^{4}J_{HH}$ = 1.5 Hz, H-15), 7.32 (d, 1H, $^{4}J_{HH}$ = 1.5 Hz, H-15), 7.43 (dd, 1H, $^{3}J_{HH}$ = 7.8 Hz, $^{4}J_{HH}$ = 1.4 Hz, H-11), 7.47 (dd, 1H, $^{3}J_{HH}$ = 7.5 Hz, $^{4}J_{HH}$ = 1.2 Hz, H-2). **$^{13}$C-NMR** (176.10 MHz, $C_6D_6$, 283 K): $\delta$ [ppm] = 20.7 (C-18), 21.5 (C-18), 21.9 (C-18), 22.0 (C-18), 22.2 (C-18), 22.3 (C-20), 22.6 (C-18), 23.4 (C-8), 23.5 (C-20), 24.3 (C-20), 24.4 (C-18), 24.5 (C-20), 24.7 (C-20), 24.8 (C-18), 24.9 (2 x C-18), 25.0 (C-20) , 25.7 (C-20), 25.8 (C-20), 25.9 (C-18), 26.0 (C-18), 26.2 (C-18), 27.4 (C-18), 27.7 (C-18), 28.5 (C-18), 30.2 (C-8), 30.5 (C-17), 30.6 (C-17), 30.7 (C-17), 30.9 (C-17), 31.3 (C-17), 31.9 (C-17), 32.0 (C-17), 32.1 (C-17), 34.4 (C-19), 34.7 (C-19), 34.9 (C-19), 34.9 (C-19), 36.8 (C-7), 120.2 (C-15), 120.6 (C-15), 120.7 (C-15), 120.9 (C-15), 121.1 (C-15), 121.2 (C-15), 121.6 (C-15), 122.6 (C-15), 123.1 (C-3), 123.7 (C-3), 124.1 (C-4), 125.1 (C-4), 127.0 (C-12), 127.3 (2 x C-24), 127.5 (C-12), 127.5 (C-26), 127.9 (2 x C-25, overlapped by solvent signal), 130.1 (C-1), 130.5 (C-11), 131.5 (C-5), 132.6 (C-11), 133.9 (C-1), 134.2 (C-11), 134.3 (C-2), 135.0 (C-11), 135.3 (C-2), 135.3 (C-5), 137.7 (C-13), 138.1 (C-13), 138.9 (C-13), 139.1 (C-13), 139.9 (C-23), 141.5 (C-9), 141.6 (C-9), 145.1 (C-14), 145.5 (C-14), 146.1 (C-14), 146.9 (C-16), 147.0 (C-14), 147.1 (C-10), 147.5 (C-14 + C-10), 147.8 (C-10), 147.9 (C-14), 148.3 (C-10), 148.4 (C-16), 148.4 (C-16), 148.5 (C-16), 148.7 (C-14), 149.1 (C-14), 151.3 (C-22, br), 154.8 (C-21), 156.0 (C-6), 158.7 (C-6), 181.6 (C-27, br). **$^{11}$B-NMR** (128.37 MHz, $C_6D_6$): $\delta$ [ppm] = –23.2. **IR** (KBr, cm$^{-1}$): 2077 ($\nu_{C\text{-}O}$). **Elemental analysis cacld (%) for** $C_{96}H_{116}BClGe_2O_2$: C 77.21, H 7.83; found: C 77.57, H 7.95.

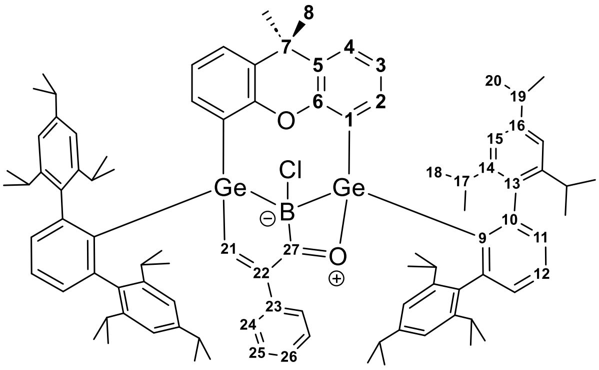


**Synthesis of compound 7**: A solution of boradigermaallyl **1** (100 mg, 73.4 µmol, 1.00 equiv.) in cyclohexane (3.00 ml) was frozen in a Schlenk tube with a volume of approximately 50 ml at –38 °C. A standard solution of phenylacetylene in cyclohexane (200 µl, 0.37 M in cyclohexane, 73.4 µmol, 1.00 equiv.) was added to the Schlenk tube in such a way that the solution froze on the cold glass wall while avoiding contact with the frozen turquoise solid of the boradigermaallyl. Subsequently, the argon atmosphere was removed under reduced pressure and replaced with one bar of carbon monoxide. In the next step, the Schlenk tube was warmed to room temperature and then heated to 50 °C for 44 hours, whereby a color change from turquoise to yellow and finally orange was visible. Following this, the solvent was removed under reduced pressure and the product was extracted using *n*-pentane (10 ml). Fine suspended particles were filtered off and the filtrate was concentrated by partial evaporation of the solvent under reduced pressure.

Crystallization overnight at room temperature led to yellow crystals of the product **7** suitable for X-ray diffraction (75.2 mg, 50.4 µmol, 69 %).

**$^1$H-NMR** (700.21 MHz, $C_6D_6$): $\delta$ [ppm] = 0.51 (d, 3H, $^3J_{HH}$ = 6.7 Hz, H-18), 0.56 (d, 3H, $^3J_{HH}$ = 6.7 Hz, H-18), 0.78 (d, 3H, $^3J_{HH}$ = 6.8 Hz, H-18), 0.90 (d, 3H, $^3J_{HH}$ = 6.8 Hz, H-18), 0.96 (d, 6H, $^3J_{HH}$ = 6.8 Hz, H-18 (3H) + H-20 (3H)), 0.99 – 1.01 (m, 6H, H-8 (3H) + H-18 (3H)), 1.01 (d, 3H, $^3J_{HH}$ = 6.9 Hz, H-20), 1.05 (d, 3H, $^3J_{HH}$ = 6.9 Hz, H-20), 1.18 (d, 3H, $^3J_{HH}$ = 6.9 Hz, H-20), 1.19 – 1.21 (m, 6H, H-18), 1.23 – 1.29 (m, 12H, H-8 (3H) + H-18 (3H) + H-20 (6H)), 1.31 (d, 3H, $^3J_{HH}$ = 6.9 Hz, H-20), 1.33 (d, 3H, $^3J_{HH}$ = 6.7 Hz, H-18), 1.36 – 1.39 (m, 6H, H-18 (3H) + H-20 (3H), 1.42 – 1.45 (m, 6H, H-18), 1.46 (d, 3H, $^3J_{HH}$ = 6.7 Hz, H-18), 1.57 (d, 3H, $^3J_{HH}$ = 6.7 Hz, H-18), 1.63 (d, 3H, $^3J_{HH}$ = 6.7 Hz, H-18), 2.50 (sept, 1H, $^3J_{HH}$ = 6.7 Hz, H-17), 2.56 (sept, 1H, $^3J_{HH}$ = 6.9 Hz, H-19), 2.60 (sept, 1H, $^3J_{HH}$ = 6.9 Hz, H-19), 2.84 – 2.91 (m, 2H, H-19), 3.11 (sept, 1H, $^3J_{HH}$ = 6.7 Hz, H-17), 3.42 (sept, 1H, $^3J_{HH}$ = 6.9 Hz, H-17), 3.54 – 3.64 (m, 3H, H-17), 3.68 (sept, 1H, $^3J_{HH}$ = 6.9 Hz, H-17), 3.84 (sept, 1H, $^3J_{HH}$ = 6.7 Hz, H-17), 6.36 (t, 1H, $^3J_{HH}$ = 7.6 Hz, H-3), 6.70 – 6.73 (m, 4H, H-3 (1H) + H-15 (1H) + H-24 (2H)), 6.82 (dd, 1H, $^3J_{HH}$ = 7.8 Hz, $^4J_{HH}$ = 1.3 Hz, H-4), 6.90 (d, 1H, $^4J_{HH}$ = 1.5 Hz, H-15), 6.97 – 7.02 (m, 7H, H-2 (1H) + H-4 (1H) + H-11 (1H) + H-15 (1H) + H-25 (2H) + H-26 (1H)), 7.03 – 7.05 (m, 2H, H-12 (1H) + H-15 (1H)), 7.12 (d, 1H, $^4J_{HH}$ = 1.6 Hz, H-15), 7.13 – 7.15 (m, 2H, H-11 (1H) + H-21 (1H)), 7.16 – 7.20 (m, 3H, H-2 (1H) + H-11 (1H) + H-12 (1H), overlapped by solvent signal), 7.21 (s, br, 2H, H-15), 7.27 (d, 1H, $^4J_{HH}$ = 1.7 Hz, H-15), 7.32 (dd, 1H, $^3J_{HH}$ = 7.0 Hz, $^4J_{HH}$ = 1.8 Hz, H-11). **$^{13}$C{$^1$H}-NMR** (176.07 MHz, $C_6D_6$): $\delta$ [ppm] = 21.5 (C-18), 22.2 (C-20), 22.9 (C-18), 23.0 (C-18), 23.4 (C-18), 23.4 (C-20), 23.6 (C-18), 23.8 (C-18 + C-20), 23.9 (C-18), 24.1 (C-20), 24.3 (C-20), 24.6 (C-20), 24.9 (C-20), 25.1 (C-18), 25.5 (C-18), 25.6 (C-18), 25.7 (C-18), 25.8 (C-20), 26.2 (C-18), 26.2 (C-18), 26.4 (C-18), 27.4 (C-18), 27.9 (C-18), 30.0 (C-8), 30.4 (C-17), 30.5 (C-17), 30.9 (2x C-17), 30.9 (C-8), 31.0 (C-17), 31.3 (C-17), 31.8 (C-17), 31.8 (C-17), 33.9 (C-19), 34.3 (C-19), 34.4 (2x C-19), 34.8 (C-7), 119.1 (C-15), 119.5 (C-15), 120.1 (C-15), 120.8 (C-15), 122.4 (C-15), 122.6 (C-15), 122.8 (C-15), 122.9 (C-3 + C-15), 123.0 (C-3), 125.1 (C-4), 125.4 (C-22), 127.1 (C-12), 127.5 (C-26), 127.7 (2x C-24), 127.8 (2x C-25), 127.9 (C-12, overlapped by solvent signal), 128.5 (C-4), 128.6 (C-1), 128.8 (C-1), 128.9 (C-5), 130.6 (C-5), 130.9 (C-11), 131.7 (C-11), 132.7 (C-11), 132.8 (C-11), 134.5 (C-2), 135.1 (C-23), 135.9 (C-2), 138.4 (C-13), 138.8 (C-13), 138.9 (C-9), 138.9 (C-13), 139.7 (C-13), 143.8 (C-9), 145.4 (C-14), 145.8 (C-10), 146.6 (C-14), 147.2 (C-14), 147.3 (C-14), 147.3 (C-14), 147.6 (C-14), 147.8 (C-14 + C-16), 147.9 (C-16), 148.1 (2x C-10), 148.3 (C-10), 148.5 (C-16), 149.4 (C-14), 149.4 (C-16), 151.8 (C-27), 152.3 (C-6), 155.6 (C-6), 181.9 (C-21). **$^{11}$B{$^1$H}-NMR** (128.37 MHz, $C_6D_6$): $\delta$ [ppm] = 10.1 (s). **IR** (ATR, cm$^{-1}$): 1401 ($\nu_{C-O}$). **Elemental analysis calcd (%)** for $C_{96}H_{116}BClGe_2O_2$: C 77.21, H 7.83; found: C 77.16, H 7.85.

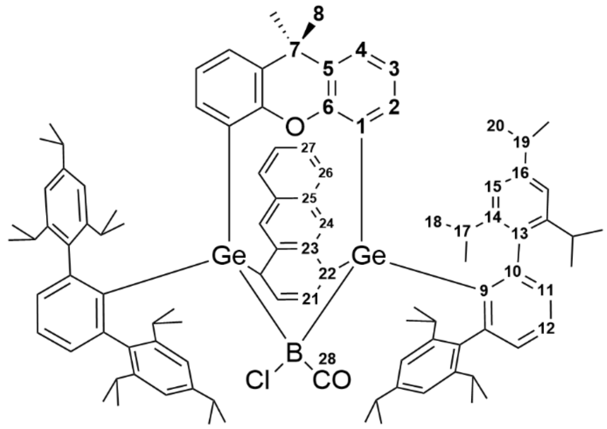


**Synthesis of compound 8**: A turquoise solution of boradigermaallyl **1** (100 mg, 73.4 µmol, 1.00 equiv.) in cyclohexane (2.00 ml) was frozen at −38 °C in a Schlenk tube with a volume of approximately 20 ml. To avoid premature contact with the next reactant, the frozen solution was covered with cyclohexane (1.00 ml) and the latter was then frozen at −38 °C as well. In the next step, a suspension of anthracene (13.1 mg, 73.4 µmol, 1.00 equiv.) in cyclohexane (5.00 ml) was added, frozen and the argon atmosphere of the Schlenk tube replaced by 1 bar carbon monoxide. The Schlenk tube was then brought to room temperature, and the entire turquoise reaction mixture was stirred for two hours at room temperature, during which a color change to pale green was observed. Volatile components were then removed under reduced pressure, the residue dissolved in *n*-pentane (10.0 ml) and the volume of the resulting green solution reduced under reduced pressure until crystals were formed. After crystallization overnight at room temperature, colorless crystals of the product **8** were obtained, which were suitable for X-ray diffraction. (76.1 mg, 48.5 µmol, 66 %). The product **8** continues to react at room temperature over the course of a few hours and should be stored at −38 °C.

**$^{1}$H-NMR** (700.21 MHz, $C_6D_6$, 283 K): $\delta$ [ppm] = 0.23 (d, 6H, $^{3}J_{HH}$ = 6.7 Hz, H-18), 0.72 (s, 3H, H-8), 0.79 (s, 3H, H-8), 0.91 (d, 6H, $^{3}J_{HH}$ = 6.6 Hz, H-18), 1.05 (d, 6H, $^{3}J_{HH}$ = 6.7 Hz, H-18), 1.15 (d, 6H, $^{3}J_{HH}$ = 6.7 Hz, H-18), 1.27 – 1.30 (m, 18H, H-20), 1.46 (d, 6H, $^{3}J_{HH}$ = 6.9 Hz, H-18), 1.47 – 1.50 (m, 12H, H-18 (6H) + H-20 (6H)), 1.65 (d, 6H, $^{3}J_{HH}$ = 6.6 Hz, H-18), 1.81 (d, 6H, $^{3}J_{HH}$ = 6.7 Hz, H-18), 2.79 (sept, 2H, $^{3}J_{HH}$ = 6.7 Hz, H-17), 2.86 (sept, 2H, $^{3}J_{HH}$ = 6.9 Hz, H-19), 3.07 (sept, 2H, $^{3}J_{HH}$ = 6.9 Hz, H-19), 3.14 – 3.18 (m, 2H, H-22), 3.32 (sept, 2H, $^{3}J_{HH}$ = 6.6 Hz, H-17), 3.58 (sept, 2H, $^{3}J_{HH}$ = 6.7 Hz, H-17), 3.80 (sept, 2H, $^{3}J_{HH}$ = 6.7 Hz, H-17), 4.93 – 4.97 (m, 2H, H-21), 5.91 (s, 2H, H-24), 6.55 (dd, 2H, $^{3}J_{HH}$ = 7.7 Hz, $^{4}J_{HH}$ = 1.0 Hz, H-4), 6.70 (t, 2H, $^{3}J_{HH}$ = 7.4 Hz, H-3), 6.83 (dd, 2H, $^{3}J_{HH}$ = 7.2 Hz, $^{4}J_{HH}$ = 1.0 Hz, H-2), 6.86 – 6.89 (m, 2H, H-26), 6.90 – 6.93 (m, 2H, H-27), 7.04 – 7.07 (m, 4H, H-12 + H-15 (2H)), 7.11 (dd, 2H, $^{3}J_{HH}$ = 7.7 Hz, $^{4}J_{HH}$ = 1.4 Hz, H-11), 7.14 (d, 2H, $^{4}J_{HH}$ = 1.5 Hz, H-15), 7.25 (d, 2H, $^{4}J_{HH}$ = 1.4 Hz, H-15), 7.32 (dd, 2H, $^{3}J_{HH}$ = 7.7 Hz, $^{4}J_{HH}$ = 1.4 Hz, H-11), 7.40 (d, 2H, $^{4}J_{HH}$ = 1.5 Hz, H-15). **$^{13}$C{$^{1}$H}-NMR** (176.07 MHz, $C_6D_6$, 283 K): $\delta$ [ppm] = 21.4 (C-8), 22.2 (C-18), 23.5 (C-20), 23.6 (C-18), 24.2 (C-18), 24.2 (C-20), 24.4 (C-18), 24.8 (C-20), 25.2 (C-20), 25.7 (C-18), 26.5 (C-18), 27.4 (C-18), 27.7 (C-18), 30.9 (C-17), 30.9 (C-17), 32.0 (C-17), 32.2 (C-17), 32.3 (C-8), 34.2 (C-19), 35.0 (C-19), 35.5 (C-7), 43.0 (C-22), 120.8 (C-15), 121.2 (C-15), 121.8 (C-15), 122.6 (C-15), 123.4

(C-27), 123.7 (C-3), 124.5 (C-24), 124.9 (C-4), 125.6 (C-21), 126.1 (C-12), 127.4 (C-26), 130.5 (C-25), 131.0 (C-5), 131.1 (C-1), 133.6 (C-2), 133.6 (C-11), 134.0 (C-11), 136.9 (C-23), 140.0 (C-13), 140.9 (C-13), 141.9 (C-9), 146.3 (C-14), 146.9 (C-14), 148.3 (C-16), 148.4 (C-10), 148.5 (C-14), 148.7 (C-14), 149.5 (C-16), 155.9 (C-6), 173.0 (C-28). **$^{11}B\{^{1}H\}$-NMR** (192.55 MHz, $C_6D_6$): $\delta$ [ppm] = −21.4 (s, br). **IR** (ATR, $cm^{-1}$): 2115 ($\nu_{C-O}$). **Elemental analysis calcd (%)** for $C_{102}H_{120}BClGe_2O_2$: C 78.06, H 7.71; found: C 78.88, H 7.74.

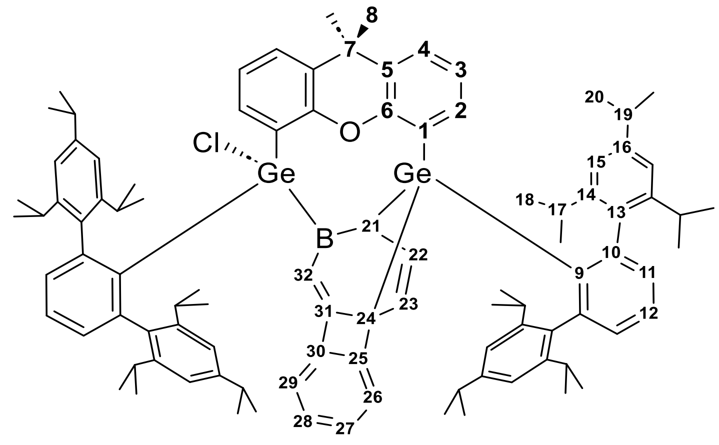


**Synthesis of compound 9**: Boradigermaallyl **1** (80.0 mg, 58.7 µmol, 1.00 equiv.) and biphenylene (8.93 µl, 58.7 µmol, 1.00 equiv.) were mixed together and *n*-pentane (3.50 ml) was added at room temperature. The turquoise reaction mixture was stirred for 2 weeks and a slow color change to green and finally orange was observed. After removing the solvent under reduced pressure, the residue was extracted with *n*-pentane. Small, suspended solids were filtered off and the yellow filtrate was concentrated under reduced pressure. Crystallization in *n*-pentane for three days at room temperature resulted in yellow crystals of **9**, suitable for X-ray structure analysis (64.8 mg, 38.0 µmol, 65 %.).

**$^{1}$H-NMR** (700.21 MHz, tol-d$_8$, 253 K): $\delta$ [ppm] = 0.40 (d, 3H, $^{3}J_{HH}$ = 6.6 Hz, H-18), 0.46 (d, 3H, $^{3}J_{HH}$ = 6.6 Hz, H-18), 0.72 (d, 3H, $^{3}J_{HH}$ = 6.9 Hz, H-20), 0.79 (d, 3H, $^{3}J_{HH}$ = 6.6 Hz, H-18), 0.82 (d, 3H, $^{3}J_{HH}$ = 6.8 Hz, H-18), 0.94 – 0.98 (m, 9H, H-18 (6H) + H-20 (3H)), 1.05 (d, 3H, $^{3}J_{HH}$ = 6.8 Hz, H-18), 1.08 (d, 3H, $^{3}J_{HH}$ = 6.8 Hz, H-18), 1.18 (d, 3H, $^{3}J_{HH}$ = 6.8 Hz, H-18), 1.24 (d, 3H, $^{3}J_{HH}$ = 6.8 Hz, H-20), 1.27 – 1.29 (m, 9H, H-8 (6H), H-18 (3H)), 1.31 (d, 3H, $^{3}J_{HH}$ = 6.5 Hz, H-18), 1.33 (d, 3H, $^{3}J_{HH}$ = 6.9 Hz, H-18), 1.36 (d, 3H, $^{3}J_{HH}$ = 7.0 Hz, H-20), 1.39 (d, 3H, $^{3}J_{HH}$ = 7.0 Hz, H-20), 1.40 (d, 3H, $^{3}J_{HH}$ = 7.0 Hz, H-20), 1.48 (d, 3H, $^{3}J_{HH}$ = 6.9 Hz, H-20), 1.50 – 1.53 (m, 6H, H-18 (6H)), 1.57 (d, 3H, $^{3}J_{HH}$ = 6.9 Hz, H-20), 1.71 (d, 3H, $^{3}J_{HH}$ = 6.9 Hz, H-18), 1.76 (d, 3H, $^{3}J_{HH}$ = 6.7 Hz, H-18), 2.08 – 2.12 (m, 1H, H-17, overlapped by solvent signal), 2.47 (sept., 1H, $^{3}J_{HH}$ = 6.8 Hz, H-19), 2.84 (sept., 1H, $^{3}J_{HH}$ = 6.9 Hz, H-19), 2.90 – 2.96 (m, 2H, H-17 (1H) + H-19 (1H)), 2.99 (sept., 1H, $^{3}J_{HH}$ = 6.7 Hz, H-17), 3.12 (sept., 1H, $^{3}J_{HH}$ = 6.8 Hz, H-19), 3.15 – 3.24 (m, 2H, H-17 (2), 3.44 (sept., 1H, $^{3}J_{HH}$ = 6.7 Hz, H-17), 3.50 (sept., 1H, $^{3}J_{HH}$ = 6.7 Hz, H-17), 3.60 (sept., 1H, $^{3}J_{HH}$ = 6.6 Hz, H-17), 3.75 (d, br, 1H, $^{3}J_{HH}$ = 5.6 Hz, H-21), 4.64 (s, 1H, H-32), 5.49 (d, 1H, $^{3}J_{HH}$ = 7.5 Hz, H-26), 6.34 – 6.37 (m, 2H, H-23 (1H) + H-29 (1H)), 6.43 – 6.46 (m, 1H, H-22), 6.59 – 6.65 (m, 2H, H-3 (1H) + H-28 (1H)), 6.73 – 6.76 (m, 1H, H-3), 6.80 – 6.84 (m, 3H, H-4 (1H) + H-15 (1H) + H-27 (1H)), 6.85 – 6.88 (m, 1H, H-11), 6.90 – 6.92 (m, 2H, H-11 (1H), H-12 (1H)), 6.92 – 6.95 (m, 2H, H-2 (1H), H-15 (1H)), 6.95 – 6.99 (m, 2H, H-4 (1H), H-15 (1H), overlapped by solvent signal), 7.01 – 7.04 (m, 1H, H-11, overlapped by solvent signal), 7.04 – 7.06 (m, 1H, H-2), 7.08 (t, 1H, $^{3}J_{HH}$ = 7.5 Hz, H-12), 7.14 – 7.16 (m, 1H, H-11), 7.19 – 7.21 (m, 3H, H-15 (3H)), 7.34 (s, br, 1H, H-15), 7.38 (s, br, 1H, H-15). **$^{13}$C-NMR** (176.07 MHz, tol-d$_8$, 253 K): $\delta$ [ppm] = 22.0 (C-18), 22.2 (C-20), 22.4 (C-20), 22.5 (C-18), 22.6 (C-18), 23.0 (C-18), 23.6 (C-20), 23.9 (C-18), 24.1 (C-18), 24.2 (C-20), 24.8 (C-20), 25.0 (C-18), 25.1 (C-18), 25.2

(C-18), 25.3 (C-20), 25.3 (C-20), 25.4 (C-18), 25.6 (C-18), 25.9 (C-18), 25.9 (C-18), 26.2 (C-18), 26.6 (C-20), 26.9 (C-18), 27.1 (C-18), 30.5 (C-17), 30.8 (C-17), 30.9 (C-17), 31.7 (C-17), 31.8 (C-17), 31.9 (2 x C-17), 32.1 (C-17), 33.1 (C-8), 33.7 (C-19), 33.8 (C-19), 33.9 (C-8), 34.1 (C-7), 34.2 (C-19), 35.0 (C-19), 43.2 (C-21), 69.0 (C-24), 118.1 (C-32), 119.0 (C-29), 119.4 (C-15), 120.0 (C-15), 121.2 (2 x C-15), 121.7 (C-15), 122.6 (C-15), 122.8 (C-3), 122.9 (C-3), 123.0 (C-15), 123.4 (C-15), 124.1 (C-26), 126.7 (C-28), 126.8 (C-12), 127.1 (C-1), 128.0 (C-12, overlapped by solvent signal), 128.0 (C-4, overlapped by solvent signal), 128.2 (C-5), 128.4 (C-4), 129.3 (C-5), 130.2 (C-1), 130.4 (C-27), 130.9 (C-11), 131.9 (C-22), 132.5 (C-11), 133.1 (C-23), 133.7 (C-2 + C-11), 133.9 (C-2), 134.1 (C-11), 137.0 (C-13), 139.2 (C-13), 139.6 (C-13), 139.8 (C-9), 141.5 (C-9), 142.0 (C-13), 142.2 (C-30), 145.3 (C-14), 145.6 (C-14), 145.8 (C-14), 145.9 (C-14), 146.2 (C-10), 146.7 (C-10), 146.9 (C-16), 146.9 (C-14), 147.0 (C-14), 147.4 (C-14), 147.6 (C-10), 148.5 (C-16), 148.5 (C-25), 148.6 (C-14), 149.1 (C-16), 149.5 (C-14), 149.6 (C-10), 151.7 (C-6), 152.1 (C-6), 168.2 (C-31). **$^{11}$B-MAS-NMR** (96.29 MHz): $\delta$ [ppm] = 71.0. **UV/Vis** (*n*-pentane, c = 0.048 mmol·L$^{-1}$): λ [nm] (ε [L·mol$^{-1}$cm$^{-1}$): 345 (11800), 320 (16400). **Elemental analysis cacld (%) for** $C_{99}H_{118}BClGe_2O$: C 78.46, H 7.85; found: C 79.09, H 7.73.

# Crystal structure determination

**Table S1:** Data of crystal structure determination.

| | **2** | **3** | **4** | **5** |
|---|---|---|---|---|
| empirical formula | $C_{93}H_{122}BClGe_2O$ ($Et_2O$) | $C_{95}H_{118}BClGe_2O$ | $C_{95}H_{116}BClGe_2O$ 2x ($Et_2O$) | $C_{95}H_{116}BClGe_2O$ |
| $M$ [g/mol] | 1521.51 | 1467.38 | 1613.61 | 1465.37 |
| $T$ [K] | 100(2) | 100(2) | 130(2) | 100(2) |
| $\lambda$ [Å] | 0.71073 | 0.71073 | 0.71073 | 0.71073 |
| crystal system | triclinic | triclinic | triclinic | triclinic |
| space group | $P\bar{1}$ | $P\bar{1}$ | $P\bar{1}$ | $P\bar{1}$ |
| Z | 2 | 2 | 2 | 2 |
| $a$ [Å] | 13.3309(4) | 13.7734(4) | 14.4877(4) | 13.5962(4) |
| $b$ [Å] | 17.1204(5) | 16.6004(5) | 18.0318(4) | 16.6710(5) |
| $c$ [Å] | 20.0227(6) | 20.0960(7) | 18.9385(5) | 20.1773(6) |
| $\alpha$ [°] | 78.851(2) | 83.116(2) | 77.2310(10) | 81.8234(16) |
| $\beta$ [°] | 72.034(2) | 85.997(2) | 89.5030(10) | 87.2510(15) |
| $\gamma$ [°] | 84.771(2) | 84.814(2) | 73.8950(10) | 84.0915(14) |
| $V$ [Å$^3$] | 4262.5(2) | 4534.9(2) | 4628.4(2) | 4500.3(2) |
| $D_c$ [g/cm$^3$] | 1.185 | 1.075 | 1.158 | 1.081 |
| μ [mm$^{-1}$] | 0.784 | 0.734 | 0.726 | 0.740 |
| F(000) | 1632 | 1564 | 1728 | 1560 |
| crystal size [mm] | 0.293 x 0.235 x 0.200 | 0.26 x 0.25 x 0.22 | 0.436 x 0.429 x 0.392 | 0.31 x 0.28 x 0.26 |
| $\theta$ range [°] | 2.438 – 30.529 | 2.785 – 26.551 | 2.415 – 29.641 | 2.530 – 28.796 |
| limiting indices | $-19 \le h \le 19$<br>$-24 \le k \le 24$<br>$-28 \le l \le 28$ | $-16 \le h \le 17$<br>$-20 \le k \le 20$<br>$-25 \le l \le 25$ | $-20 \le h \le 20$<br>$-25 \le k \le 25$<br>$-26 \le l \le 26$ | $-18 \le h \le 18$<br>$-22 \le k \le 22$<br>$-27 \le l \le 26$ |
| reflections collected | 89239 | 66600 | 292533 | 127644 |
| independent reflections | 25701 | 18029 | 25905 | 23374 |
| $R_{int}$ | 0.0501 | 0.0619 | 0.0320 | 0.0537 |
| Completeness [%] | 98.6 | 95.3 | 99.2 | 99.5 |
| absorption correction | multi-scan | multi-scan | multi-scan | multi-scan |
| max., min. transmission | 0.7034, 0.7461 | 0.6050, 0.7454 | 0.9387, 1.0000 | 0.6851, 0.7458 |
| parameter/restraints | 956 / 0 | 976 / 399 | 1030 / 139 | 930 / 0 |
| $R_1$, $\omega R_2$ [$I > 2\sigma(I)$] | 0.0412, 0.0957 | 0.0638, 0.1498 | 0.0378, 0.0977 | 0.0377, 0.1042 |
| $R_1$, $\omega R_2$ (all data) | 0.0637, 0.1049 | 0.1038, 0.1678 | 0.0463, 0.1041 | 0.0536, 0.1114 |
| GooF on $F^2$ | 1.019 | 1.040 | 1.032 | 1.038 |
| peak / hole [e·Å$^{-3}$] | 1.317, −0.555 | 1.937, −1.008 | 1.481, −1.175 | 0.467, −0.389 |
| CCDC | 2418123 | 2418127 | 2418126 | 2418129 |

**Table S2:** Data of crystal structure determination.

| | **6** | **7** | **8** | **9** |
|---|---|---|---|---|
| empirical formula | $C_{96}H_{116}BClGe_2O_2$ $(Et_2O)$ | $C_{96}H_{116}BClGe_2O_2$ 2x (*n*-pentane) | $C_{102}H_{120}BClGe_2O$ 2.5x (*n*-pentane) | $C_{99}H_{118}BClGe_2O$ |
| *M* [g/mol] | 1568.50 | 1637.61 | 1749.78 | 1515.42 |
| *T* [K] | 120(2) | 120 (2) | 120 (2) | 100(2) |
| *λ* [Å] | 0.71073 | 0.71073 | 0.71073 | 0.71073 |
| crystal system | triclinic | monoclinic | monoclinic | triclinic |
| space group | $P\bar{1}$ | $P2_1/n$ | *P2/c* | $P\bar{1}$ |
| Z | 2 | 4 | 4 | 2 |
| *a* [Å] | 14.1976(3) | 19.5707(6) | 25.9105(14) | 15.9892(3) |
| *b* [Å] | 14.5282(3) | 18.9384(6) | 14.4062(8) | 19.2578(3) |
| *c* [Å] | 22.8599(4) | 26.3009(8) | 28.4018(16) | 20.5718(4) |
| *α* [°] | 87.8450(10) | 90 | 90 | 102.6310(10) |
| *β* [°] | 74.4580(10) | 107.021(2) | 109.755(3) | 112.2980(10) |
| *γ* [°] | 75.2490(10) | 90 | 90 | 106.5770(10) |
| *V* [$Å^3$] | 4390.70(15) | 9321.1(5) | 9977.6(10) | 5221.48(17) |
| $D_c$ [$g/cm^3$] | 1.186 | 1.167 | 1.165 | 0.964 |
| μ [$mm^{-1}$] | 0.764 | 0.722 | 0.678 | 0.639 |
| F(000) | 1672 | 3512 | 3756 | 1612 |
| crystal size [mm] | 0.30 x 0.28 x 0.27 | 0.31 x 0.27 x 0.26 | 0.30 x 0.28 x 0.26 | 0.27 x 0.26 x 0.25 |
| *θ* range [°] | 2.956 – 27.990 | 1.530 – 27.619 | 1.469 – 30.533 | 1.954 – 30.434 |
| limiting indices | −18 ≤ h ≤ 18<br>−19 ≤ k ≤ 18<br>−30 ≤ l ≤ 30 | −25 ≤ h ≤ 25<br>−24 ≤ k ≤ 24<br>−34 ≤ l ≤ 34 | −37 ≤ h ≤ 36<br>−20 ≤ k ≤ 20<br>−38 ≤ l ≤ 40 | −22 ≤ h ≤ 22<br>−27 ≤ k ≤ 27<br>−29 ≤ l ≤ 29 |
| reflections collected | 105316 | 195734 | 331399 | 281112 |
| independent reflections | 20982 | 21434 | 30530 | 31410 |
| $R_{int}$ | 0.0625 | 0.0397 | 0.0549 | 0.0335 |
| Completeness [%] | 99.1 | 98.9 | 99.9 | 99.2 |
| absorption correction | multi-scan | multi-scan | multi-scan | multi-scan |
| max., min. transmission | 0.6961, 0.7456 | 0.6666, 0.7456 | 0.6299, 0.7461 | 0.7133, 0.7461 |
| parameter/restraints | 992 / 0 | 1039 / 0 | 1363 / 960 | 984 / 75 |
| $R_1$, $\omega R_2$ [$I > 2\sigma(I)$] | 0.0555, 0.1163 | 0.0351, 0.0875 | 0.0578, 0.1256 | 0.0487, 0.1290 |
| $R_1$, $\omega R_2$ (all data) | 0.0895, 0.1299 | 0.0544, 0.0992 | 0.0738, 0.1315 | 0.0632, 0.1407 |
| GooF on $F^2$ | 1.015 | 1.027 | 1.151 | 1.047 |
| peak / hole [$e \cdot Å^{-3}$] | 2.525, −1.659 | 0.920, −0.480 | 1.062, −1.275 | 1.660, −2.192 |
| CCDC | 2418124 | 2418130 | 2418128 | 2418125 |

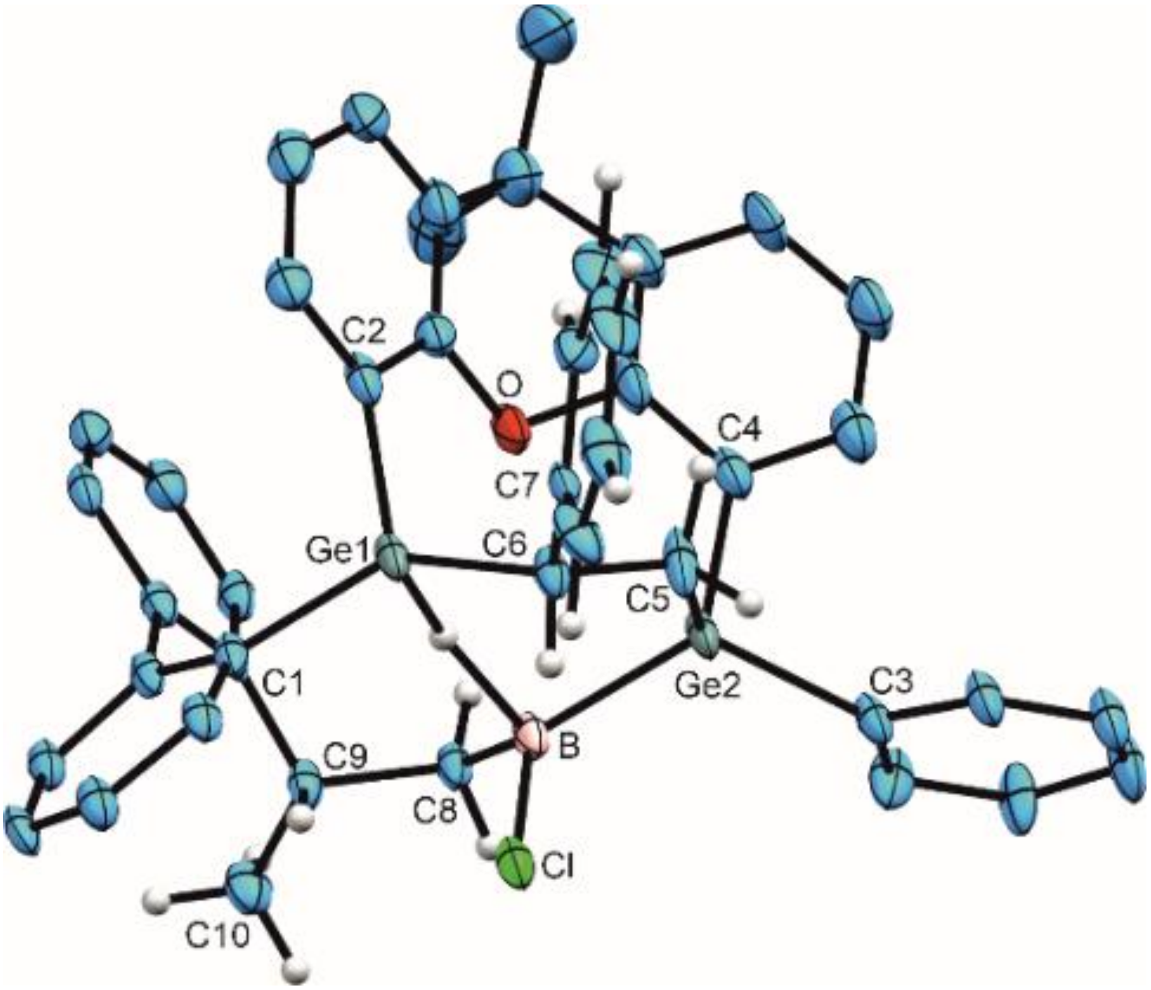


Figure S1. ORTEP of the molecular structure of **3**. Thermal ellipsoids are shown at 50 % probability level. Hydrogen atoms, $^{i}$Pr and Trip groups except the styrene H-atoms and the reacting $^{i}$Pr-group have been omitted. Selected interatomic distances [Å]: Ge1-C1 1.973(4), Ge1-C2 1.937(4), Ge1-C6 1.984(4), Ge2-C3 1.994(4), Ge2-C4 1.980(4), Ge2-C5 1.995(4), Ge2-B 2.081(4), C5-C6 1.562(5), B-C8 1.553(6), B-Cl 1.854(5).

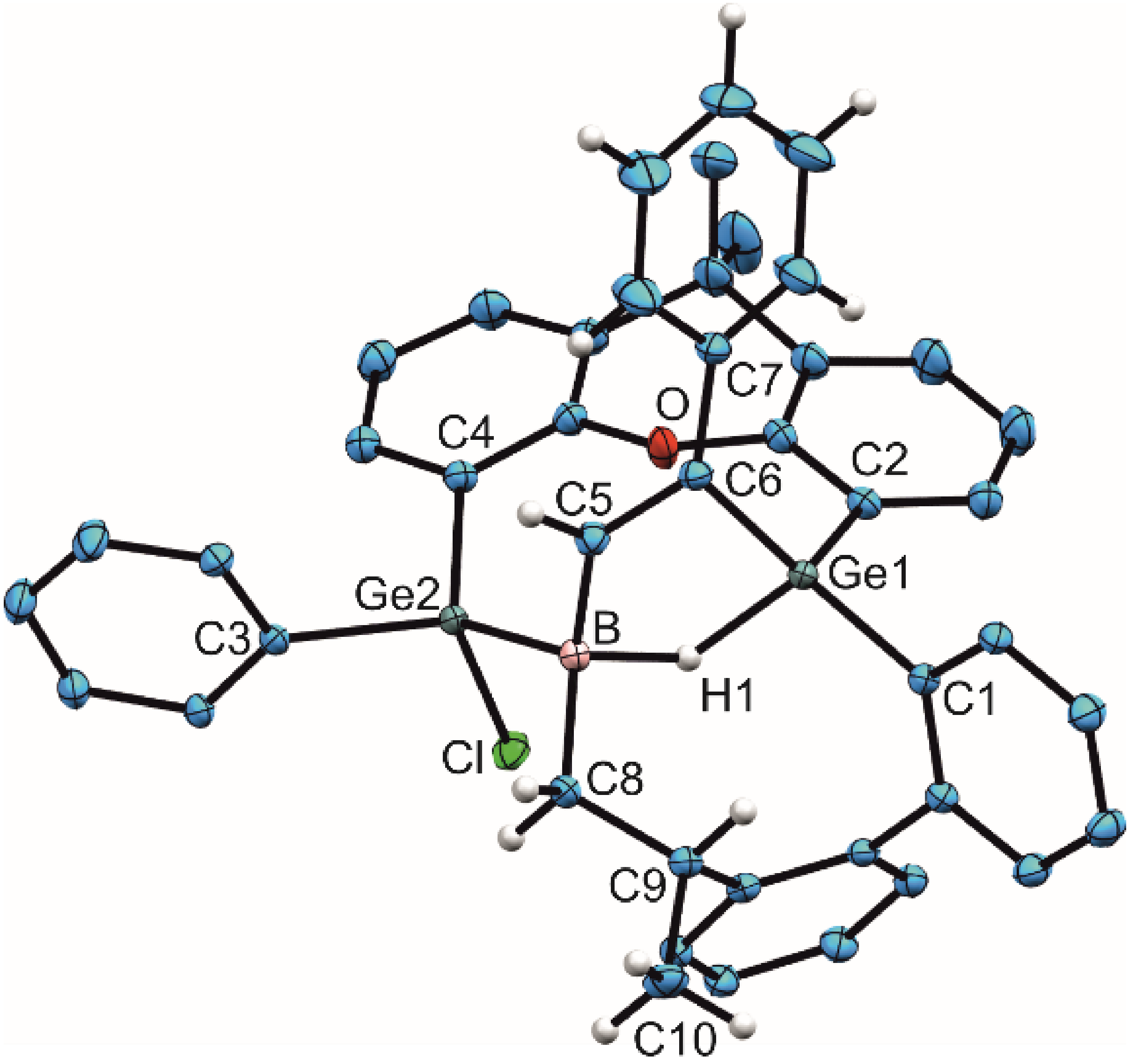


Figure S2. ORTEP of the molecular structure of **4**. Thermal ellipsoids are shown at 50 % probability level. Hydrogen atoms, $^{i}$Pr and Trip groups except the phenylalkyne H-atoms and the reacting $^{i}$Pr group have been omitted. Selected interatomic distances [Å]: Ge1-C1 1.9550(14), Ge1-C2 1.9331(15), Ge1 C6 1.9356(14), Ge2-C3 2.0077(14), Ge2-C4 1.9834(14), Ge2-Cl 2.2042(4), Ge2-B 2.1280(16), B-C5 1.576(2), B-C8 1.608(2), C5-C6 1.348(2), B-H 1.46(2), Ge1-H 1.64(2).

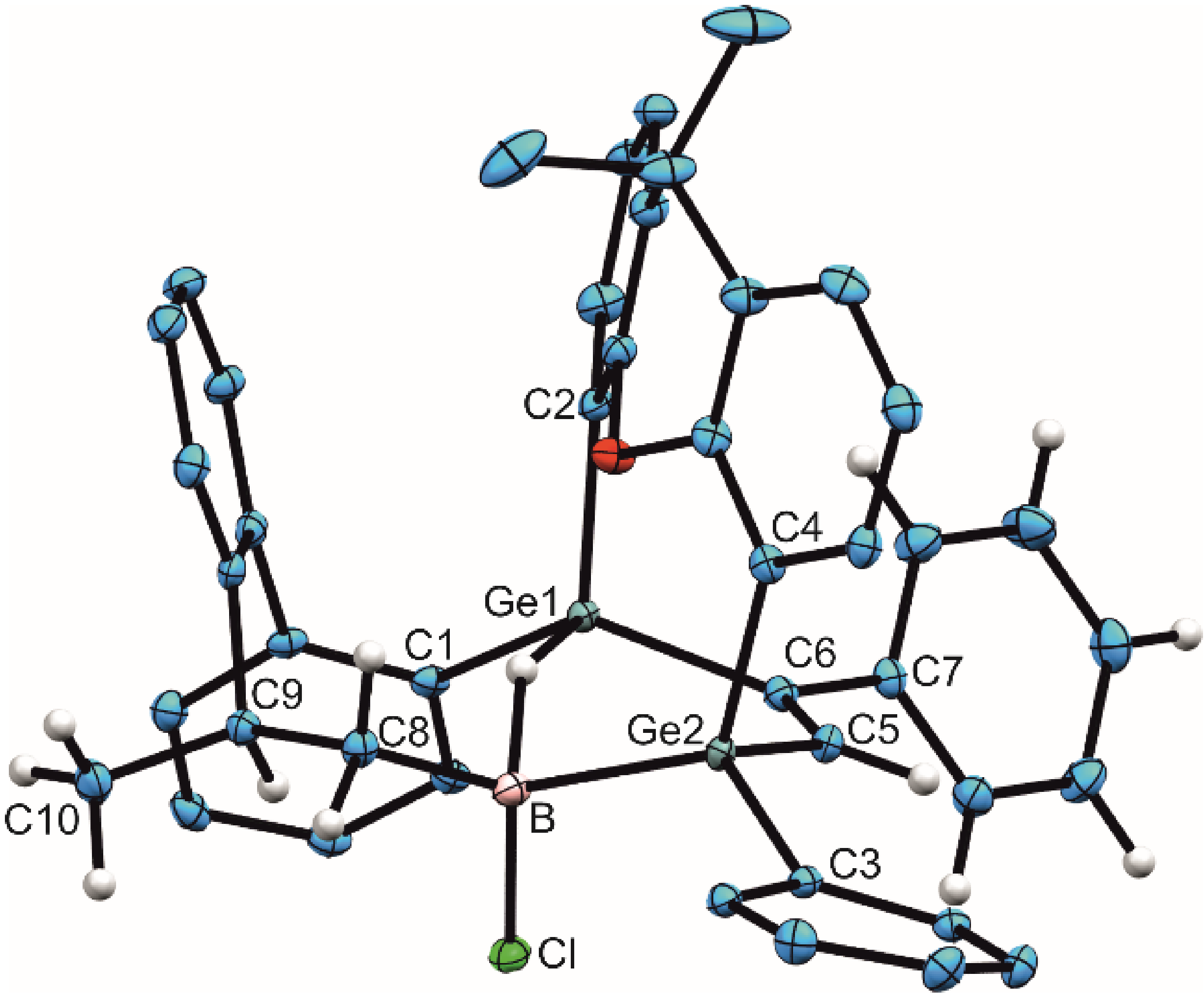


Figure S3. ORTEP of the molecular structure of **5**. Thermal ellipsoids are shown at 50 % probability level. Hydrogen atoms, $^{i}$Pr and Trip groups except the phenylalkyne H-atoms and the reacting $^{i}$Pr group have been omitted. Selected interatomic distances [Å]: Ge1-C1 1.9704(17), Ge1-C2 1.9579(17), Ge1-C6 1.9747(18), Ge2-C3 1.9931(17), Ge2-C4 1.9897(17), Ge2-C5 1.9820(18), Ge2-B 2.073(2), B-Cl 1.838(2), B-C8 1.571(3), B-H 1.56(2), Ge1-H 1.54(2), C5-C6 1.345(2).

# NMR spectroscopy

## NMR spectra of compound **2.**

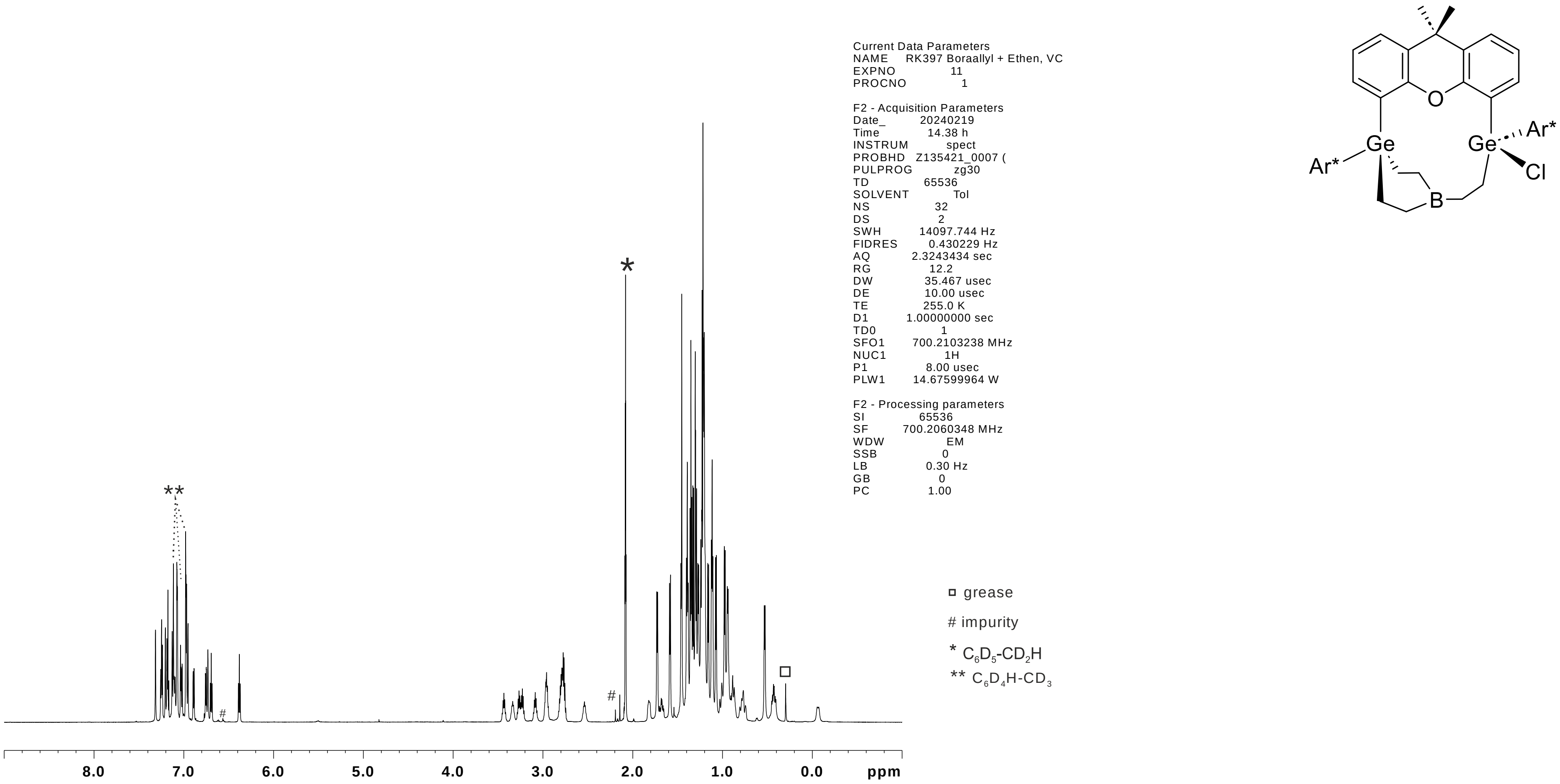


Figure S4. $^1$H NMR spectrum of compound **2.**

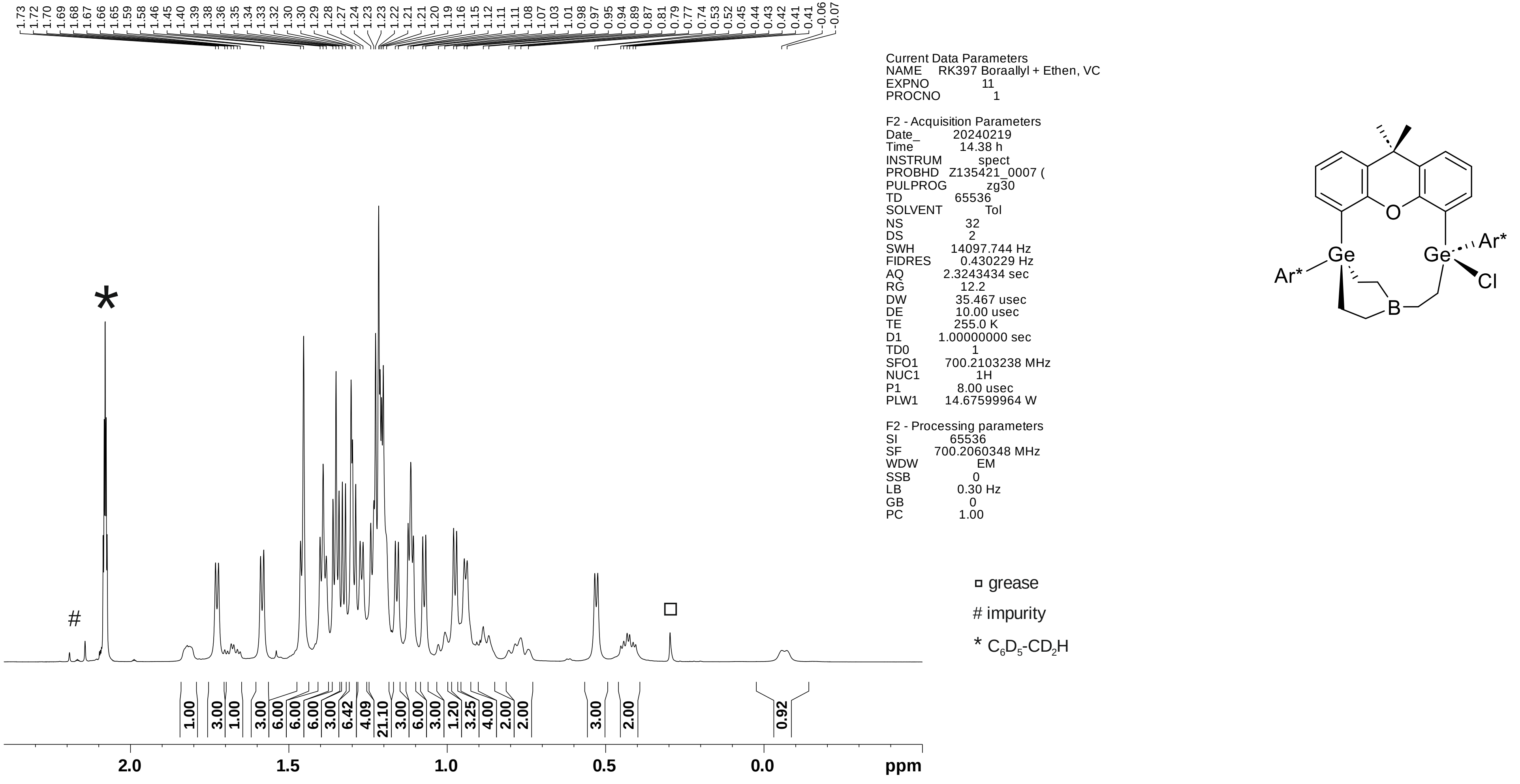


Figure S5. $^1H$ NMR spectrum of compound **2** (−0.5 – 2.4 ppm).

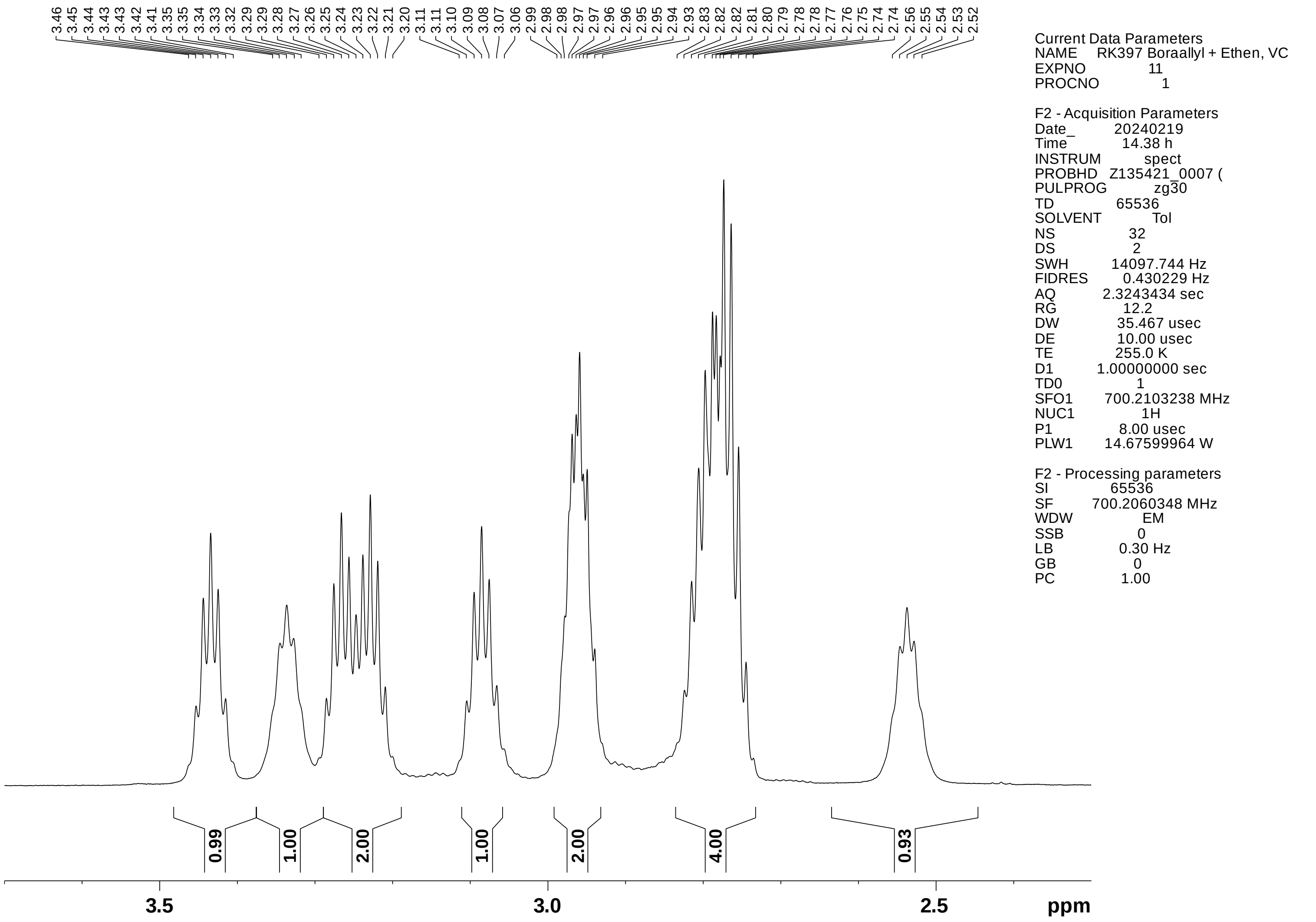


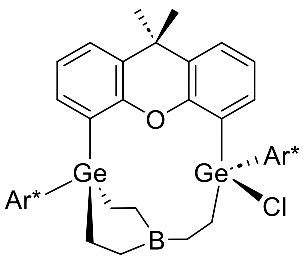


Figure S6. $^{1}$H NMR spectrum of compound **2** (2.3 – 3.7 ppm).

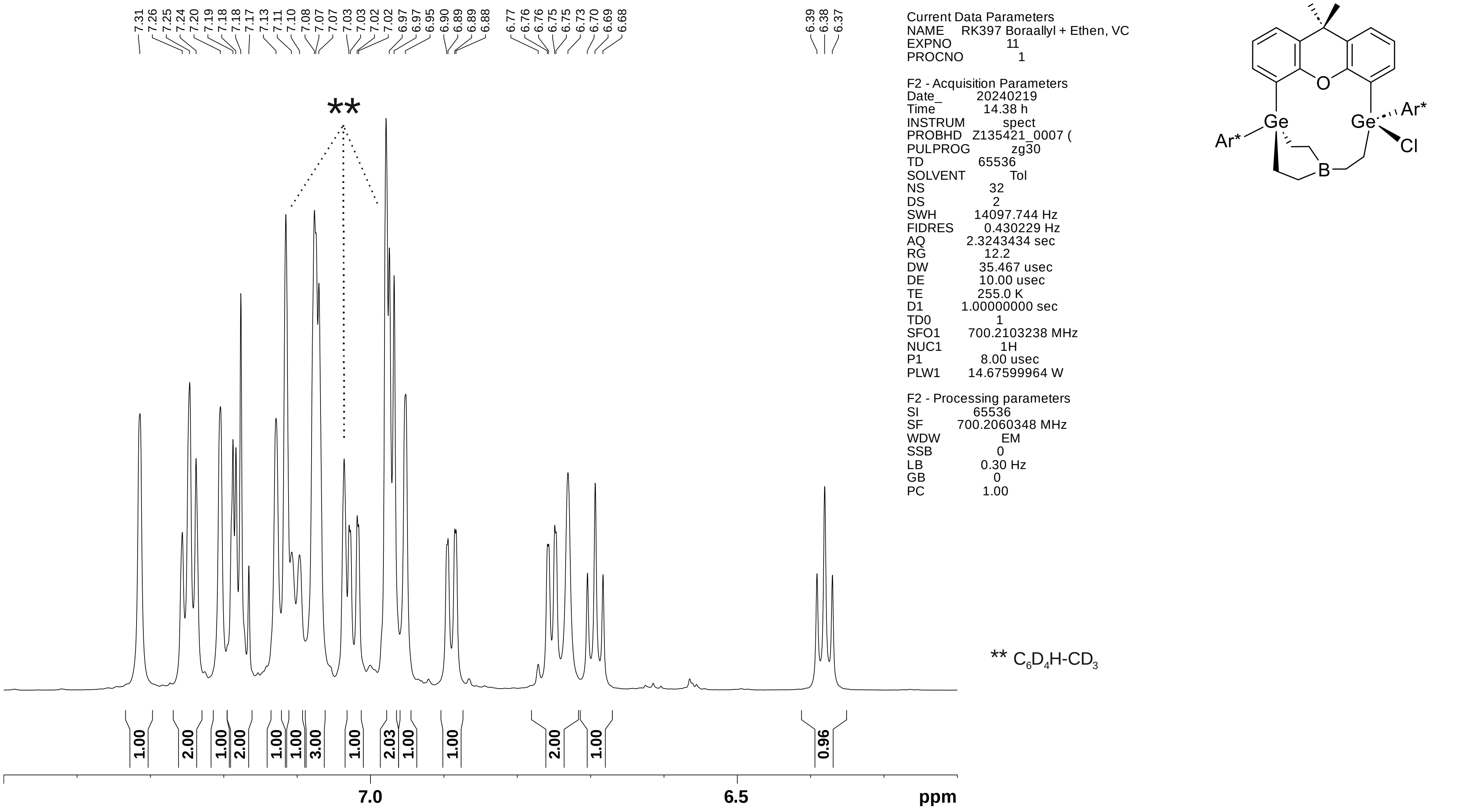


Figure S7. $^1$H NMR spectrum of compound **2** (6.2 – 7.5 ppm).

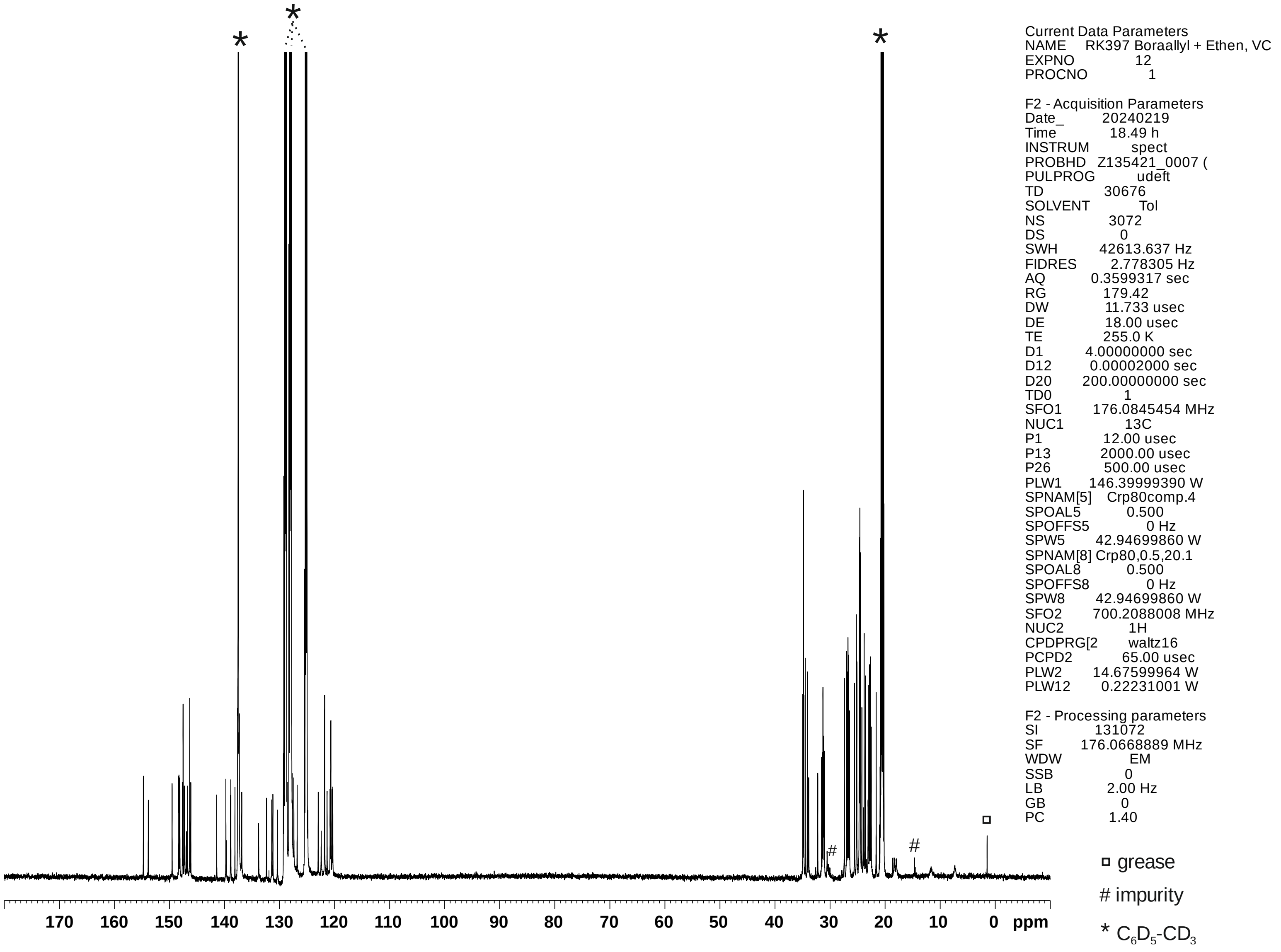


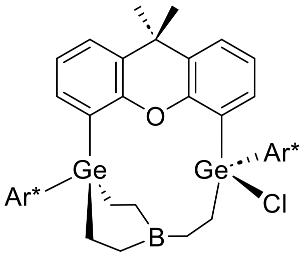


Figure S8. $^{13}C\{^1H\}$ NMR spectrum of compound **2**.

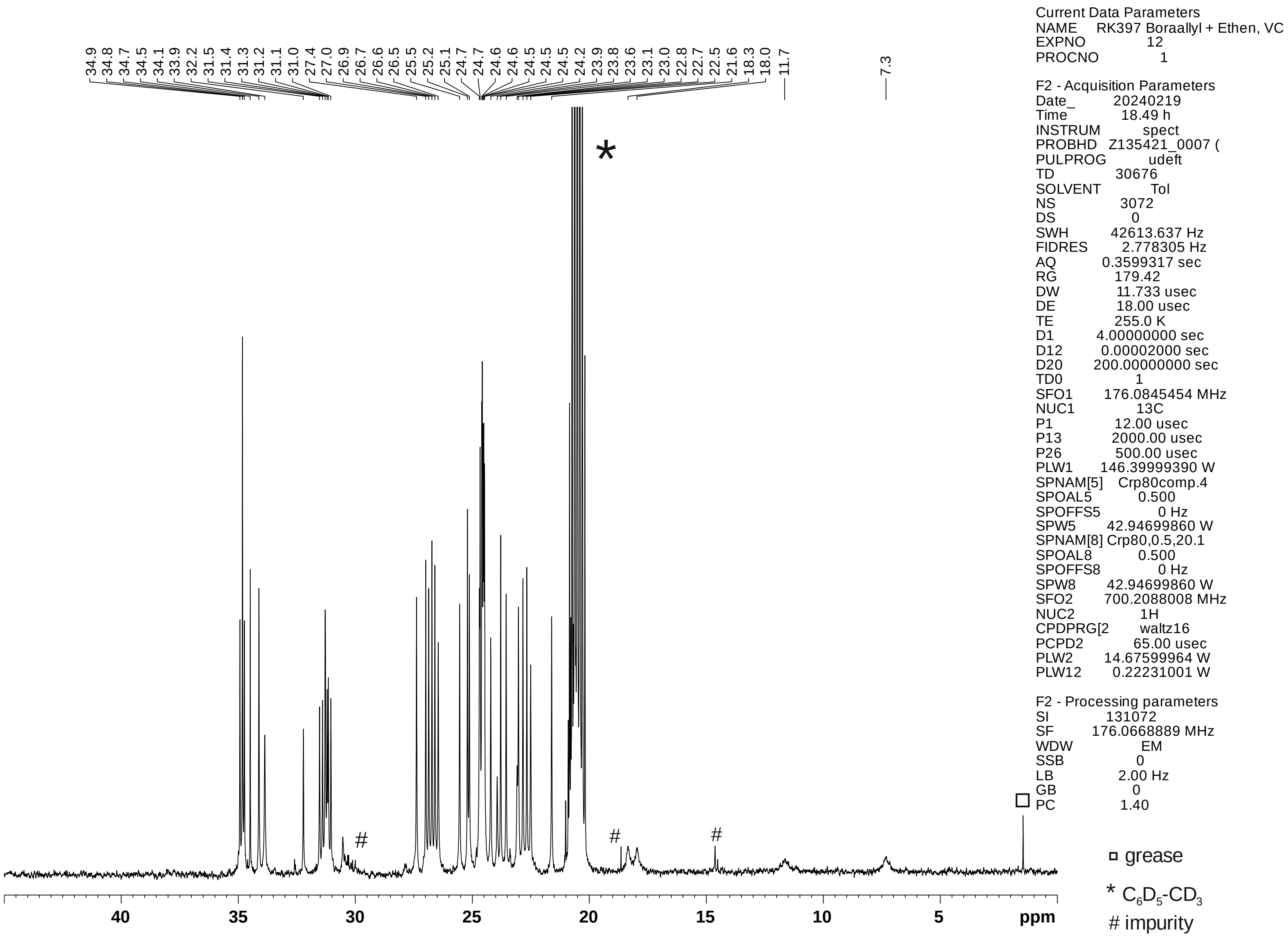


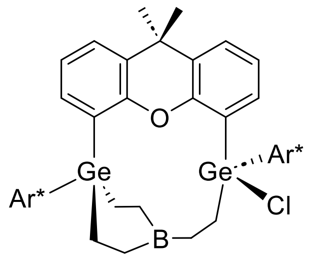


Figure S9. $^{13}C\{^{1}H\}$ NMR spectrum of compound **2** (0 – 45 ppm).

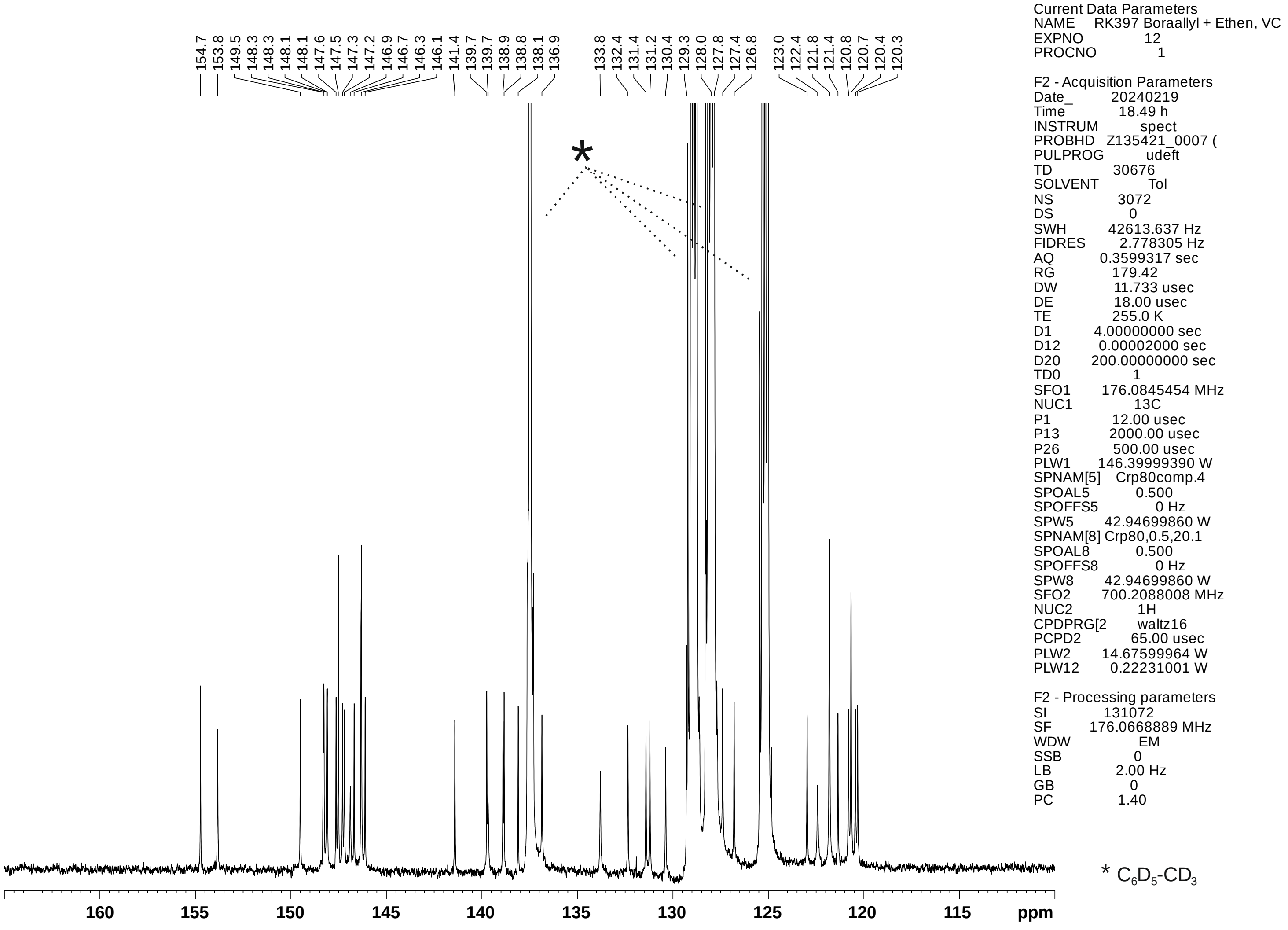


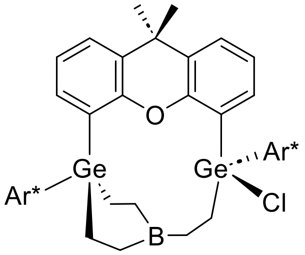


Figure S10. $^{13}C\{^{1}H\}$ NMR spectrum of compound **2** (110 – 165 ppm).

NMR spectra of compound **3**.

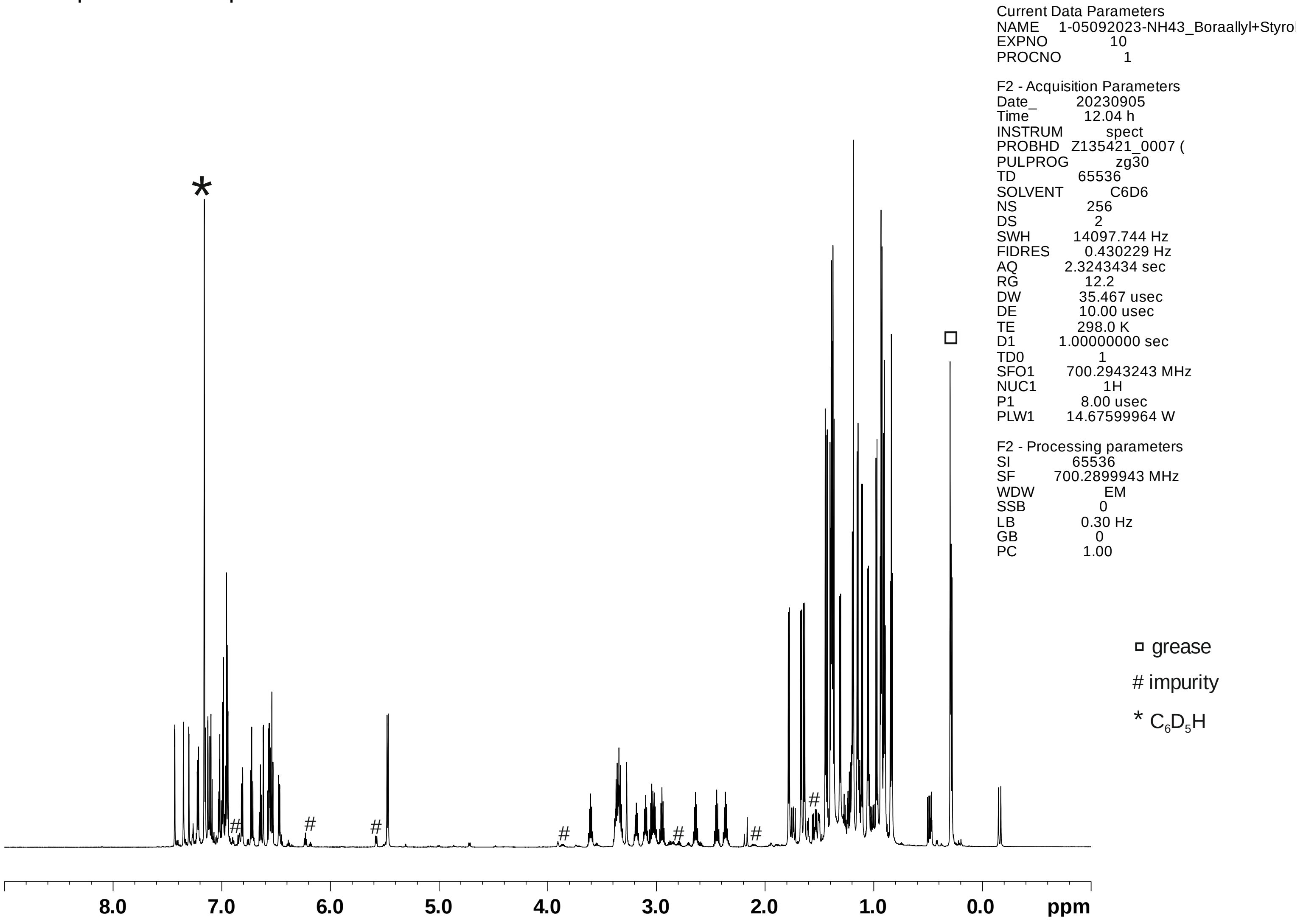


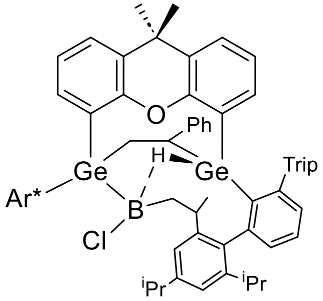


Figure S11. $^{1}$H NMR spectrum of compound **3**.

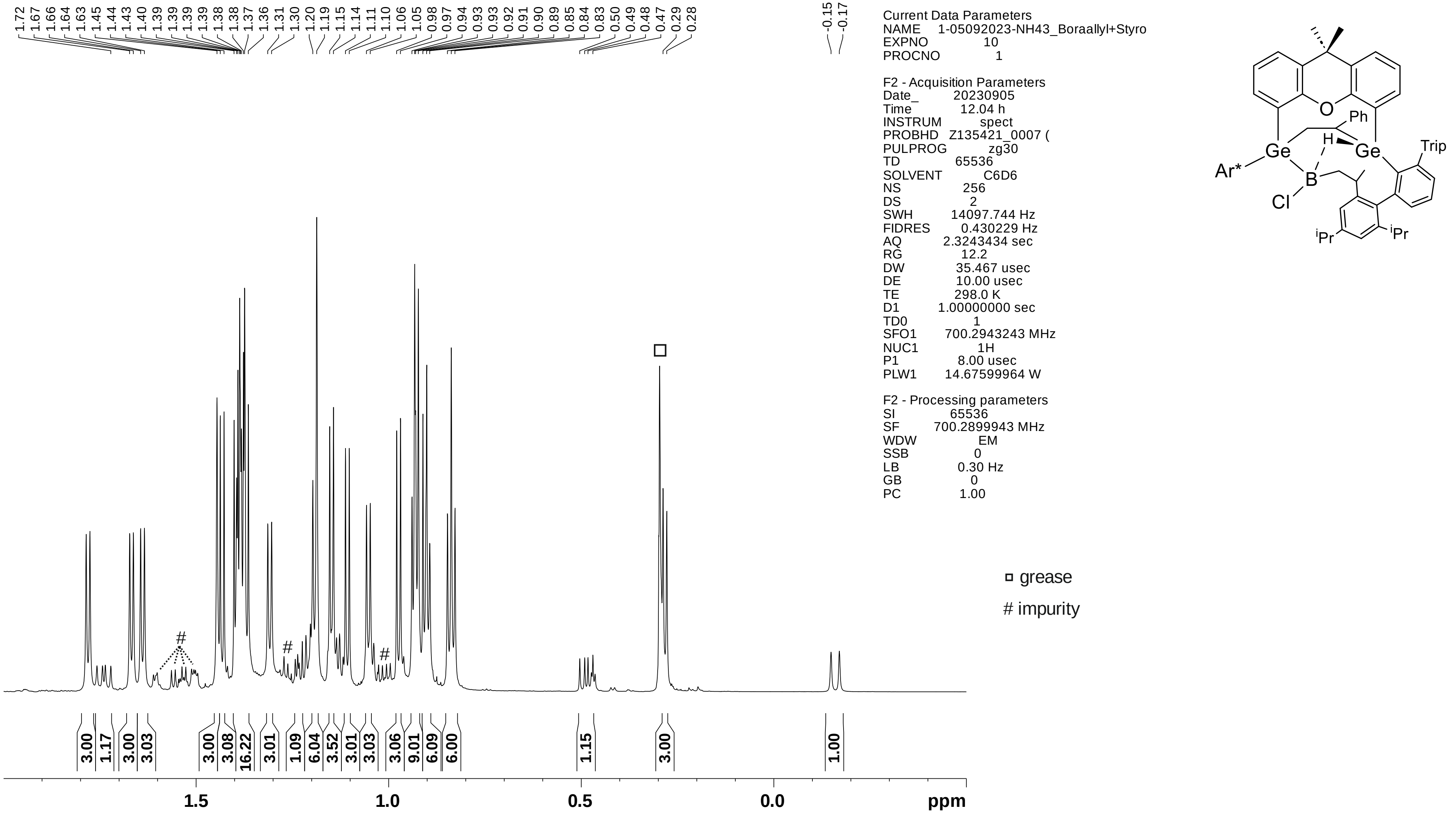


Figure S12. $^1H$ NMR spectrum of compound **3** (−0.5 – 2.0 ppm).

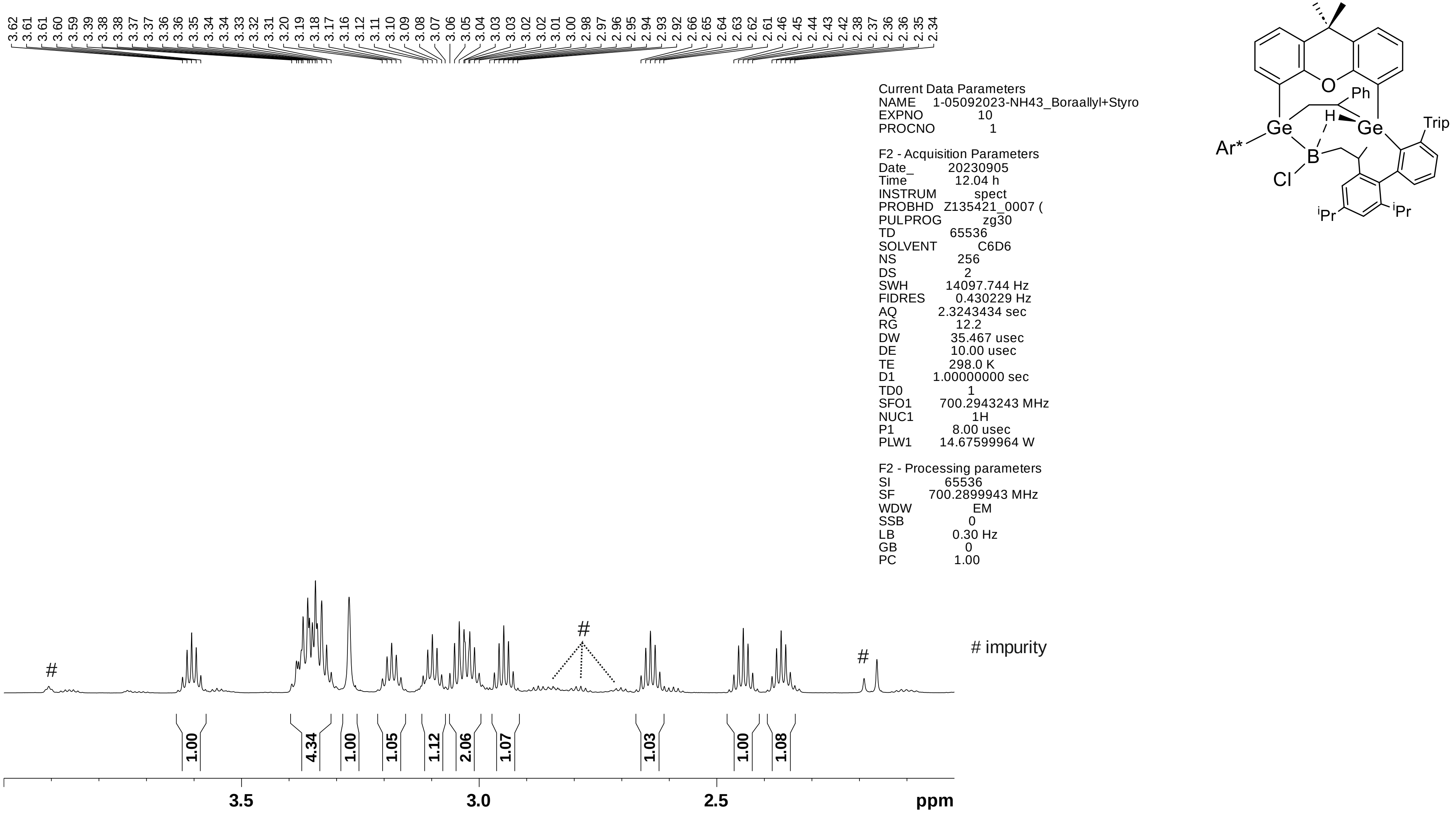


Figure S13. $^{1}$H NMR spectrum of compound **3** (2.0 – 4.0 ppm).

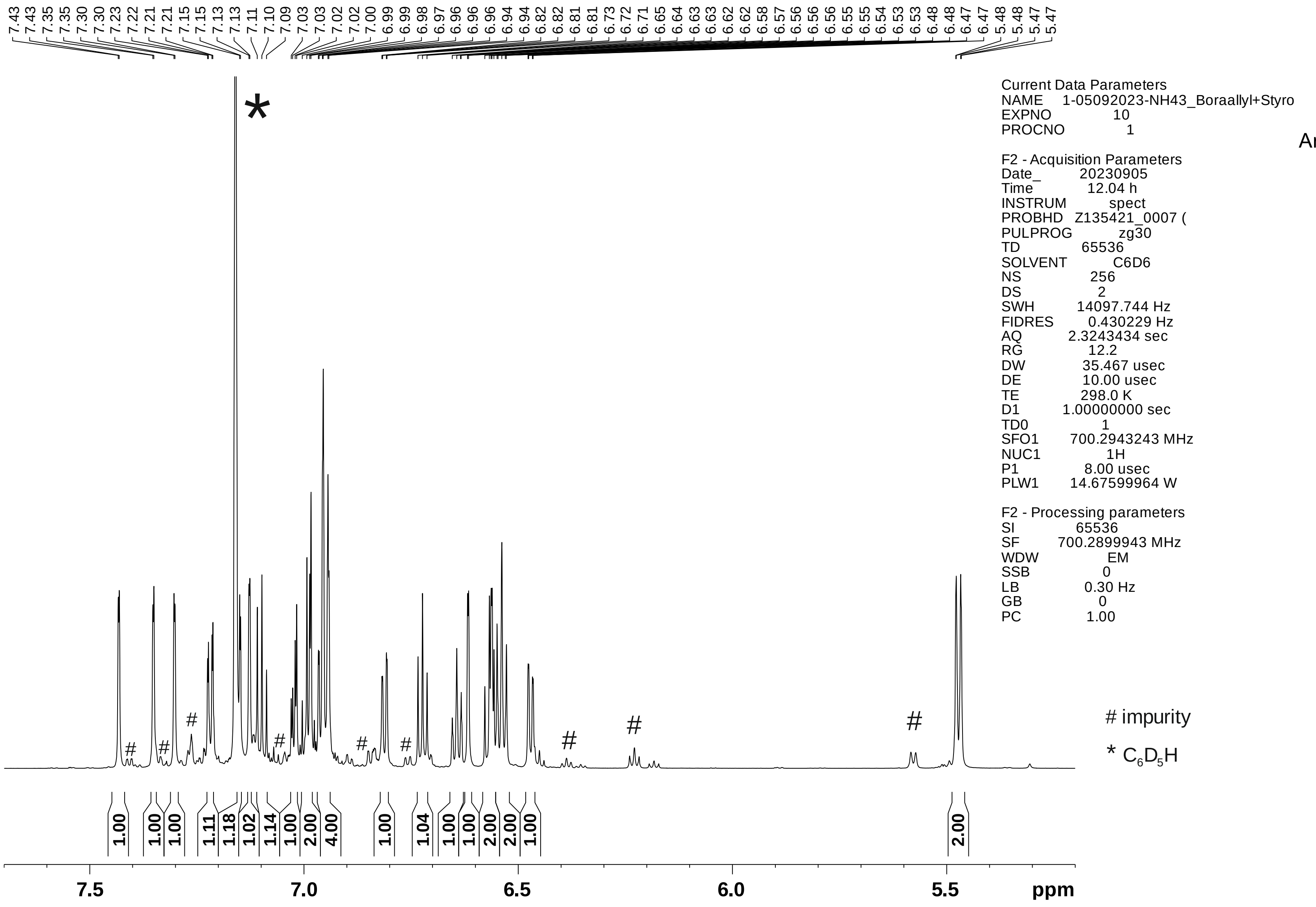

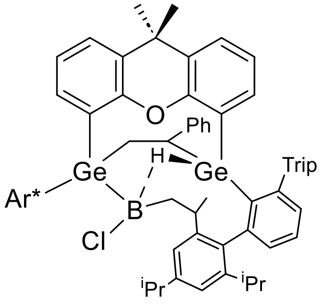


Figure S14. $^{1}$H NMR spectrum of compound **3** (5.2 – 7.7 ppm).

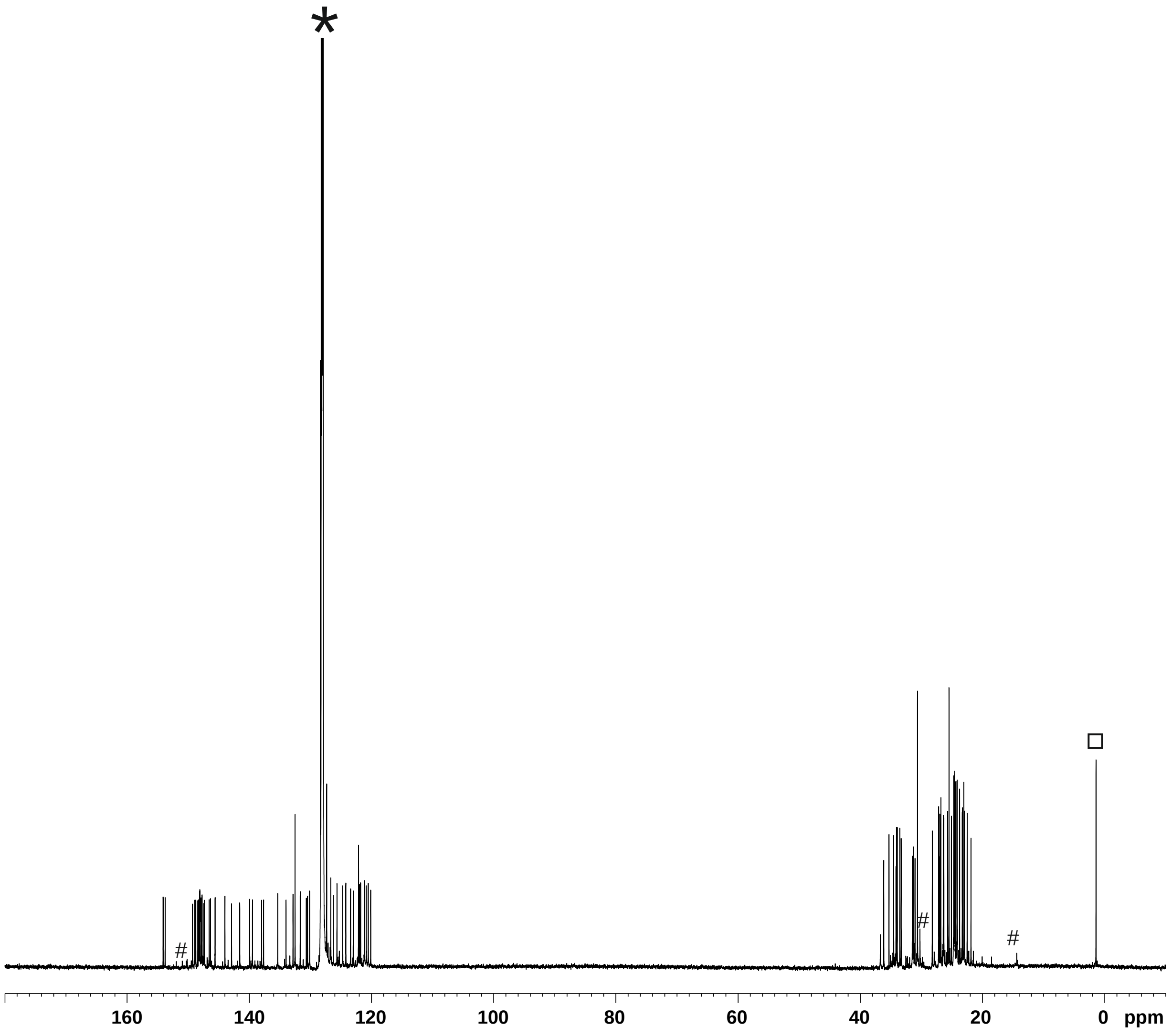


Current Data Parameters
NAME 1-05092023-NH43_Boraallyl+Styrol
EXPNO 11
PROCNO 1

F2 - Acquisition Parameters
Date_ 20230905
Time 17.36 h
INSTRUM spect
PROBHD Z135421_0007 (
PULPROG udeft
TD 30676
SOLVENT C6D6
NS 4096
DS 0
SWH 42613.637 Hz
FIDRES 2.778305 Hz
AQ 0.3599317 sec
RG 179.42
DW 11.733 usec
DE 18.00 usec
TE 298.0 K
D1 4.00000000 sec
D12 0.00002000 sec
D20 200.00000000 sec
TD0 1
SFO1 176.1056694 MHz
NUC1 13C
P1 12.00 usec
P13 2000.00 usec
P26 500.00 usec
PLW1 146.39999390 W
SPNAM[5] Crp80comp.4
SPOAL5 0.500
SPOFFS5 0 Hz
SPW5 42.94699860 W
SPNAM[8] Crp80,0.5,20.1
SPOAL8 0.500
SPOFFS8 0 Hz
SPW8 42.94699860 W
SFO2 700.2928012 MHz
NUC2 1H
CPDPRG[2 waltz16
PCPD2 65.00 usec
PLW2 14.67599964 W
PLW12 0.22231001 W

F2 - Processing parameters
SI 131072
SF 176.0879952 MHz
WDW EM
SSB 0
LB 2.00 Hz
GB 0
PC 1.40

□ grease
# impurity
* $C_6D_6$

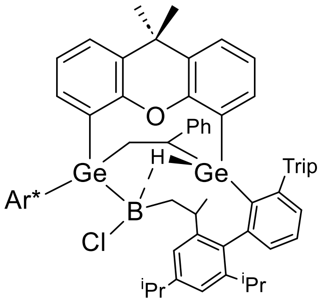


Figure S15. $^{13}C\{^1H\}$ NMR spectrum of compound **3**.

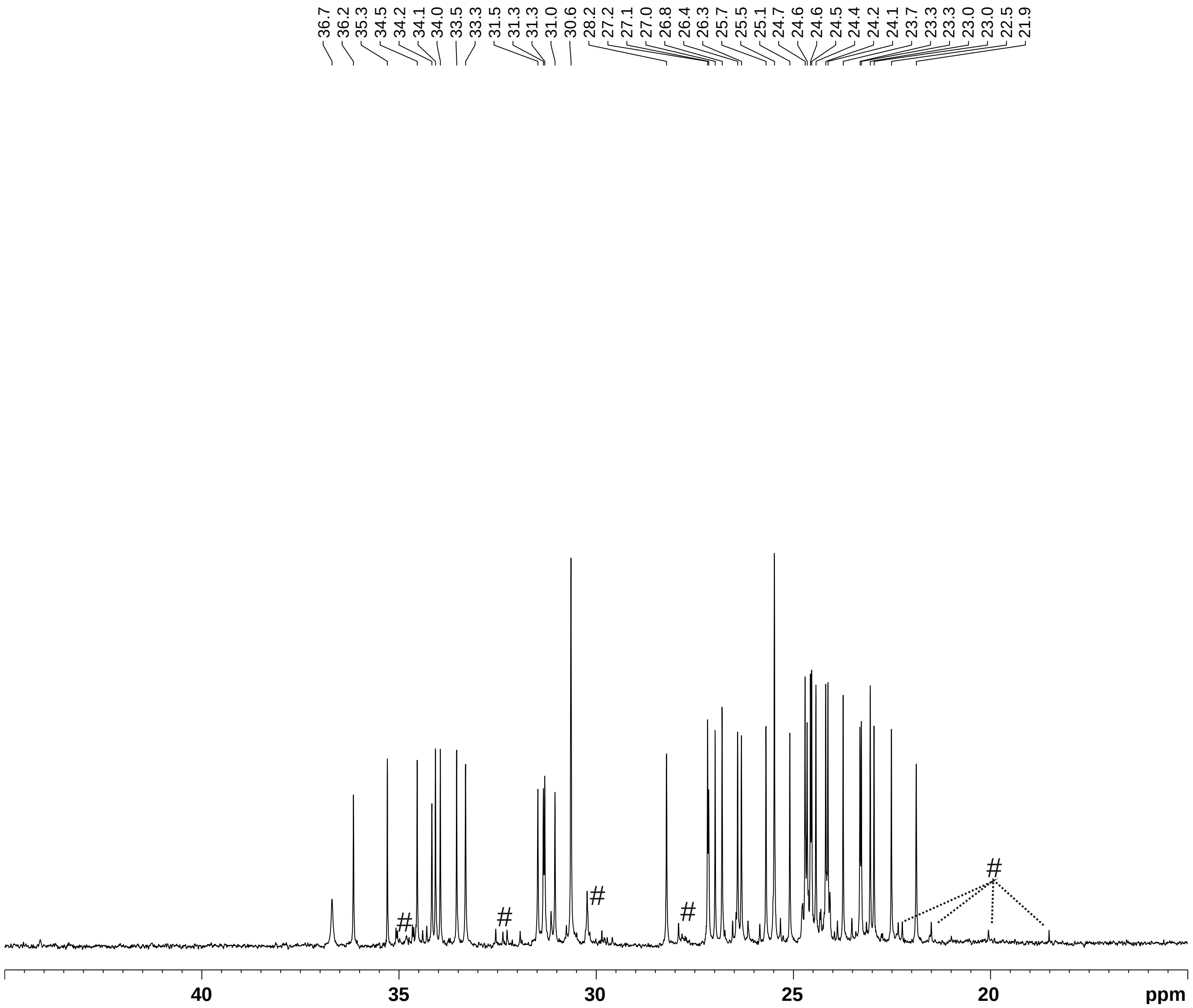

Current Data Parameters
NAME 1-05092023-NH43_Boraallyl+Styrol
EXPNO 11
PROCNO 1

F2 - Acquisition Parameters
Date_ 20230905
Time 17.36 h
INSTRUM spect
PROBHD Z135421_0007 (
PULPROG udeft
TD 30676
SOLVENT C6D6
NS 4096
DS 0
SWH 42613.637 Hz
FIDRES 2.778305 Hz
AQ 0.3599317 sec
RG 179.42
DW 11.733 usec
DE 18.00 usec
TE 298.0 K
D1 4.00000000 sec
D12 0.00002000 sec
D20 200.00000000 sec
TD0 1
SFO1 176.1056694 MHz
NUC1 13C
P1 12.00 usec
P13 2000.00 usec
P26 500.00 usec
PLW1 146.39999390 W
SPNAM[5] Crp80comp.4
SPOAL5 0.500
SPOFFS5 0 Hz
SPW5 42.94699860 W
SPNAM[8] Crp80,0.5,20.1
SPOAL8 0.500
SPOFFS8 0 Hz
SPW8 42.94699860 W
SFO2 700.2928012 MHz
NUC2 1H
CPDPRG[2 waltz16
PCPD2 65.00 usec
PLW2 14.67599964 W
PLW12 0.22231001 W

F2 - Processing parameters
SI 131072
SF 176.0879952 MHz
WDW EM
SSB 0
LB 2.00 Hz
GB 0
PC 1.40

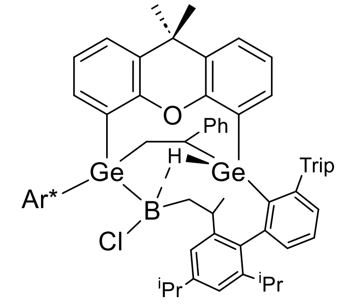


# impurity

Figure S16. $^{13}C\{^{1}H\}$ NMR spectrum of compound **3** (15 – 45 ppm).

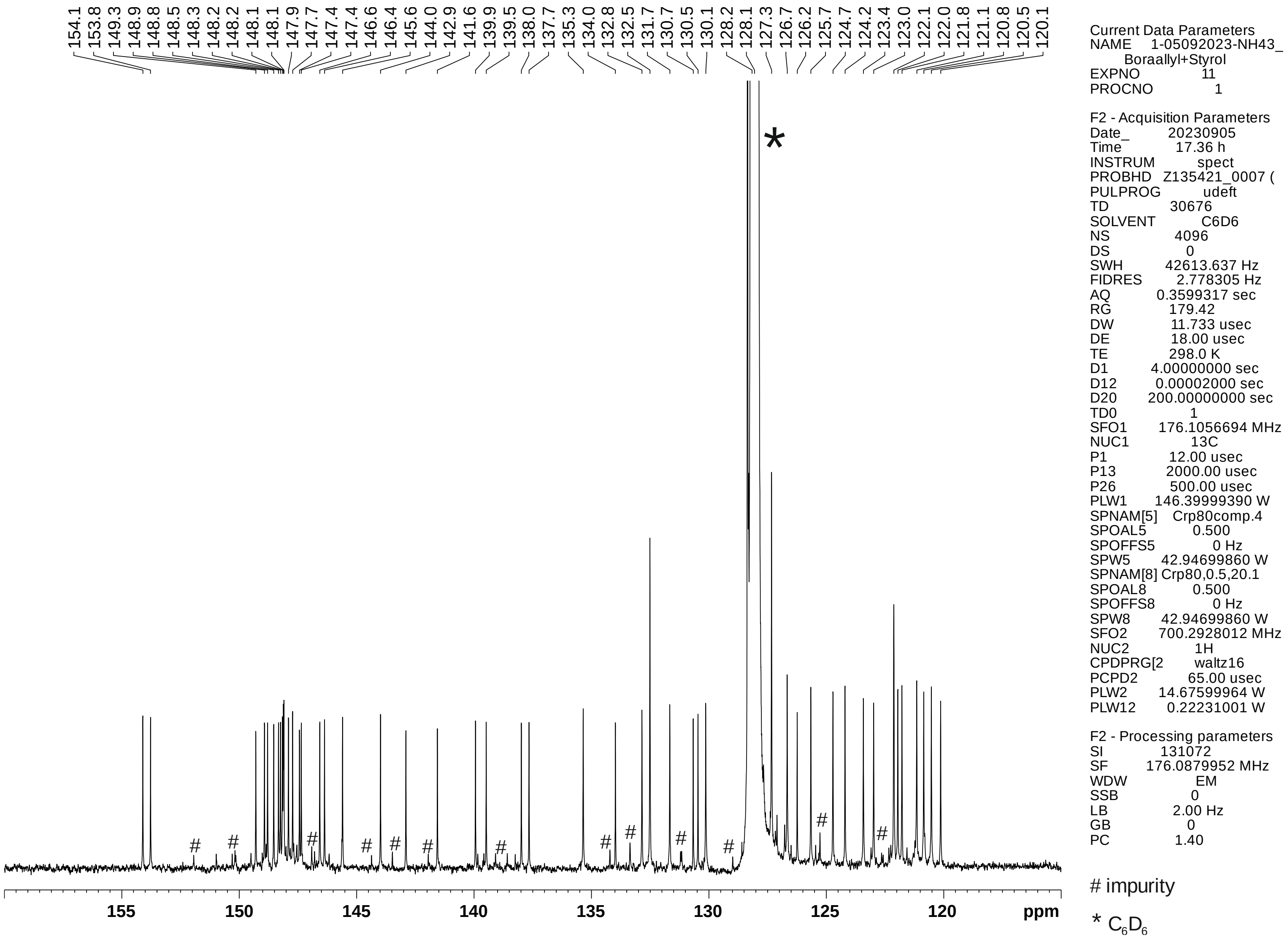


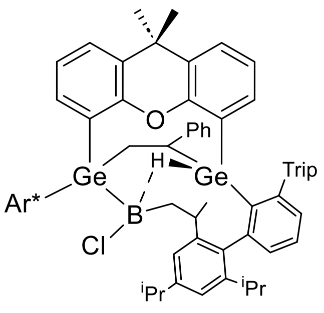


Figure S17. $^{13}C\{^{1}H\}$ NMR spectrum of compound **3** (115 – 160 ppm).

NMR spectra of compound **6**.

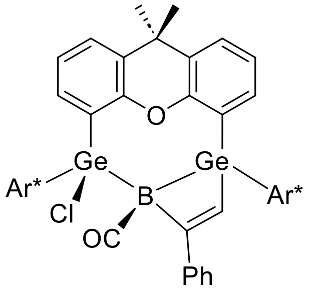


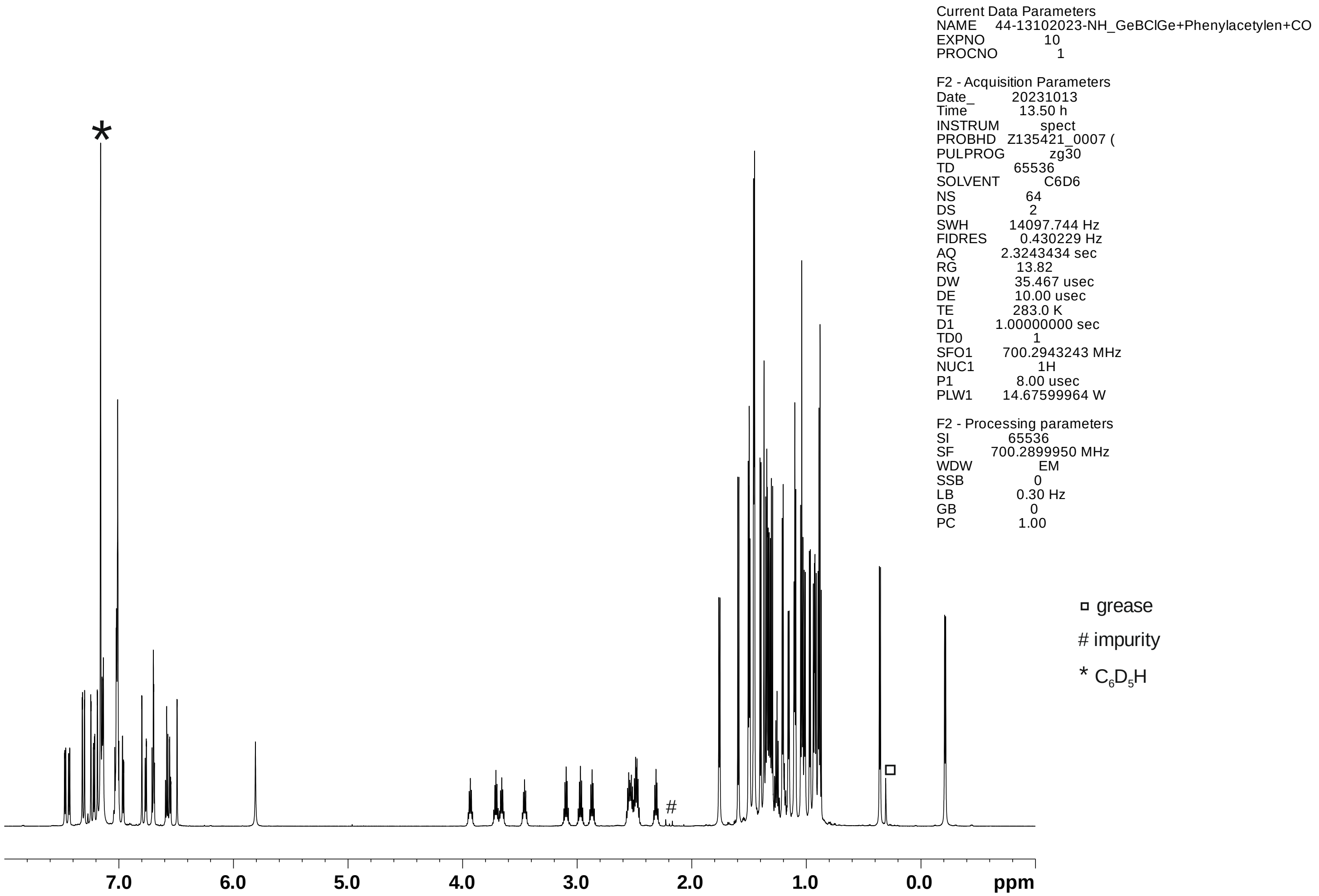


Figure S18. $^{1}H$ NMR spectrum of compound **6**.

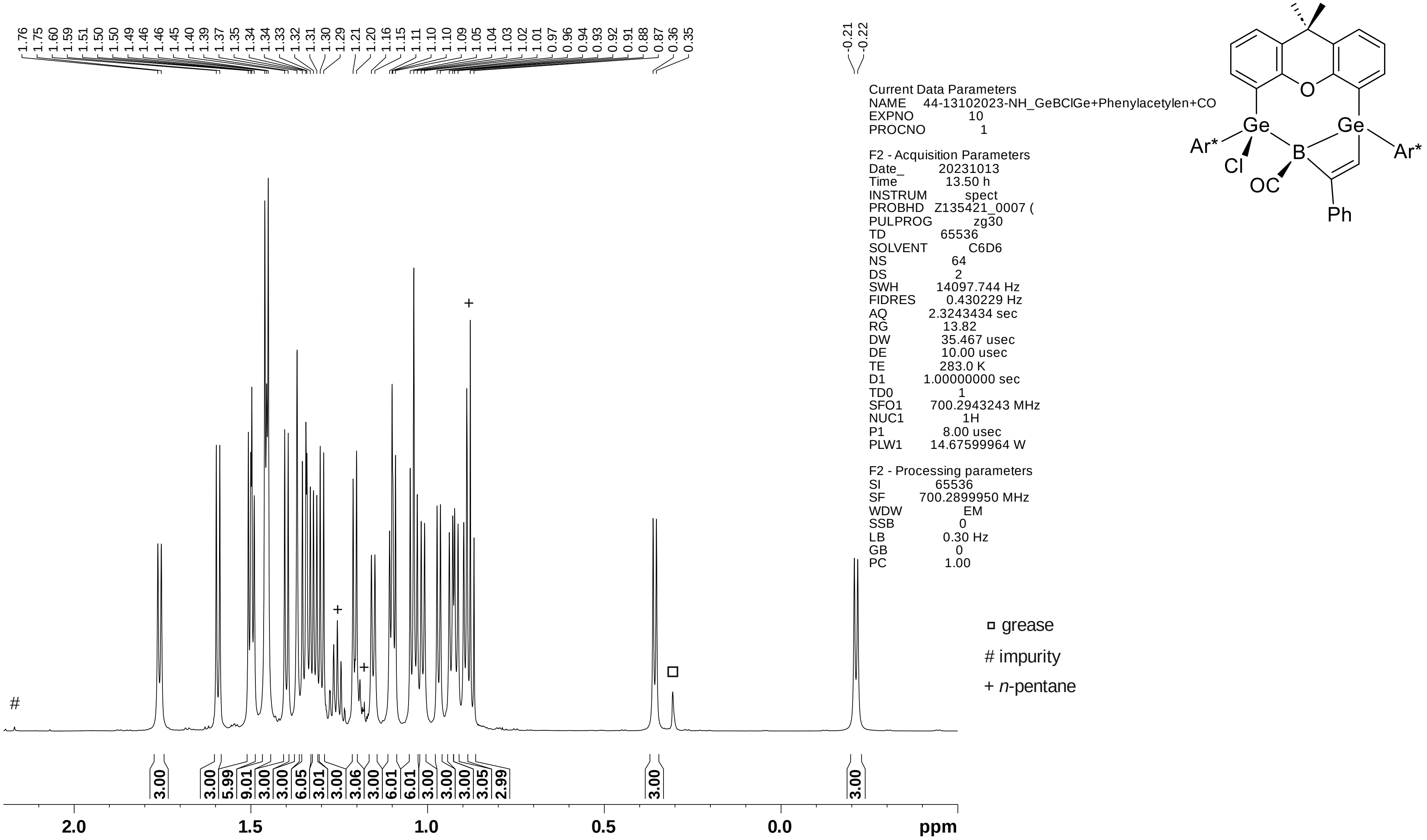


Figure S19. $^{1}$H NMR spectrum of compound **6** (−0.5 – 2.2 ppm).

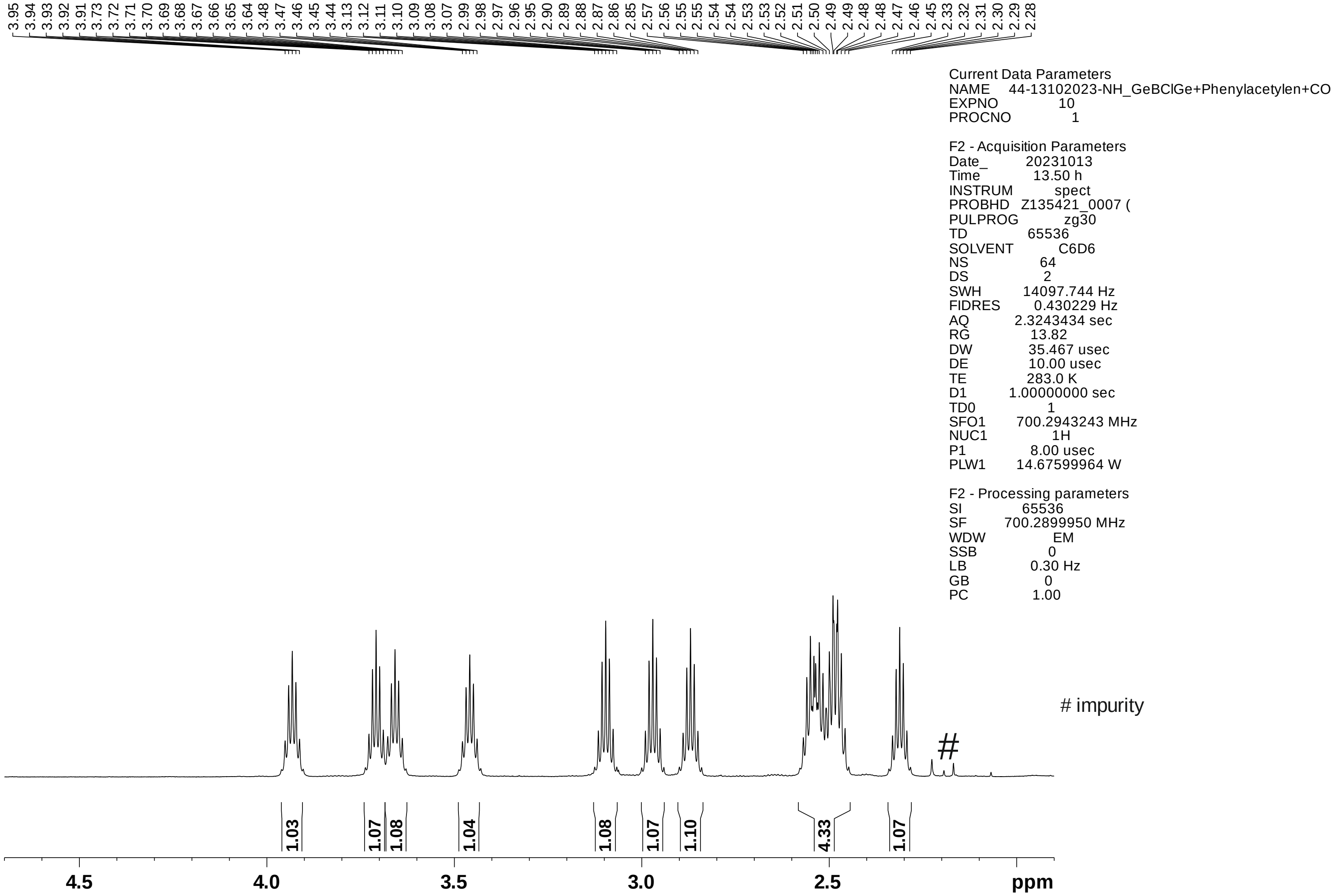


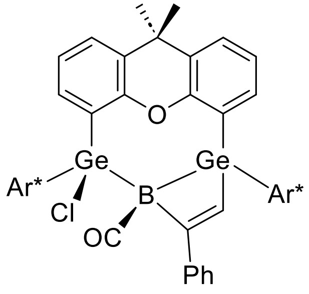


Figure S20. $^{1}$H NMR spectrum of compound **6** (1.9 – 4.7 ppm).

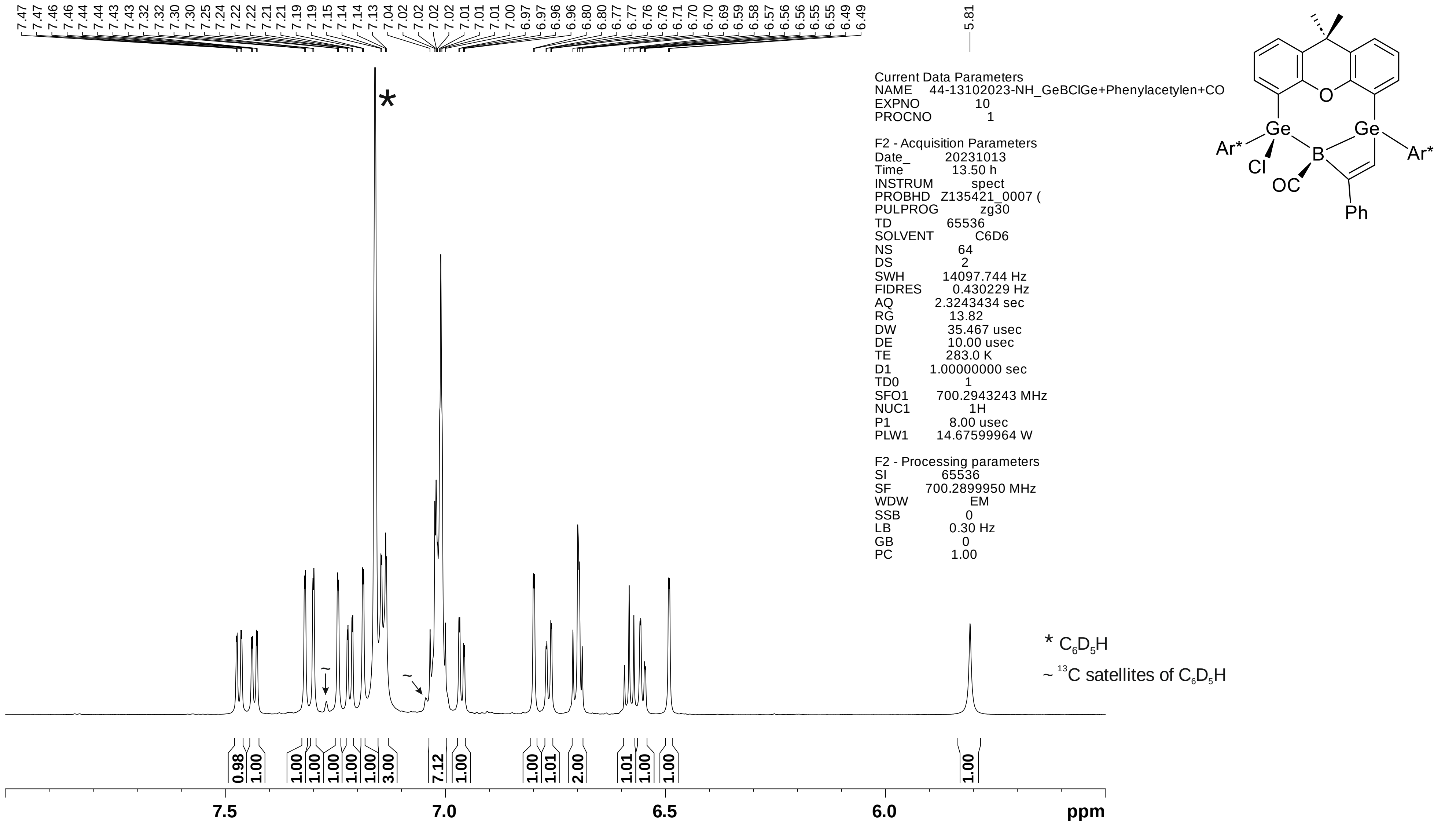


Figure S21. $^1$H NMR spectrum of compound **6** (5.5 – 8.0 ppm).

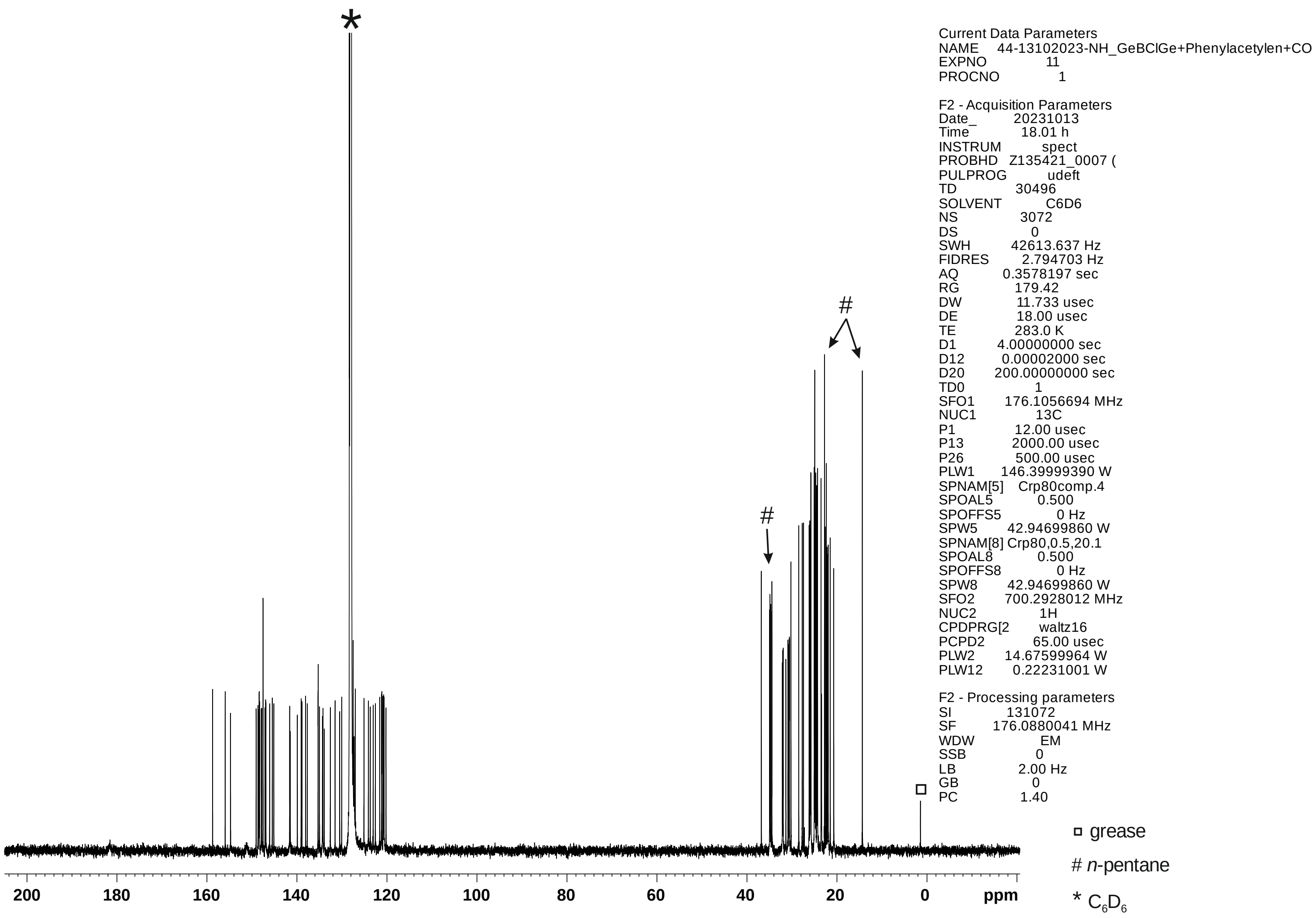


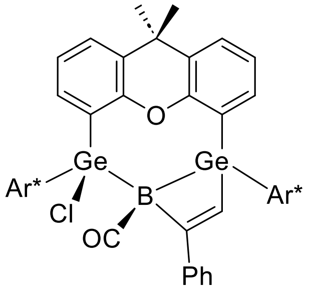


Figure S22. $^{13}C\{^{1}H\}$ NMR spectrum of compound **6**.

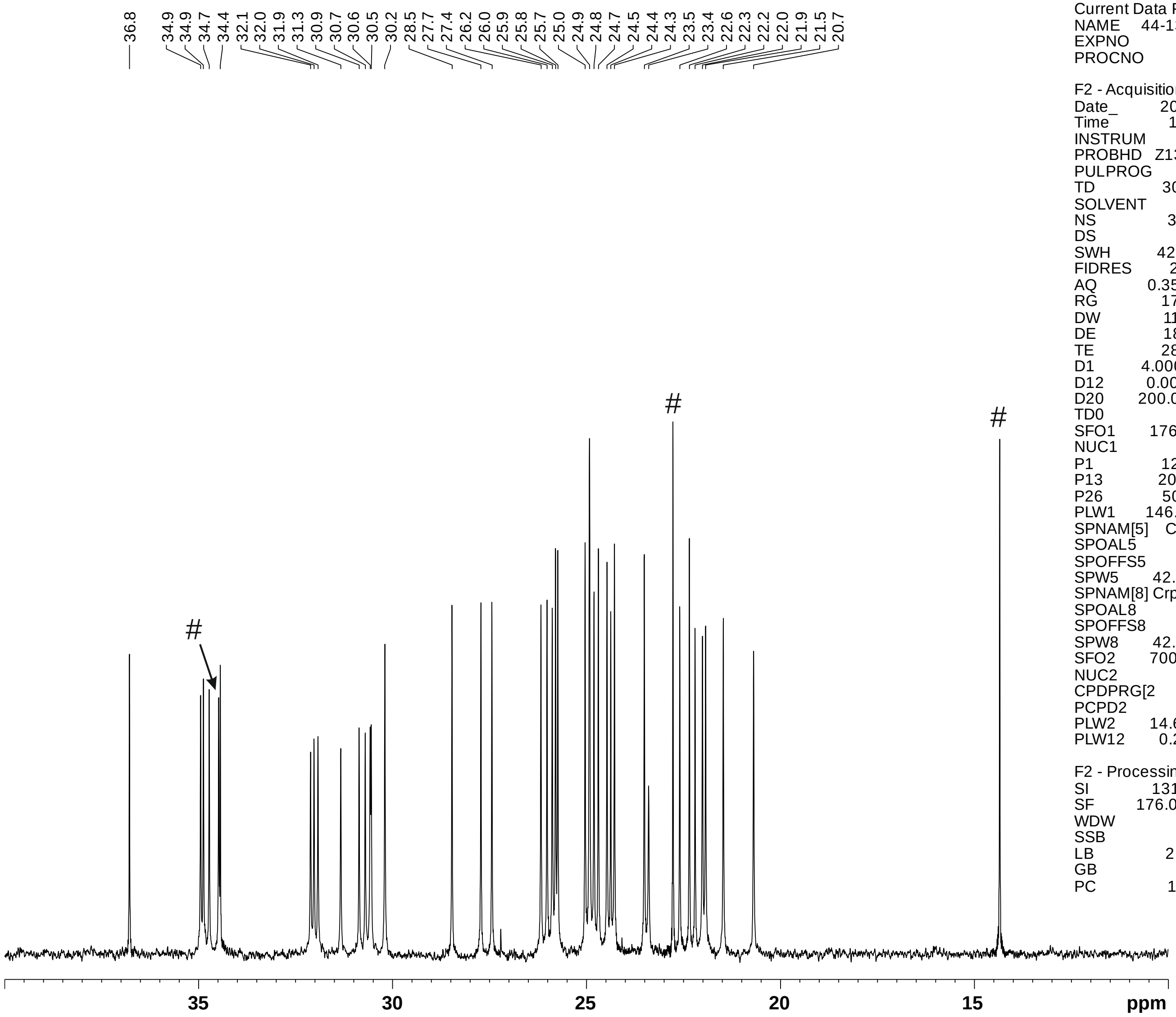


Current Data Parameters
NAME 44-13102023-NH_GeBClGe+Phenylacetylen+CC
EXPNO 11
PROCNO 1

F2 - Acquisition Parameters
Date_ 20231013
Time 18.01 h
INSTRUM spect
PROBHD Z135421_0007 (
PULPROG udeft
TD 30496
SOLVENT C6D6
NS 3072
DS 0
SWH 42613.637 Hz
FIDRES 2.794703 Hz
AQ 0.3578197 sec
RG 179.42
DW 11.733 usec
DE 18.00 usec
TE 283.0 K
D1 4.00000000 sec
D12 0.00002000 sec
D20 200.00000000 sec
TD0 1
SFO1 176.1056694 MHz
NUC1 13C
P1 12.00 usec
P13 2000.00 usec
P26 500.00 usec
PLW1 146.39999390 W
SPNAM[5] Crp80comp.4
SPOAL5 0.500
SPOFFS5 0 Hz
SPW5 42.94699860 W
SPNAM[8] Crp80,0.5,20.1
SPOAL8 0.500
SPOFFS8 0 Hz
SPW8 42.94699860 W
SFO2 700.2928012 MHz
NUC2 1H
CPDPRG[2 waltz16
PCPD2 65.00 usec
PLW2 14.67599964 W
PLW12 0.22231001 W

F2 - Processing parameters
SI 131072
SF 176.0880041 MHz
WDW EM
SSB 0
LB 2.00 Hz
GB 0
PC 1.40

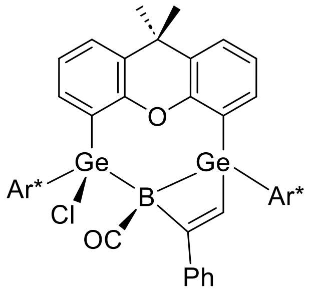


# *n*-pentane

Figure S23. $^{13}C\{^{1}H\}$ NMR spectrum of compound **6** (10 – 40 ppm).

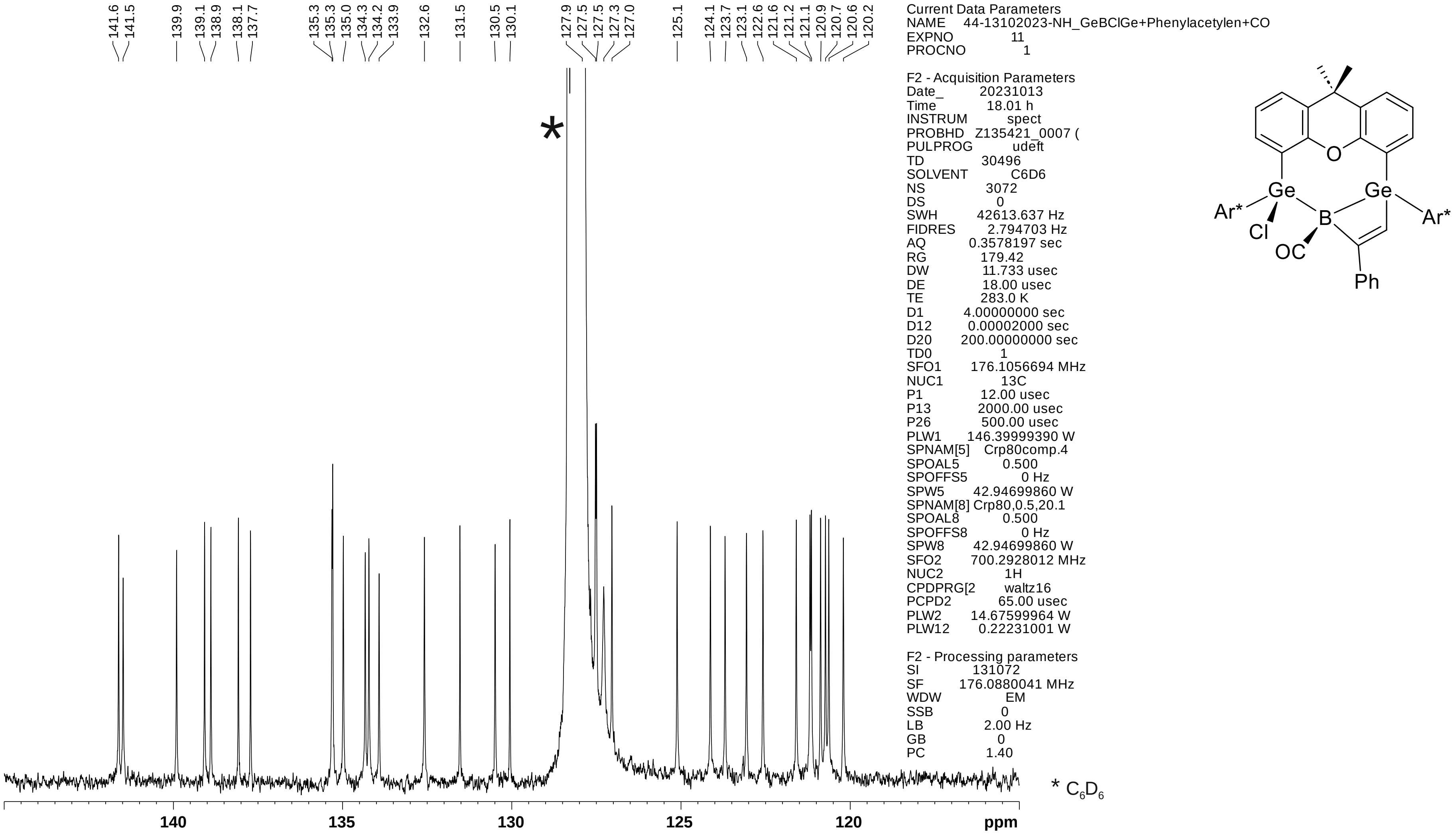


Figure S24. $^{13}C\{^{1}H\}$ NMR spectrum of compound **6** (115 – 145 ppm).

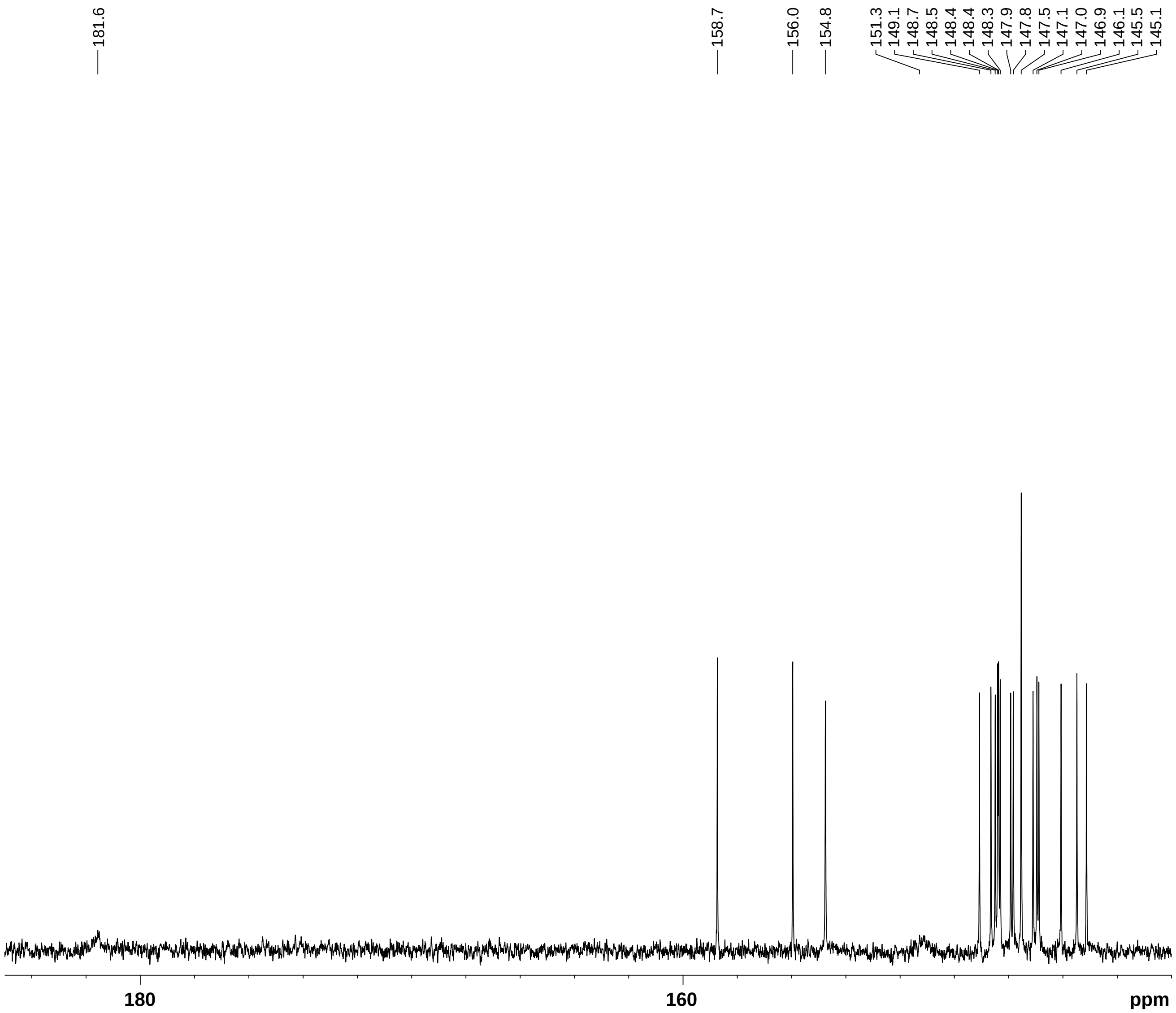


Current Data Parameters
NAME 44-13102023-NH_
GeBClGe+Phenylacetylen+CO
EXPNO 11
PROCNO 1

F2 - Acquisition Parameters
Date_ 20231013
Time 18.01 h
INSTRUM spect
PROBHD Z135421_0007 (
PULPROG udeft
TD 30496
SOLVENT C6D6
NS 3072
DS 0
SWH 42613.637 Hz
FIDRES 2.794703 Hz
AQ 0.3578197 sec
RG 179.42
DW 11.733 usec
DE 18.00 usec
TE 283.0 K
D1 4.00000000 sec
D12 0.00002000 sec
D20 200.00000000 sec
TD0 1
SFO1 176.1056694 MHz
NUC1 13C
P1 12.00 usec
P13 2000.00 usec
P26 500.00 usec
PLW1 146.39999390 W
SPNAM[5] Crp80comp.4
SPOAL5 0.500
SPOFFS5 0 Hz
SPW5 42.94699860 W
SPNAM[8] Crp80,0.5,20.1
SPOAL8 0.500
SPOFFS8 0 Hz
SPW8 42.94699860 W
SFO2 700.2928012 MHz
NUC2 1H
CPDPRG[2 waltz16
PCPD2 65.00 usec
PLW2 14.67599964 W
PLW12 0.22231001 W

F2 - Processing parameters
SI 131072
SF 176.0880041 MHz
WDW EM
SSB 0
LB 2.00 Hz
GB 0
PC 1.40

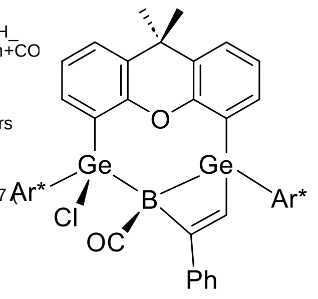


Figure S25. $^{13}C\{^{1}H\}$ NMR spectrum of compound **6** (142 – 185 ppm).

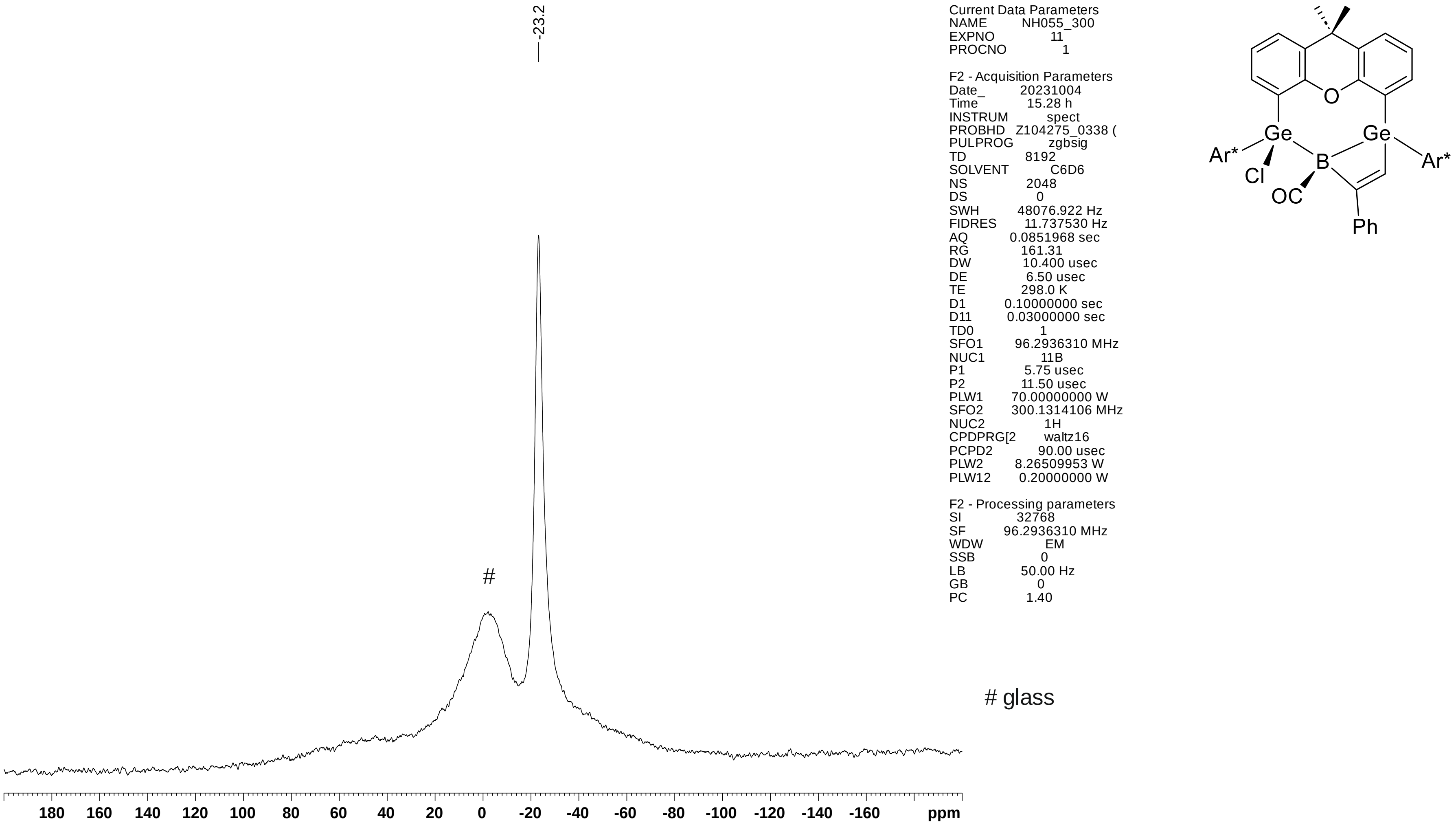


Figure S26. $^{11}B\{^{1}H\}$ NMR spectrum of compound **6**.

# NMR spectra of compound **7**.

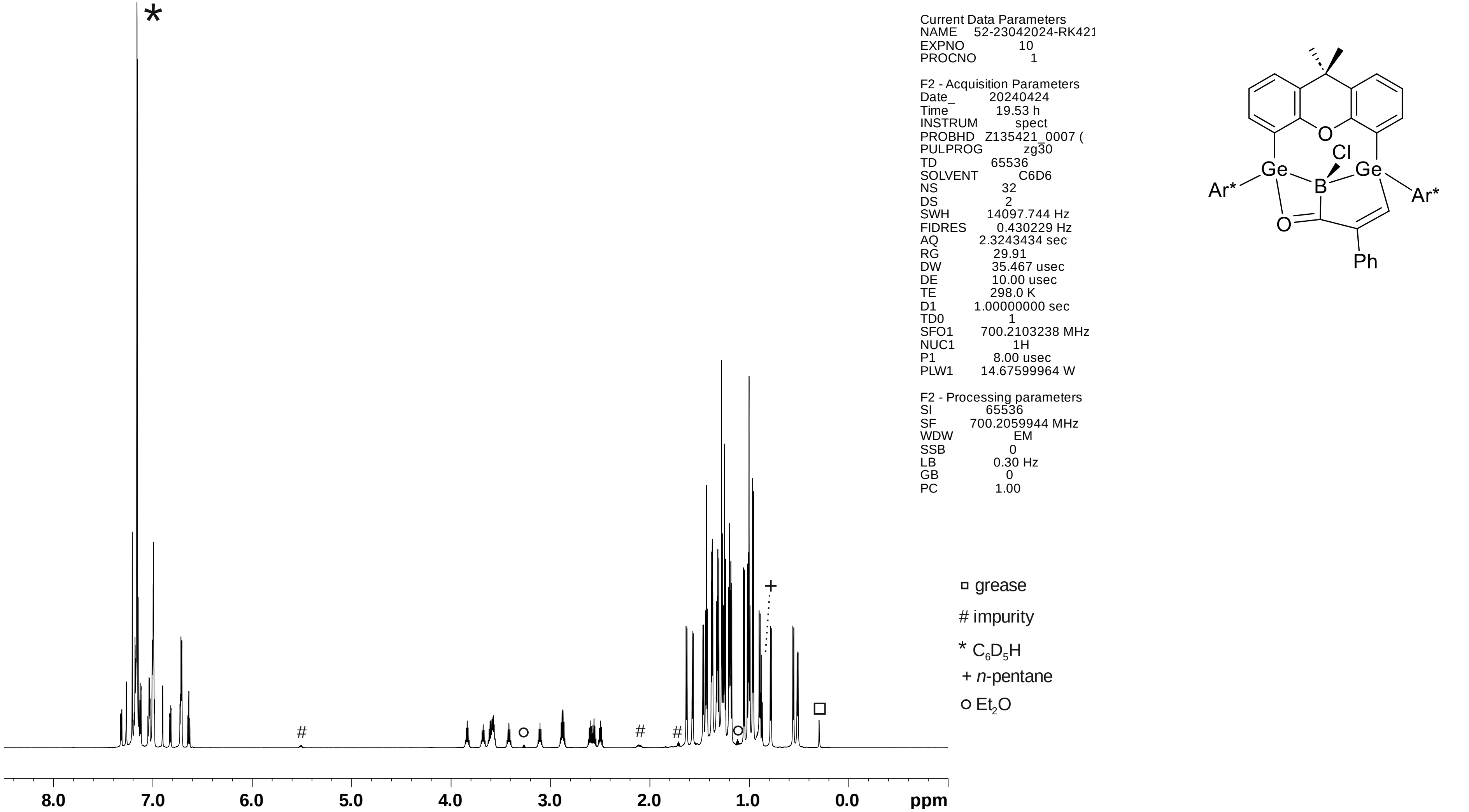


Figure S27. $^1$H NMR spectrum of compound **7**.

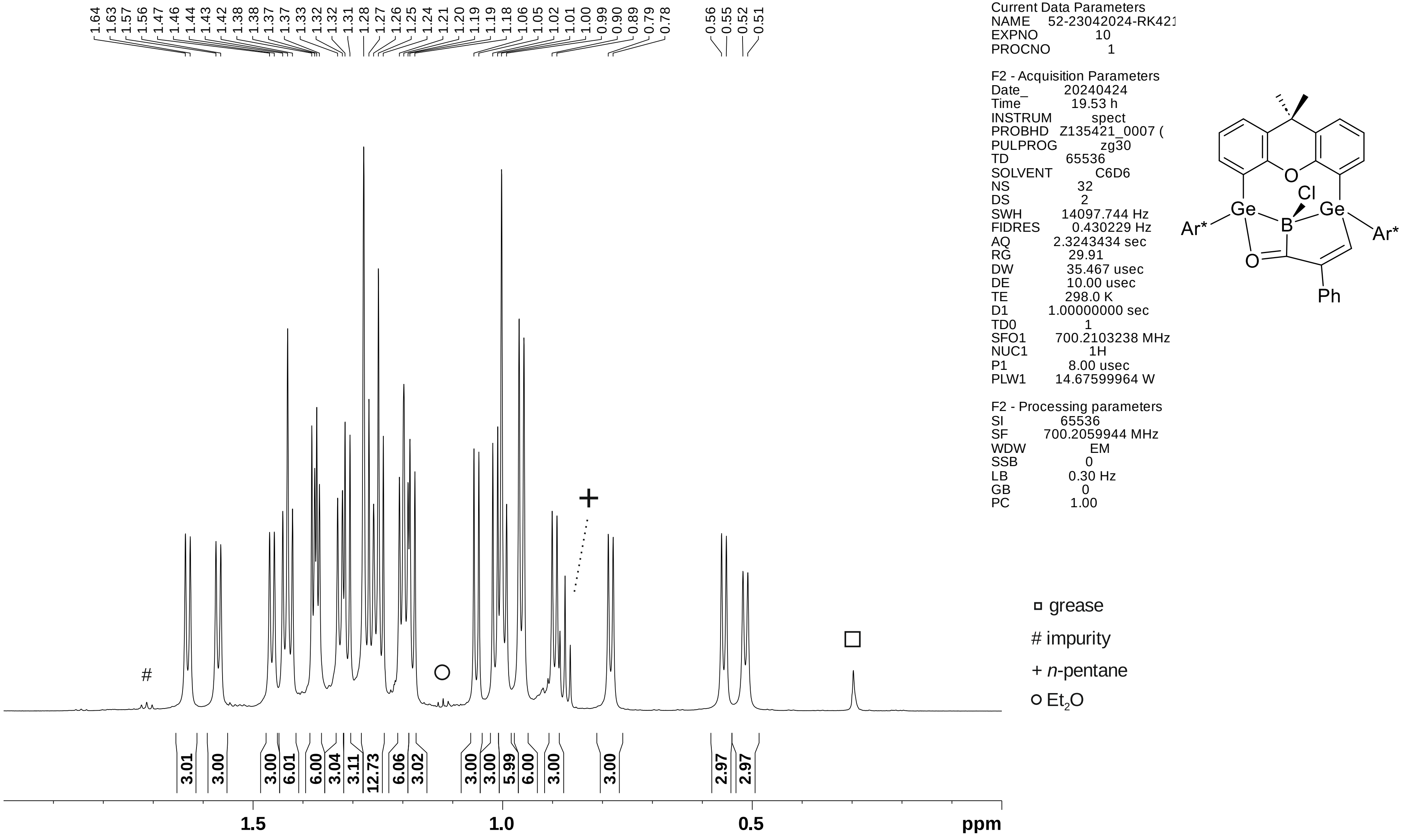


Figure S28. $^{1}H$ NMR spectrum of compound **7** (0 – 2.0 ppm).

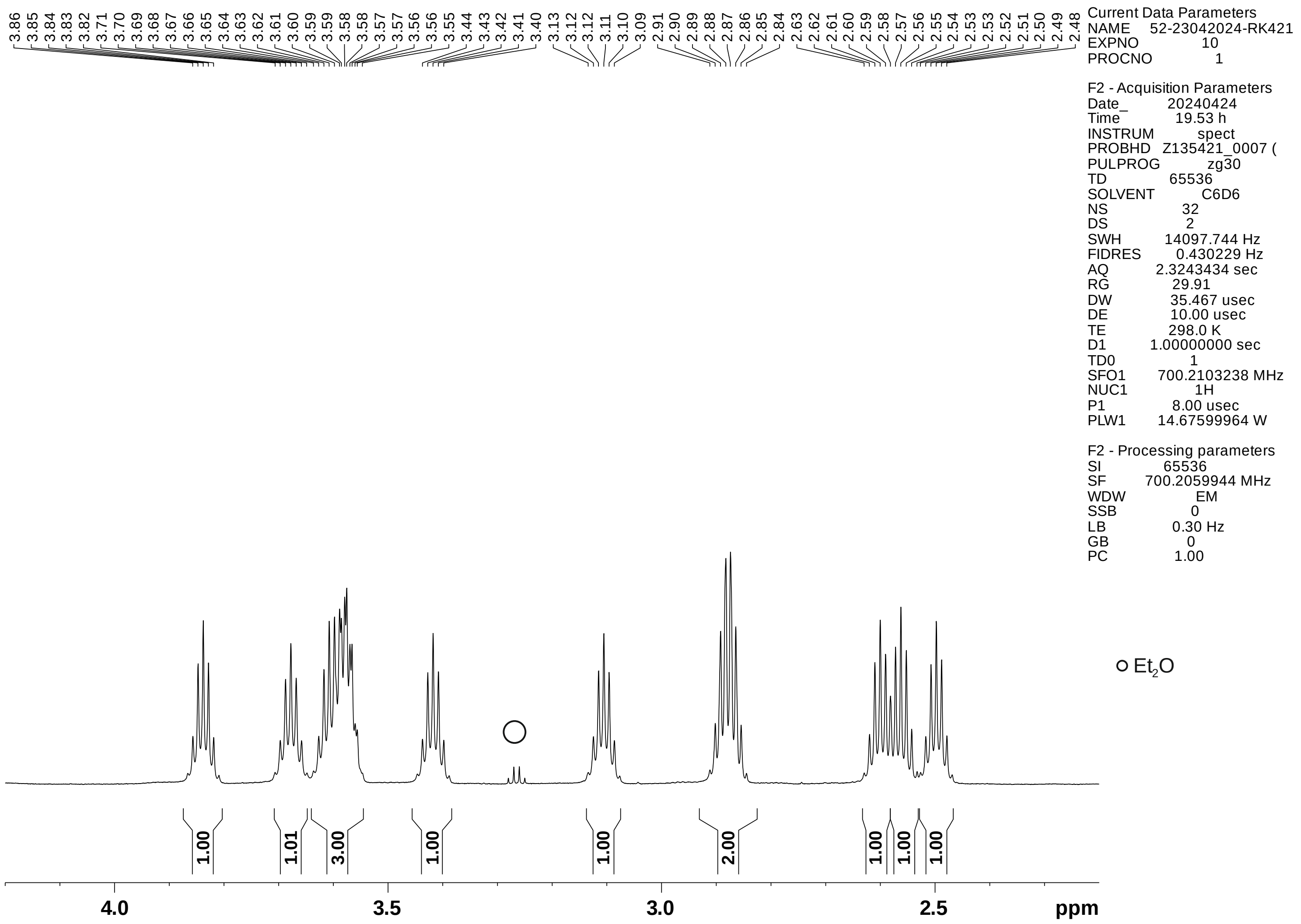


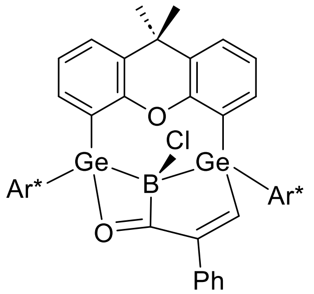


Figure S29. $^{1}$H NMR spectrum of compound **7** (2.2 – 4.2).

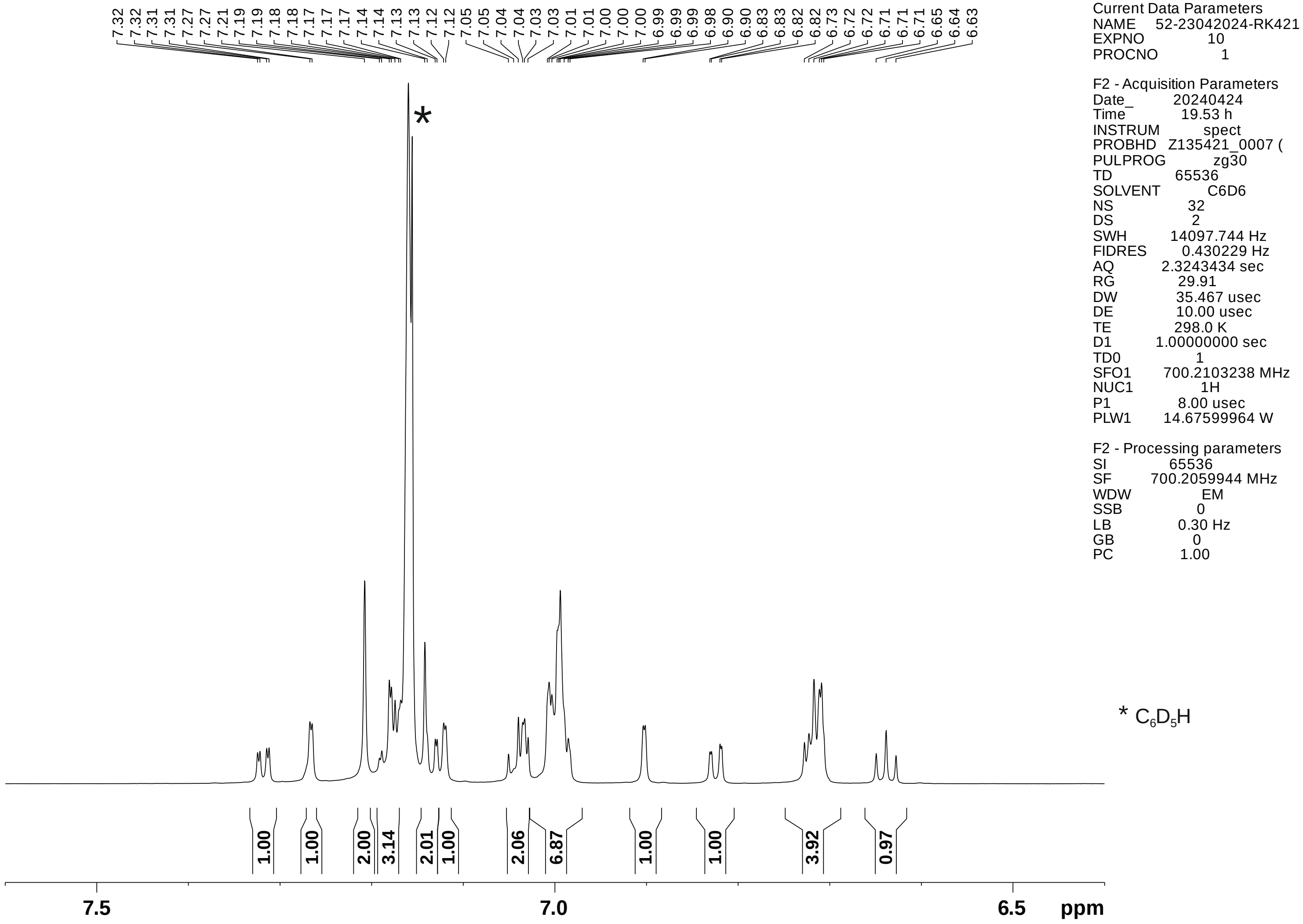


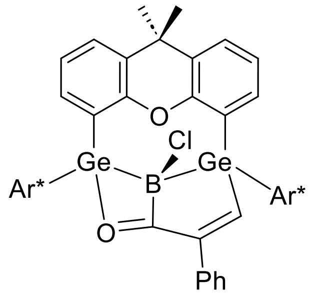


Figure S30. $^1$H NMR spectrum of compound **7** (6.4 – 7.6 ppm).

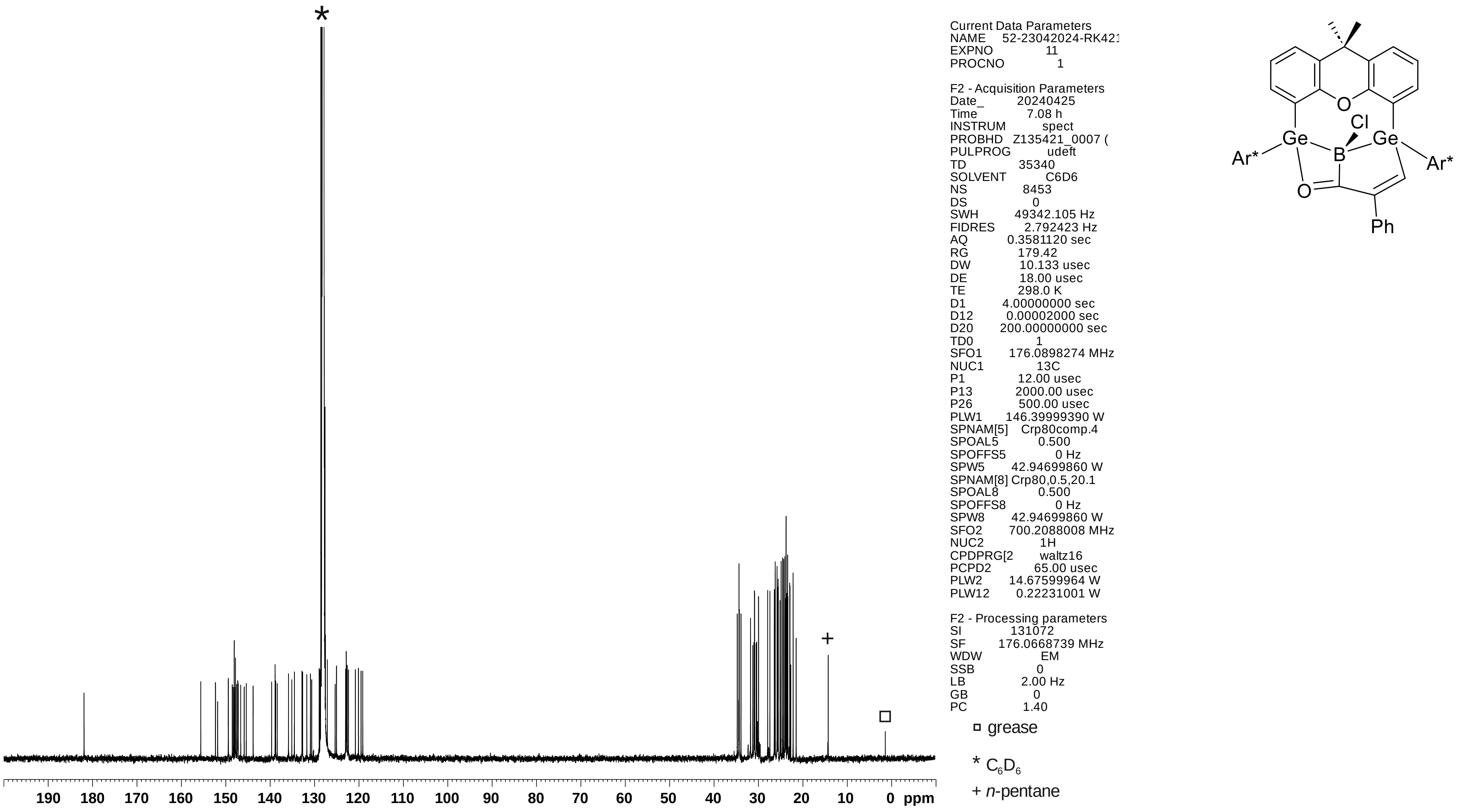


Figure S31. $^{13}C\{^{1}H\}$ NMR spectrum of compound **7**.

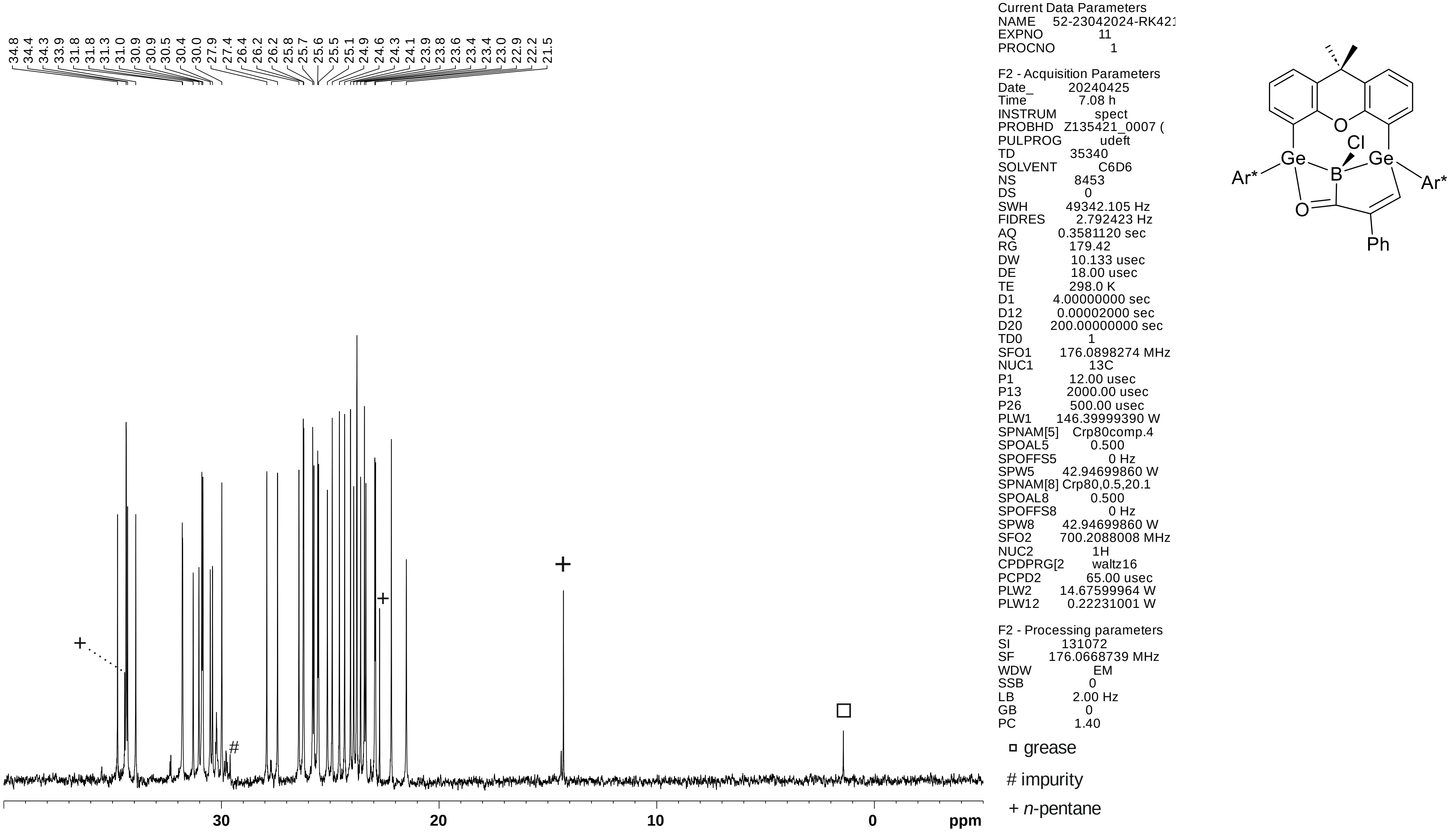


Figure S32. $^{13}C\{^{1}H\}$ NMR spectrum of compound **7** (−5 – 40 ppm).

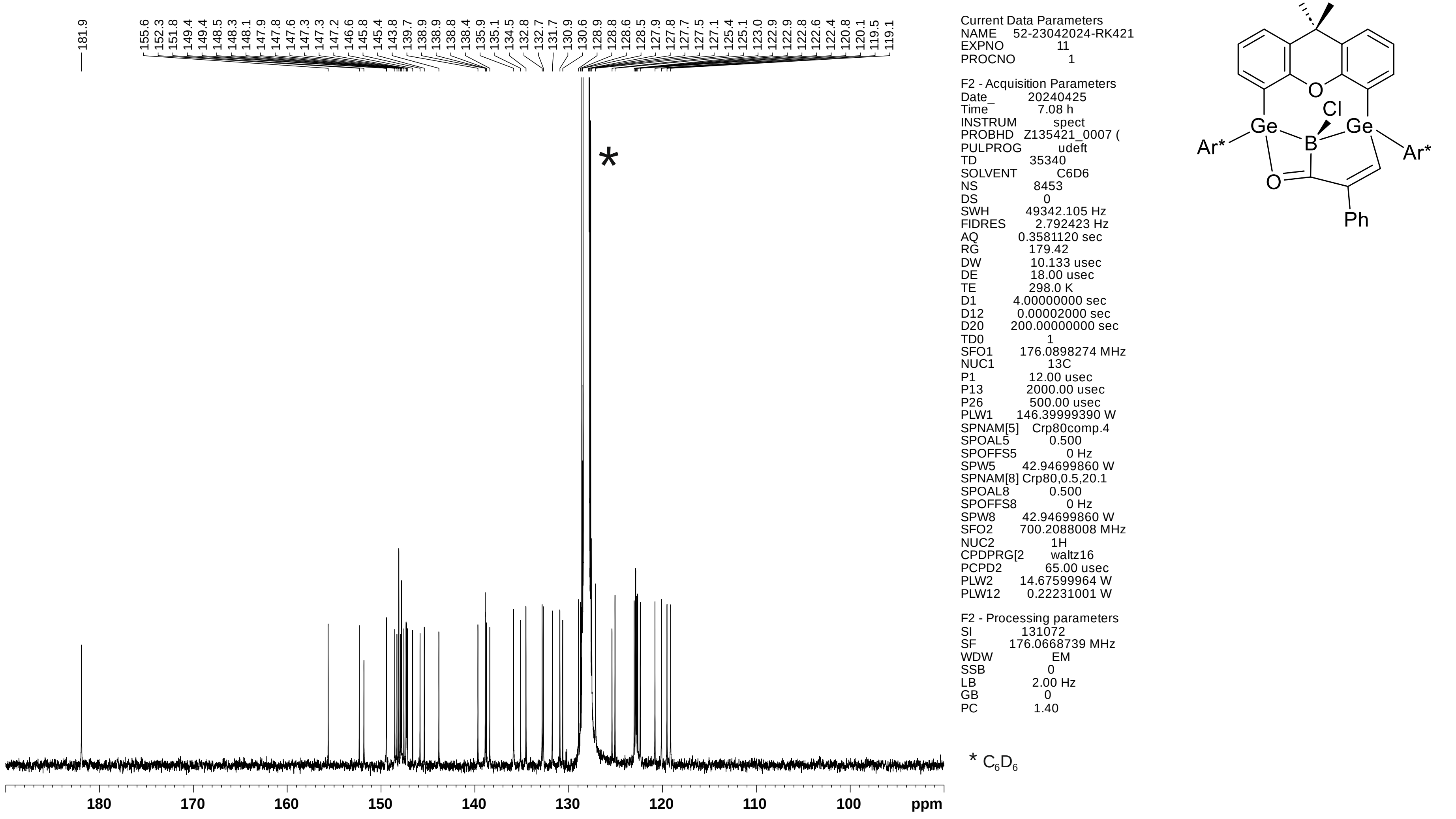


Figure S33. $^{13}C\{^{1}H\}$ NMR spectrum of compound **7** (90 – 190 ppm).

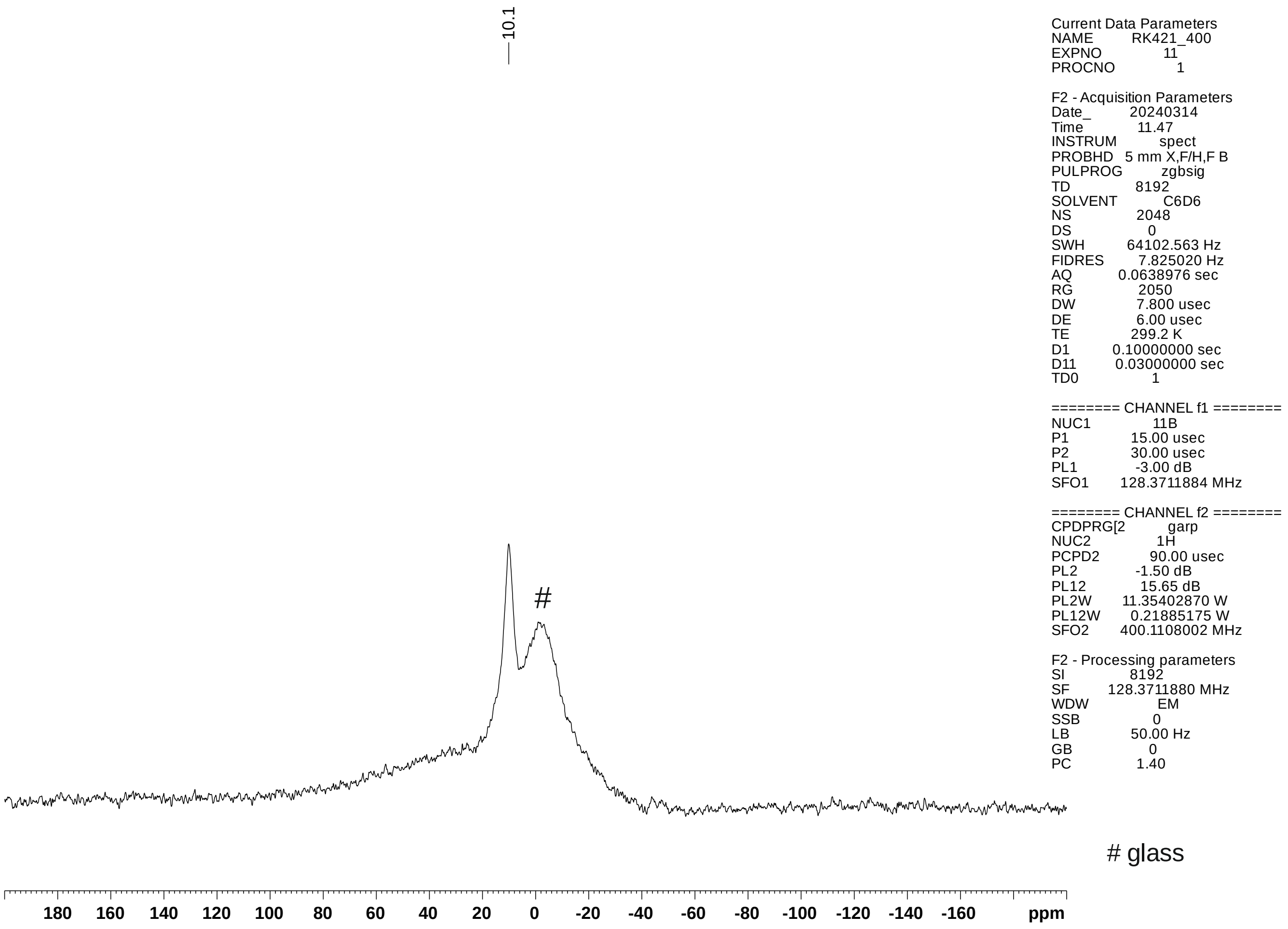


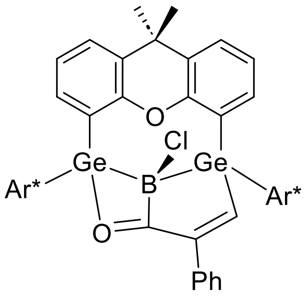


# glass

Figure S34. $^{11}B\{^{1}H\}$ NMR spectrum of compound **7**.

NMR spectra of compound **8**.

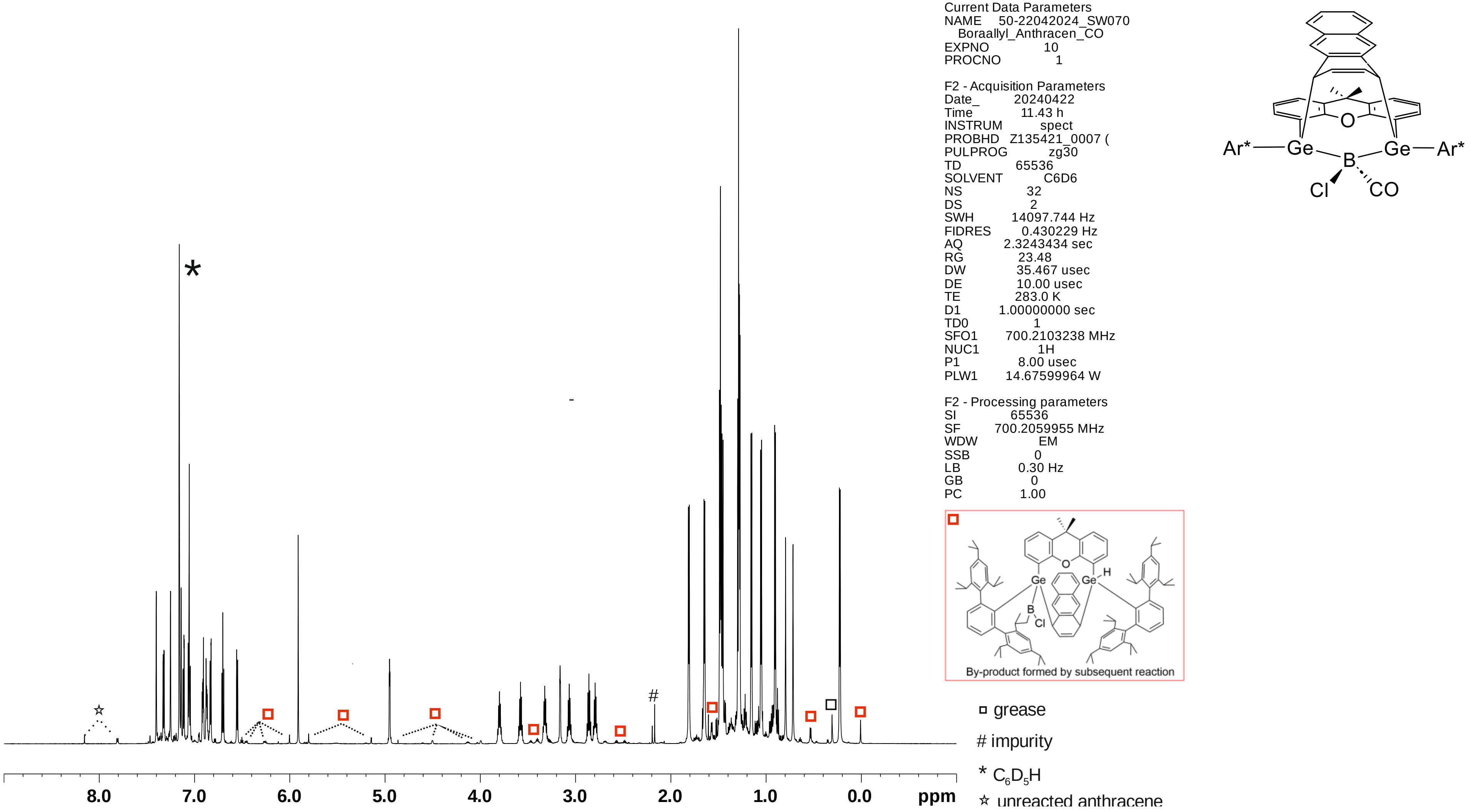


Figure S35. $^{1}$H NMR spectrum of compound **8**.

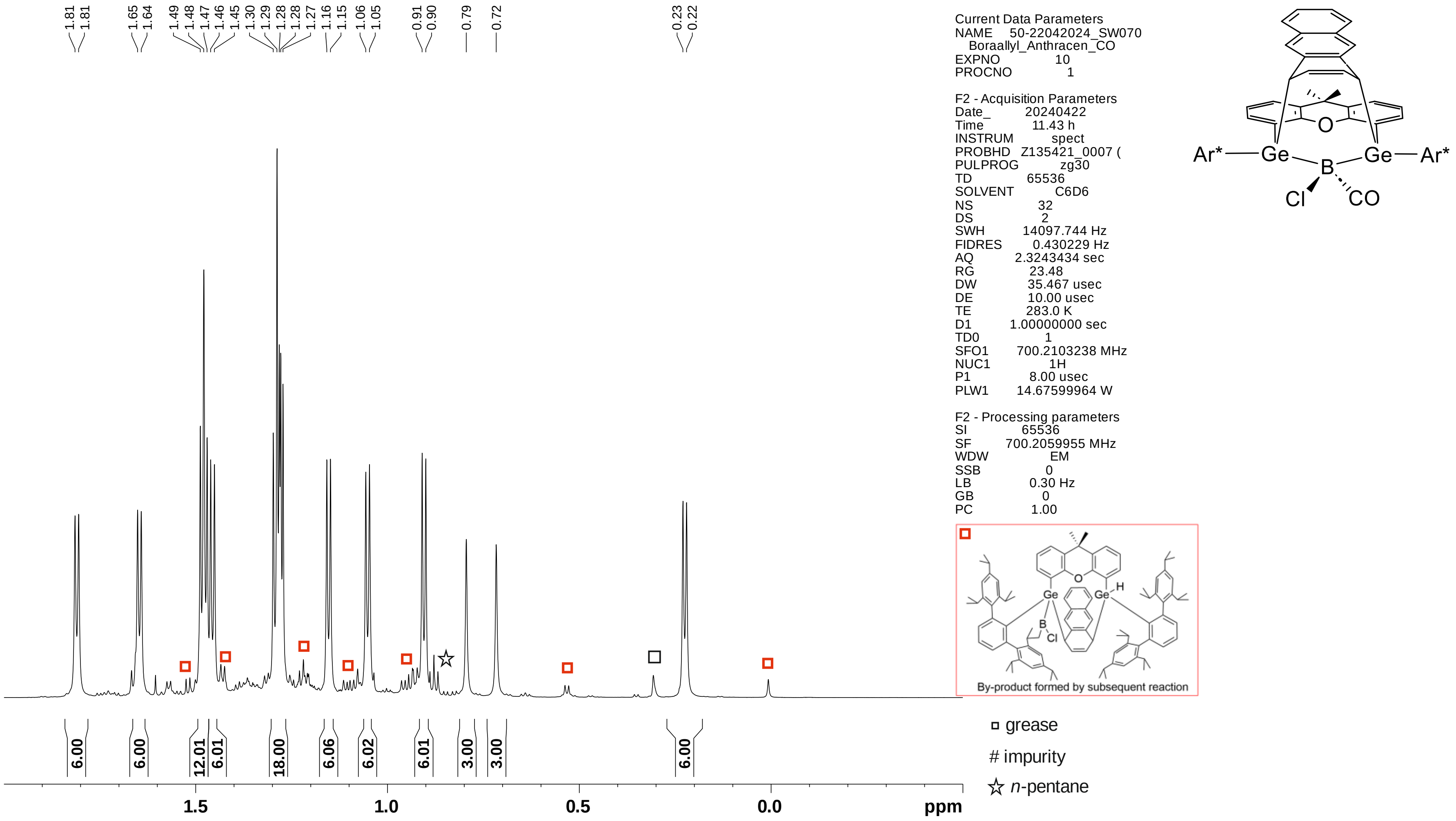


Figure S36. $^{1}$H NMR spectrum of compound **8** (−0.5 – 2.0 ppm).

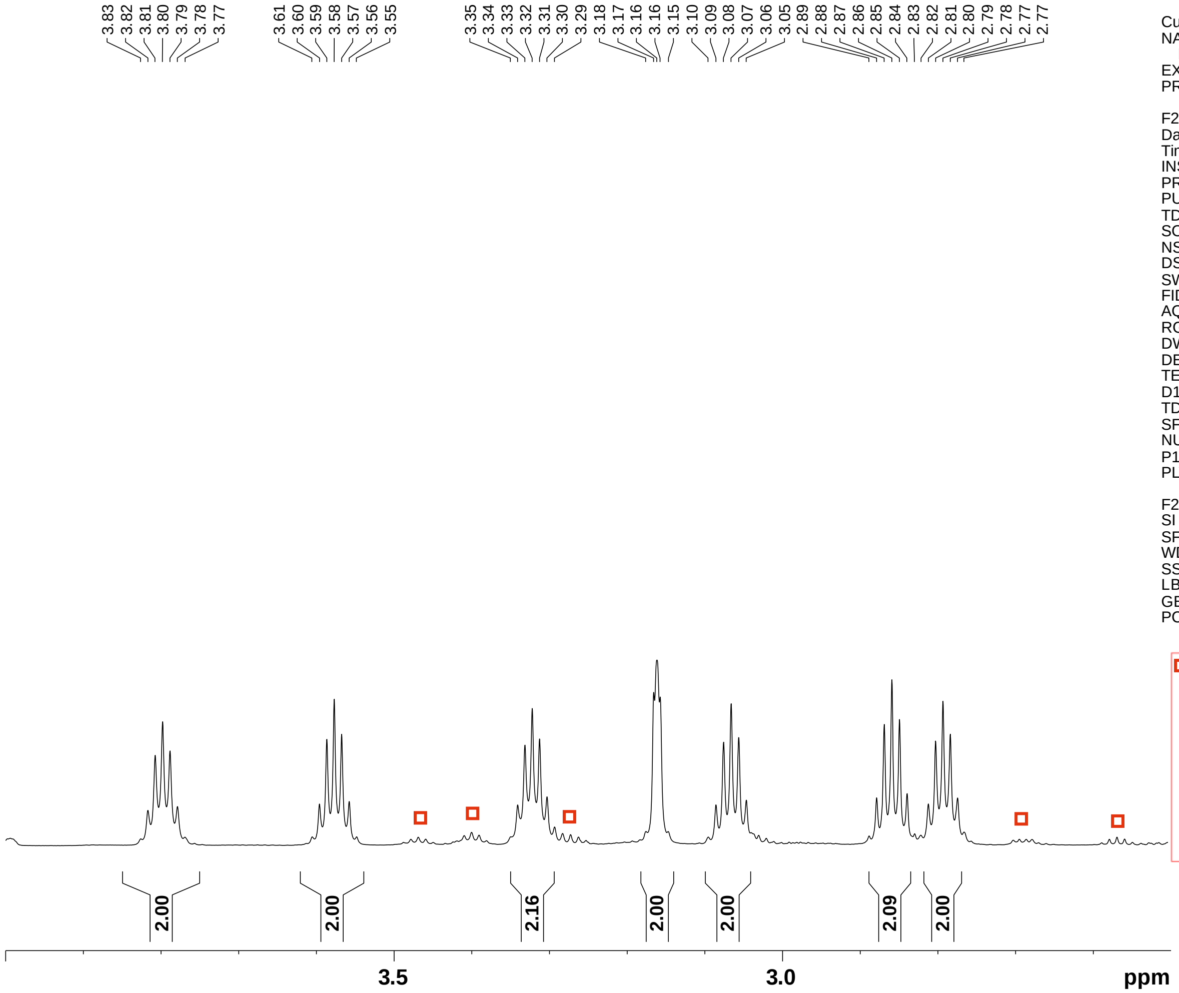


Current Data Parameters
NAME 50-22042024_SW070
Boraallyl_Anthracen_CO
EXPNO 10
PROCNO 1

F2 - Acquisition Parameters
Date_ 20240422
Time 11.43 h
INSTRUM spect
PROBHD Z135421_0007 (
PULPROG zg30
TD 65536
SOLVENT C6D6
NS 32
DS 2
SWH 14097.744 Hz
FIDRES 0.430229 Hz
AQ 2.3243434 sec
RG 23.48
DW 35.467 usec
DE 10.00 usec
TE 283.0 K
D1 1.00000000 sec
TD0 1
SFO1 700.2103238 MHz
NUC1 1H
P1 8.00 usec
PLW1 14.67599964 W

F2 - Processing parameters
SI 65536
SF 700.2059955 MHz
WDW EM
SSB 0
LB 0.30 Hz
GB 0
PC 1.00

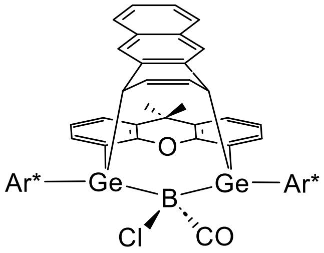


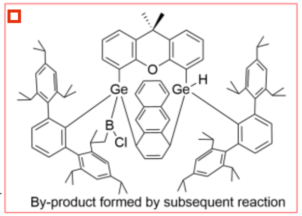


# impurity

Figure S37. $^{1}$H NMR spectrum of compound **8** (2.5 – 4.0 ppm).

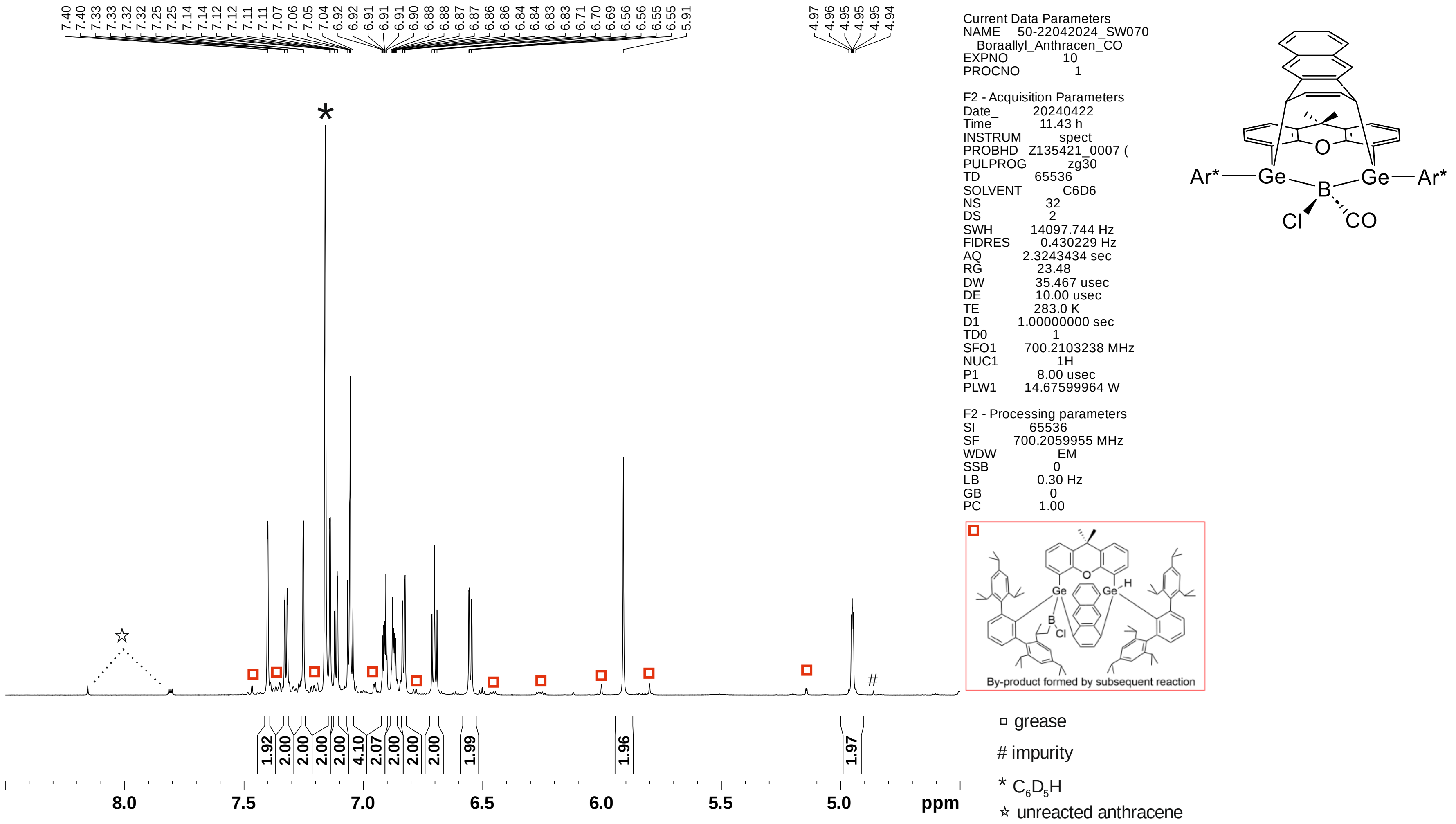


Figure S38. $^{1}$H NMR spectrum of compound **8** (4.5 – 8.5 ppm).

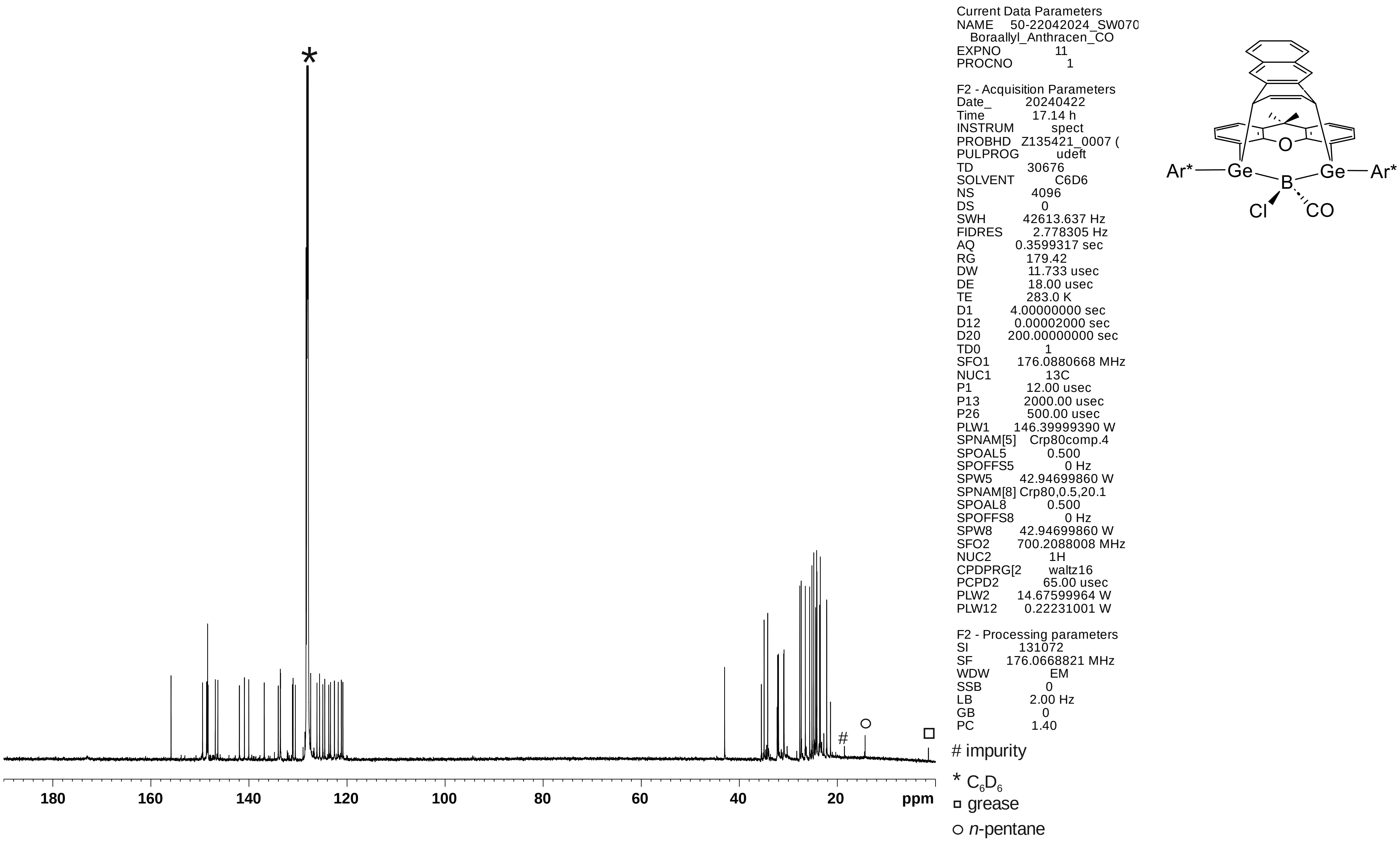


Figure S39. $^{13}C\{^1H\}$ NMR spectrum of compound **8**.

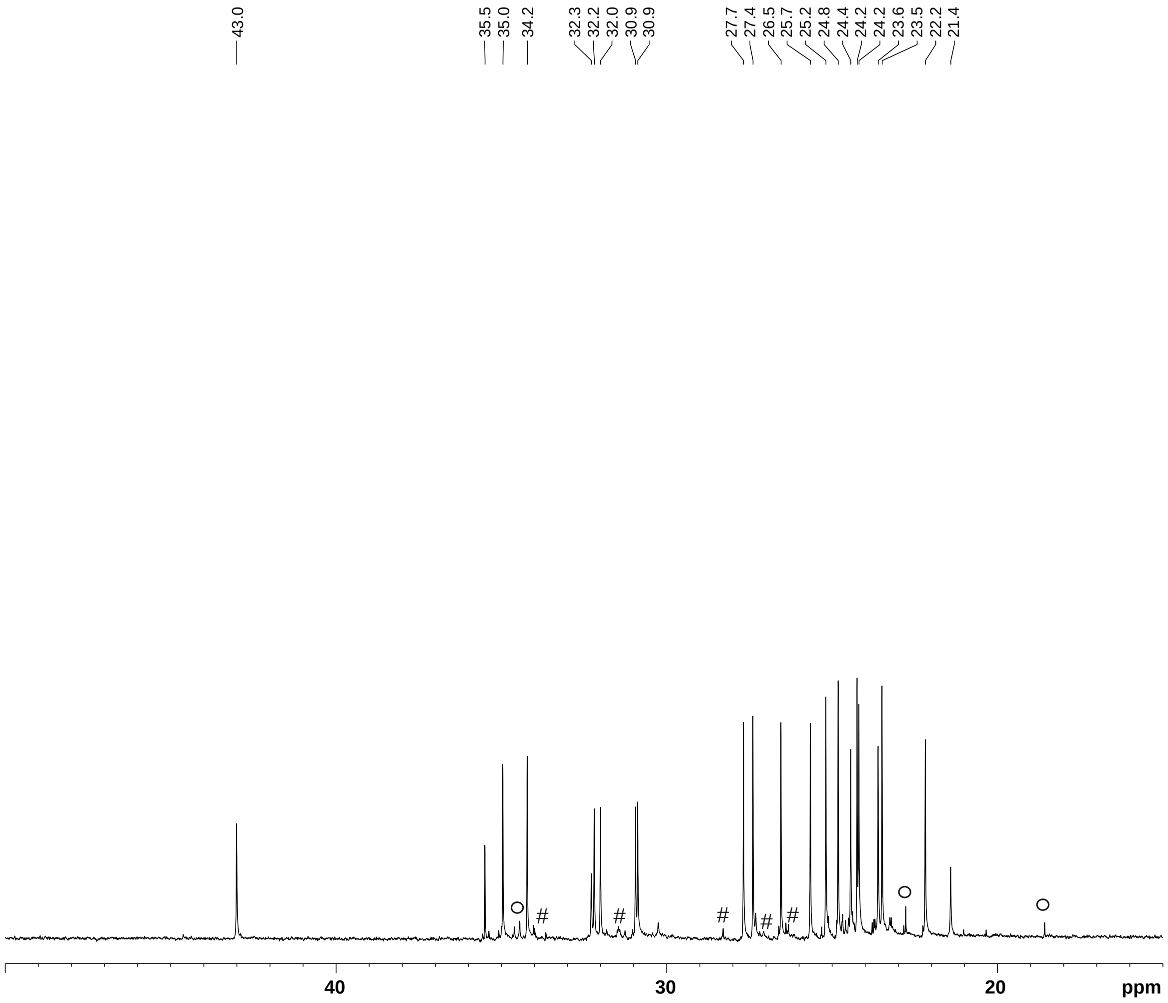


Current Data Parameters
NAME 50-22042024_SW070
Boraallyl_Anthracen_CO
EXPNO 11
PROCNO 1

F2 - Acquisition Parameters
Date_ 20240422
Time 17.14 h
INSTRUM spect
PROBHD Z135421_0007 (
PULPROG udeft
TD 30676
SOLVENT C6D6
NS 4096
DS 0
SWH 42613.637 Hz
FIDRES 2.778305 Hz
AQ 0.3599317 sec
RG 179.42
DW 11.733 usec
DE 18.00 usec
TE 283.0 K
D1 4.00000000 sec
D12 0.00002000 sec
D20 200.00000000 sec
TD0 1
SFO1 176.0880668 MHz
NUC1 13C
P1 12.00 usec
P13 2000.00 usec
P26 500.00 usec
PLW1 146.39999390 W
SPNAM[5] Crp80comp.4
SPOAL5 0.500
SPOFFS5 0 Hz
SPW5 42.94699860 W
SPNAM[8] Crp80,0.5,20.1
SPOAL8 0.500
SPOFFS8 0 Hz
SPW8 42.94699860 W
SFO2 700.2088008 MHz
NUC2 1H
CPDPRG[2 waltz16
PCPD2 65.00 usec
PLW2 14.67599964 W
PLW12 0.22231001 W

F2 - Processing parameters
SI 131072
SF 176.0668821 MHz
WDW EM
SSB 0
LB 2.00 Hz
GB 0
PC 1.40

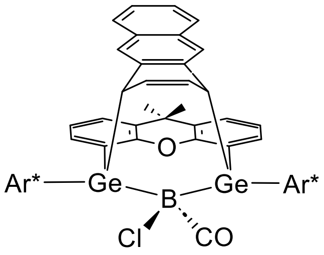


Figure S40. $^{13}C\{^{1}H\}$ NMR spectrum of compound **8** (15 – 50 ppm).

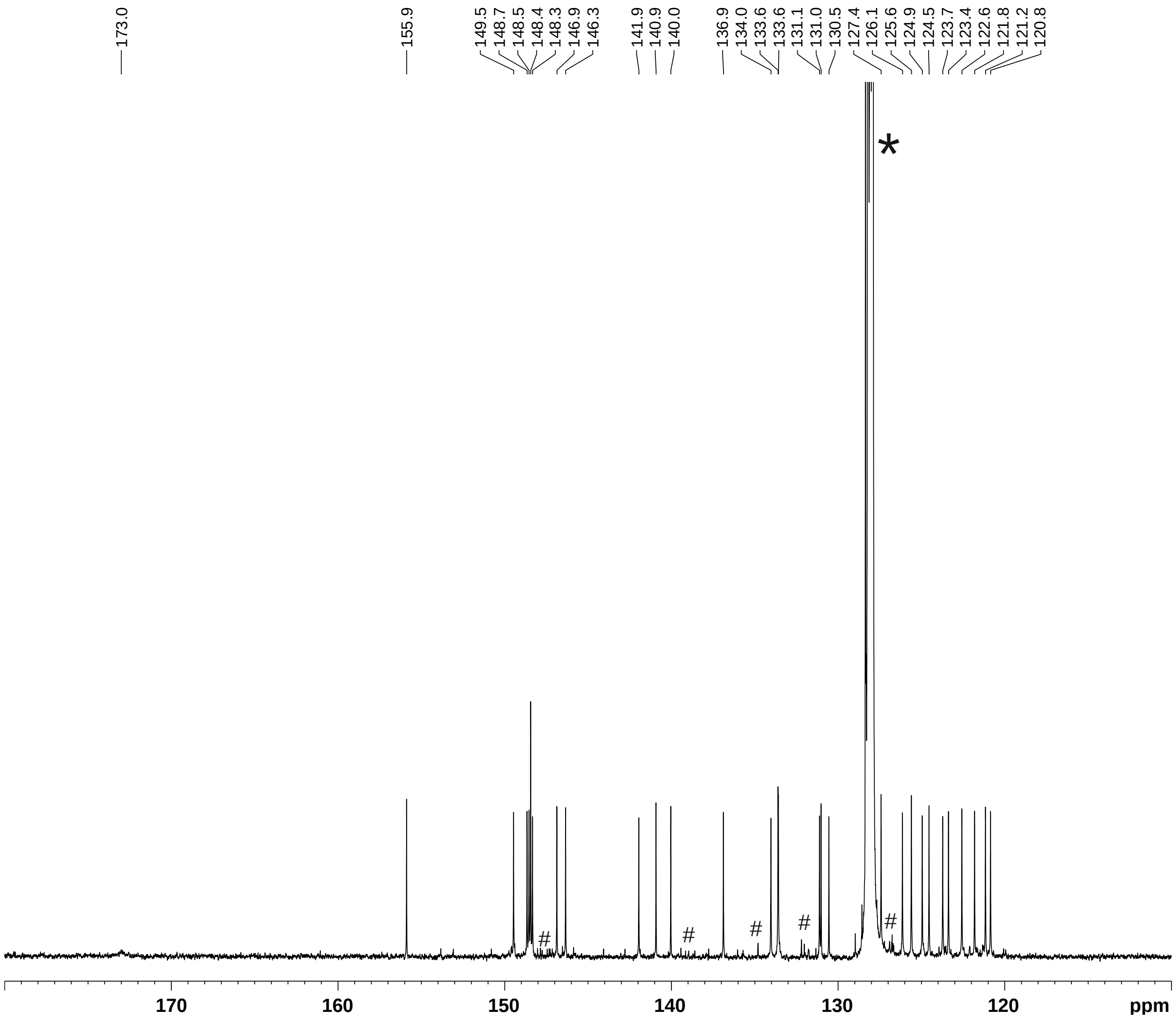


Current Data Parameters
NAME 50-22042024_SW070
Boraallyl_Anthracen_CO
EXPNO 11
PROCNO 1

F2 - Acquisition Parameters
Date_ 20240422
Time 17.14 h
INSTRUM spect
PROBHD Z135421_0007 (
PULPROG udeft
TD 30676
SOLVENT C6D6
NS 4096
DS 0
SWH 42613.637 Hz
FIDRES 2.778305 Hz
AQ 0.3599317 sec
RG 179.42
DW 11.733 usec
DE 18.00 usec
TE 283.0 K
D1 4.00000000 sec
D12 0.00002000 sec
D20 200.00000000 sec
TD0 1
SFO1 176.0880668 MHz
NUC1 13C
P1 12.00 usec
P13 2000.00 usec
P26 500.00 usec
PLW1 146.39999390 W
SPNAM[5] Crp80comp.4
SPOAL5 0.500
SPOFFS5 0 Hz
SPW5 42.94699860 W
SPNAM[8] Crp80,0.5,20.1
SPOAL8 0.500
SPOFFS8 0 Hz
SPW8 42.94699860 W
SFO2 700.2088008 MHz
NUC2 1H
CPDPRG[2 waltz16
PCPD2 65.00 usec
PLW2 14.67599964 W
PLW12 0.22231001 W

F2 - Processing parameters
SI 131072
SF 176.0668821 MHz
WDW EM
SSB 0
LB 2.00 Hz
GB 0
PC 1.40

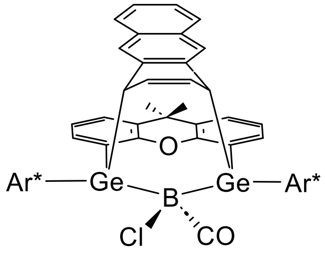


# impurity/by-product see $^{1}$H

* $C_6D_6$

Figure S41. $^{13}C\{^{1}H\}$ NMR spectrum of compound **8** (110 – 180 ppm).

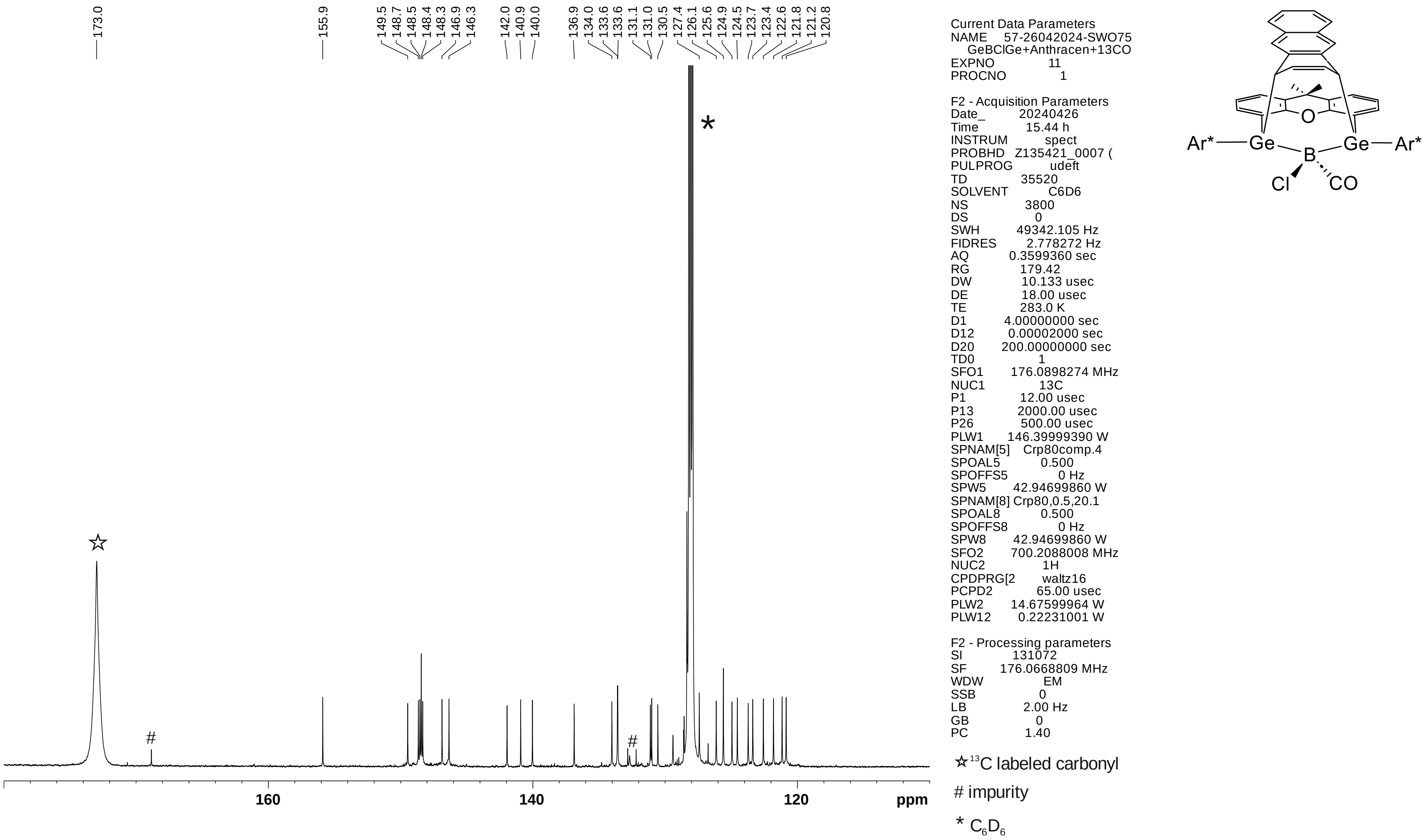


Figure S42. $^{13}C\{^{1}H\}$ NMR spectrum of compound **8**, after the reaction with $^{13}C$-labeled CO (110 – 180 ppm).

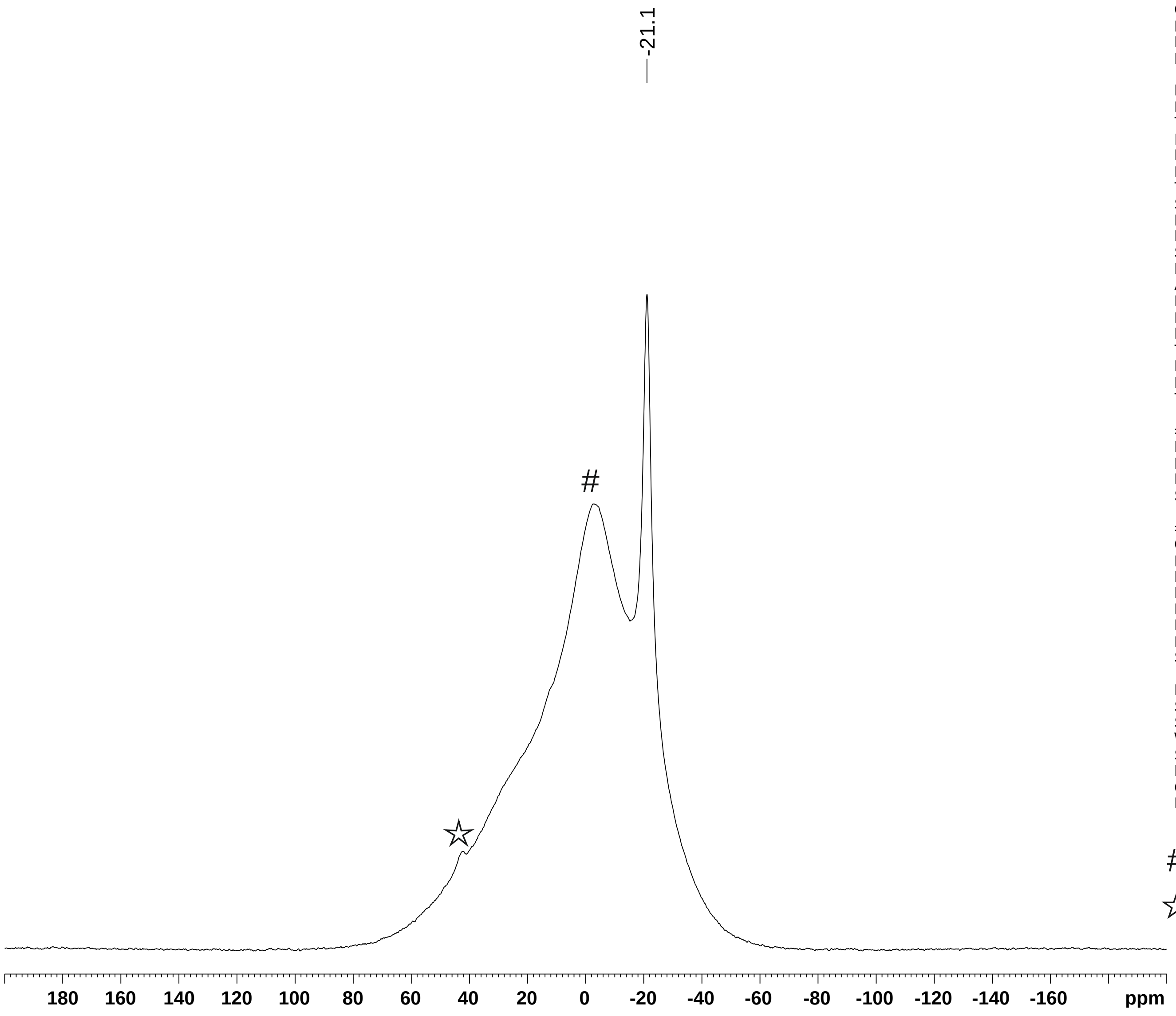


Current Data Parameters
NAME SW070
EXPNO 11
PROCNO 1

F2 - Acquisition Parameters
Date_ 20240418
Time 9.29
INSTRUM spect
PROBHD 5 mm X,F/H,F B
PULPROG zgig30
TD 8192
SOLVENT C6D6
NS 1024
DS 0
SWH 64102.563 Hz
FIDRES 7.825020 Hz
AQ 0.0638976 sec
RG 1440
DW 7.800 usec
DE 6.00 usec
TE 299.2 K
D1 0.10000000 sec
D11 0.03000000 sec
TD0 1

======== CHANNEL f1 ========
NUC1 11B
P1 15.00 usec
PL1 -3.00 dB
SFO1 128.3711880 MHz

======== CHANNEL f2 ========
CPDPRG[2 waltz16
NUC2 1H
PCPD2 90.00 usec
PL2 -1.50 dB
PL12 15.65 dB
PL2W 11.35402870 W
PL12W 0.21885175 W
SFO2 400.1108002 MHz

F2 - Processing parameters
SI 8192
SF 128.3711880 MHz
WDW EM
SSB 0
LB 50.00 Hz
GB 0
PC 1.40

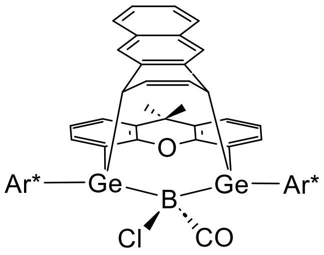


# glass
☆unreacted Boradigermaallyl

Figure S43. $^{11}B\{^{1}H\}$ NMR spectrum of compound **8**.

NMR spectra of compound **9**.

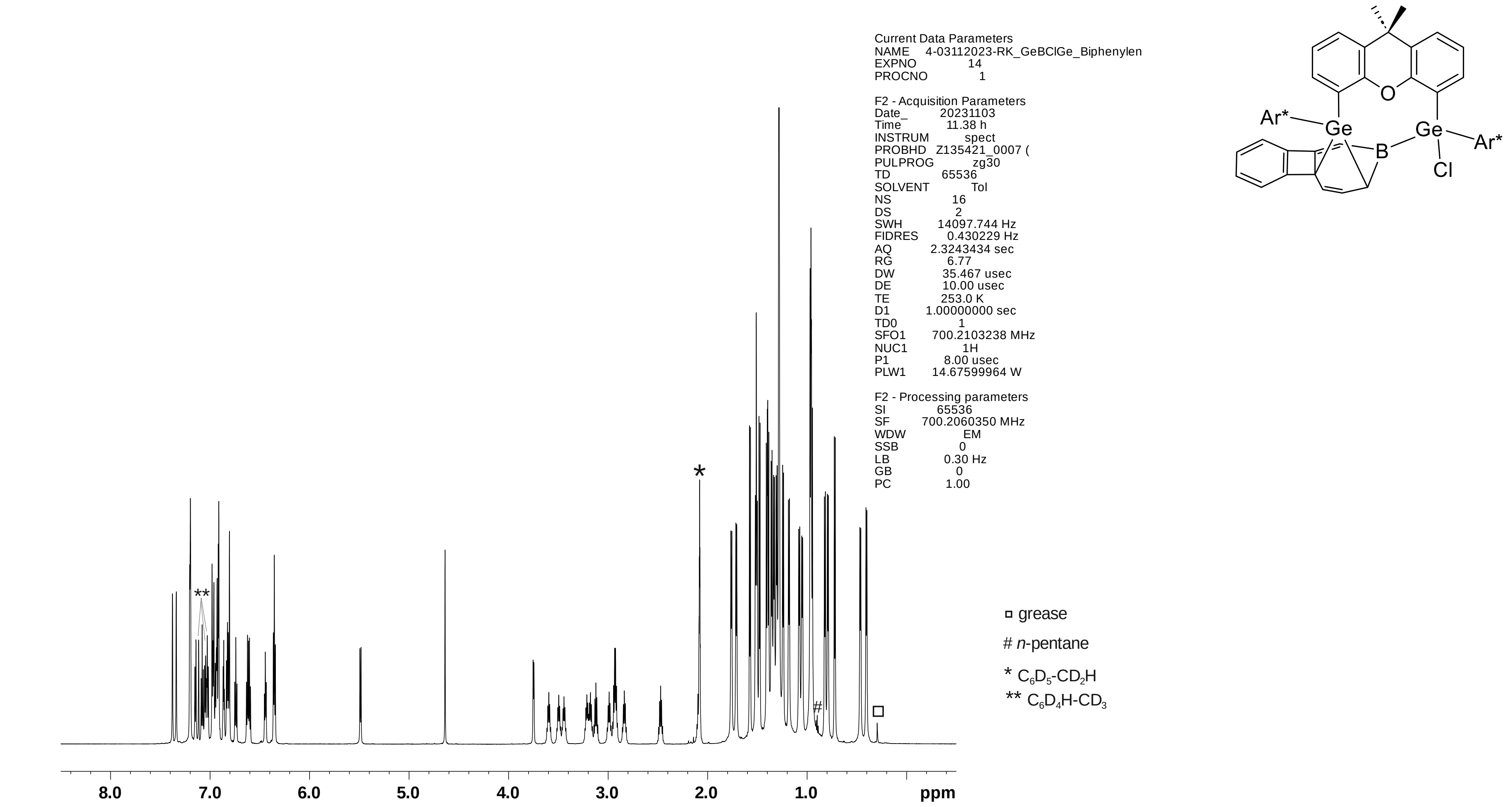


Figure S44. $^{1}$H NMR spectrum of compound **9**.

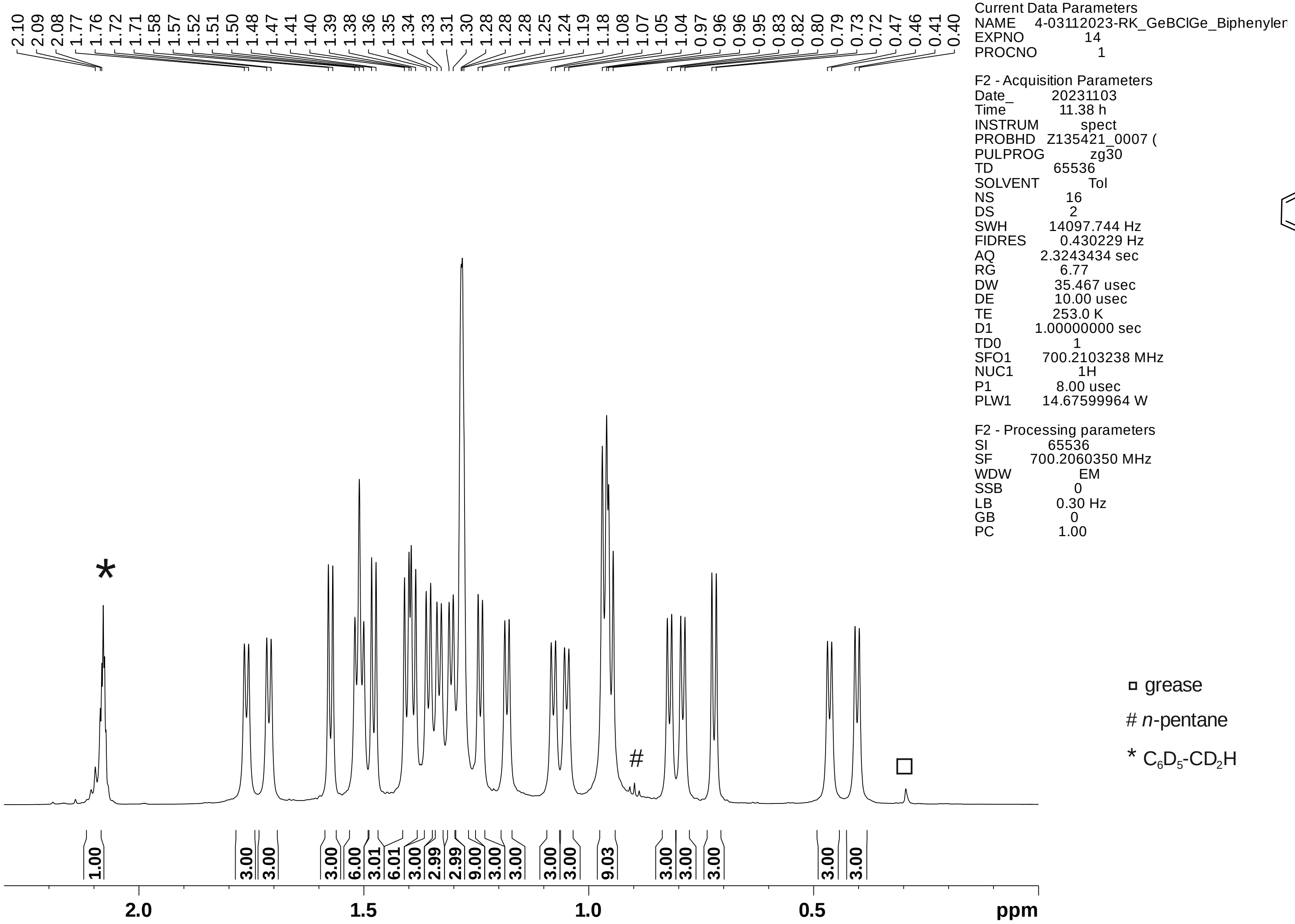

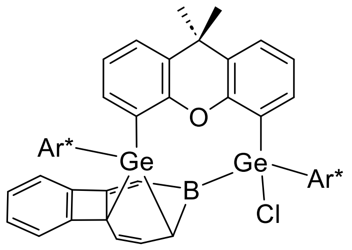

Figure S45. $^{1}$H NMR spectrum of compound **9** (0 – 2.3 ppm).

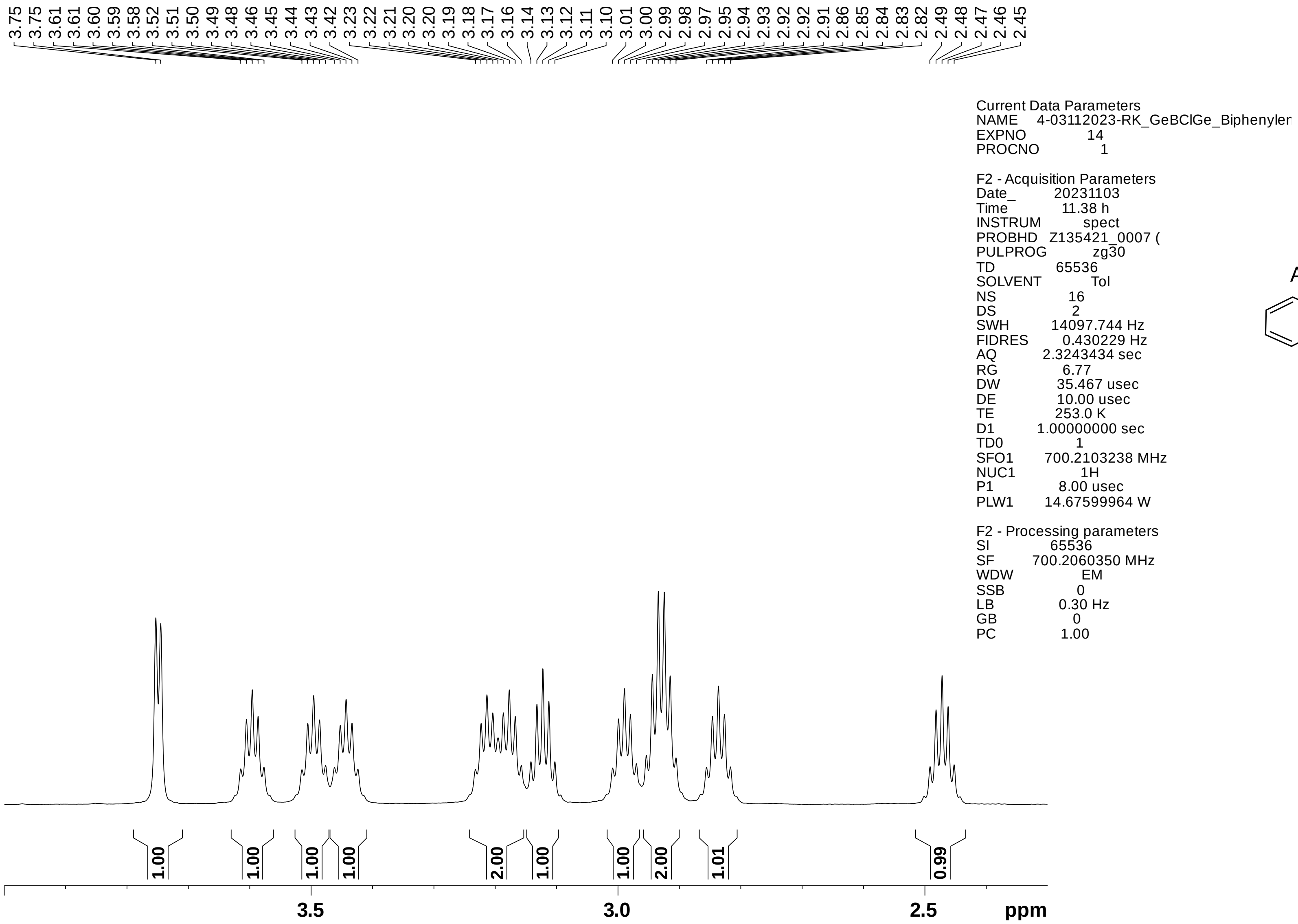


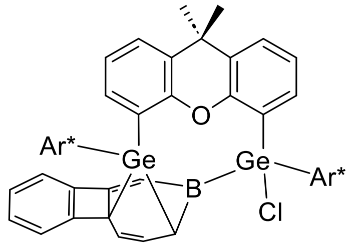


Figure S46. $^{1}$H NMR spectrum of compound **9** (2.3 – 4.0 ppm).

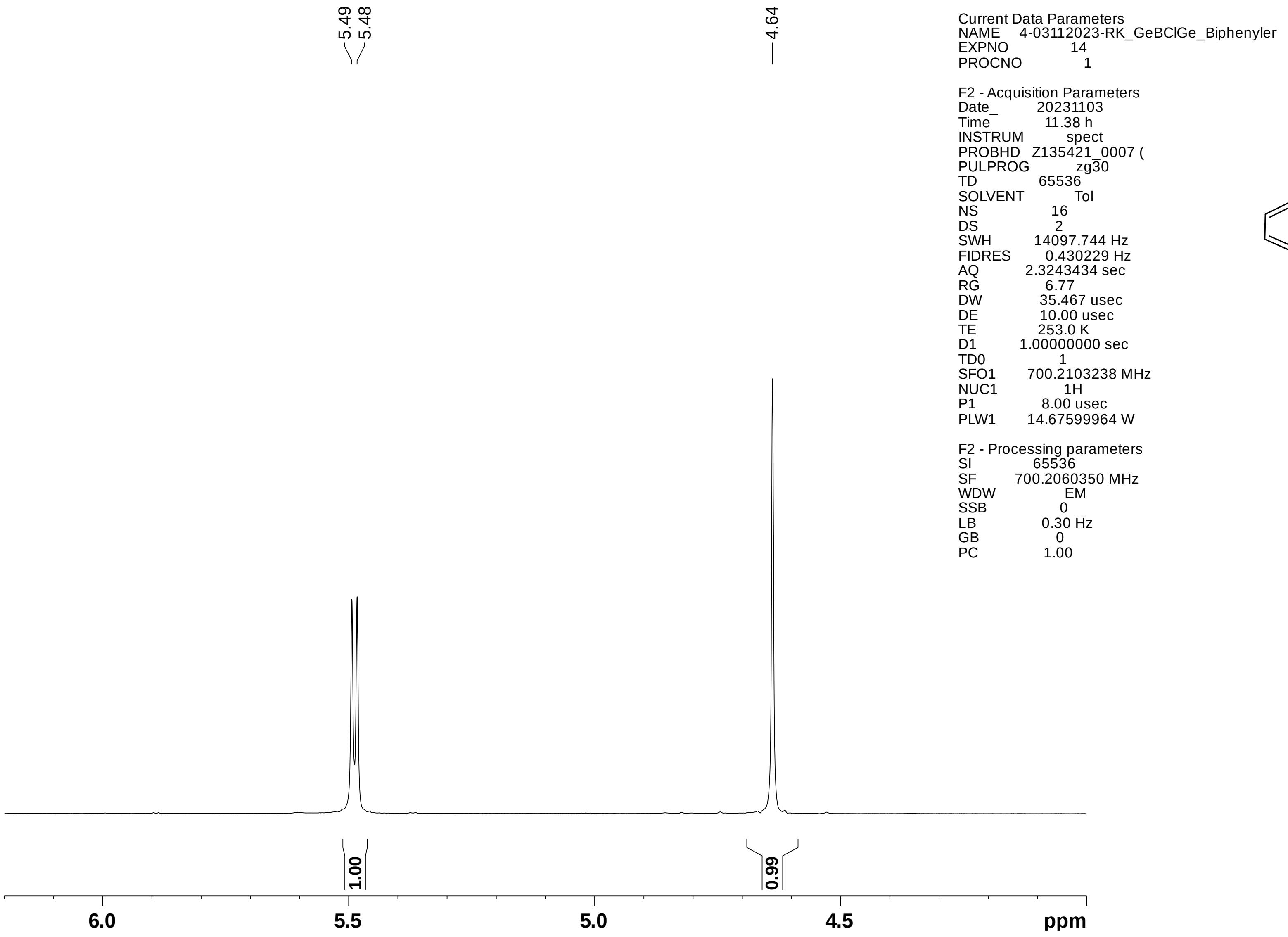


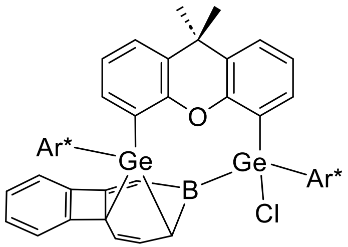


Figure S47. $^{1}H$ NMR spectrum of compound **9** (4.0 – 6.2 ppm).

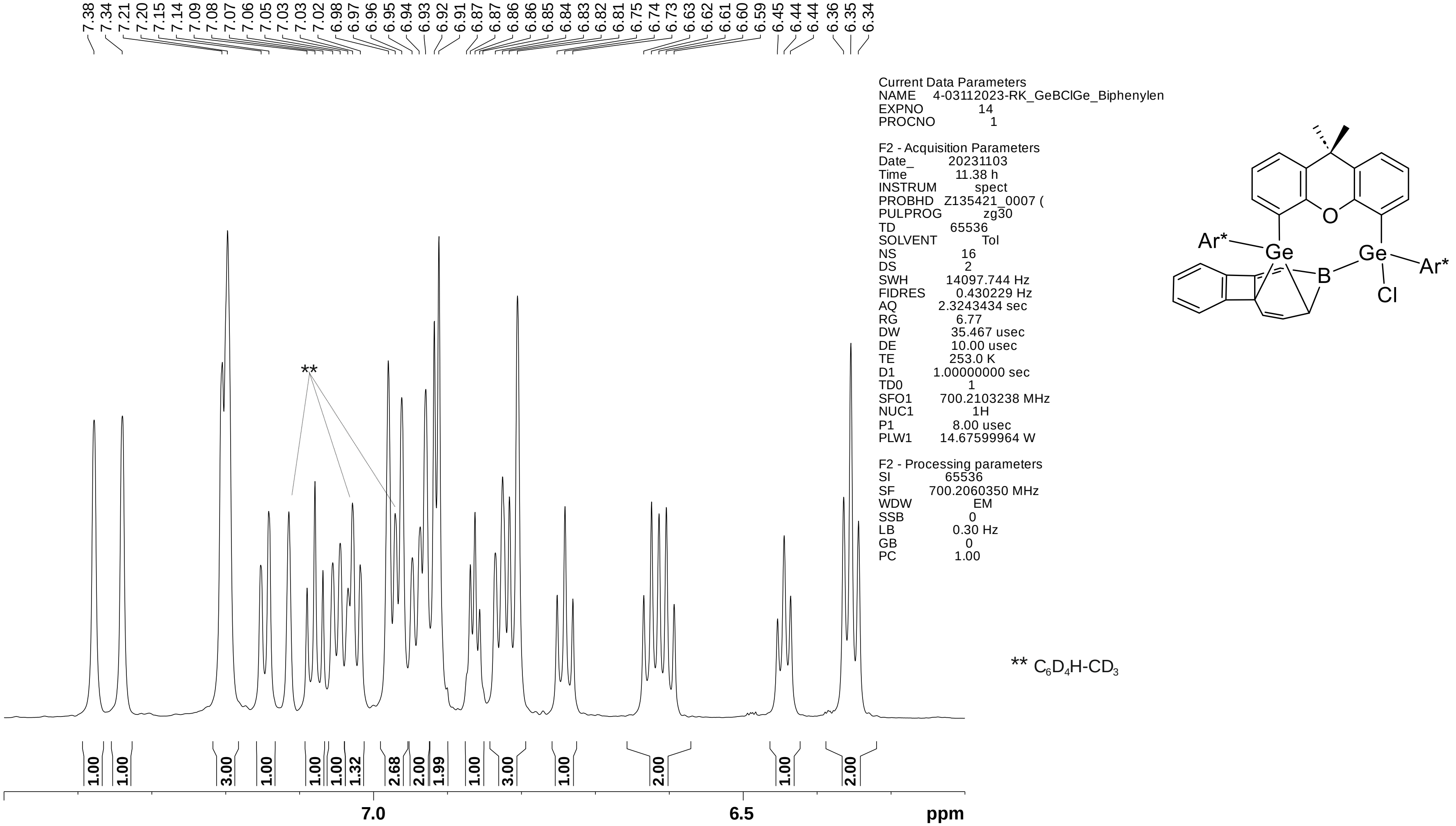


Figure S48. $^{1}$H NMR spectrum of compound **9** (6.2 – 7.5 ppm).

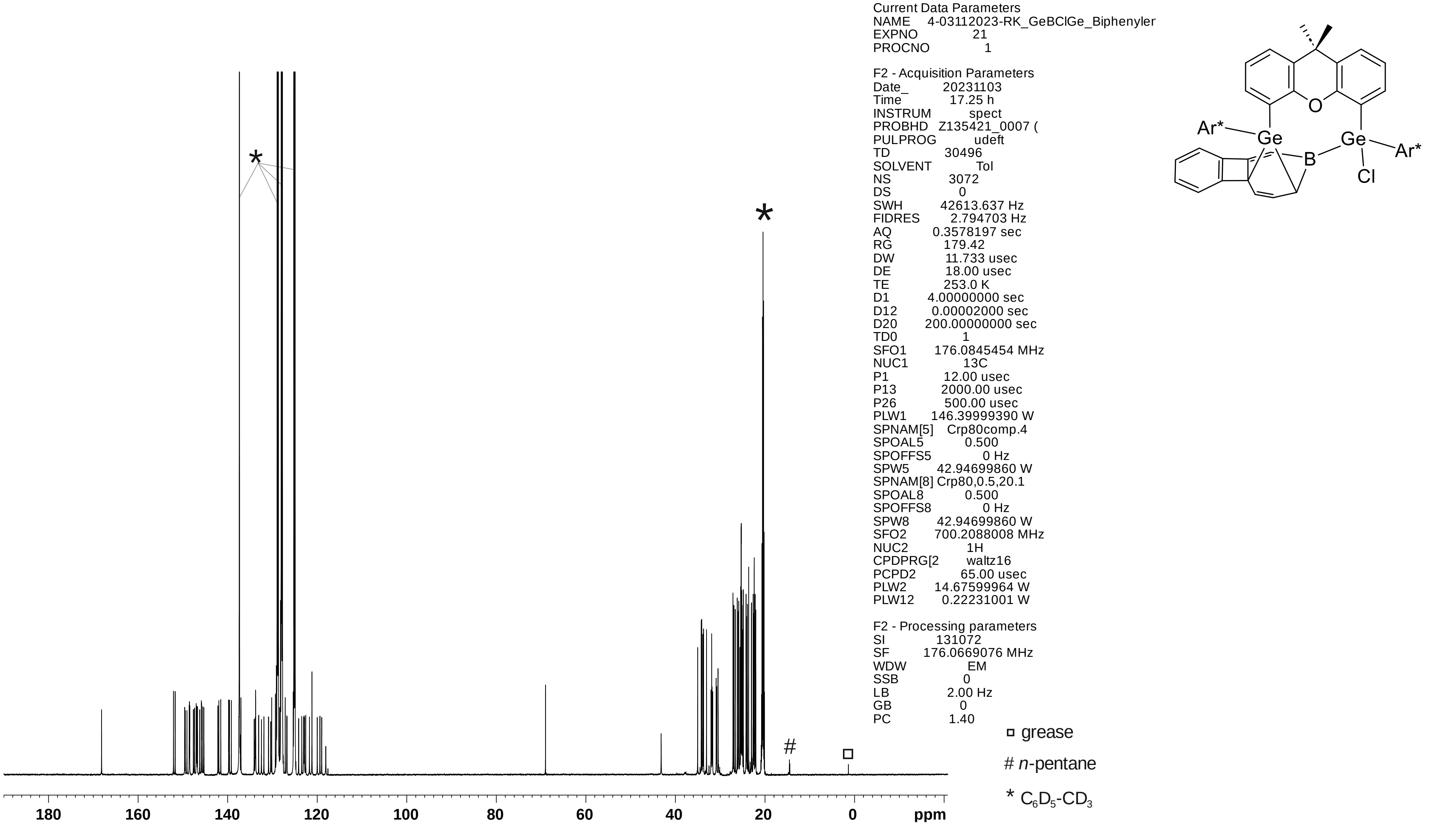


Figure S49. $^{13}C\{^{1}H\}$ NMR spectrum of compound **9**.

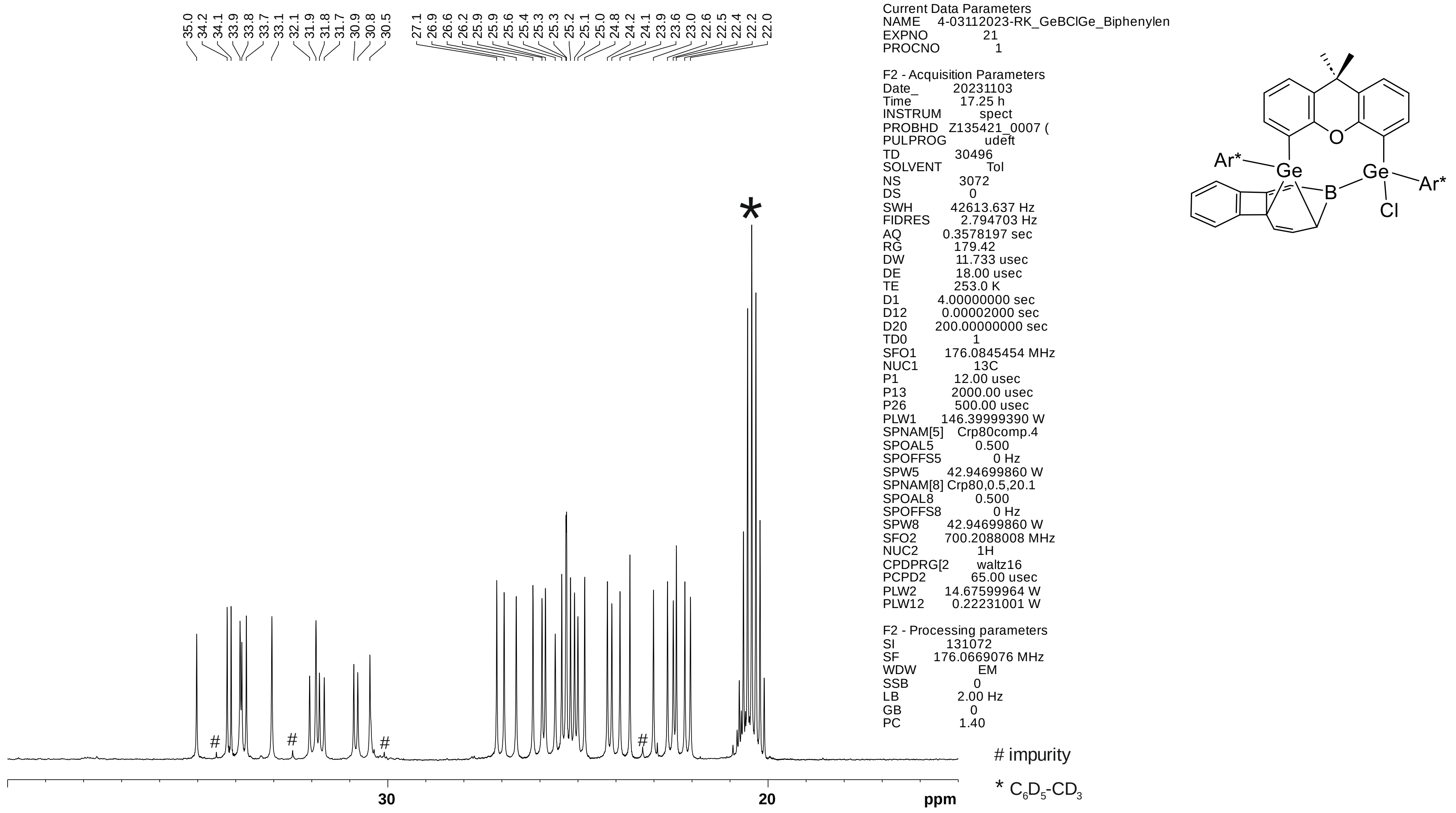


Figure S50. $^{13}C\{^{1}H\}$ NMR spectrum of compound **9** (15 – 40 ppm).

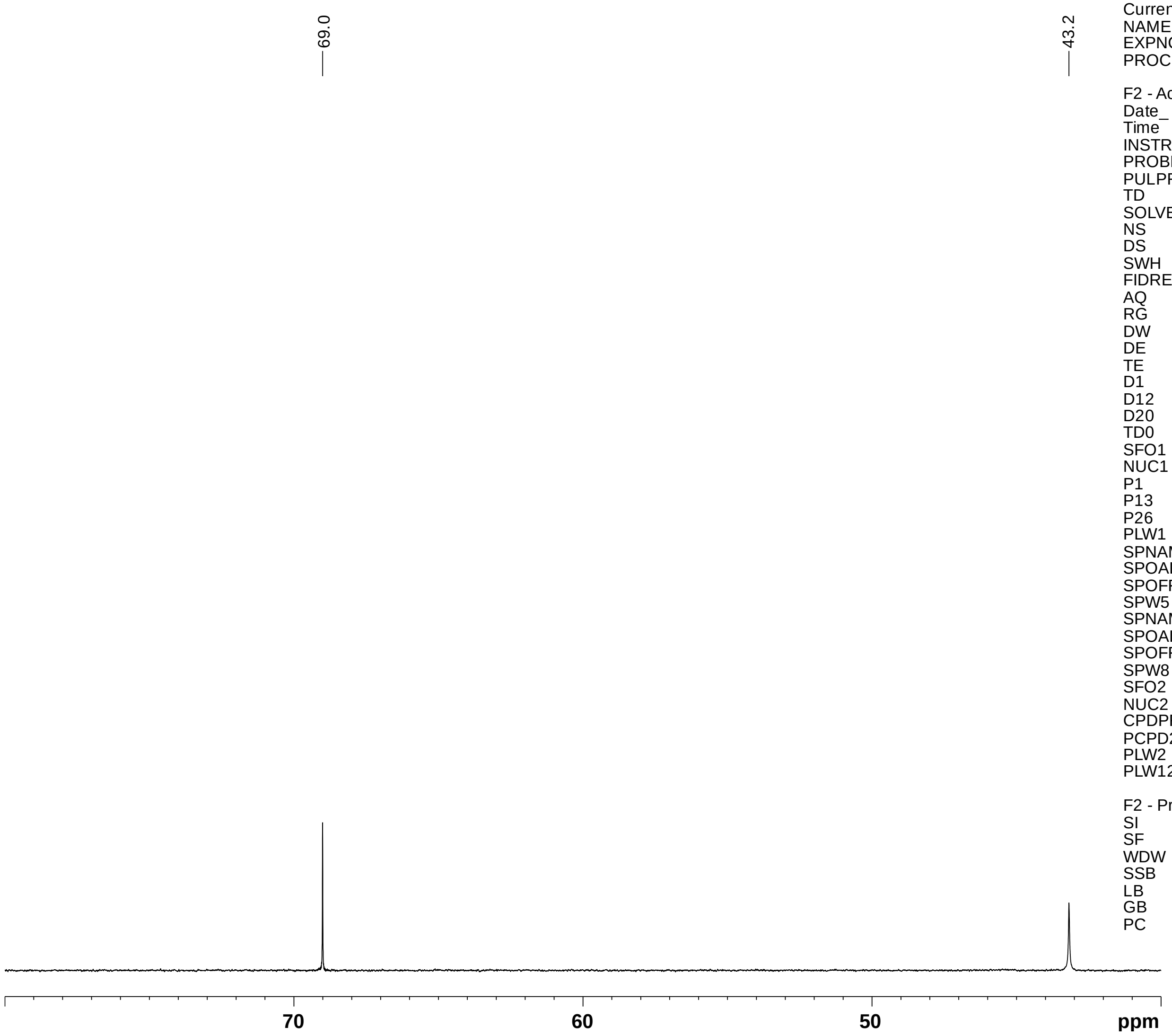


Current Data Parameters
NAME 4-03112023-RK_GeBClGe_Biphenyler
EXPNO 21
PROCNO 1

F2 - Acquisition Parameters
Date_ 20231103
Time 17.25 h
INSTRUM spect
PROBHD Z135421_0007 (
PULPROG udeft
TD 30496
SOLVENT Tol
NS 3072
DS 0
SWH 42613.637 Hz
FIDRES 2.794703 Hz
AQ 0.3578197 sec
RG 179.42
DW 11.733 usec
DE 18.00 usec
TE 253.0 K
D1 4.00000000 sec
D12 0.00002000 sec
D20 200.00000000 sec
TD0 1
SFO1 176.0845454 MHz
NUC1 13C
P1 12.00 usec
P13 2000.00 usec
P26 500.00 usec
PLW1 146.39999390 W
SPNAM[5] Crp80comp.4
SPOAL5 0.500
SPOFFS5 0 Hz
SPW5 42.94699860 W
SPNAM[8] Crp80,0.5,20.1
SPOAL8 0.500
SPOFFS8 0 Hz
SPW8 42.94699860 W
SFO2 700.2088008 MHz
NUC2 1H
CPDPRG[2 waltz16
PCPD2 65.00 usec
PLW2 14.67599964 W
PLW12 0.22231001 W

F2 - Processing parameters
SI 131072
SF 176.0669076 MHz
WDW EM
SSB 0
LB 2.00 Hz
GB 0
PC 1.40

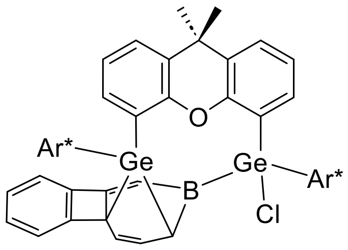


Figure S51. $^{13}C\{^{1}H\}$ NMR spectrum of compound **9** (40 – 80 ppm).

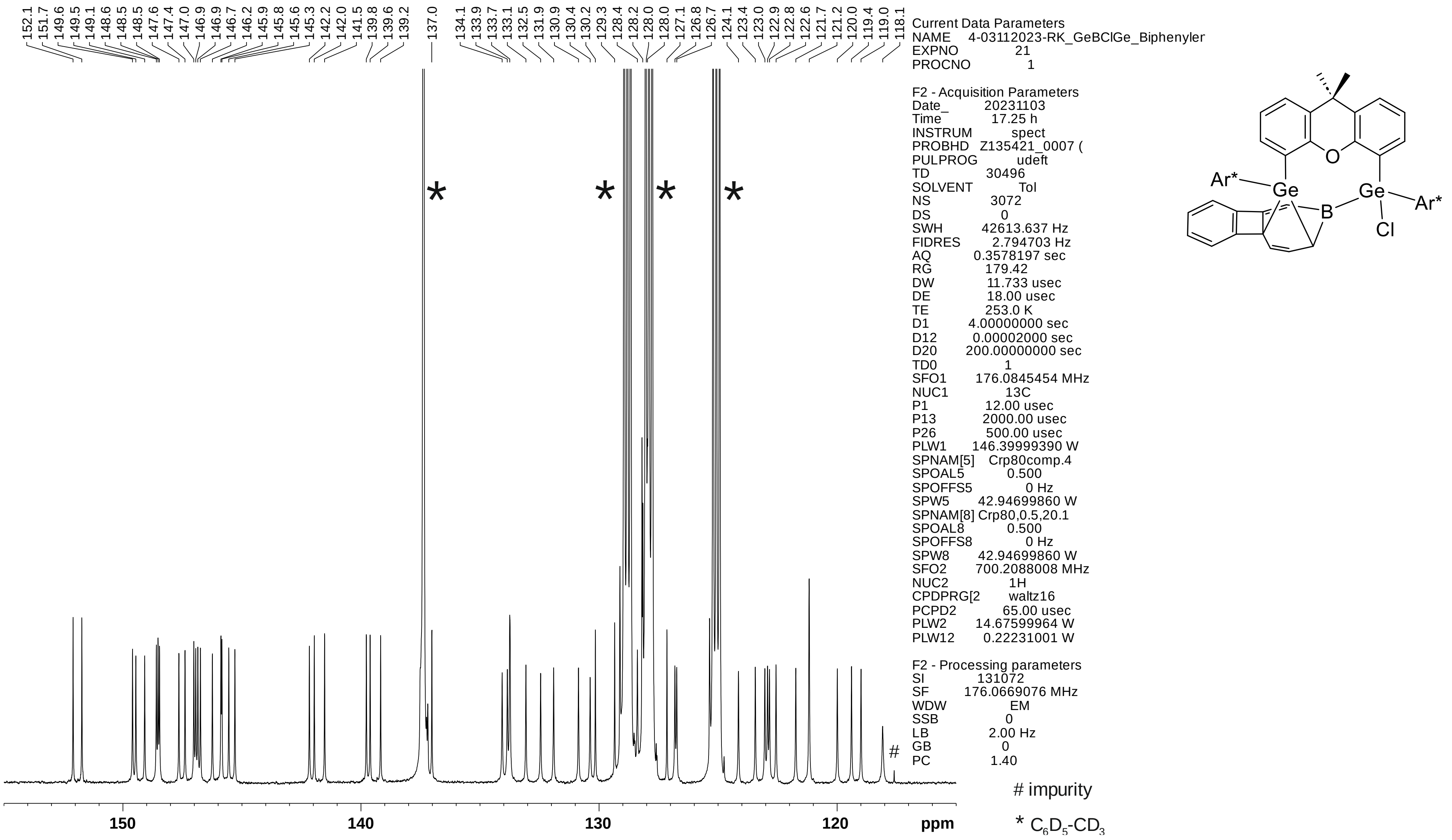


Figure S52. $^{13}C\{^{1}H\}$ NMR spectrum of compound **9** (115 – 155 ppm).

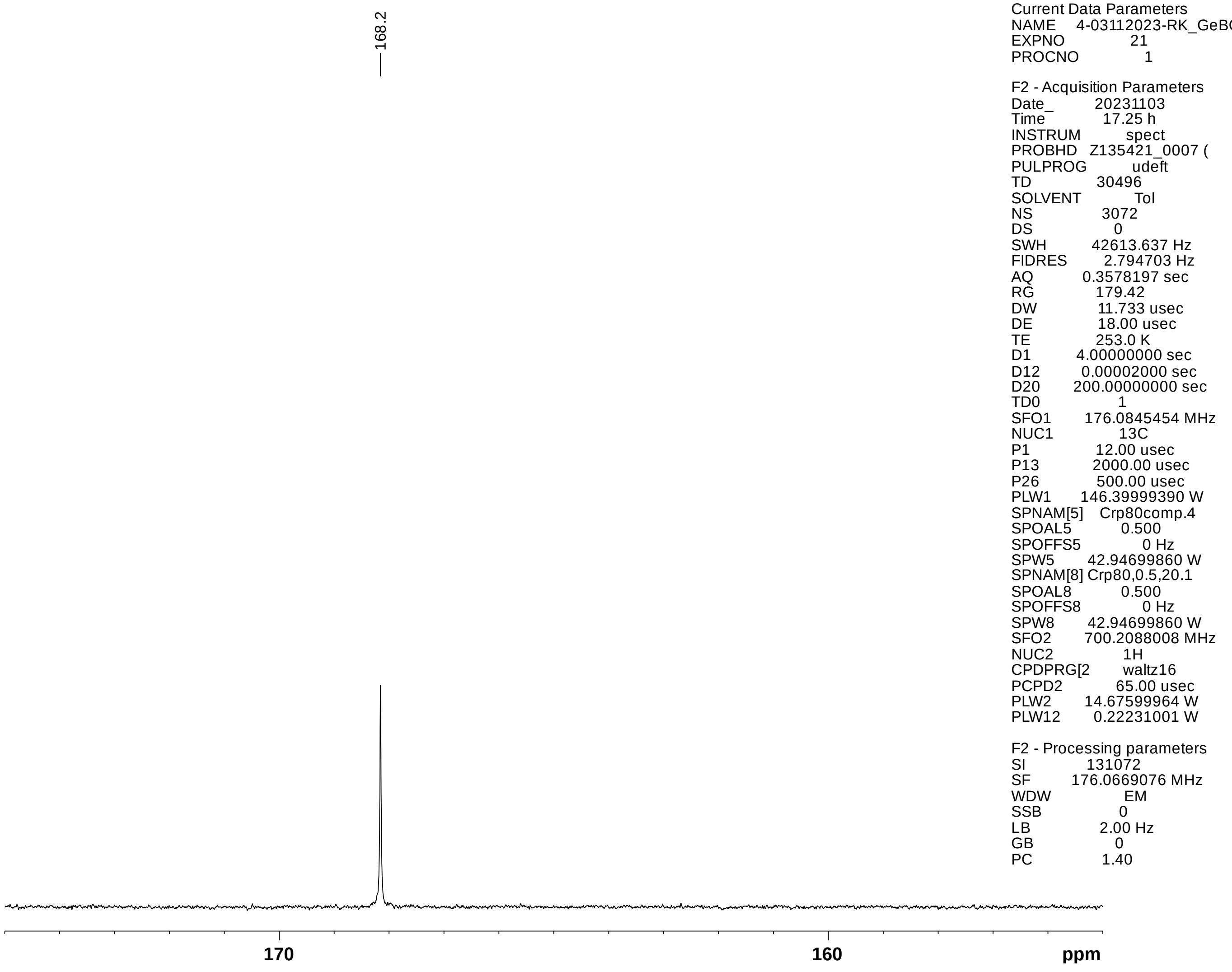


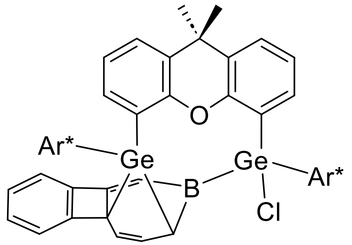


Figure S53. $^{13}C\{^{1}H\}$ NMR spectrum of compound **9** (155 – 175 ppm).

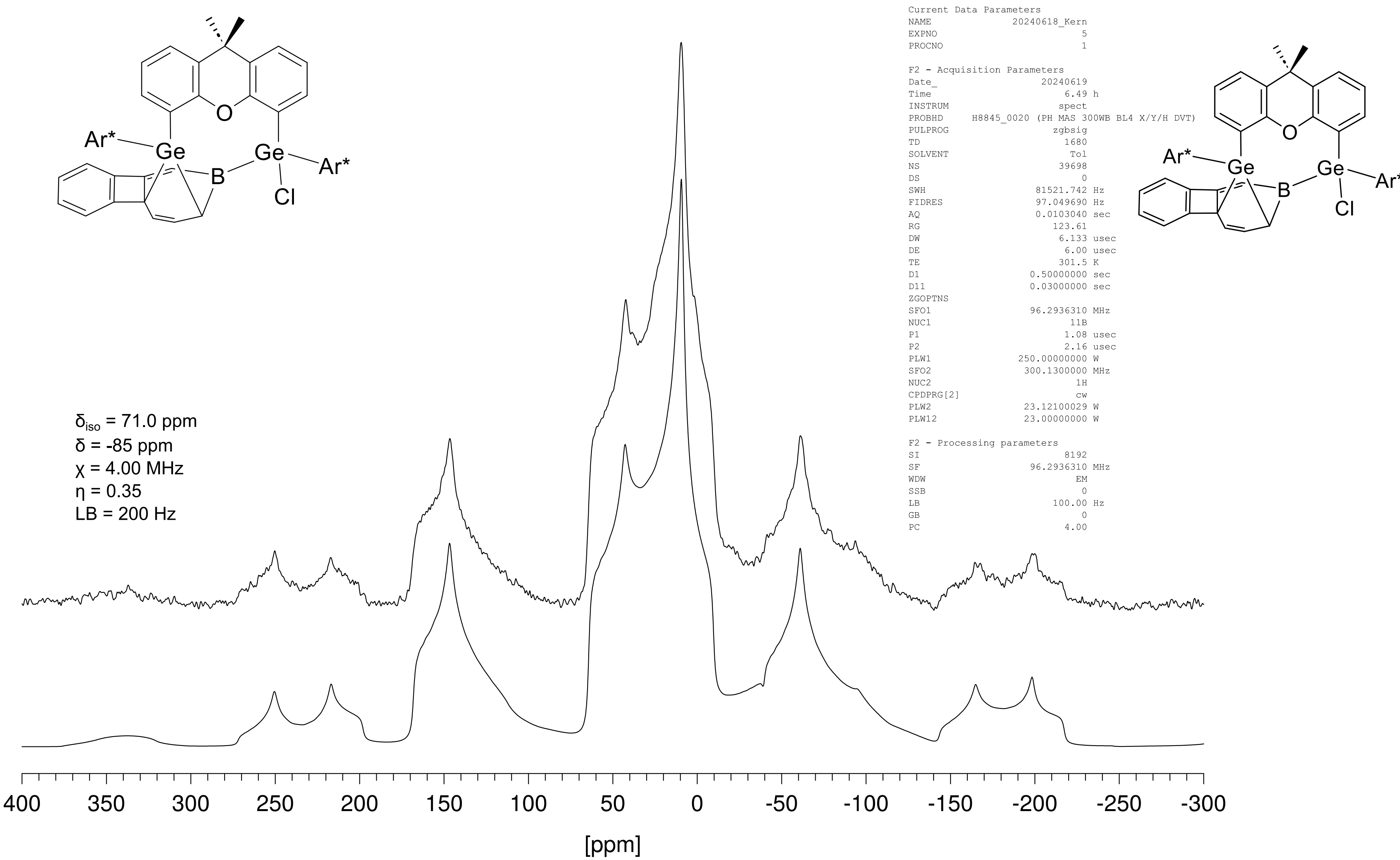


Figure S54. Experimental (top) and (bottom) calculated 96.29 MHz $^{11}$B MAS NMR spectra of the central transition of **9**, spinning at 10 kHz.

# IR-spectroscopy

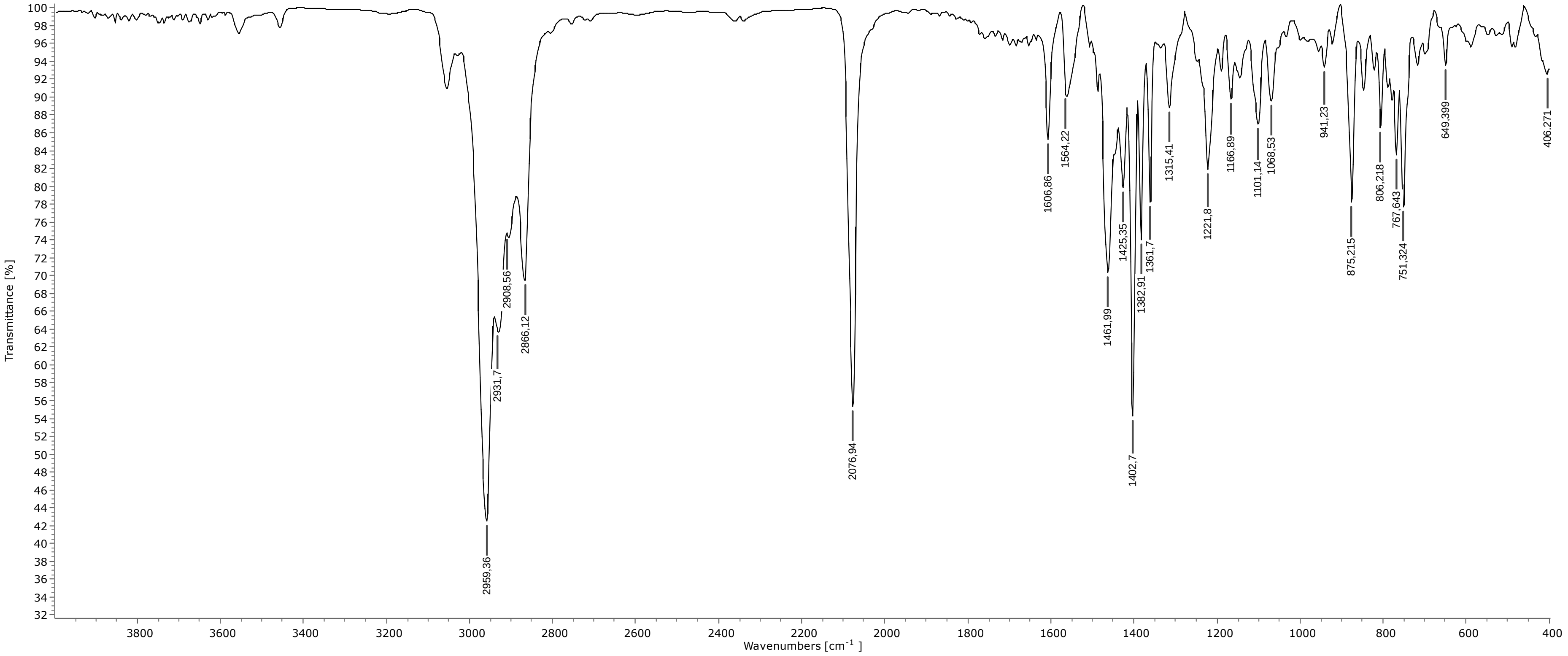


Figure S55. IR (KBr) spectrum of compound **6**.

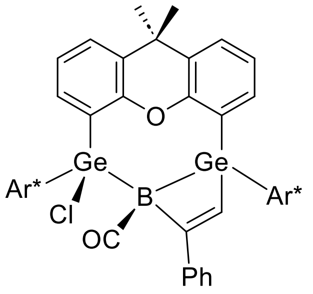

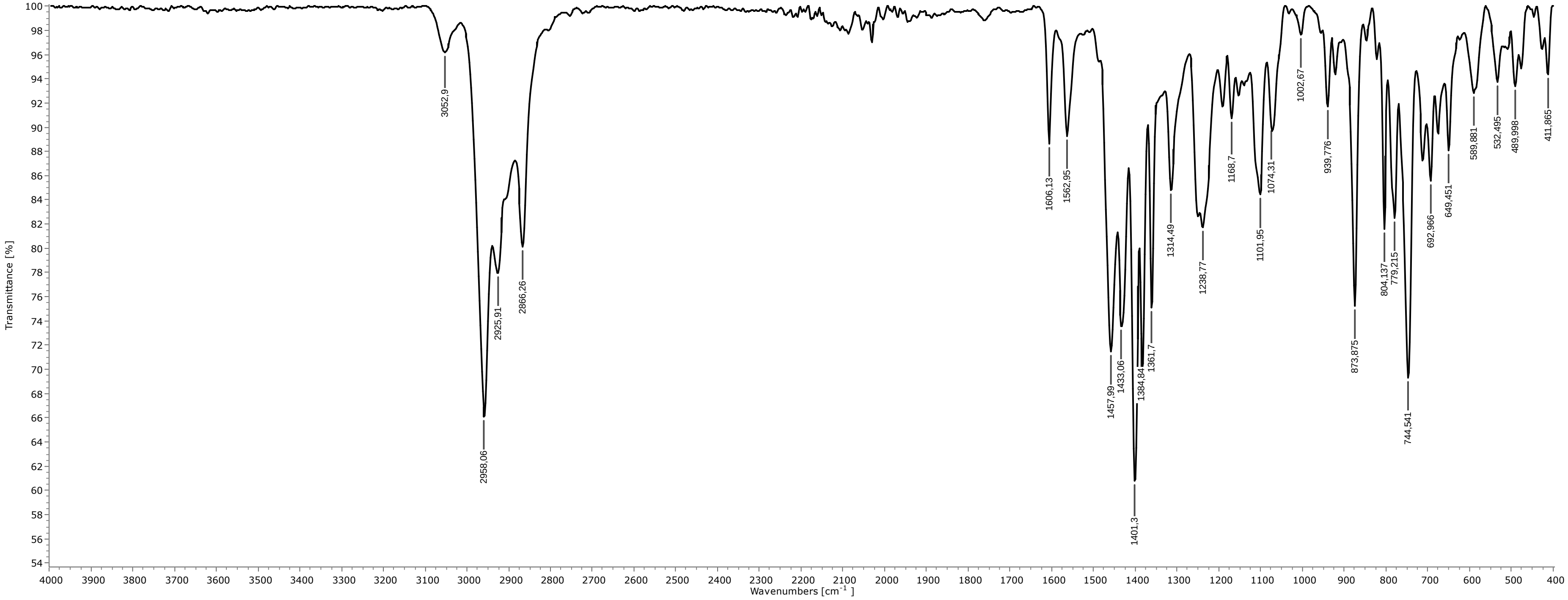


Figure S56. IR (ATR) spectrum of compound **7**.

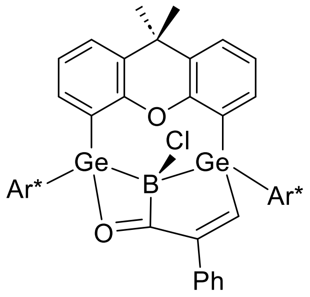

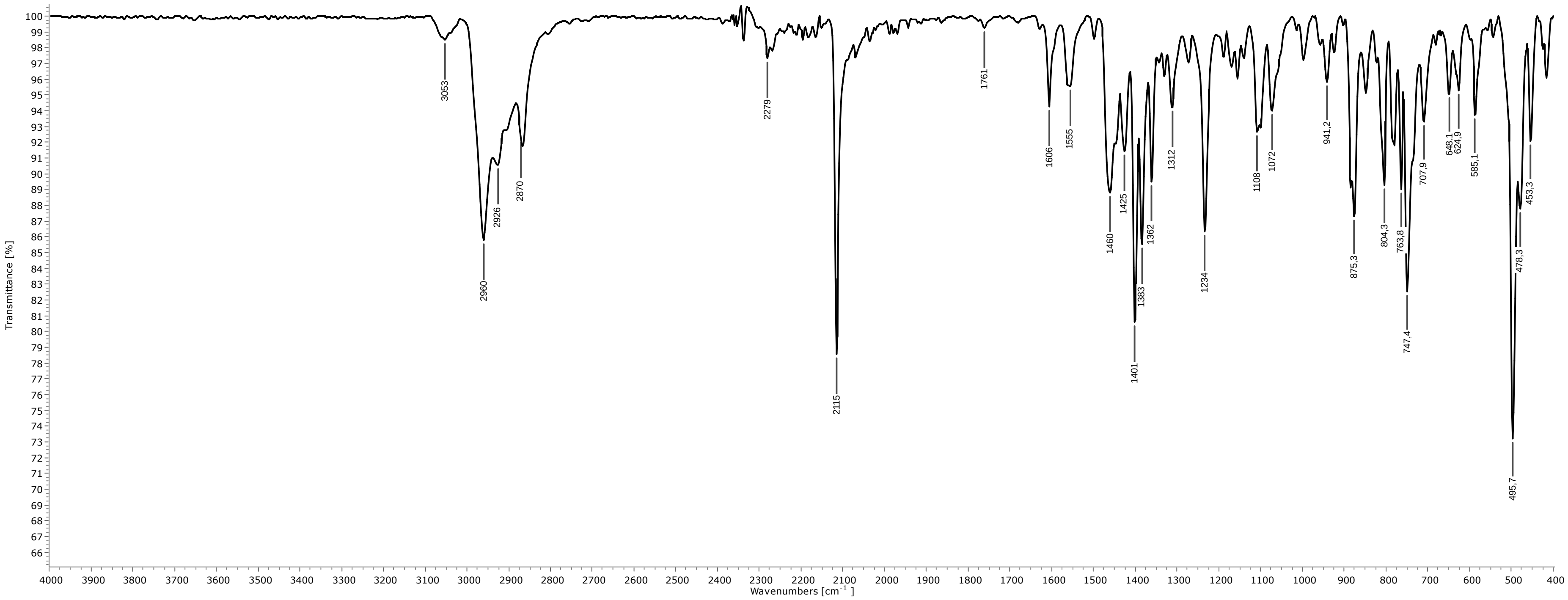


Figure S57. IR (ATR) spectrum of compound **8**.

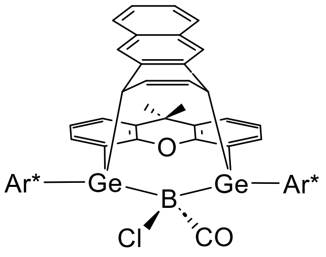

# UV-Vis spectroscopy

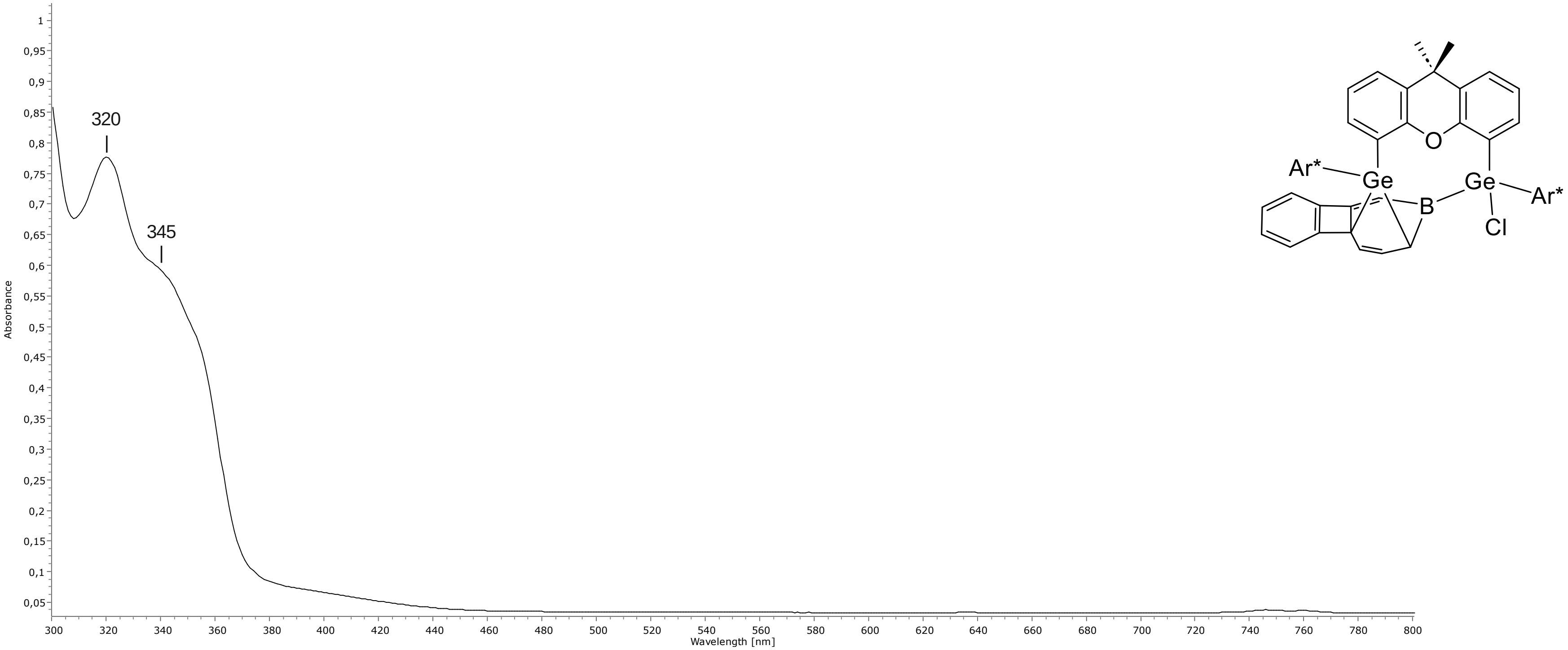


Figure S58. UV-Vis spectrum of compound **9.**

# Computational Methods

The computations of the relative energies of structural isomers **6** and **7** used the experimental X-ray single crystal structures as models and starting point for geometry optimizations. The obtained structures were subsequently used for structural optimization of smaller model systems by removing groups and adding hydrogen atoms as appropriate. All geometry optimizations were performed using the “Swiss army knife” method $r^2$-SCAN-3c[10] that employs the $r^2$SCAN[11, 12] meta-GGA functional along with a triple-ζ Gaussian basis set (def2-mTZVPP) and a fitting basis set for the resolution of the identity approximation,[10] the D4 correction for London dispersion,[13, 14] and a geometrical counterpoise correction for the basis set superposition error.[15] All geometry optimizations were followed by computations of harmonic vibrational frequencies to confirm the nature of the stationary points as minima. The Gibbs free energy correction obtained from harmonic vibrational frequencies computed at the $r^2$SCAN-3c level give the final Gibbs energies at T = 298.15 K reported in the manuscript.[16] In addition, the structures were also optimized under the consideration of cyclohexane solvent using the conductor-like polarizable continuum model (CPCM).[17]

Single point energy evaluations were conducted using a range of different computational methods: RI-MP2, DLPNO-CCSD(T),[18-20] for the smallest model system, the double hybrid functionals the revDSD-PBEP86-D4[21] and B2-PLYP,[22] the hybrid B3LYP[23-25] functional, the hybrid meta-GGA functional M06-2X,[26] the range-separated hybrid functional ωB97X,[27] and ωB97X-V,[28] a range separated hybrid with non-local correction. The B3LYP and ωB97X functionals were either used without any correction for London dispersion interactions, with the atom-pairwise correction including Becke-Johnson damping D3(BJ)[29] and charge dependent atom-pairwise correction D4.[13, 14] For B3LYP, the non-local density dependent dispersion correction (NL)[30] that uses the nonlocal part of the VV10 functional was also used.[31] The single points were also computed using the geometries obtained from the CPCM computations and employed in these cases also the CPMC model. All single point runs were conducted with tight SCF convergence criteria and with the def2-TZVP and appropriate fitting basis sets (def2-TZVP/C, def2/J and def2/JK) for the RI approximation.[32-35] For the DFT computations the RIJK approximation was used except for the ωB97X family of functionals that were run with the RIJCOSX approximation. No approximations were employed for the Hartree-Fock part of the RI-MP2 and DLPNO-CCSD(T) computations. All these computations were performed with ORCA 5.0.4.[36-38]

We draw the following conclusions from this data (Table S3):

1. With the smaller substituents (R = Ph, Me, H) **6** is more stable than **7** at all computational models. This suggests that the observation of the fast reaction of **6** to **7** is not reflecting inherent properties of the chemical cores of these isomers. Rather the reaction is driven by steric effects. It turns out that the description of these steric effects vs. London dispersion is quite challenging in these systems.

2. Methods that correct for London dispersion interactions using D3(BJ) or D4 incorrectly place the energy of **7** above that of **6**. A case in point is the data obtained for the hybrid functional B3LYP. Without any consideration of London dispersion B3LYP is qualitatively correct (**7** more stable than **6** by 7 kcal/mol), but this is reversed using D3(BJ) or D4. The NL correction performs somewhat better as both isomers are essentially isoenergetic.

3. MP2 is known to overestimate London dispersion interactions, and this method indeed favors **6** most strongly. The double hybrid functionals that include MP2 correlation to some extent, reduce the energy difference between isomers. B2PLYP has **7** lower in energy than **6**, but explicit inclusion of London dispersion using D3(BJ) overcorrects. Also, revDSD-PBEP86-D4 overbinds **6**.

4. The composite DFT method using the meta-GGA functional $r^2$SCAN ($r^2$SCAN-3c) that is including D4 correction and the hybrid meta-GGA functional M06-2X that takes London dispersion into account by its construction also incorrectly favor **6** over **7**.

5. The range-separated hybrid functional ωB97X appears qualitatively correct, but with D3(BJ) correction both isomers become isoenergetic. The ωB97X-V, a range separated hybrid with non-local correction, seems to be able to provide a balanced description of steric strain and attractive London dispersion interactions.

Table S3. Computed relative electronic energies ΔE and relative Gibbs free energies ΔG (all in kcal/mol) of isomer **7** relative to that of isomer **6** depending on the substituent R bound to the Ge atoms (Ar* = $C_6H_3$-2,6-(Trip)$_2$ (Trip = 2,4,6-$C_6H_2$*i*$Pr_3$); terphenyl = [1,1':3',1''-terphenyl]-2'-yl). Geometries were optimized with the r$^2$SCAN-3c method, all other data are single point energies with the def2-TZVP basis set.

| method | Ar* | | terphenyl | | $C_6H_5$ | | $CH_3$ | | H | |
|---|---|---|---|---|---|---|---|---|---|---|
| | ΔE | ΔG | ΔE | ΔG | ΔE | ΔG | ΔE | ΔG | ΔE | ΔG |
| r$^2$SCAN-3c | 0.2 | 0.2 | -0.4 | 1.3 | 9.8 | 11.5 | 8.8 | 9.8 | 8.1 | 9.7 |
| DLPNO-CCSD(T) | | | | | | | | | 7.3 | 8.9 |
| M06-2X | 2.1 | 2.1 | 0.9 | 2.7 | 9.7 | 11.4 | 8.8 | 9.8 | 8.7 | 10.3 |
| B3LYP | -7.6 | -7.6 | 3.3 | 5.1 | 6.1 | 7.8 | 7.1 | 8 | 8.3 | 9.9 |
| B3LYP-D3(BJ) | 3.3 | 3.2 | 1.5 | 3.2 | 11.6 | 13.3 | 9.8 | 10.8 | 9.1 | 10.6 |
| B3LYP-D4 | 1.4 | 1.6 | -0.2 | 1.5 | 10.3 | 12 | 8.3 | 9.2 | 7.6 | 9.2 |
| B3LYP-NL | 0.1 | 0 | -0.9 | 0.9 | 8.0 | 9.7 | 6.5 | 7.5 | 6.0 | 7.6 |
| RI-MP2 | 11.9 | 11.8 | 3.8 | 5.6 | 15.8 | 17.5 | 12.3 | 13.3 | 10.7 | 12.3 |
| B2-PLYP | -1.1 | -1.2 | 2.9 | 4.7 | 8.8 | 10.5 | 8.3 | 9.3 | 8.6 | 10.1 |
| B2-PLYP-D3(BJ) | 4.5 | 4.4 | 2.0 | 3.8 | 11.8 | 13.5 | 9.8 | 10.8 | 9.0 | 10.6 |
| revDSD-PBEP86-D4 | 2.1 | 2.0 | 1.0 | 1 | 9.4 | 11.1 | 7.9 | 8.9 | 7.6 | 9.2 |
| ωB97X | -4.3 | -4.4 | -1.4 | 0.3 | 5.4 | 7.1 | 4.5 | 5.4 | 5.3 | 6.8 |
| ωB97X-D3(BJ) | 0.3 | 0.2 | -1.5 | 0.3 | 7.8 | 9.5 | 5.9 | 6.8 | 5.8 | 7.4 |
| ωB97X-V | -1.7 | -1.8 | -3.3 | -1.5 | 5.5 | 7.2 | 3.4 | 4.3 | 3.3 | 4.9 |
| | CPCM(cyclohexane) | | | | | | | | | |
| r$^2$SCAN-3c | 0.5 | 0.7 | 0.7 | 2.5 | 9.5 | 10.9 | 8.3 | 9.3 | 7.7 | 9.3 |
| DLPNO-CCSD(T) | | | | | | | | | 6.9 | 8.4 |
| M06-2X | 2.4 | 2.6 | 2.1 | 3.8 | 9.3 | 10.7 | 8.4 | 9.3 | 8.3 | 9.8 |
| B3LYP | -7.0 | -6.8 | 3.8 | 5.6 | 6.2 | 7.6 | 6.8 | 7.8 | 8.0 | 9.6 |
| B3LYP-D3(BJ) | 3.4 | 3.6 | 2.3 | 4.1 | 11.3 | 12.7 | 9.4 | 10.4 | 8.7 | 10.3 |
| B3LYP-D4 | 1.7 | 1.8 | 0.6 | 2.4 | 10.0 | 11.4 | 7.9 | 8.9 | 7.3 | 8.8 |
| B3LYP-NL | 0.3 | 0.5 | 0.0 | 1.8 | 7.8 | 9.1 | 6.1 | 7.1 | 5.7 | 7.2 |
| RI-MP2 | 11.7 | 11.9 | 4.9 | 6.7 | 15.2 | 16.7 | 11.8 | 12.8 | 10.3 | 11.8 |
| B2-PLYP | -0.8 | -0.7 | 3.7 | 5.5 | 8.6 | 10.0 | 7.9 | 8.9 | 8.2 | 9.8 |
| B2-PLYP-D3(BJ) | 4.6 | 4.8 | 2.9 | 4.7 | 11.4 | 12.8 | 9.4 | 10.4 | 8.6 | 10.2 |
| revDSD-PBEP86-D4 | 2.2 | 2.3 | 1.8 | 3.6 | 9.0 | 10.4 | 7.4 | 8.4 | 7.2 | 8.7 |
| ωB97X | -4.1 | -4.0 | -0.8 | 1.0 | 5.1 | 6.5 | 4.0 | 5.0 | 4.8 | 6.4 |
| ωB97X-D3(BJ) | 0.3 | 0.5 | -0.7 | 1.1 | 7.4 | 8.8 | 5.4 | 6.4 | 5.3 | 1.6 |
| ωB97X-V | -1.7 | -1.5 | -2.4 | -0.7 | 5.1 | 6.5 | 2.8 | 3.8 | 2.8 | 4.4 |

## Cartesian Coordinates

All structures were computed using r²-SCAN-3c without or with CPCM(cyclohexane) as implemented in Orca 5.0.4 and are given in Å.
In addition to **6** and **7** variants with smaller substituents on the Ge atoms were studied computationally. They are named as **6-X and 7-X**, where X denotes the substituent on the two Ge atoms (-H, -Me, -Ph, and -Terph for -terphenyl).

A. r²-SCAN-3c
Coordinates of compound **6**

| | | | |
|---|---|---|---|
| Ge | 4.568837000 | 4.286617000 | 4.700326000 |
| Ge | 7.538984000 | 4.502740000 | 7.153271000 |
| Cl | 7.597201000 | 6.727029000 | 7.113596000 |
| O | 4.677253000 | 3.375122000 | 7.583052000 |
| O | 7.543513000 | 6.197291000 | 3.802259000 |
| C | 11.675043000 | 3.363935000 | 6.728779000 |
| H | 12.561856000 | 3.766819000 | 6.247210000 |
| C | 3.373864000 | 3.748594000 | 7.314510000 |
| C | 2.400425000 | 7.319402000 | 2.661890000 |
| H | 2.232735000 | 8.388092000 | 2.763155000 |
| C | 0.822830000 | 4.558834000 | 6.731216000 |
| H | -0.188533000 | 4.879337000 | 6.498506000 |
| C | 5.252988000 | 2.555888000 | 4.163395000 |
| H | 4.785706000 | 1.689617000 | 3.699784000 |
| C | 3.112336000 | 4.197240000 | 6.028725000 |
| C | 5.249511000 | 9.121898000 | 5.753607000 |
| H | 5.179104000 | 9.441245000 | 6.790331000 |
| C | 11.704331000 | 2.101413000 | 7.295578000 |
| H | 12.598193000 | 1.486921000 | 7.230645000 |
| C | 2.430068000 | 3.735030000 | 8.342625000 |
| C | 1.889309000 | 5.222894000 | 1.624116000 |
| H | 1.326981000 | 4.636517000 | 0.901596000 |
| C | 1.582818000 | 6.558973000 | 1.836964000 |
| H | 0.747333000 | 7.019766000 | 1.317472000 |
| C | 10.905807000 | 7.905578000 | 6.420435000 |
| H | 10.950318000 | 8.784024000 | 7.058306000 |
| C | 1.803419000 | 4.598216000 | 5.746408000 |
| H | 1.557246000 | 4.982119000 | 4.759989000 |
| C | 6.194836000 | 9.710384000 | 4.919833000 |
| C | 6.240619000 | 9.298431000 | 3.592260000 |
| H | 6.950611000 | 9.778002000 | 2.922596000 |
| C | 5.394613000 | 8.310641000 | 3.088643000 |
| C | 7.610030000 | 1.736047000 | 4.042828000 |
| C | 2.930605000 | 4.606729000 | 2.326435000 |
| C | 3.885543000 | 0.575534000 | 1.104492000 |
| C | 7.112873000 | 10.805366000 | 5.425107000 |
| H | 7.790554000 | 11.066631000 | 4.599991000 |
| C | 10.522761000 | 4.157845000 | 6.764851000 |
| C | 6.450378000 | 4.357433000 | 8.787696000 |
| C | 4.455481000 | 7.710836000 | 3.958560000 |
| C | 1.137850000 | 4.145579000 | 8.024431000 |
| H | 0.368283000 | 4.160961000 | 8.789428000 |
| C | 3.681871000 | 5.350807000 | 3.266018000 |
| C | 10.739904000 | 6.649679000 | 6.994613000 |
| C | 11.072241000 | 4.498502000 | 3.808492000 |

| | | | |
|---|---|---|---|
| H | 10.936952000 | 3.569676000 | 4.365665000 |
| C | 3.478239000 | 6.747497000 | 3.345821000 |
| C | 5.155795000 | 3.867128000 | 8.784078000 |
| C | 5.767229000 | 0.247812000 | 6.886682000 |
| H | 5.445262000 | -0.674131000 | 7.384256000 |
| H | 5.716301000 | 0.066980000 | 5.807787000 |
| H | 5.063695000 | 1.048371000 | 7.125213000 |
| C | 4.367027000 | 8.146624000 | 5.293204000 |
| C | 6.564802000 | 0.512948000 | 9.706157000 |
| H | 5.750842000 | -0.150276000 | 9.426365000 |
| C | 4.718636000 | 1.646982000 | 0.790810000 |
| H | 5.620407000 | 1.451913000 | 0.216712000 |
| C | 9.357560000 | 3.681012000 | 7.421558000 |
| C | 10.660324000 | 5.516409000 | 6.149820000 |
| C | 6.316432000 | 12.064153000 | 5.795374000 |
| H | 5.710321000 | 12.409694000 | 4.951668000 |
| H | 6.990539000 | 12.874958000 | 6.094270000 |
| H | 5.640831000 | 11.862755000 | 6.634409000 |
| C | 4.298829000 | 3.915779000 | 9.887067000 |
| C | 4.781446000 | 4.530215000 | 11.038049000 |
| H | 4.148325000 | 4.621978000 | 11.914705000 |
| C | 7.309744000 | 0.672784000 | 3.172654000 |
| H | 6.308119000 | 0.581499000 | 2.764591000 |
| C | 7.390696000 | 1.039627000 | 8.712294000 |
| C | 10.594685000 | 1.659395000 | 7.994678000 |
| H | 10.615910000 | 0.701324000 | 8.505113000 |
| C | 12.104101000 | 6.043976000 | 9.013735000 |
| H | 12.865871000 | 6.791544000 | 8.763577000 |
| H | 12.399986000 | 5.091403000 | 8.565964000 |
| H | 12.090359000 | 5.920170000 | 10.103246000 |
| C | 10.860674000 | 5.668520000 | 4.761352000 |
| C | 3.267380000 | 3.194375000 | 1.953852000 |
| C | 2.738821000 | 0.836494000 | 1.845966000 |
| H | 2.075476000 | 0.016074000 | 2.109591000 |
| C | 6.073929000 | 5.050672000 | 11.074860000 |
| H | 6.428135000 | 5.541358000 | 11.976716000 |
| C | 5.327442000 | 1.211584000 | 13.108463000 |
| H | 6.132909000 | 1.605639000 | 13.737830000 |
| H | 4.584258000 | 0.755295000 | 13.771818000 |
| H | 4.866220000 | 2.060180000 | 12.593016000 |
| C | 6.752981000 | 0.801228000 | 11.053742000 |
| C | 6.571205000 | 2.706960000 | 4.433011000 |
| C | 7.813599000 | 1.630322000 | 11.406728000 |
| H | 7.987949000 | 1.851268000 | 12.456014000 |
| C | 2.408133000 | 2.125776000 | 2.268096000 |
| C | 5.382833000 | 4.076682000 | 0.807026000 |
| H | 5.369180000 | 4.816801000 | 1.615427000 |
| C | 3.261676000 | 7.660826000 | 6.206700000 |
| H | 2.797842000 | 6.793315000 | 5.731916000 |
| C | 10.982432000 | 6.955205000 | 4.234368000 |
| H | 11.099854000 | 7.067962000 | 3.159673000 |
| C | 2.909115000 | 3.306808000 | 9.724186000 |
| C | 7.969701000 | 10.331814000 | 6.604550000 |
| H | 7.344661000 | 10.080385000 | 7.468998000 |
| H | 8.666917000 | 11.119596000 | 6.913909000 |
| H | 8.544349000 | 9.438613000 | 6.341427000 |
| C | 7.202735000 | 0.618412000 | 7.264977000 |
| H | 7.501118000 | 1.465230000 | 6.635092000 |
| C | 4.445204000 | 2.948452000 | 1.208088000 |
| C | 5.441769000 | 7.999228000 | 1.596900000 |

| | | | |
|---|---|---|---|
| H | 4.879296000 | 7.075440000 | 1.421266000 |
| C | 4.222055000 | -0.826644000 | 0.638622000 |
| H | 5.209059000 | -0.777866000 | 0.156573000 |
| C | 9.437435000 | 2.438645000 | 8.102523000 |
| C | 6.900536000 | 4.960434000 | 9.962906000 |
| H | 7.893594000 | 5.402632000 | 9.991855000 |
| C | 9.588735000 | -0.116330000 | 3.275749000 |
| H | 10.350829000 | -0.831790000 | 2.980633000 |
| C | 5.859043000 | 0.179473000 | 12.107229000 |
| H | 4.997748000 | -0.257273000 | 11.581582000 |
| C | 9.549361000 | 3.937817000 | 12.105250000 |
| H | 8.647810000 | 4.529389000 | 11.938853000 |
| H | 10.390445000 | 4.621453000 | 12.265042000 |
| H | 9.413600000 | 3.375087000 | 13.035554000 |
| C | 11.126027000 | 9.473390000 | 4.453455000 |
| H | 10.805453000 | 10.184669000 | 5.227925000 |
| C | 7.239881000 | 5.272338000 | 4.405845000 |
| C | 10.298191000 | 7.763167000 | 9.267420000 |
| H | 11.062158000 | 8.547122000 | 9.210087000 |
| H | 10.166406000 | 7.522645000 | 10.328330000 |
| H | 9.354523000 | 8.166700000 | 8.889733000 |
| C | 10.102775000 | 4.460438000 | 2.622376000 |
| H | 9.080941000 | 4.254496000 | 2.945648000 |
| H | 10.388188000 | 3.657515000 | 1.933355000 |
| H | 10.099806000 | 5.403244000 | 2.064320000 |
| C | 8.665206000 | 2.182787000 | 10.453677000 |
| C | 9.858481000 | 3.008055000 | 10.923610000 |
| H | 10.193018000 | 3.630890000 | 10.084821000 |
| C | 8.284299000 | -0.240401000 | 2.798011000 |
| H | 8.026922000 | -1.050987000 | 2.121099000 |
| C | 1.172751000 | 1.470896000 | 4.370260000 |
| H | 1.132331000 | 0.398394000 | 4.149173000 |
| H | 0.313898000 | 1.716701000 | 5.004588000 |
| H | 2.082114000 | 1.666501000 | 4.947219000 |
| C | 1.141110000 | 2.308594000 | 3.084222000 |
| H | 1.078128000 | 3.360444000 | 3.377067000 |
| C | 8.924941000 | 1.848958000 | 4.502613000 |
| H | 9.177606000 | 2.657680000 | 5.178384000 |
| C | 10.984705000 | 8.084459000 | 5.040737000 |
| C | 8.433229000 | 1.907732000 | 9.087208000 |
| C | 10.724500000 | 6.503302000 | 8.510424000 |
| H | 10.005237000 | 5.713522000 | 8.757500000 |
| C | 6.838989000 | 3.644023000 | 0.619926000 |
| H | 7.206321000 | 3.073950000 | 1.479759000 |
| H | 7.470190000 | 4.531583000 | 0.501980000 |
| H | 6.974452000 | 3.032581000 | -0.279379000 |
| C | 1.952118000 | 3.746533000 | 10.831114000 |
| H | 2.325354000 | 3.421515000 | 11.807019000 |
| H | 0.971545000 | 3.280299000 | 10.697423000 |
| H | 1.823991000 | 4.833989000 | 10.849264000 |
| C | 4.750534000 | 9.121236000 | 0.804129000 |
| H | 3.722304000 | 9.278694000 | 1.141030000 |
| H | 4.728057000 | 8.877634000 | -0.264437000 |
| H | 5.292840000 | 10.065715000 | 0.928396000 |
| C | 9.903631000 | 0.935166000 | 4.129094000 |
| H | 10.913276000 | 1.047331000 | 4.515212000 |
| C | 4.858145000 | 4.763455000 | -0.463845000 |
| H | 5.516448000 | 5.589119000 | -0.756613000 |
| H | 3.851138000 | 5.164390000 | -0.312867000 |
| H | 4.819363000 | 4.045626000 | -1.291707000 |

| | | | |
|---|---|---|---|
| C | 3.029381000 | 1.764252000 | 9.756102000 |
| H | 3.712541000 | 1.404700000 | 8.983103000 |
| H | 2.046178000 | 1.309470000 | 9.592443000 |
| H | 3.414754000 | 1.440131000 | 10.728606000 |
| C | 12.523106000 | 4.510677000 | 3.296053000 |
| H | 12.702435000 | 5.374899000 | 2.646809000 |
| H | 12.728960000 | 3.603916000 | 2.715877000 |
| H | 13.240657000 | 4.566273000 | 4.121159000 |
| C | 8.126625000 | -0.561423000 | 6.918886000 |
| H | 9.181338000 | -0.302570000 | 7.033768000 |
| H | 7.970318000 | -0.869387000 | 5.878923000 |
| H | 7.905325000 | -1.413757000 | 7.572521000 |
| C | 6.858632000 | 7.788952000 | 1.046686000 |
| H | 7.469701000 | 8.693486000 | 1.139261000 |
| H | 6.806281000 | 7.544785000 | -0.020277000 |
| H | 7.380705000 | 6.978911000 | 1.559563000 |
| C | 6.590218000 | -0.958080000 | 12.834743000 |
| H | 6.937329000 | -1.717472000 | 12.126932000 |
| H | 5.932692000 | -1.439608000 | 13.567603000 |
| H | 7.466467000 | -0.570272000 | 13.366868000 |
| C | 4.318072000 | -1.817157000 | 1.806018000 |
| H | 5.036377000 | -1.479415000 | 2.560424000 |
| H | 4.634816000 | -2.802948000 | 1.447835000 |
| H | 3.348285000 | -1.940438000 | 2.300453000 |
| C | 10.226726000 | 9.678164000 | 3.229305000 |
| H | 10.589918000 | 9.108335000 | 2.366597000 |
| H | 10.207222000 | 10.734510000 | 2.938569000 |
| H | 9.204014000 | 9.350347000 | 3.439865000 |
| C | 3.757320000 | 7.224070000 | 7.588171000 |
| H | 4.579112000 | 6.505778000 | 7.515596000 |
| H | 2.938290000 | 6.757023000 | 8.146824000 |
| H | 4.119799000 | 8.074483000 | 8.176529000 |
| C | 3.210820000 | -1.314664000 | -0.407777000 |
| H | 3.491892000 | -2.302763000 | -0.788982000 |
| H | 3.152781000 | -0.620658000 | -1.252191000 |
| H | 2.209298000 | -1.394333000 | 0.030066000 |
| C | 2.170836000 | 8.734159000 | 6.339126000 |
| H | 2.569489000 | 9.635756000 | 6.818720000 |
| H | 1.340687000 | 8.359044000 | 6.949233000 |
| H | 1.775944000 | 9.021500000 | 5.358861000 |
| C | -0.109017000 | 1.987315000 | 2.253934000 |
| H | -0.156409000 | 2.606046000 | 1.351784000 |
| H | -1.016061000 | 2.165871000 | 2.842267000 |
| H | -0.114453000 | 0.937878000 | 1.938285000 |
| C | 12.592582000 | 9.774127000 | 4.113207000 |
| H | 13.231672000 | 9.668843000 | 4.995779000 |
| H | 12.702805000 | 10.792944000 | 3.724154000 |
| H | 12.957113000 | 9.076502000 | 3.350117000 |
| C | 11.026235000 | 2.089453000 | 11.322864000 |
| H | 10.719432000 | 1.428466000 | 12.142069000 |
| H | 11.879785000 | 2.685927000 | 11.665669000 |
| H | 11.360876000 | 1.465100000 | 10.491871000 |
| B | 6.748989000 | 4.124472000 | 5.209409000 |

Coordinates of compound **7**

| | | | |
|---|---|---|---|
| Ge | -4.827875000 | -4.798486000 | -17.076919000 |
| Ge | -1.238664000 | -5.360893000 | -15.432188000 |
| Cl | -4.082100000 | -5.144591000 | -13.834366000 |
| O | -2.098814000 | -4.867983000 | -18.241498000 |
| O | -4.050011000 | -2.959958000 | -16.765750000 |
| C | -5.716945000 | 0.443270000 | -16.332257000 |
| C | -6.906908000 | -9.848739000 | -16.179210000 |
| C | -0.086968000 | -1.850281000 | -11.869917000 |
| H | -0.678855000 | -1.072255000 | -11.391206000 |
| C | -0.623926000 | -3.132323000 | -11.993293000 |
| C | -7.019294000 | -8.185342000 | -17.950046000 |
| C | -2.010770000 | -3.390539000 | -11.434552000 |
| H | -2.323905000 | -4.388749000 | -11.757953000 |
| C | -0.297924000 | -5.572670000 | -12.635724000 |
| C | -6.869986000 | -9.505974000 | -17.521559000 |
| H | -6.713572000 | -10.279735000 | -18.267484000 |
| C | -4.319148000 | -4.941510000 | -18.978318000 |
| C | -6.811391000 | -1.652823000 | -15.752943000 |
| C | -6.809623000 | -4.616028000 | -17.250452000 |
| C | -8.693463000 | -3.279352000 | -18.007708000 |
| H | -9.124234000 | -2.297035000 | -18.183517000 |
| C | -6.280246000 | -0.417098000 | -15.393987000 |
| H | -6.318013000 | -0.113890000 | -14.352096000 |
| C | -5.181875000 | 1.802466000 | -15.932811000 |
| H | -4.507572000 | 2.130149000 | -16.736312000 |
| C | 0.298334000 | -11.015578000 | -18.459320000 |
| H | 1.372889000 | -11.245753000 | -18.491037000 |
| C | -2.003201000 | -3.363365000 | -9.898402000 |
| H | -1.713496000 | -2.373430000 | -9.526816000 |
| H | -3.002560000 | -3.589868000 | -9.510084000 |
| H | -1.302710000 | -4.093271000 | -9.481847000 |
| C | -5.226637000 | -4.983992000 | -20.036811000 |
| H | -6.289789000 | -4.889589000 | -19.836244000 |
| C | -1.777231000 | -1.163681000 | -15.958282000 |
| C | -3.421074000 | -5.280931000 | -21.611132000 |
| H | -3.099132000 | -5.421112000 | -22.638100000 |
| C | -0.388865000 | -8.598916000 | -14.931342000 |
| C | -0.233462000 | -6.232123000 | -11.403446000 |
| H | -0.003291000 | -5.652277000 | -10.514124000 |
| C | -0.565958000 | -6.307983000 | -13.815674000 |
| C | -0.426203000 | -7.602214000 | -11.312181000 |
| H | -0.412972000 | -8.096111000 | -10.344671000 |
| C | -1.017573000 | -0.385462000 | -15.075873000 |
| H | -0.623729000 | -0.844310000 | -14.173428000 |
| C | -1.456618000 | -8.933040000 | -15.779977000 |
| C | -7.087844000 | -8.825157000 | -15.249289000 |
| H | -7.113279000 | -9.090742000 | -14.196169000 |
| C | 1.174786000 | -1.522066000 | -12.352593000 |
| C | 1.431588000 | -3.825173000 | -13.106799000 |
| C | -7.590707000 | -5.778293000 | -17.429464000 |
| C | 0.955717000 | -6.378673000 | -19.548480000 |
| H | 1.458523000 | -6.602059000 | -20.484025000 |
| C | -7.399776000 | -3.352458000 | -17.484136000 |
| C | -4.781689000 | -5.152383000 | -21.343401000 |

| | | | |
|---|---|---|---|
| H | -5.496947000 | -5.189885000 | -22.160049000 |
| C | -5.704288000 | 0.040973000 | -17.663032000 |
| H | -5.270984000 | 0.709838000 | -18.404927000 |
| C | 0.899318000 | -9.114516000 | -15.194371000 |
| C | -8.883678000 | -5.662548000 | -17.957841000 |
| H | -9.474524000 | -6.566638000 | -18.078816000 |
| C | -7.202100000 | -7.161772000 | -16.999365000 |
| C | -2.473806000 | -5.205326000 | -20.589352000 |
| C | -0.548746000 | -8.343160000 | -12.478397000 |
| H | -0.594308000 | -9.427745000 | -12.435972000 |
| C | 0.960394000 | -6.434639000 | -17.135254000 |
| H | 1.442911000 | -6.709853000 | -16.204502000 |
| C | 1.574423000 | -6.722596000 | -18.349601000 |
| H | 2.540427000 | -7.219633000 | -18.363779000 |
| C | -0.285439000 | -5.740212000 | -19.557832000 |
| C | 0.053745000 | -10.194646000 | -17.210230000 |
| C | -1.216855000 | -9.714504000 | -16.907755000 |
| H | -2.045065000 | -9.955260000 | -17.567927000 |
| C | -0.872145000 | -5.496136000 | -18.317812000 |
| C | -7.472449000 | -6.440016000 | -14.543072000 |
| H | -6.896956000 | -5.548440000 | -14.820498000 |
| C | 1.089536000 | -9.902010000 | -16.331075000 |
| H | 2.081401000 | -10.291304000 | -16.549292000 |
| C | -0.964481000 | -5.188752000 | -20.808569000 |
| C | -9.422045000 | -4.427924000 | -18.281792000 |
| H | -10.422740000 | -4.358404000 | -18.698865000 |
| C | -2.305831000 | -0.561201000 | -17.106395000 |
| H | -2.906922000 | -1.142447000 | -17.794182000 |
| C | -6.794886000 | -11.281736000 | -15.699158000 |
| H | -6.306628000 | -11.249923000 | -14.714075000 |
| C | -2.962984000 | -5.019764000 | -19.297166000 |
| C | 2.360296000 | -4.880815000 | -13.687820000 |
| H | 1.749981000 | -5.721647000 | -14.030184000 |
| C | -1.925243000 | -2.614560000 | -15.701668000 |
| C | -6.964565000 | -7.921684000 | -19.444630000 |
| H | -7.068426000 | -6.844599000 | -19.599569000 |
| C | 2.085996000 | -8.875707000 | -14.268576000 |
| H | 1.833896000 | -8.048160000 | -13.595498000 |
| C | -4.733918000 | -1.523170000 | -20.101419000 |
| H | -4.069843000 | -2.169362000 | -19.520133000 |
| H | -4.722697000 | -1.879562000 | -21.137642000 |
| H | -4.324495000 | -0.506361000 | -20.091351000 |
| C | -6.214967000 | -1.191879000 | -18.070793000 |
| C | -0.569388000 | -7.721519000 | -13.729405000 |
| C | -6.162544000 | -1.559740000 | -19.543159000 |
| H | -6.525640000 | -2.587941000 | -19.645862000 |
| C | -0.291768000 | -5.829310000 | -17.096770000 |
| C | -6.761218000 | -2.052824000 | -17.104692000 |
| C | -3.136148000 | -3.375149000 | -15.967911000 |
| C | -0.881891000 | -3.408262000 | -15.330917000 |
| H | 0.101249000 | -2.959017000 | -15.199753000 |
| C | -3.039621000 | -2.384221000 | -11.968106000 |
| H | -2.851053000 | -1.374089000 | -11.587140000 |
| H | -3.030228000 | -2.344185000 | -13.061183000 |
| H | -4.045837000 | -2.675983000 | -11.650139000 |
| C | 0.137289000 | -4.137379000 | -12.625191000 |
| C | 1.917509000 | -2.526290000 | -12.966131000 |
| H | 2.910892000 | -2.297546000 | -13.338545000 |
| C | -7.233148000 | -7.491258000 | -15.618796000 |
| C | -6.994530000 | -6.864766000 | -13.151782000 |

| | | | |
|---|---|---|---|
| H | -7.658212000 | -7.617402000 | -12.709598000 |
| H | -5.975732000 | -7.261306000 | -13.173614000 |
| H | -6.998168000 | -5.995492000 | -12.486319000 |
| C | -1.284723000 | 1.543270000 | -16.497960000 |
| H | -1.092706000 | 2.591327000 | -16.710073000 |
| C | -0.777576000 | 0.957002000 | -15.341384000 |
| H | -0.194602000 | 1.548297000 | -14.640355000 |
| C | -2.047149000 | 0.778215000 | -17.374934000 |
| H | -2.446262000 | 1.225449000 | -18.281587000 |
| C | -3.521986000 | -9.614300000 | -14.562201000 |
| H | -4.543217000 | -9.322667000 | -14.295344000 |
| H | -3.564966000 | -10.565571000 | -15.106424000 |
| H | -2.953049000 | -9.774420000 | -13.640689000 |
| C | -7.517662000 | -2.491697000 | -14.700936000 |
| H | -7.548548000 | -3.525435000 | -15.058752000 |
| C | -2.871266000 | -8.527014000 | -15.431409000 |
| H | -2.831035000 | -7.617983000 | -14.819022000 |
| C | -4.371034000 | 1.761848000 | -14.634144000 |
| H | -3.613840000 | 0.973216000 | -14.667090000 |
| H | -3.862987000 | 2.718958000 | -14.473010000 |
| H | -5.015626000 | 1.583136000 | -13.766003000 |
| C | -0.533159000 | -3.708178000 | -20.961536000 |
| H | 0.553197000 | -3.644021000 | -21.087721000 |
| H | -1.022263000 | -3.259988000 | -21.833734000 |
| H | -0.810772000 | -3.129178000 | -20.074852000 |
| C | -0.050014000 | -10.220148000 | -19.724585000 |
| H | 0.494092000 | -9.270707000 | -19.751097000 |
| H | 0.198225000 | -10.795298000 | -20.623937000 |
| H | -1.120616000 | -9.987804000 | -19.758927000 |
| C | -0.561828000 | -5.962824000 | -22.066308000 |
| H | -0.847346000 | -7.017490000 | -21.993596000 |
| H | -1.030657000 | -5.532038000 | -22.955011000 |
| H | 0.517716000 | -5.902211000 | -22.227364000 |
| C | 2.342930000 | -10.120834000 | -13.404196000 |
| H | 1.455898000 | -10.402262000 | -12.830884000 |
| H | 2.615537000 | -10.973081000 | -14.037587000 |
| H | 3.164219000 | -9.939652000 | -12.701294000 |
| C | -8.957081000 | -6.045290000 | -14.469269000 |
| H | -9.115373000 | -5.329527000 | -13.654181000 |
| H | -9.309941000 | -5.586384000 | -15.396236000 |
| H | -9.572480000 | -6.930340000 | -14.269119000 |
| C | -8.122303000 | -8.607739000 | -20.183210000 |
| H | -9.092687000 | -8.296509000 | -19.783708000 |
| H | -8.094335000 | -8.358671000 | -21.250033000 |
| H | -8.058940000 | -9.697872000 | -20.092720000 |
| C | -5.616353000 | -8.353321000 | -20.039126000 |
| H | -5.476442000 | -9.437311000 | -19.959416000 |
| H | -5.564150000 | -8.084470000 | -21.099734000 |
| H | -4.782652000 | -7.863674000 | -19.527266000 |
| C | 1.715138000 | -0.117957000 | -12.170539000 |
| H | 0.844135000 | 0.548625000 | -12.081955000 |
| C | 3.378643000 | -8.500542000 | -15.006720000 |
| H | 4.161837000 | -8.258001000 | -14.280345000 |
| H | 3.752593000 | -9.327449000 | -15.619351000 |
| H | 3.245667000 | -7.633372000 | -15.660166000 |
| C | 3.286447000 | -5.404870000 | -12.579352000 |
| H | 2.705853000 | -5.828860000 | -11.753373000 |
| H | 3.955222000 | -6.181608000 | -12.967039000 |
| H | 3.901848000 | -4.590312000 | -12.179433000 |
| C | -7.090379000 | -0.658229000 | -20.369819000 |

| | | | |
|---|---|---|---|
| H | -6.764440000 | 0.387065000 | -20.322960000 |
| H | -7.089098000 | -0.965407000 | -21.421946000 |
| H | -8.119611000 | -0.702169000 | -19.998981000 |
| C | -6.328978000 | 2.819001000 | -15.830143000 |
| H | -5.945757000 | 3.815417000 | -15.581319000 |
| H | -6.884493000 | 2.885439000 | -16.771371000 |
| H | -7.033542000 | 2.519284000 | -15.045503000 |
| C | -3.721210000 | -8.230044000 | -16.663948000 |
| H | -4.668686000 | -7.776407000 | -16.362854000 |
| H | -3.201621000 | -7.546641000 | -17.345451000 |
| H | -3.971898000 | -9.140537000 | -17.218147000 |
| C | 2.509855000 | -0.021587000 | -10.858872000 |
| H | 1.896710000 | -0.321378000 | -10.003107000 |
| H | 3.382775000 | -0.683826000 | -10.894798000 |
| H | 2.864117000 | 1.001918000 | -10.691105000 |
| C | -8.197138000 | -11.881763000 | -15.508523000 |
| H | -8.793983000 | -11.280339000 | -14.815560000 |
| H | -8.727683000 | -11.914463000 | -16.467276000 |
| H | -8.134838000 | -12.903041000 | -15.115830000 |
| C | -5.948730000 | -12.173267000 | -16.610057000 |
| H | -5.779574000 | -13.145184000 | -16.135055000 |
| H | -6.450016000 | -12.362910000 | -17.565972000 |
| H | -4.973592000 | -11.721517000 | -16.820671000 |
| C | 3.173368000 | -4.391242000 | -14.890758000 |
| H | 3.913977000 | -3.635325000 | -14.608649000 |
| H | 3.724651000 | -5.229480000 | -15.330628000 |
| H | 2.529658000 | -3.967212000 | -15.668892000 |
| C | -0.465732000 | -12.345604000 | -18.418240000 |
| H | -1.548263000 | -12.174406000 | -18.408640000 |
| H | -0.231458000 | -12.954779000 | -19.298594000 |
| H | -0.209949000 | -12.919535000 | -17.521768000 |
| C | -6.806201000 | -2.498519000 | -13.345571000 |
| H | -7.270797000 | -3.240014000 | -12.685619000 |
| H | -5.749463000 | -2.757335000 | -13.453647000 |
| H | -6.876037000 | -1.528164000 | -12.841109000 |
| C | 2.560806000 | 0.365527000 | -13.352342000 |
| H | 3.517126000 | -0.166540000 | -13.403291000 |
| H | 2.040772000 | 0.218183000 | -14.304477000 |
| H | 2.789446000 | 1.431195000 | -13.243587000 |
| B | -3.319214000 | -4.903323000 | -15.509224000 |
| C | -8.971827000 | -2.017938000 | -14.548506000 |
| H | -8.999859000 | -0.974421000 | -14.213486000 |
| H | -9.510542000 | -2.084084000 | -15.499210000 |
| H | -9.502082000 | -2.631047000 | -13.810652000 |

Coordinates of compound **6-Terph**

| | | | |
|---|---|---|---|
| Ge | 4.444433000 | 4.167223000 | 4.776626000 |
| Ge | 7.572669000 | 4.611932000 | 6.940083000 |
| Cl | 7.728142000 | 6.822851000 | 6.862528000 |
| O | 4.753805000 | 3.518453000 | 7.644880000 |
| O | 7.295368000 | 5.821124000 | 3.197074000 |
| C | 11.827268000 | 4.167562000 | 7.480503000 |
| H | 12.724620000 | 4.708374000 | 7.192051000 |
| C | 3.445607000 | 3.909020000 | 7.474583000 |
| C | 3.860289000 | 7.181291000 | 1.797018000 |
| H | 4.005259000 | 8.255126000 | 1.719340000 |
| C | 0.869937000 | 4.733459000 | 7.042395000 |
| H | -0.151250000 | 5.062618000 | 6.874881000 |
| C | 5.154108000 | 2.415251000 | 4.320240000 |
| H | 4.701818000 | 1.495863000 | 3.951438000 |
| C | 3.075848000 | 4.243204000 | 6.180406000 |
| C | 4.240761000 | 8.305374000 | 6.399767000 |
| H | 3.715749000 | 8.284964000 | 7.350487000 |
| C | 11.911857000 | 3.069424000 | 8.321387000 |
| H | 12.875602000 | 2.746141000 | 8.704558000 |
| C | 2.595149000 | 3.993846000 | 8.577735000 |
| C | 3.231984000 | 5.090157000 | 0.807828000 |
| H | 2.885882000 | 4.511890000 | -0.044787000 |
| C | 3.451804000 | 6.454208000 | 0.689948000 |
| H | 3.288039000 | 6.954030000 | -0.260548000 |
| C | 11.277272000 | 8.080420000 | 5.688689000 |
| H | 11.676606000 | 9.015020000 | 6.072372000 |
| C | 1.756258000 | 4.652141000 | 5.972846000 |
| H | 1.430183000 | 4.952927000 | 4.978921000 |
| C | 5.281013000 | 9.202852000 | 6.192985000 |
| C | 5.940283000 | 9.218695000 | 4.967306000 |
| H | 6.768527000 | 9.901923000 | 4.803064000 |
| C | 5.561559000 | 8.344291000 | 3.957944000 |
| C | 7.565733000 | 1.790218000 | 3.966776000 |
| C | 3.422112000 | 4.444182000 | 2.033555000 |
| C | 2.657599000 | 0.226170000 | 2.161203000 |
| C | 10.596224000 | 4.593206000 | 6.964864000 |
| C | 6.652786000 | 4.455959000 | 8.674917000 |
| C | 4.515710000 | 7.431701000 | 4.153679000 |
| C | 1.290117000 | 4.417348000 | 8.334617000 |
| H | 0.586635000 | 4.509447000 | 9.155879000 |
| C | 3.857350000 | 5.169008000 | 3.164568000 |
| C | 11.186143000 | 6.975859000 | 6.524366000 |
| C | 4.070164000 | 6.561050000 | 3.035902000 |
| C | 5.348815000 | 4.005263000 | 8.793547000 |
| C | 3.858151000 | 7.431152000 | 5.388089000 |
| C | 6.418434000 | 0.607988000 | 7.894482000 |
| H | 5.764243000 | 0.227380000 | 7.115061000 |
| C | 3.576501000 | 0.747429000 | 1.253741000 |
| H | 4.112944000 | 0.084745000 | 0.580132000 |
| C | 9.410396000 | 3.911806000 | 7.317455000 |
| C | 10.648576000 | 5.770914000 | 6.059681000 |
| C | 4.606509000 | 4.065351000 | 9.974942000 |
| C | 5.254248000 | 4.564397000 | 11.102040000 |
| H | 4.725497000 | 4.637980000 | 12.046827000 |
| C | 7.299801000 | 0.693643000 | 3.129388000 |
| H | 6.279215000 | 0.497008000 | 2.812859000 |
| C | 7.488982000 | 1.424755000 | 7.546488000 |

| | | | |
|---|---|---|---|
| C | 10.760318000 | 2.369864000 | 8.644453000 |
| H | 10.814346000 | 1.480933000 | 9.266990000 |
| C | 10.240560000 | 5.689226000 | 4.727740000 |
| C | 3.151982000 | 2.985307000 | 2.082679000 |
| C | 1.975738000 | 1.084941000 | 3.019253000 |
| H | 1.249027000 | 0.689713000 | 3.723201000 |
| C | 6.582672000 | 4.983568000 | 11.032658000 |
| H | 7.071269000 | 5.368571000 | 11.922908000 |
| C | 6.195939000 | 0.283426000 | 9.227057000 |
| C | 6.478748000 | 2.666847000 | 4.436470000 |
| C | 7.045745000 | 0.787353000 | 10.210570000 |
| H | 6.868471000 | 0.550333000 | 11.255924000 |
| C | 2.217089000 | 2.453548000 | 2.975673000 |
| C | 10.339854000 | 6.793045000 | 3.886806000 |
| H | 10.016051000 | 6.710902000 | 2.853581000 |
| C | 3.159764000 | 3.576983000 | 9.932403000 |
| C | 3.820289000 | 2.115842000 | 1.213241000 |
| C | 9.514639000 | 2.769926000 | 8.148299000 |
| C | 7.272453000 | 4.942550000 | 9.827393000 |
| H | 8.294223000 | 5.311640000 | 9.774642000 |
| C | 9.647634000 | 0.133315000 | 3.051725000 |
| H | 10.449179000 | -0.508058000 | 2.697042000 |
| C | 7.058574000 | 5.114303000 | 4.068154000 |
| C | 8.116173000 | 1.598564000 | 9.861989000 |
| C | 8.327619000 | -0.123113000 | 2.681511000 |
| H | 8.101040000 | -0.963378000 | 2.030924000 |
| C | 8.898566000 | 2.039783000 | 4.317548000 |
| H | 9.128164000 | 2.868493000 | 4.979964000 |
| C | 10.849996000 | 7.993863000 | 4.365987000 |
| C | 8.348940000 | 1.932678000 | 8.522063000 |
| C | 2.332052000 | 4.122491000 | 11.095859000 |
| H | 2.745579000 | 3.786003000 | 12.050784000 |
| H | 1.306942000 | 3.743763000 | 11.048092000 |
| H | 2.303695000 | 5.217091000 | 11.092923000 |
| C | 9.928863000 | 1.219970000 | 3.872452000 |
| H | 10.950983000 | 1.433190000 | 4.172521000 |
| C | 3.154774000 | 2.030276000 | 9.992600000 |
| H | 3.724866000 | 1.608803000 | 9.161139000 |
| H | 2.126060000 | 1.656650000 | 9.940261000 |
| H | 3.613368000 | 1.691007000 | 10.928074000 |
| B | 6.641213000 | 4.145425000 | 5.108809000 |
| H | 6.095238000 | 8.346585000 | 3.011680000 |
| H | 4.557475000 | 2.519173000 | 0.523821000 |
| H | 2.470326000 | -0.843121000 | 2.195908000 |
| H | 1.673804000 | 3.118136000 | 3.640528000 |
| H | 5.364746000 | -0.358813000 | 9.503539000 |
| H | 7.680575000 | 1.634753000 | 6.500723000 |
| H | 8.761144000 | 2.010773000 | 10.633298000 |
| H | 11.499299000 | 7.049104000 | 7.562502000 |
| H | 10.921188000 | 8.857258000 | 3.710791000 |
| H | 5.587527000 | 9.877698000 | 6.986629000 |
| H | 3.013634000 | 6.771556000 | 5.546743000 |
| H | 9.863775000 | 4.749200000 | 4.335012000 |

Coordinates of compound **7-Terph**

| | | | |
|---|---|---|---|
| Ge | 4.593584000 | 4.798202000 | 17.198687000 |
| Ge | 1.237287000 | 5.044678000 | 15.309050000 |
| Cl | 4.148233000 | 4.738238000 | 13.880898000 |
| O | 1.922203000 | 5.059333000 | 18.242911000 |
| O | 3.700580000 | 2.963850000 | 17.233078000 |
| C | 5.313456000 | -0.308809000 | 15.958413000 |
| C | 5.993993000 | 9.578468000 | 16.311653000 |
| C | 1.143254000 | 2.375878000 | 11.666644000 |
| H | 2.090105000 | 1.877865000 | 11.476446000 |
| C | 1.139564000 | 3.706505000 | 12.071895000 |
| C | 6.939834000 | 8.132237000 | 17.997094000 |
| C | -0.078401000 | 5.799514000 | 12.720434000 |
| C | 6.536942000 | 9.395398000 | 17.581932000 |
| H | 6.645293000 | 10.242579000 | 18.253167000 |
| C | 4.094573000 | 5.465737000 | 18.976879000 |
| C | 6.038351000 | 1.963918000 | 15.617632000 |
| C | 6.554050000 | 4.526104000 | 17.285274000 |
| C | 8.478535000 | 3.182293000 | 17.889516000 |
| H | 8.943612000 | 2.203716000 | 17.974765000 |
| C | 5.440879000 | 0.806044000 | 15.135542000 |
| H | 5.067783000 | 0.777600000 | 14.115527000 |
| C | 4.967347000 | 5.916371000 | 19.965462000 |
| H | 6.041127000 | 5.842248000 | 19.815142000 |
| C | 1.856996000 | 0.904787000 | 15.905294000 |
| C | 3.089611000 | 6.553397000 | 21.349388000 |
| H | 2.728148000 | 6.979647000 | 22.279990000 |
| C | 1.027169000 | 8.163475000 | 15.483229000 |
| C | -0.749648000 | 6.700556000 | 11.884971000 |
| H | -1.212296000 | 6.327878000 | 10.974734000 |
| C | 0.533911000 | 6.254136000 | 13.902991000 |
| C | -0.807319000 | 8.050407000 | 12.199375000 |
| H | -1.317289000 | 8.743503000 | 11.536207000 |
| C | 1.443478000 | 0.075537000 | 14.855962000 |
| H | 1.196391000 | 0.521684000 | 13.895834000 |
| C | 2.357695000 | 7.935465000 | 15.847751000 |
| C | 5.840645000 | 8.486863000 | 15.464375000 |
| H | 5.419383000 | 8.618510000 | 14.472021000 |
| C | -0.056329000 | 1.687574000 | 11.507284000 |
| C | -1.266879000 | 3.674831000 | 12.144174000 |
| C | 7.321514000 | 5.687292000 | 17.509277000 |
| C | -1.351360000 | 6.435167000 | 19.070472000 |
| H | -1.929058000 | 6.814001000 | 19.907577000 |
| C | 7.168234000 | 3.265508000 | 17.403765000 |
| C | 4.465154000 | 6.467883000 | 21.140782000 |
| H | 5.146546000 | 6.826614000 | 21.906464000 |
| C | 5.793673000 | -0.260759000 | 17.263900000 |
| H | 5.687043000 | -1.125848000 | 17.912463000 |
| C | 0.197364000 | 8.859923000 | 16.370344000 |
| C | 8.632356000 | 5.578246000 | 17.983809000 |
| H | 9.219283000 | 6.480086000 | 18.136692000 |
| C | 6.797401000 | 7.026037000 | 17.149872000 |
| C | 2.180073000 | 6.080096000 | 20.402339000 |
| C | -0.201552000 | 8.512484000 | 13.359607000 |
| H | -0.232105000 | 9.569281000 | 13.611945000 |
| C | -1.233345000 | 5.896465000 | 16.718276000 |
| H | -1.697448000 | 5.887251000 | 15.734175000 |
| C | -1.954498000 | 6.352067000 | 17.818327000 |

| | | | |
|---|---|---|---|
| H | -2.988486000 | 6.662490000 | 17.698628000 |
| C | -0.030273000 | 6.027980000 | 19.261645000 |
| C | 1.981991000 | 8.990425000 | 17.984773000 |
| C | 2.827093000 | 8.336774000 | 17.095073000 |
| H | 3.857046000 | 8.138487000 | 17.373154000 |
| C | 0.637883000 | 5.542645000 | 18.139728000 |
| C | 0.668990000 | 9.266804000 | 17.611521000 |
| H | 0.000856000 | 9.778230000 | 18.298925000 |
| C | 0.670119000 | 6.005059000 | 20.618638000 |
| C | 9.193951000 | 4.328275000 | 18.208610000 |
| H | 10.212675000 | 4.248216000 | 18.577080000 |
| C | 2.191516000 | 0.328564000 | 17.135971000 |
| H | 2.524804000 | 0.960216000 | 17.951665000 |
| C | 2.725573000 | 5.552476000 | 19.234274000 |
| C | 1.920039000 | 2.366100000 | 15.692773000 |
| C | 6.392339000 | 0.896862000 | 17.748673000 |
| C | 0.466183000 | 7.631452000 | 14.215358000 |
| C | 0.095235000 | 5.500576000 | 16.860987000 |
| C | 6.508450000 | 2.025815000 | 16.933486000 |
| C | 3.028096000 | 3.197446000 | 16.163287000 |
| C | 0.944316000 | 3.095441000 | 15.082889000 |
| H | 0.047307000 | 2.600486000 | 14.711667000 |
| C | -0.065716000 | 4.370348000 | 12.316245000 |
| C | -1.262181000 | 2.340693000 | 11.749694000 |
| H | -2.202516000 | 1.809791000 | 11.631246000 |
| C | 6.233473000 | 7.218456000 | 15.879941000 |
| C | 1.687203000 | -1.866900000 | 16.263137000 |
| H | 1.620567000 | -2.942047000 | 16.403870000 |
| C | 1.363750000 | -1.300277000 | 15.032490000 |
| H | 1.048731000 | -1.932410000 | 14.206852000 |
| C | 2.095456000 | -1.047117000 | 17.311029000 |
| H | 2.348414000 | -1.482023000 | 18.274089000 |
| C | 0.358785000 | 4.646700000 | 21.294801000 |
| H | -0.721173000 | 4.539773000 | 21.445373000 |
| H | 0.863492000 | 4.582886000 | 22.265346000 |
| H | 0.702932000 | 3.815795000 | 20.671110000 |
| C | 0.177084000 | 7.142072000 | 21.520495000 |
| H | 0.383827000 | 8.118635000 | 21.070551000 |
| H | 0.656160000 | 7.097856000 | 22.502204000 |
| H | -0.898151000 | 7.059515000 | 21.697820000 |
| B | 3.303194000 | 4.636186000 | 15.542316000 |
| H | 5.681861000 | 10.567355000 | 15.989439000 |
| H | 6.153485000 | 6.377229000 | 15.195699000 |
| H | 7.360740000 | 7.994517000 | 18.989649000 |
| H | 6.749749000 | 0.940107000 | 18.774551000 |
| H | 6.139410000 | 2.833384000 | 14.973228000 |
| H | 4.823758000 | -1.205548000 | 15.589837000 |
| H | 2.075659000 | 4.241612000 | 12.194991000 |
| H | -0.053471000 | 0.647651000 | 11.192393000 |
| H | -2.207971000 | 4.183491000 | 12.338342000 |
| H | 3.027478000 | 7.439707000 | 15.150490000 |
| H | 2.349406000 | 9.282698000 | 18.964704000 |
| H | -0.841441000 | 9.030500000 | 16.101068000 |

Coordinates of compound **6-Ph**

| | | | |
|---|---|---|---|
| Ge | -4.136387000 | -4.207966000 | -4.705085000 |
| Ge | -7.330243000 | -4.573808000 | -6.850495000 |
| Cl | -7.686202000 | -6.760427000 | -6.943356000 |
| O | -4.562709000 | -3.411611000 | -7.516565000 |
| O | -6.976744000 | -6.102620000 | -3.360269000 |
| C | -11.450209000 | -3.770581000 | -6.208993000 |
| H | -12.300646000 | -4.277505000 | -5.761006000 |
| C | -3.256986000 | -3.841729000 | -7.427097000 |
| C | -2.585736000 | -7.369365000 | -2.351847000 |
| H | -2.210931000 | -8.373853000 | -2.527717000 |
| C | -0.691512000 | -4.748658000 | -7.158847000 |
| H | 0.328187000 | -5.107984000 | -7.058198000 |
| C | -4.861980000 | -2.471850000 | -4.214267000 |
| H | -4.460327000 | -1.543834000 | -3.807677000 |
| C | -2.850000000 | -4.288383000 | -6.176638000 |
| C | -11.596322000 | -2.484684000 | -6.722953000 |
| H | -12.559287000 | -1.984004000 | -6.670879000 |
| C | -2.445703000 | -3.829728000 | -8.559609000 |
| C | -3.148025000 | -5.585307000 | -0.832417000 |
| H | -3.209608000 | -5.193747000 | 0.179178000 |
| C | -2.656941000 | -6.868434000 | -1.054560000 |
| H | -2.334634000 | -7.480372000 | -0.216836000 |
| C | -1.533076000 | -4.736387000 | -6.049639000 |
| H | -1.169125000 | -5.088670000 | -5.087885000 |
| C | -7.278072000 | -1.759737000 | -4.340209000 |
| C | -3.563709000 | -4.802941000 | -1.906301000 |
| C | -10.216212000 | -4.411406000 | -6.272459000 |
| C | -6.518624000 | -4.161698000 | -8.587707000 |
| C | -1.144942000 | -4.306033000 | -8.401183000 |
| H | -0.468257000 | -4.332829000 | -9.249334000 |
| C | -3.485811000 | -5.289928000 | -3.214951000 |
| C | -2.999392000 | -6.585661000 | -3.424654000 |
| C | -5.200707000 | -3.742634000 | -8.694455000 |
| C | -9.117794000 | -3.774206000 | -6.854868000 |
| C | -4.497085000 | -3.678106000 | -9.897806000 |
| C | -5.202457000 | -4.000234000 | -11.055110000 |
| H | -4.705939000 | -3.971892000 | -12.019728000 |
| C | -7.090512000 | -0.390009000 | -4.572774000 |
| H | -6.114120000 | -0.035295000 | -4.891635000 |
| C | -10.508528000 | -1.843293000 | -7.307842000 |
| H | -10.618038000 | -0.839797000 | -7.709340000 |
| C | -6.548439000 | -4.361696000 | -10.995771000 |
| H | -7.081274000 | -4.598196000 | -11.911725000 |
| C | -6.165900000 | -2.710055000 | -4.489020000 |
| C | -3.034808000 | -3.243415000 | -9.840590000 |
| C | -9.276297000 | -2.485943000 | -7.374632000 |
| C | -7.201320000 | -4.452743000 | -9.771701000 |
| H | -8.236457000 | -4.783249000 | -9.730926000 |
| C | -9.404792000 | 0.043614000 | -4.048032000 |
| H | -10.230189000 | 0.741758000 | -3.942671000 |
| C | -6.730796000 | -5.253279000 | -4.092010000 |
| C | -8.145643000 | 0.501811000 | -4.429408000 |
| H | -7.988277000 | 1.558938000 | -4.624544000 |
| C | -8.553130000 | -2.207823000 | -3.974414000 |
| H | -8.721225000 | -3.269609000 | -3.814603000 |
| C | -2.255348000 | -3.672712000 | -11.083149000 |
| H | -2.680529000 | -3.215012000 | -11.980845000 |

| | | | |
|---|---|---|---|
| H | -1.217864000 | -3.332161000 | -11.020371000 |
| H | -2.261476000 | -4.760520000 | -11.207328000 |
| C | -9.604122000 | -1.314603000 | -3.817631000 |
| H | -10.585735000 | -1.683098000 | -3.535379000 |
| C | -2.987762000 | -1.698858000 | -9.725369000 |
| H | -3.540461000 | -1.353539000 | -8.847075000 |
| H | -1.949309000 | -1.362350000 | -9.635114000 |
| H | -3.433813000 | -1.243482000 | -10.616147000 |
| B | -6.307107000 | -4.221425000 | -5.059640000 |
| H | -3.953910000 | -3.803430000 | -1.724461000 |
| H | -2.943942000 | -6.990689000 | -4.433364000 |
| H | -10.111426000 | -5.419203000 | -5.876909000 |
| H | -8.431578000 | -1.974830000 | -7.832566000 |

Coordinates of compound **7-Ph**

| | | | |
|---|---|---|---|
| Ge | 4.648007000 | 4.779335000 | 17.504708000 |
| Ge | 1.359365000 | 5.291543000 | 15.617604000 |
| Cl | 4.251060000 | 5.102586000 | 14.168659000 |
| O | 1.908759000 | 4.809488000 | 18.552260000 |
| O | 3.859308000 | 2.917714000 | 17.196415000 |
| C | 0.687037000 | 5.767824000 | 12.826741000 |
| C | 4.097549000 | 5.147149000 | 19.341597000 |
| C | 6.575763000 | 4.506605000 | 17.464936000 |
| C | 8.586720000 | 3.507149000 | 18.374585000 |
| H | 9.070510000 | 2.880374000 | 19.118599000 |
| C | 4.956684000 | 5.530357000 | 20.375390000 |
| H | 6.030920000 | 5.544517000 | 20.209540000 |
| C | 2.025661000 | 1.079099000 | 15.548524000 |
| C | 3.061798000 | 5.932287000 | 21.822739000 |
| H | 2.683487000 | 6.247667000 | 22.789800000 |
| C | 0.318157000 | 6.515308000 | 11.712073000 |
| H | 0.253522000 | 6.039451000 | 10.737374000 |
| C | 0.775032000 | 6.360978000 | 14.089702000 |
| C | 0.039732000 | 7.872338000 | 11.845317000 |
| H | -0.246296000 | 8.457945000 | 10.976033000 |
| C | 1.467348000 | 0.466544000 | 14.418882000 |
| H | 1.095629000 | 1.086943000 | 13.607658000 |
| C | 7.342151000 | 5.095431000 | 16.452985000 |
| C | -1.143233000 | 6.585143000 | 19.477713000 |
| H | -1.695793000 | 6.914221000 | 20.351773000 |
| C | 7.211383000 | 3.708244000 | 18.424239000 |
| C | 4.439174000 | 5.920986000 | 21.606409000 |
| H | 5.109746000 | 6.227406000 | 22.403508000 |
| C | 8.720188000 | 4.902189000 | 16.411430000 |
| H | 9.307386000 | 5.364305000 | 15.622956000 |
| C | 2.172111000 | 5.547193000 | 20.820440000 |
| C | 0.137541000 | 8.479254000 | 13.094512000 |
| H | -0.070328000 | 9.540455000 | 13.201786000 |
| C | -0.956787000 | 6.463630000 | 17.069845000 |
| H | -1.355799000 | 6.699499000 | 16.086585000 |
| C | -1.649371000 | 6.861954000 | 18.209853000 |
| H | -2.589352000 | 7.396908000 | 18.111871000 |
| C | 0.053374000 | 5.885757000 | 19.629946000 |
| C | 0.713381000 | 5.504055000 | 18.462461000 |
| C | 0.656800000 | 5.456854000 | 20.964554000 |
| C | 9.342012000 | 4.109027000 | 17.370859000 |

| | | | |
|---|---|---|---|
| H | 10.416751000 | 3.954209000 | 17.334063000 |
| C | 2.543923000 | 0.270926000 | 16.568640000 |
| H | 2.979205000 | 0.729489000 | 17.448659000 |
| C | 2.728657000 | 5.169190000 | 19.600085000 |
| C | 2.044403000 | 2.553818000 | 15.631180000 |
| C | 0.505956000 | 7.728818000 | 14.207245000 |
| C | 0.261057000 | 5.787383000 | 17.178132000 |
| C | 3.157302000 | 3.322407000 | 16.203040000 |
| C | 1.027899000 | 3.368590000 | 15.243393000 |
| H | 0.096225000 | 2.930727000 | 14.883626000 |
| C | 1.919565000 | -1.713333000 | 15.340096000 |
| H | 1.880117000 | -2.795775000 | 15.259925000 |
| C | 1.413819000 | -0.916750000 | 14.315963000 |
| H | 0.985317000 | -1.375004000 | 13.429287000 |
| C | 2.482468000 | -1.114171000 | 16.462452000 |
| H | 2.878842000 | -1.729464000 | 17.265164000 |
| C | 0.285268000 | 3.971656000 | 21.205129000 |
| H | -0.802876000 | 3.866120000 | 21.271631000 |
| H | 0.735498000 | 3.618391000 | 22.139286000 |
| H | 0.643361000 | 3.339029000 | 20.387905000 |
| C | 0.135818000 | 6.292987000 | 22.133193000 |
| H | 0.371479000 | 7.354902000 | 22.007525000 |
| H | 0.568831000 | 5.946026000 | 23.075777000 |
| H | -0.947946000 | 6.183433000 | 22.229230000 |
| B | 3.408515000 | 4.852200000 | 15.805644000 |
| H | 6.859859000 | 5.696034000 | 15.685917000 |
| H | 6.629022000 | 3.232167000 | 19.210292000 |
| H | 0.919529000 | 4.711101000 | 12.708561000 |
| H | 0.581849000 | 8.217471000 | 15.176659000 |

Coordinates of compound **6-Me**

| | | | |
|---|---|---|---|
| Ge | 4.429398000 | 3.998305000 | 4.719769000 |
| Ge | 7.399270000 | 3.806734000 | 7.059213000 |
| Cl | 9.355817000 | 4.854003000 | 7.141134000 |
| O | 4.630578000 | 3.341813000 | 7.714768000 |
| O | 7.215803000 | 6.510683000 | 4.314491000 |
| C | 3.327841000 | 3.698979000 | 7.421688000 |
| C | 0.752982000 | 4.395643000 | 6.790304000 |
| H | -0.270378000 | 4.660681000 | 6.542045000 |
| C | 5.318507000 | 2.443298000 | 3.957148000 |
| H | 5.005459000 | 1.587387000 | 3.360495000 |
| C | 3.019871000 | 3.913886000 | 6.083627000 |
| C | 2.424669000 | 3.888620000 | 8.469420000 |
| C | 1.697977000 | 4.255343000 | 5.779526000 |
| H | 1.407343000 | 4.442778000 | 4.749140000 |
| C | 7.827049000 | 2.125503000 | 3.794826000 |
| C | 6.485458000 | 4.410152000 | 8.685694000 |
| C | 1.120363000 | 4.236921000 | 8.125036000 |
| H | 0.378422000 | 4.396375000 | 8.900752000 |
| C | 5.162116000 | 4.014327000 | 8.806557000 |
| C | 4.359265000 | 4.268667000 | 9.914470000 |
| C | 4.946550000 | 4.984873000 | 10.956852000 |
| H | 4.370165000 | 5.225880000 | 11.844350000 |
| C | 7.802068000 | 0.807136000 | 3.312578000 |
| H | 6.858479000 | 0.271187000 | 3.267798000 |
| C | 6.275781000 | 5.399197000 | 10.876362000 |
| H | 6.712255000 | 5.955637000 | 11.700457000 |

| | | | |
|---|---|---|---|
| C | 6.596732000 | 2.808378000 | 4.222485000 |
| C | 2.941395000 | 3.704213000 | 9.893990000 |
| C | 7.047466000 | 5.109476000 | 9.753302000 |
| H | 8.080730000 | 5.440469000 | 9.697251000 |
| C | 10.193103000 | 0.842761000 | 2.991689000 |
| H | 11.107497000 | 0.343288000 | 2.685111000 |
| C | 6.972508000 | 5.440485000 | 4.654084000 |
| C | 8.970722000 | 0.175423000 | 2.914956000 |
| H | 8.933146000 | -0.847370000 | 2.550606000 |
| C | 9.063609000 | 2.778297000 | 3.876705000 |
| H | 9.111762000 | 3.789517000 | 4.272603000 |
| C | 2.038144000 | 4.374331000 | 10.928595000 |
| H | 2.433697000 | 4.218545000 | 11.936340000 |
| H | 1.038472000 | 3.930660000 | 10.911870000 |
| H | 1.948048000 | 5.450883000 | 10.750040000 |
| C | 10.234776000 | 2.146112000 | 3.474766000 |
| H | 11.182022000 | 2.672000000 | 3.549511000 |
| C | 3.016249000 | 2.187717000 | 10.200734000 |
| H | 3.657596000 | 1.670403000 | 9.481967000 |
| H | 2.015110000 | 1.745813000 | 10.151433000 |
| H | 3.425039000 | 2.029725000 | 11.204516000 |
| B | 6.551462000 | 4.128118000 | 5.167242000 |
| C | 7.806372000 | 1.902765000 | 7.278084000 |
| H | 6.879392000 | 1.332873000 | 7.160759000 |
| H | 8.526294000 | 1.574102000 | 6.525925000 |
| H | 8.212718000 | 1.736881000 | 8.279174000 |
| C | 3.787065000 | 5.235020000 | 3.328064000 |
| H | 4.582010000 | 5.409836000 | 2.597934000 |
| H | 2.929789000 | 4.806158000 | 2.799544000 |
| H | 3.486012000 | 6.191411000 | 3.765112000 |

Coordinates of compound **7-Me**

| | | | |
|---|---|---|---|
| Ge | 4.625572000 | 4.903121000 | 17.450743000 |
| Ge | 1.299501000 | 5.354401000 | 15.591114000 |
| Cl | 4.230439000 | 5.213014000 | 14.164325000 |
| O | 1.878239000 | 4.831656000 | 18.519882000 |
| O | 3.964592000 | 3.006580000 | 17.086671000 |
| C | 4.070579000 | 5.168517000 | 19.303953000 |
| C | 4.933664000 | 5.494437000 | 20.353507000 |
| H | 6.008821000 | 5.502917000 | 20.191216000 |
| C | 2.051912000 | 1.140620000 | 15.606710000 |
| C | 3.045872000 | 5.847494000 | 21.820792000 |
| H | 2.671454000 | 6.127068000 | 22.800294000 |
| C | 1.504733000 | 0.493620000 | 14.491371000 |
| H | 1.126794000 | 1.089961000 | 13.665012000 |
| C | -1.145478000 | 6.627238000 | 19.500537000 |
| H | -1.686699000 | 6.947054000 | 20.384983000 |
| C | 4.423032000 | 5.832437000 | 21.602781000 |
| H | 5.097101000 | 6.094166000 | 22.412768000 |
| C | 2.152038000 | 5.506179000 | 20.806433000 |
| C | -0.969946000 | 6.559923000 | 17.090478000 |
| H | -1.367849000 | 6.835541000 | 16.117006000 |
| C | -1.646732000 | 6.952136000 | 18.242383000 |
| H | -2.569794000 | 7.518674000 | 18.162240000 |
| C | 0.035859000 | 5.897411000 | 19.629486000 |
| C | 0.683385000 | 5.531868000 | 18.449952000 |

| | | | |
|---|---|---|---|
| C | 0.636342000 | 5.423459000 | 20.950315000 |
| C | 2.575013000 | 0.365223000 | 16.649430000 |
| H | 2.999961000 | 0.851769000 | 17.519488000 |
| C | 2.703031000 | 5.170069000 | 19.571126000 |
| C | 2.049752000 | 2.617762000 | 15.654882000 |
| C | 0.226616000 | 5.844194000 | 17.173758000 |
| C | 3.168166000 | 3.413460000 | 16.167255000 |
| C | 1.001432000 | 3.405274000 | 15.295272000 |
| H | 0.067450000 | 2.932975000 | 14.987320000 |
| C | 1.981311000 | -1.656848000 | 15.468249000 |
| H | 1.955745000 | -2.741394000 | 15.415225000 |
| C | 1.469469000 | -0.892809000 | 14.422787000 |
| H | 1.049588000 | -1.378654000 | 13.546719000 |
| C | 2.531169000 | -1.022779000 | 16.578086000 |
| H | 2.930531000 | -1.612871000 | 17.398090000 |
| C | 0.252281000 | 3.935036000 | 21.146657000 |
| H | -0.836800000 | 3.836559000 | 21.208442000 |
| H | 0.697939000 | 3.550501000 | 22.070623000 |
| H | 0.606447000 | 3.324050000 | 20.311507000 |
| C | 0.123563000 | 6.228669000 | 22.144028000 |
| H | 0.369170000 | 7.291630000 | 22.050291000 |
| H | 0.553176000 | 5.849562000 | 23.075695000 |
| H | -0.961223000 | 6.126267000 | 22.237145000 |
| B | 3.340875000 | 4.963772000 | 15.789060000 |
| C | 6.574723000 | 4.770948000 | 17.364749000 |
| H | 6.940012000 | 4.023956000 | 18.075461000 |
| H | 6.875060000 | 4.491576000 | 16.352396000 |
| H | 7.026910000 | 5.738418000 | 17.606103000 |
| C | 0.622427000 | 6.383933000 | 14.063032000 |
| H | 1.260395000 | 6.195580000 | 13.195849000 |
| H | -0.403464000 | 6.094012000 | 13.815599000 |
| H | 0.642602000 | 7.455019000 | 14.283615000 |

Coordinates of compound **6-H**

| | | | |
|---|---|---|---|
| Ge | -4.156894000 | -3.829828000 | -4.903341000 |
| Ge | -7.226736000 | -3.611463000 | -7.104187000 |
| Cl | -9.144625000 | -4.715604000 | -7.110353000 |
| O | -4.453017000 | -3.151770000 | -7.865108000 |
| O | -7.091240000 | -6.147649000 | -4.192926000 |
| C | -3.157477000 | -3.578283000 | -7.636528000 |
| C | -0.606375000 | -4.442370000 | -7.123684000 |
| H | 0.406468000 | -4.778761000 | -6.923780000 |
| C | -4.944349000 | -2.217831000 | -4.155669000 |
| H | -4.561076000 | -1.354868000 | -3.613996000 |
| C | -2.809124000 | -3.846183000 | -6.319036000 |
| C | -2.304732000 | -3.780465000 | -8.722339000 |
| C | -1.502199000 | -4.277187000 | -6.072584000 |
| H | -1.191040000 | -4.512554000 | -5.057671000 |
| C | -7.428412000 | -1.751330000 | -3.957558000 |
| C | -6.385818000 | -4.112524000 | -8.799127000 |
| C | -1.011548000 | -4.215120000 | -8.437713000 |
| H | -0.308398000 | -4.389770000 | -9.245560000 |
| C | -5.053922000 | -3.759976000 | -8.956608000 |
| C | -4.306031000 | -4.006313000 | -10.105115000 |
| C | -4.962599000 | -4.653974000 | -11.150244000 |
| H | -4.430323000 | -4.883323000 | -12.067828000 |

| | | | |
|---|---|---|---|
| C | -7.314942000 | -0.429604000 | -3.497272000 |
| H | -6.339076000 | 0.046041000 | -3.467914000 |
| C | -6.304351000 | -5.016107000 | -11.034252000 |
| H | -6.795922000 | -5.519097000 | -11.861465000 |
| C | -6.247184000 | -2.520983000 | -4.373090000 |
| C | -2.861340000 | -3.514271000 | -10.118234000 |
| C | -7.017642000 | -4.745616000 | -9.869526000 |
| H | -8.058898000 | -5.042862000 | -9.783423000 |
| C | -9.698829000 | -0.309944000 | -3.147879000 |
| H | -10.575873000 | 0.250060000 | -2.836853000 |
| C | -6.777565000 | -5.134825000 | -4.629076000 |
| C | -8.436049000 | 0.280025000 | -3.095942000 |
| H | -8.329177000 | 1.303091000 | -2.746485000 |
| C | -8.704647000 | -2.325450000 | -4.015646000 |
| H | -8.824465000 | -3.339935000 | -4.388081000 |
| C | -2.031386000 | -4.188380000 | -11.210079000 |
| H | -2.450010000 | -3.969485000 | -12.196549000 |
| H | -1.009009000 | -3.799734000 | -11.210038000 |
| H | -1.994620000 | -5.274760000 | -11.078323000 |
| C | -9.828654000 | -1.614555000 | -3.610609000 |
| H | -10.807783000 | -2.081036000 | -3.665349000 |
| C | -2.867808000 | -1.984960000 | -10.365649000 |
| H | -3.451061000 | -1.462654000 | -9.602497000 |
| H | -1.843211000 | -1.598476000 | -10.342737000 |
| H | -3.307398000 | -1.768037000 | -11.345023000 |
| B | -6.291291000 | -3.888725000 | -5.250131000 |
| H | -3.612815000 | -4.787535000 | -3.823165000 |
| H | -7.647667000 | -2.135376000 | -7.176217000 |

Coordinates of compound **7-H**

| | | | |
|---|---|---|---|
| Ge | 4.612506000 | 4.898574000 | 17.450773000 |
| Ge | 1.300068000 | 5.345627000 | 15.600722000 |
| Cl | 4.225954000 | 5.211749000 | 14.150501000 |
| O | 1.873633000 | 4.826742000 | 18.519005000 |
| O | 3.977156000 | 3.010287000 | 17.068432000 |
| C | 4.066766000 | 5.156530000 | 19.300868000 |
| C | 4.935151000 | 5.480141000 | 20.347178000 |
| H | 6.009560000 | 5.480616000 | 20.180642000 |
| C | 2.063156000 | 1.136502000 | 15.587363000 |
| C | 3.049848000 | 5.846177000 | 21.815422000 |
| H | 2.678591000 | 6.130041000 | 22.794877000 |
| C | 1.504032000 | 0.493731000 | 14.475545000 |
| H | 1.114452000 | 1.092521000 | 13.656451000 |
| C | -1.141986000 | 6.638710000 | 19.494889000 |
| H | -1.682224000 | 6.963909000 | 20.377958000 |
| C | 4.426813000 | 5.823740000 | 21.595651000 |
| H | 5.103042000 | 6.083426000 | 22.404377000 |
| C | 2.151790000 | 5.506425000 | 20.804038000 |
| C | -0.965711000 | 6.566338000 | 17.084445000 |
| H | -1.358443000 | 6.840561000 | 16.108328000 |
| C | -1.641264000 | 6.962834000 | 18.235449000 |
| H | -2.561174000 | 7.534065000 | 18.153667000 |
| C | 0.035971000 | 5.904026000 | 19.627580000 |
| C | 0.681518000 | 5.531952000 | 18.449387000 |
| C | 0.635520000 | 5.432726000 | 20.950004000 |
| C | 2.602834000 | 0.358342000 | 16.619414000 |

| | | | |
|---|---|---|---|
| H | 3.037413000 | 0.841273000 | 17.486750000 |
| C | 2.699513000 | 5.164118000 | 19.569517000 |
| C | 2.057935000 | 2.612979000 | 15.642423000 |
| C | 0.226230000 | 5.844139000 | 17.173296000 |
| C | 3.174682000 | 3.410403000 | 16.150724000 |
| C | 1.006508000 | 3.400751000 | 15.290898000 |
| H | 0.070819000 | 2.931313000 | 14.986639000 |
| C | 2.001577000 | -1.659533000 | 15.435347000 |
| H | 1.979947000 | -2.743844000 | 15.376797000 |
| C | 1.473352000 | -0.892341000 | 14.400385000 |
| H | 1.044645000 | -1.375297000 | 13.527079000 |
| C | 2.563392000 | -1.029361000 | 16.541325000 |
| H | 2.976165000 | -1.622050000 | 17.352685000 |
| C | 0.243634000 | 3.947380000 | 21.153824000 |
| H | -0.845834000 | 3.855184000 | 21.217912000 |
| H | 0.688609000 | 3.564715000 | 22.078844000 |
| H | 0.592893000 | 3.330220000 | 20.321118000 |
| C | 0.128898000 | 6.246791000 | 22.140312000 |
| H | 0.380693000 | 7.307799000 | 22.041311000 |
| H | 0.557095000 | 5.869474000 | 23.073321000 |
| H | -0.956345000 | 6.151242000 | 22.235016000 |
| B | 3.344444000 | 4.960187000 | 15.766736000 |
| H | 6.150356000 | 4.789272000 | 17.438562000 |
| H | 0.728518000 | 6.152669000 | 14.424646000 |

B. r²-SCAN-3c(CPCM: cyclohexane)

Coordinates of compound **6** (CPCM(cyclohexane))

| | | | |
|---|---|---|---|
| Ge | 4.555948000 | 4.279594000 | 4.691283000 |
| Ge | 7.542981000 | 4.489470000 | 7.145749000 |
| Cl | 7.606305000 | 6.721559000 | 7.098373000 |
| O | 4.672961000 | 3.385038000 | 7.579044000 |
| O | 7.526081000 | 6.182418000 | 3.795877000 |
| C | 11.682319000 | 3.359206000 | 6.728719000 |
| H | 12.570244000 | 3.761983000 | 6.249118000 |
| C | 3.367847000 | 3.755320000 | 7.312427000 |
| C | 2.393644000 | 7.316715000 | 2.649991000 |
| H | 2.223497000 | 8.385126000 | 2.751045000 |
| C | 0.812782000 | 4.551503000 | 6.727002000 |
| H | -0.200854000 | 4.864304000 | 6.493155000 |
| C | 5.238769000 | 2.546377000 | 4.159550000 |
| H | 4.771097000 | 1.683604000 | 3.689963000 |
| C | 3.103185000 | 4.194653000 | 6.023859000 |
| C | 5.203014000 | 9.147195000 | 5.746864000 |
| H | 5.118184000 | 9.476631000 | 6.779536000 |
| C | 11.713338000 | 2.097439000 | 7.297734000 |
| H | 12.608048000 | 1.484106000 | 7.234128000 |
| C | 2.426340000 | 3.746211000 | 8.342678000 |
| C | 1.889735000 | 5.221211000 | 1.605724000 |
| H | 1.330023000 | 4.636230000 | 0.879959000 |
| C | 1.580559000 | 6.556716000 | 1.819596000 |
| H | 0.746840000 | 7.017651000 | 1.297166000 |
| C | 10.933131000 | 7.899619000 | 6.437262000 |
| H | 10.981254000 | 8.774399000 | 7.079959000 |
| C | 1.792108000 | 4.588023000 | 5.740110000 |
| H | 1.541821000 | 4.961869000 | 4.750938000 |
| C | 6.147898000 | 9.741736000 | 4.915685000 |
| C | 6.211812000 | 9.318026000 | 3.591765000 |
| H | 6.921650000 | 9.800959000 | 2.924273000 |
| C | 5.380567000 | 8.316323000 | 3.088753000 |
| C | 7.595042000 | 1.718999000 | 4.048171000 |
| C | 2.928270000 | 4.604802000 | 2.312376000 |
| C | 3.883508000 | 0.572591000 | 1.091473000 |
| C | 7.053954000 | 10.845965000 | 5.423303000 |
| H | 7.690284000 | 11.155206000 | 4.582312000 |
| C | 10.528507000 | 4.151053000 | 6.762000000 |
| C | 6.453148000 | 4.358011000 | 8.780147000 |
| C | 4.439757000 | 7.712528000 | 3.954851000 |
| C | 1.131564000 | 4.149621000 | 8.023419000 |
| H | 0.362732000 | 4.166562000 | 8.789185000 |
| C | 3.674754000 | 5.347981000 | 3.256743000 |
| C | 10.753957000 | 6.640985000 | 7.003793000 |
| C | 11.088355000 | 4.502882000 | 3.808865000 |
| H | 10.943450000 | 3.573057000 | 4.362018000 |
| C | 3.468992000 | 6.744501000 | 3.338007000 |
| C | 5.154307000 | 3.877208000 | 8.778045000 |
| C | 5.760360000 | 0.242507000 | 6.914527000 |
| H | 5.449184000 | -0.682485000 | 7.413144000 |
| H | 5.700498000 | 0.066520000 | 5.835102000 |
| H | 5.053955000 | 1.037033000 | 7.165214000 |
| C | 4.336346000 | 8.157048000 | 5.286056000 |
| C | 6.572602000 | 0.519272000 | 9.726753000 |
| H | 5.750800000 | -0.136876000 | 9.453189000 |
| C | 4.723269000 | 1.642254000 | 0.787396000 |

| | | | |
|---|---|---|---|
| H | 5.628817000 | 1.444685000 | 0.220012000 |
| C | 9.362528000 | 3.672384000 | 7.416614000 |
| C | 10.670574000 | 5.512332000 | 6.152856000 |
| C | 6.244658000 | 12.069596000 | 5.873769000 |
| H | 5.595265000 | 12.430748000 | 5.069392000 |
| H | 6.913942000 | 12.884789000 | 6.171390000 |
| H | 5.612219000 | 11.823691000 | 6.734368000 |
| C | 4.299721000 | 3.930930000 | 9.883204000 |
| C | 4.789961000 | 4.539972000 | 11.034678000 |
| H | 4.160648000 | 4.631321000 | 11.914049000 |
| C | 7.291029000 | 0.638855000 | 3.199882000 |
| H | 6.283975000 | 0.528864000 | 2.810176000 |
| C | 7.394528000 | 1.042796000 | 8.727091000 |
| C | 10.604184000 | 1.655521000 | 7.997923000 |
| H | 10.630265000 | 0.699880000 | 8.512636000 |
| C | 12.111872000 | 6.031701000 | 9.024932000 |
| H | 12.869741000 | 6.788585000 | 8.790440000 |
| H | 12.417123000 | 5.087680000 | 8.564978000 |
| H | 12.090815000 | 5.892390000 | 10.112450000 |
| C | 10.880413000 | 5.670206000 | 4.765765000 |
| C | 3.265868000 | 3.192332000 | 1.940947000 |
| C | 2.730226000 | 0.835511000 | 1.822980000 |
| H | 2.061078000 | 0.016591000 | 2.076557000 |
| C | 6.087527000 | 5.049756000 | 11.070192000 |
| H | 6.449933000 | 5.531783000 | 11.973613000 |
| C | 5.351836000 | 1.225443000 | 13.126718000 |
| H | 6.160015000 | 1.640355000 | 13.739253000 |
| H | 4.623659000 | 0.766929000 | 13.805073000 |
| H | 4.868085000 | 2.057725000 | 12.604944000 |
| C | 6.775826000 | 0.802133000 | 11.074207000 |
| C | 6.556505000 | 2.691611000 | 4.434697000 |
| C | 7.846935000 | 1.622010000 | 11.419706000 |
| H | 8.036935000 | 1.833082000 | 12.468603000 |
| C | 2.400768000 | 2.125352000 | 2.246025000 |
| C | 5.391723000 | 4.071140000 | 0.809736000 |
| H | 5.374398000 | 4.812136000 | 1.617216000 |
| C | 3.230957000 | 7.663953000 | 6.195857000 |
| H | 2.777472000 | 6.790840000 | 5.721305000 |
| C | 11.017217000 | 6.958831000 | 4.245856000 |
| H | 11.148337000 | 7.076365000 | 3.173002000 |
| C | 2.906574000 | 3.327444000 | 9.726979000 |
| C | 7.968432000 | 10.350463000 | 6.550042000 |
| H | 7.381556000 | 10.043114000 | 7.423420000 |
| H | 8.654437000 | 11.145349000 | 6.866047000 |
| H | 8.560129000 | 9.487188000 | 6.229000000 |
| C | 7.197048000 | 0.620078000 | 7.281396000 |
| H | 7.489581000 | 1.466115000 | 6.647169000 |
| C | 4.449334000 | 2.944215000 | 1.204151000 |
| C | 5.439922000 | 7.999087000 | 1.598552000 |
| H | 4.886243000 | 7.069891000 | 1.423741000 |
| C | 4.218747000 | -0.829258000 | 0.623404000 |
| H | 5.215384000 | -0.785330000 | 0.161688000 |
| C | 9.444687000 | 2.432491000 | 8.102270000 |
| C | 6.911503000 | 4.954252000 | 9.955928000 |
| H | 7.910205000 | 5.383018000 | 9.985461000 |
| C | 9.579859000 | -0.126958000 | 3.279012000 |
| H | 10.344524000 | -0.839063000 | 2.981896000 |
| C | 5.886368000 | 0.185524000 | 12.134797000 |
| H | 5.026775000 | -0.260680000 | 11.614834000 |
| C | 9.599802000 | 3.947156000 | 12.081868000 |

| | | | |
|---|---|---|---|
| H | 8.738015000 | 4.584382000 | 11.876924000 |
| H | 10.470633000 | 4.588767000 | 12.256398000 |
| H | 9.404837000 | 3.406841000 | 13.014813000 |
| C | 11.180848000 | 9.473755000 | 4.475754000 |
| H | 10.937029000 | 10.188493000 | 5.274258000 |
| C | 7.226429000 | 5.253965000 | 4.397809000 |
| C | 10.300014000 | 7.744103000 | 9.279339000 |
| H | 11.063007000 | 8.529390000 | 9.228400000 |
| H | 10.161922000 | 7.497334000 | 10.338003000 |
| H | 9.358461000 | 8.148920000 | 8.896979000 |
| C | 10.124444000 | 4.479774000 | 2.617767000 |
| H | 9.099151000 | 4.280683000 | 2.934920000 |
| H | 10.409061000 | 3.679270000 | 1.925286000 |
| H | 10.134351000 | 5.426062000 | 2.065669000 |
| C | 8.691660000 | 2.174555000 | 10.459527000 |
| C | 9.897202000 | 2.985499000 | 10.923046000 |
| H | 10.252299000 | 3.584071000 | 10.075479000 |
| C | 8.268540000 | -0.271385000 | 2.824266000 |
| H | 8.008282000 | -1.095189000 | 2.164521000 |
| C | 1.142281000 | 1.464344000 | 4.332166000 |
| H | 1.108436000 | 0.393405000 | 4.102609000 |
| H | 0.272292000 | 1.703560000 | 4.954038000 |
| H | 2.043071000 | 1.660290000 | 4.922918000 |
| C | 1.126697000 | 2.310098000 | 3.050943000 |
| H | 1.065329000 | 3.360544000 | 3.349324000 |
| C | 8.915830000 | 1.851320000 | 4.486168000 |
| H | 9.171084000 | 2.674026000 | 5.144195000 |
| C | 11.024712000 | 8.084677000 | 5.058871000 |
| C | 8.445002000 | 1.905266000 | 9.093918000 |
| C | 10.731968000 | 6.488054000 | 8.518767000 |
| H | 10.015626000 | 5.694451000 | 8.760958000 |
| C | 6.848688000 | 3.636509000 | 0.632762000 |
| H | 7.210475000 | 3.074044000 | 1.500265000 |
| H | 7.479874000 | 4.523887000 | 0.512426000 |
| H | 6.987633000 | 3.017163000 | -0.260506000 |
| C | 1.953735000 | 3.779987000 | 10.832545000 |
| H | 2.328881000 | 3.460854000 | 11.809566000 |
| H | 0.970976000 | 3.317566000 | 10.702714000 |
| H | 1.831078000 | 4.868261000 | 10.842256000 |
| C | 4.744433000 | 9.113506000 | 0.797976000 |
| H | 3.715310000 | 9.270024000 | 1.133379000 |
| H | 4.724582000 | 8.861471000 | -0.268780000 |
| H | 5.285228000 | 10.059915000 | 0.915672000 |
| C | 9.898051000 | 0.941136000 | 4.111161000 |
| H | 10.913682000 | 1.069826000 | 4.476325000 |
| C | 4.877722000 | 4.757389000 | -0.466070000 |
| H | 5.535260000 | 5.586925000 | -0.749863000 |
| H | 3.866833000 | 5.152818000 | -0.325240000 |
| H | 4.852268000 | 4.040184000 | -1.295236000 |
| C | 3.020051000 | 1.784439000 | 9.770711000 |
| H | 3.697901000 | 1.414409000 | 8.997632000 |
| H | 2.033296000 | 1.334426000 | 9.615363000 |
| H | 3.406489000 | 1.466681000 | 10.744941000 |
| C | 12.541596000 | 4.506315000 | 3.302509000 |
| H | 12.728968000 | 5.372972000 | 2.658545000 |
| H | 12.740597000 | 3.600955000 | 2.717635000 |
| H | 13.256011000 | 4.548753000 | 4.131226000 |
| C | 8.122045000 | -0.558339000 | 6.932510000 |
| H | 9.176499000 | -0.300610000 | 7.053101000 |
| H | 7.967636000 | -0.861328000 | 5.890680000 |

| | | | |
|---|---|---|---|
| H | 7.897474000 | -1.413670000 | 7.581319000 |
| C | 6.861360000 | 7.798977000 | 1.055910000 |
| H | 7.466840000 | 8.706190000 | 1.158759000 |
| H | 6.814584000 | 7.560761000 | -0.012632000 |
| H | 7.382911000 | 6.986141000 | 1.565061000 |
| C | 6.624904000 | -0.939824000 | 12.874215000 |
| H | 6.977426000 | -1.704808000 | 12.174669000 |
| H | 5.968031000 | -1.417722000 | 13.610045000 |
| H | 7.496852000 | -0.540278000 | 13.405095000 |
| C | 4.282108000 | -1.828814000 | 1.785499000 |
| H | 4.988256000 | -1.501512000 | 2.555944000 |
| H | 4.599239000 | -2.813699000 | 1.425073000 |
| H | 3.300591000 | -1.947577000 | 2.257664000 |
| C | 10.212709000 | 9.718098000 | 3.312427000 |
| H | 10.485148000 | 9.118939000 | 2.436170000 |
| H | 10.230596000 | 10.771816000 | 3.011937000 |
| H | 9.189330000 | 9.451513000 | 3.594741000 |
| C | 3.726545000 | 7.234819000 | 7.580133000 |
| H | 4.546178000 | 6.513129000 | 7.509082000 |
| H | 2.907492000 | 6.770464000 | 8.141179000 |
| H | 4.088705000 | 8.089778000 | 8.162292000 |
| C | 3.223083000 | -1.301878000 | -0.445254000 |
| H | 3.503766000 | -2.290436000 | -0.825775000 |
| H | 3.188591000 | -0.603229000 | -1.287532000 |
| H | 2.213187000 | -1.374855000 | -0.025359000 |
| C | 2.130718000 | 8.728614000 | 6.322819000 |
| H | 2.520041000 | 9.632174000 | 6.806707000 |
| H | 1.299994000 | 8.346252000 | 6.927907000 |
| H | 1.739592000 | 9.012968000 | 5.339969000 |
| C | -0.116992000 | 1.997650000 | 2.207291000 |
| H | -0.152195000 | 2.620450000 | 1.307161000 |
| H | -1.028186000 | 2.179565000 | 2.788363000 |
| H | -0.123587000 | 0.948579000 | 1.889927000 |
| C | 12.632008000 | 9.726161000 | 4.042038000 |
| H | 13.323171000 | 9.589896000 | 4.880355000 |
| H | 12.750613000 | 10.745505000 | 3.656871000 |
| H | 12.921100000 | 9.026580000 | 3.248880000 |
| C | 11.039655000 | 2.046963000 | 11.349474000 |
| H | 10.718531000 | 1.425086000 | 12.193711000 |
| H | 11.912325000 | 2.629862000 | 11.666735000 |
| H | 11.349130000 | 1.382676000 | 10.539272000 |
| B | 6.740703000 | 4.109900000 | 5.205854000 |

Coordinates of compound **7** (CPCM(cyclohexane))

| | | | |
|---|---|---|---|
| Ge | -4.821747000 | -4.797854000 | -17.080868000 |
| Ge | -1.227586000 | -5.363512000 | -15.437158000 |
| Cl | -4.071820000 | -5.159993000 | -13.838014000 |
| O | -2.089057000 | -4.864621000 | -18.244445000 |
| O | -4.046241000 | -2.958170000 | -16.755262000 |
| C | -5.747789000 | 0.466355000 | -16.342228000 |
| C | -6.906756000 | -9.845268000 | -16.174719000 |
| C | -0.078449000 | -1.857918000 | -11.856566000 |
| H | -0.668617000 | -1.082266000 | -11.371879000 |
| C | -0.615816000 | -3.139523000 | -11.986031000 |
| C | -7.014661000 | -8.182927000 | -17.947663000 |
| C | -2.001612000 | -3.398857000 | -11.424456000 |
| H | -2.313806000 | -4.398053000 | -11.745688000 |
| C | -0.290266000 | -5.578076000 | -12.639978000 |
| C | -6.867789000 | -9.503884000 | -17.517860000 |
| H | -6.712245000 | -10.278531000 | -18.263147000 |
| C | -4.310272000 | -4.937334000 | -18.980751000 |
| C | -6.825966000 | -1.638441000 | -15.760160000 |
| C | -6.803421000 | -4.612430000 | -17.252057000 |
| C | -8.687241000 | -3.276499000 | -18.012830000 |
| H | -9.119185000 | -2.294939000 | -18.190528000 |
| C | -6.312076000 | -0.393920000 | -15.403249000 |
| H | -6.365278000 | -0.084155000 | -14.363686000 |
| C | -5.223844000 | 1.830968000 | -15.945559000 |
| H | -4.552762000 | 2.162249000 | -16.750208000 |
| C | 0.285790000 | -11.065579000 | -18.442575000 |
| H | 1.355780000 | -11.316069000 | -18.464251000 |
| C | -1.992314000 | -3.368982000 | -9.888148000 |
| H | -1.707950000 | -2.375967000 | -9.520459000 |
| H | -2.990469000 | -3.599852000 | -9.498837000 |
| H | -1.287041000 | -4.094705000 | -9.471885000 |
| C | -5.218874000 | -4.981011000 | -20.038645000 |
| H | -6.282173000 | -4.888191000 | -19.838662000 |
| C | -1.775509000 | -1.164726000 | -15.944838000 |
| C | -3.413781000 | -5.276380000 | -21.614885000 |
| H | -3.092578000 | -5.414480000 | -22.642405000 |
| C | -0.382925000 | -8.605708000 | -14.939225000 |
| C | -0.226179000 | -6.239724000 | -11.408368000 |
| H | 0.002796000 | -5.662573000 | -10.516890000 |
| C | -0.556834000 | -6.312445000 | -13.821474000 |
| C | -0.418089000 | -7.610643000 | -11.319475000 |
| H | -0.404723000 | -8.106121000 | -10.352630000 |
| C | -1.027904000 | -0.385561000 | -15.052527000 |
| H | -0.636397000 | -0.845746000 | -14.149532000 |
| C | -1.455429000 | -8.950533000 | -15.778051000 |
| C | -7.089404000 | -8.820615000 | -15.245441000 |
| H | -7.117058000 | -9.086291000 | -14.192369000 |
| C | 1.183549000 | -1.527700000 | -12.338910000 |
| C | 1.437380000 | -3.827058000 | -13.107641000 |
| C | -7.584895000 | -5.774739000 | -17.430753000 |
| C | 0.970063000 | -6.364037000 | -19.556393000 |
| H | 1.473109000 | -6.584975000 | -20.492352000 |
| C | -7.393684000 | -3.349041000 | -17.488080000 |
| C | -4.774670000 | -5.149227000 | -21.345813000 |
| H | -5.490598000 | -5.188003000 | -22.161870000 |
| C | -5.719820000 | 0.056665000 | -17.671236000 |
| H | -5.285243000 | 0.724665000 | -18.413076000 |

| | | | |
|---|---|---|---|
| C | 0.904764000 | -9.121962000 | -15.204981000 |
| C | -8.877220000 | -5.659881000 | -17.961368000 |
| H | -9.468263000 | -6.563642000 | -18.084560000 |
| C | -7.197878000 | -7.158049000 | -16.998096000 |
| C | -2.465579000 | -5.200712000 | -20.593121000 |
| C | -0.539798000 | -8.350297000 | -12.487231000 |
| H | -0.584842000 | -9.434966000 | -12.445146000 |
| C | 0.978100000 | -6.420028000 | -17.141863000 |
| H | 1.464552000 | -6.691166000 | -16.211771000 |
| C | 1.593170000 | -6.703062000 | -18.357621000 |
| H | 2.563067000 | -7.192710000 | -18.373272000 |
| C | -0.274567000 | -5.731222000 | -19.564362000 |
| C | 0.046615000 | -10.232489000 | -17.200252000 |
| C | -1.223273000 | -9.748883000 | -16.896517000 |
| H | -2.055749000 | -10.001568000 | -17.547058000 |
| C | -0.861230000 | -5.489591000 | -18.323274000 |
| C | -7.480253000 | -6.434685000 | -14.543261000 |
| H | -6.906153000 | -5.541802000 | -14.819876000 |
| C | 1.088918000 | -9.924239000 | -16.333039000 |
| H | 2.080021000 | -10.314994000 | -16.552208000 |
| C | -0.956591000 | -5.185018000 | -20.815890000 |
| C | -9.415595000 | -4.425505000 | -18.287170000 |
| H | -10.415641000 | -4.356371000 | -18.706127000 |
| C | -2.297171000 | -0.560474000 | -17.095628000 |
| H | -2.885091000 | -1.143442000 | -17.793650000 |
| C | -6.795031000 | -11.277833000 | -15.692722000 |
| H | -6.306001000 | -11.244460000 | -14.708193000 |
| C | -2.954130000 | -5.015706000 | -19.300613000 |
| C | 2.363930000 | -4.877910000 | -13.700258000 |
| H | 1.753946000 | -5.719556000 | -14.040646000 |
| C | -1.919188000 | -2.617618000 | -15.694972000 |
| C | -6.960054000 | -7.920988000 | -19.442600000 |
| H | -7.061371000 | -6.843944000 | -19.599463000 |
| C | 2.096220000 | -8.872487000 | -14.288416000 |
| H | 1.846947000 | -8.037212000 | -13.623876000 |
| C | -4.722941000 | -1.506370000 | -20.098629000 |
| H | -4.057333000 | -2.153531000 | -19.519652000 |
| H | -4.707352000 | -1.853327000 | -21.138191000 |
| H | -4.320556000 | -0.487006000 | -20.077626000 |
| C | -6.216408000 | -1.183688000 | -18.075995000 |
| C | -0.560198000 | -7.726266000 | -13.737560000 |
| C | -6.153616000 | -1.556650000 | -19.546656000 |
| H | -6.505981000 | -2.588742000 | -19.647318000 |
| C | -0.278535000 | -5.822782000 | -17.102724000 |
| C | -6.761333000 | -2.045538000 | -17.109395000 |
| C | -3.129121000 | -3.378538000 | -15.963519000 |
| C | -0.873868000 | -3.410901000 | -15.328607000 |
| H | 0.109657000 | -2.962197000 | -15.198544000 |
| C | -3.032757000 | -2.394570000 | -11.958180000 |
| H | -2.838128000 | -1.382936000 | -11.584397000 |
| H | -3.030826000 | -2.362725000 | -13.051837000 |
| H | -4.037251000 | -2.682821000 | -11.630755000 |
| C | 0.143514000 | -4.141939000 | -12.625576000 |
| C | 1.925172000 | -2.529313000 | -12.958958000 |
| H | 2.919566000 | -2.300408000 | -13.328952000 |
| C | -7.233821000 | -7.486787000 | -15.617002000 |
| C | -7.010005000 | -6.857468000 | -13.148525000 |
| H | -7.673879000 | -7.613193000 | -12.712174000 |
| H | -5.990616000 | -7.253153000 | -13.164085000 |
| H | -7.021935000 | -5.987757000 | -12.483370000 |

| | | | |
|---|---|---|---|
| C | -1.298052000 | 1.549123000 | -16.467404000 |
| H | -1.112887000 | 2.600015000 | -16.672032000 |
| C | -0.794507000 | 0.960173000 | -15.309869000 |
| H | -0.219717000 | 1.551406000 | -14.601947000 |
| C | -2.048155000 | 0.783152000 | -17.354996000 |
| H | -2.445047000 | 1.232645000 | -18.261501000 |
| C | -3.525029000 | -9.618936000 | -14.557970000 |
| H | -4.540311000 | -9.316183000 | -14.280194000 |
| H | -3.587066000 | -10.566400000 | -15.106962000 |
| H | -2.948916000 | -9.791461000 | -13.642817000 |
| C | -7.527553000 | -2.480349000 | -14.707392000 |
| H | -7.560023000 | -3.512931000 | -15.068239000 |
| C | -2.867871000 | -8.536805000 | -15.429044000 |
| H | -2.819718000 | -7.626527000 | -14.819123000 |
| C | -4.414013000 | 1.798031000 | -14.645657000 |
| H | -3.645735000 | 1.019735000 | -14.680800000 |
| H | -3.919276000 | 2.761878000 | -14.482215000 |
| H | -5.057802000 | 1.607379000 | -13.779479000 |
| C | -0.525277000 | -3.705039000 | -20.976345000 |
| H | 0.560782000 | -3.643094000 | -21.106704000 |
| H | -1.016110000 | -3.262847000 | -21.850635000 |
| H | -0.801010000 | -3.121246000 | -20.091866000 |
| C | -0.037883000 | -10.267567000 | -19.713401000 |
| H | 0.529263000 | -9.331234000 | -19.743042000 |
| H | 0.202842000 | -10.852314000 | -20.608595000 |
| H | -1.103349000 | -10.012605000 | -19.752973000 |
| C | -0.556581000 | -5.964250000 | -22.071567000 |
| H | -0.842231000 | -7.018691000 | -21.993608000 |
| H | -1.028854000 | -5.536626000 | -22.959808000 |
| H | 0.522690000 | -5.904078000 | -22.234203000 |
| C | 2.357367000 | -10.108519000 | -13.411489000 |
| H | 1.471373000 | -10.388497000 | -12.835323000 |
| H | 2.635726000 | -10.964585000 | -14.037494000 |
| H | 3.178287000 | -9.915462000 | -12.711208000 |
| C | -8.966012000 | -6.042468000 | -14.475771000 |
| H | -9.125916000 | -5.316579000 | -13.669867000 |
| H | -9.318045000 | -5.596218000 | -15.409316000 |
| H | -9.579111000 | -6.926884000 | -14.265148000 |
| C | -8.119525000 | -8.606461000 | -20.179607000 |
| H | -9.088895000 | -8.294649000 | -19.777550000 |
| H | -8.092448000 | -8.355369000 | -21.246090000 |
| H | -8.054160000 | -9.696714000 | -20.090092000 |
| C | -5.613388000 | -8.357256000 | -20.037501000 |
| H | -5.476059000 | -9.441269000 | -19.953431000 |
| H | -5.564286000 | -8.091947000 | -21.099302000 |
| H | -4.778571000 | -7.866303000 | -19.528043000 |
| C | 1.724685000 | -0.124927000 | -12.148436000 |
| H | 0.854297000 | 0.540989000 | -12.053114000 |
| C | 3.385837000 | -8.506204000 | -15.036444000 |
| H | 4.172049000 | -8.260862000 | -14.314221000 |
| H | 3.752952000 | -9.339451000 | -15.644707000 |
| H | 3.252495000 | -7.642449000 | -15.694317000 |
| C | 3.302846000 | -5.403867000 | -12.603266000 |
| H | 2.731674000 | -5.827293000 | -11.770064000 |
| H | 3.964349000 | -6.182057000 | -13.000522000 |
| H | 3.925045000 | -4.590202000 | -12.211709000 |
| C | -7.086797000 | -0.666219000 | -20.379890000 |
| H | -6.767699000 | 0.381538000 | -20.336699000 |
| H | -7.079223000 | -0.979144000 | -21.430389000 |
| H | -8.116839000 | -0.718146000 | -20.011619000 |

| | | | |
|---|---|---|---|
| C | -6.378273000 | 2.839430000 | -15.843170000 |
| H | -6.000563000 | 3.838659000 | -15.597000000 |
| H | -6.934574000 | 2.901216000 | -16.784496000 |
| H | -7.078923000 | 2.536192000 | -15.056087000 |
| C | -3.715709000 | -8.235570000 | -16.662152000 |
| H | -4.665501000 | -7.785552000 | -16.362303000 |
| H | -3.196501000 | -7.546831000 | -17.338723000 |
| H | -3.961072000 | -9.144457000 | -17.220921000 |
| C | 2.523698000 | -0.038489000 | -10.838326000 |
| H | 1.912793000 | -0.343525000 | -9.982476000 |
| H | 3.397466000 | -0.699459000 | -10.882912000 |
| H | 2.877230000 | 0.984488000 | -10.665560000 |
| C | -8.197588000 | -11.876673000 | -15.498231000 |
| H | -8.791928000 | -11.274776000 | -14.803165000 |
| H | -8.729798000 | -11.911263000 | -16.456260000 |
| H | -8.133053000 | -12.897497000 | -15.104552000 |
| C | -5.952260000 | -12.171969000 | -16.604451000 |
| H | -5.787400000 | -13.144153000 | -16.128209000 |
| H | -6.454864000 | -12.358281000 | -17.560365000 |
| H | -4.975270000 | -11.723665000 | -16.815040000 |
| C | 3.164110000 | -4.380286000 | -14.908877000 |
| H | 3.907895000 | -3.626840000 | -14.628334000 |
| H | 3.707317000 | -5.217294000 | -15.361249000 |
| H | 2.510123000 | -3.948912000 | -15.674655000 |
| C | -0.505046000 | -12.380021000 | -18.404907000 |
| H | -1.584125000 | -12.187501000 | -18.408531000 |
| H | -0.271786000 | -12.995502000 | -19.281141000 |
| H | -0.268796000 | -12.956611000 | -17.504460000 |
| C | -6.808150000 | -2.490370000 | -13.355800000 |
| H | -7.271294000 | -3.231469000 | -12.694205000 |
| H | -5.752278000 | -2.751705000 | -13.470715000 |
| H | -6.871162000 | -1.519191000 | -12.852059000 |
| C | 2.568348000 | 0.366241000 | -13.328527000 |
| H | 3.520599000 | -0.172021000 | -13.390138000 |
| H | 2.041464000 | 0.233536000 | -14.279249000 |
| H | 2.802097000 | 1.429539000 | -13.207994000 |
| B | -3.309337000 | -4.910389000 | -15.516660000 |
| C | -8.980891000 | -2.007137000 | -14.544670000 |
| H | -9.006556000 | -0.967007000 | -14.198559000 |
| H | -9.522984000 | -2.063910000 | -15.494404000 |
| H | -9.507448000 | -2.627750000 | -13.810301000 |

Coordinates of compound **6-Terph** (CPCM(cyclohexane))

| | | | |
|---|---|---|---|
| Ge | 4.433908000 | 4.186417000 | 4.769282000 |
| Ge | 7.573798000 | 4.583755000 | 6.936728000 |
| Cl | 7.676405000 | 6.809268000 | 6.874125000 |
| O | 4.750482000 | 3.512235000 | 7.630875000 |
| O | 7.299268000 | 5.786172000 | 3.184323000 |
| C | 11.840006000 | 4.215479000 | 7.455324000 |
| H | 12.726613000 | 4.776790000 | 7.172627000 |
| C | 3.444299000 | 3.917967000 | 7.468506000 |
| C | 3.960774000 | 7.182610000 | 1.752434000 |
| H | 4.128724000 | 8.252312000 | 1.663896000 |
| C | 0.873842000 | 4.766900000 | 7.054917000 |
| H | -0.146006000 | 5.104049000 | 6.894546000 |
| C | 5.117604000 | 2.419952000 | 4.328247000 |
| H | 4.647767000 | 1.508782000 | 3.961556000 |
| C | 3.074798000 | 4.276356000 | 6.180268000 |
| C | 4.201674000 | 8.358781000 | 6.349885000 |
| H | 3.643535000 | 8.356509000 | 7.282051000 |
| C | 11.948971000 | 3.106958000 | 8.280343000 |
| H | 12.920518000 | 2.795414000 | 8.653501000 |
| C | 2.596867000 | 3.989635000 | 8.574721000 |
| C | 3.318133000 | 5.090876000 | 0.771603000 |
| H | 2.984457000 | 4.509501000 | -0.083912000 |
| C | 3.569951000 | 6.448894000 | 0.642663000 |
| H | 3.444114000 | 6.939378000 | -0.318484000 |
| C | 11.195205000 | 8.156660000 | 5.750738000 |
| H | 11.569453000 | 9.092923000 | 6.155449000 |
| C | 1.757122000 | 4.697104000 | 5.981500000 |
| H | 1.427866000 | 5.010024000 | 4.992672000 |
| C | 5.251974000 | 9.250511000 | 6.163996000 |
| C | 5.951118000 | 9.246255000 | 4.959573000 |
| H | 6.783905000 | 9.927849000 | 4.810514000 |
| C | 5.601948000 | 8.358481000 | 3.950316000 |
| C | 7.520820000 | 1.741690000 | 4.001306000 |
| C | 3.461154000 | 4.456877000 | 2.010334000 |
| C | 2.588432000 | 0.261630000 | 2.188468000 |
| C | 10.598396000 | 4.626685000 | 6.952754000 |
| C | 6.660076000 | 4.415047000 | 8.672918000 |
| C | 4.546216000 | 7.452144000 | 4.125153000 |
| C | 1.293943000 | 4.426448000 | 8.341536000 |
| H | 0.592062000 | 4.507982000 | 9.165320000 |
| C | 3.883013000 | 5.187107000 | 3.143502000 |
| C | 11.130517000 | 7.031967000 | 6.562880000 |
| C | 4.124121000 | 6.573657000 | 3.004268000 |
| C | 5.350871000 | 3.977279000 | 8.785740000 |
| C | 3.849640000 | 7.470529000 | 5.338343000 |
| C | 6.463560000 | 0.597604000 | 7.883551000 |
| H | 5.798410000 | 0.224387000 | 7.109514000 |
| C | 3.538095000 | 0.744529000 | 1.290556000 |
| H | 4.072403000 | 0.057223000 | 0.640315000 |
| C | 9.426486000 | 3.919629000 | 7.302130000 |
| C | 10.625671000 | 5.823230000 | 6.070397000 |
| C | 4.609749000 | 4.028163000 | 9.968782000 |
| C | 5.261568000 | 4.509571000 | 11.101764000 |
| H | 4.733764000 | 4.575002000 | 12.047701000 |
| C | 7.234206000 | 0.573611000 | 3.273763000 |
| H | 6.204207000 | 0.330608000 | 3.028234000 |
| C | 7.530245000 | 1.418400000 | 7.531065000 |

| | | | |
|---|---|---|---|
| C | 10.810911000 | 2.385029000 | 8.603475000 |
| H | 10.884851000 | 1.492167000 | 9.218401000 |
| C | 10.227844000 | 5.758148000 | 4.734320000 |
| C | 3.153056000 | 3.006182000 | 2.073576000 |
| C | 1.909776000 | 1.151872000 | 3.017384000 |
| H | 1.158106000 | 0.786792000 | 3.711594000 |
| C | 6.594364000 | 4.917917000 | 11.036911000 |
| H | 7.087027000 | 5.286932000 | 11.931809000 |
| C | 6.257952000 | 0.258968000 | 9.215956000 |
| C | 6.446538000 | 2.645548000 | 4.449404000 |
| C | 7.125411000 | 0.747203000 | 10.192916000 |
| H | 6.964138000 | 0.495576000 | 11.237588000 |
| C | 2.186379000 | 2.513717000 | 2.955947000 |
| C | 10.300703000 | 6.882215000 | 3.916479000 |
| H | 9.986260000 | 6.812524000 | 2.879267000 |
| C | 3.160871000 | 3.547398000 | 9.921710000 |
| C | 3.816009000 | 2.106144000 | 1.231018000 |
| C | 9.554913000 | 2.770786000 | 8.121095000 |
| C | 7.285129000 | 4.881850000 | 9.831395000 |
| H | 8.312334000 | 5.237120000 | 9.785735000 |
| C | 9.586008000 | 0.037620000 | 3.132678000 |
| H | 10.380598000 | -0.622339000 | 2.796660000 |
| C | 7.059670000 | 5.084196000 | 4.059337000 |
| C | 8.192392000 | 1.562213000 | 9.839102000 |
| C | 8.253879000 | -0.266022000 | 2.848727000 |
| H | 8.010868000 | -1.163012000 | 2.285610000 |
| C | 8.864359000 | 2.034868000 | 4.268709000 |
| H | 9.110175000 | 2.919809000 | 4.847352000 |
| C | 10.774771000 | 8.087055000 | 4.423931000 |
| C | 8.403363000 | 1.916393000 | 8.500366000 |
| C | 2.337431000 | 4.077135000 | 11.095363000 |
| H | 2.753068000 | 3.723868000 | 12.043241000 |
| H | 1.311094000 | 3.702899000 | 11.041471000 |
| H | 2.312917000 | 5.171872000 | 11.109599000 |
| C | 9.887267000 | 1.193738000 | 3.845141000 |
| H | 10.918901000 | 1.443950000 | 4.076495000 |
| C | 3.151303000 | 1.999378000 | 9.958280000 |
| H | 3.722613000 | 1.585487000 | 9.123253000 |
| H | 2.121060000 | 1.631601000 | 9.897497000 |
| H | 3.603502000 | 1.646171000 | 10.891727000 |
| B | 6.635893000 | 4.127233000 | 5.106487000 |
| H | 6.162953000 | 8.350765000 | 3.019930000 |
| H | 4.575324000 | 2.477878000 | 0.547642000 |
| H | 2.374720000 | -0.802260000 | 2.238055000 |
| H | 1.642947000 | 3.203535000 | 3.594734000 |
| H | 5.427242000 | -0.381971000 | 9.496863000 |
| H | 7.709048000 | 1.641565000 | 6.485611000 |
| H | 8.850554000 | 1.960364000 | 10.606797000 |
| H | 11.441610000 | 7.091490000 | 7.602689000 |
| H | 10.825122000 | 8.965903000 | 3.787399000 |
| H | 5.531846000 | 9.939599000 | 6.955566000 |
| H | 2.997142000 | 6.816423000 | 5.477723000 |
| H | 9.882592000 | 4.816262000 | 4.318047000 |

Coordinates of compound **7-Terph** (CPCM(cyclohexane))

| | | | |
|---|---|---|---|
| Ge | 4.600389000 | 4.806241000 | 17.189343000 |
| Ge | 1.235133000 | 5.053159000 | 15.303120000 |
| Cl | 4.140945000 | 4.732660000 | 13.869799000 |
| O | 1.927233000 | 5.064143000 | 18.232144000 |
| O | 3.703900000 | 2.968051000 | 17.226747000 |
| C | 5.336753000 | -0.322791000 | 15.961726000 |
| C | 5.999500000 | 9.590170000 | 16.338515000 |
| C | 1.118130000 | 2.362953000 | 11.663133000 |
| H | 2.059428000 | 1.857819000 | 11.463590000 |
| C | 1.126002000 | 3.697060000 | 12.058595000 |
| C | 6.946819000 | 8.130066000 | 18.012642000 |
| C | -0.074698000 | 5.801073000 | 12.709762000 |
| C | 6.544359000 | 9.396950000 | 17.607266000 |
| H | 6.656505000 | 10.239382000 | 18.284023000 |
| C | 4.099279000 | 5.465858000 | 18.969983000 |
| C | 6.041878000 | 1.956132000 | 15.615281000 |
| C | 6.559784000 | 4.528479000 | 17.277020000 |
| C | 8.483854000 | 3.183266000 | 17.882682000 |
| H | 8.949029000 | 2.204892000 | 17.970562000 |
| C | 5.452962000 | 0.792043000 | 15.136045000 |
| H | 5.080433000 | 0.757370000 | 14.115789000 |
| C | 4.972347000 | 5.907372000 | 19.963261000 |
| H | 6.046507000 | 5.838717000 | 19.813221000 |
| C | 1.850240000 | 0.913430000 | 15.909787000 |
| C | 3.093147000 | 6.527951000 | 21.354696000 |
| H | 2.731557000 | 6.942675000 | 22.290429000 |
| C | 1.024645000 | 8.175987000 | 15.467141000 |
| C | -0.738822000 | 6.700708000 | 11.866501000 |
| H | -1.203155000 | 6.325524000 | 10.958062000 |
| C | 0.535924000 | 6.259707000 | 13.892196000 |
| C | -0.791450000 | 8.053112000 | 12.172977000 |
| H | -1.296481000 | 8.744712000 | 11.504365000 |
| C | 1.462439000 | 0.079403000 | 14.854122000 |
| H | 1.236193000 | 0.520312000 | 13.886460000 |
| C | 2.351358000 | 7.944615000 | 15.843973000 |
| C | 5.842875000 | 8.504905000 | 15.483034000 |
| H | 5.421509000 | 8.645094000 | 14.491742000 |
| C | -0.087332000 | 1.679327000 | 11.525582000 |
| C | -1.280016000 | 3.678604000 | 12.162987000 |
| C | 7.326686000 | 5.689321000 | 17.506297000 |
| C | -1.353704000 | 6.413055000 | 19.077405000 |
| H | -1.933134000 | 6.779087000 | 19.919071000 |
| C | 7.173320000 | 3.267143000 | 17.396303000 |
| C | 4.469279000 | 6.445763000 | 21.144844000 |
| H | 5.150622000 | 6.797637000 | 21.913863000 |
| C | 5.819392000 | -0.268378000 | 17.266747000 |
| H | 5.723165000 | -1.133288000 | 17.917340000 |
| C | 0.191408000 | 8.885018000 | 16.341688000 |
| C | 8.637498000 | 5.579686000 | 17.981743000 |
| H | 9.222988000 | 6.481069000 | 18.142649000 |
| C | 6.801094000 | 7.030350000 | 17.156754000 |
| C | 2.183592000 | 6.064522000 | 20.401998000 |
| C | -0.189593000 | 8.518863000 | 13.334337000 |
| H | -0.220275000 | 9.577030000 | 13.581133000 |
| C | -1.236288000 | 5.894520000 | 16.719220000 |
| H | -1.703623000 | 5.887720000 | 15.736536000 |
| C | -1.958642000 | 6.336164000 | 17.824986000 |

| | | | |
|---|---|---|---|
| H | -2.995131000 | 6.640352000 | 17.709849000 |
| C | -0.028488000 | 6.014541000 | 19.262981000 |
| C | 1.964446000 | 9.019593000 | 17.969911000 |
| C | 2.812745000 | 8.353215000 | 17.092240000 |
| H | 3.839956000 | 8.152213000 | 17.378943000 |
| C | 0.640325000 | 5.542370000 | 18.135738000 |
| C | 0.655824000 | 9.300988000 | 17.583123000 |
| H | -0.013477000 | 9.825236000 | 18.259834000 |
| C | 0.673588000 | 5.989357000 | 20.619365000 |
| C | 9.199761000 | 4.329102000 | 18.203242000 |
| H | 10.217845000 | 4.248282000 | 18.573494000 |
| C | 2.155669000 | 0.342823000 | 17.150917000 |
| H | 2.461203000 | 0.978180000 | 17.974934000 |
| C | 2.729892000 | 5.549322000 | 19.228679000 |
| C | 1.915843000 | 2.374676000 | 15.693064000 |
| C | 6.410375000 | 0.895563000 | 17.747839000 |
| C | 0.470781000 | 7.639135000 | 14.197909000 |
| C | 0.095257000 | 5.505657000 | 16.857384000 |
| C | 6.515302000 | 2.024453000 | 16.929922000 |
| C | 3.026097000 | 3.204122000 | 16.160871000 |
| C | 0.941934000 | 3.103592000 | 15.079445000 |
| H | 0.045330000 | 2.608419000 | 14.707505000 |
| C | -0.072691000 | 4.368616000 | 12.315090000 |
| C | -1.287017000 | 2.341110000 | 11.778150000 |
| H | -2.231761000 | 1.814500000 | 11.675486000 |
| C | 6.234950000 | 7.232559000 | 15.889084000 |
| C | 1.680371000 | -1.858056000 | 16.273949000 |
| H | 1.615542000 | -2.933152000 | 16.416505000 |
| C | 1.382264000 | -1.296595000 | 15.033967000 |
| H | 1.088134000 | -1.932402000 | 14.203364000 |
| C | 2.061292000 | -1.033198000 | 17.328947000 |
| H | 2.292970000 | -1.463844000 | 18.299308000 |
| C | 0.362324000 | 4.630585000 | 21.294949000 |
| H | -0.717650000 | 4.526382000 | 21.447546000 |
| H | 0.867495000 | 4.568338000 | 22.265332000 |
| H | 0.705380000 | 3.798951000 | 20.671117000 |
| C | 0.181812000 | 7.126049000 | 21.522998000 |
| H | 0.390543000 | 8.103139000 | 21.074620000 |
| H | 0.661868000 | 7.078642000 | 22.503951000 |
| H | -0.893652000 | 7.043727000 | 21.698632000 |
| B | 3.301775000 | 4.641911000 | 15.538657000 |
| H | 5.690922000 | 10.582633000 | 16.023182000 |
| H | 6.156186000 | 6.397010000 | 15.197569000 |
| H | 7.371913000 | 7.984948000 | 19.002384000 |
| H | 6.772912000 | 0.941347000 | 18.771859000 |
| H | 6.136725000 | 2.824540000 | 14.968283000 |
| H | 4.858239000 | -1.226187000 | 15.594268000 |
| H | 2.066505000 | 4.228554000 | 12.163690000 |
| H | -0.093315000 | 0.636381000 | 11.220777000 |
| H | -2.216484000 | 4.193172000 | 12.364209000 |
| H | 3.025939000 | 7.442886000 | 15.155690000 |
| H | 2.324710000 | 9.320042000 | 18.950163000 |
| H | -0.843340000 | 9.063278000 | 16.061375000 |

Coordinates of compound **6-$C_6H_5$** (CPCM(cyclohexane))

| | | | |
|---|---|---|---|
| Ge | -4.130056000 | -4.202437000 | -4.702955000 |
| Ge | -7.320401000 | -4.545256000 | -6.851007000 |
| Cl | -7.614876000 | -6.753002000 | -6.960581000 |
| O | -4.552616000 | -3.393507000 | -7.513702000 |
| O | -6.972521000 | -6.071721000 | -3.357702000 |
| C | -11.454125000 | -3.862541000 | -6.168001000 |
| H | -12.276406000 | -4.379226000 | -5.680202000 |
| C | -3.250954000 | -3.839446000 | -7.427101000 |
| C | -2.639570000 | -7.390962000 | -2.347503000 |
| H | -2.286884000 | -8.403729000 | -2.522594000 |
| C | -0.696182000 | -4.777811000 | -7.164789000 |
| H | 0.319490000 | -5.149536000 | -7.066536000 |
| C | -4.840560000 | -2.458035000 | -4.216578000 |
| H | -4.428752000 | -1.534390000 | -3.809993000 |
| C | -2.848631000 | -4.296242000 | -6.178434000 |
| C | -11.658406000 | -2.608148000 | -6.738844000 |
| H | -12.639352000 | -2.142852000 | -6.693289000 |
| C | -2.440956000 | -3.831880000 | -8.560609000 |
| C | -3.157316000 | -5.590623000 | -0.829410000 |
| H | -3.206403000 | -5.195140000 | 0.181506000 |
| C | -2.695916000 | -6.885679000 | -1.050422000 |
| H | -2.384680000 | -7.503021000 | -0.212263000 |
| C | -1.536865000 | -4.761088000 | -6.054227000 |
| H | -1.174910000 | -5.122672000 | -5.095117000 |
| C | -7.250560000 | -1.722683000 | -4.341504000 |
| C | -3.559097000 | -4.801049000 | -1.904213000 |
| C | -10.196849000 | -4.457899000 | -6.223528000 |
| C | -6.516993000 | -4.117773000 | -8.587103000 |
| C | -1.145095000 | -4.323979000 | -8.405267000 |
| H | -0.468435000 | -4.353016000 | -9.253441000 |
| C | -3.496125000 | -5.293354000 | -3.212239000 |
| C | -3.038779000 | -6.600142000 | -3.421418000 |
| C | -5.194391000 | -3.711489000 | -8.692905000 |
| C | -9.133648000 | -3.807726000 | -6.855846000 |
| C | -4.490865000 | -3.648827000 | -9.896539000 |
| C | -5.201363000 | -3.955806000 | -11.055468000 |
| H | -4.705950000 | -3.926053000 | -12.020643000 |
| C | -7.047392000 | -0.351818000 | -4.558261000 |
| H | -6.066082000 | -0.001656000 | -4.867360000 |
| C | -10.605098000 | -1.953155000 | -7.370921000 |
| H | -10.760511000 | -0.975061000 | -7.818035000 |
| C | -6.551973000 | -4.302205000 | -10.996992000 |
| H | -7.088628000 | -4.525740000 | -11.914163000 |
| C | -6.146874000 | -2.683346000 | -4.492146000 |
| C | -3.022814000 | -3.233854000 | -9.839708000 |
| C | -9.349009000 | -2.551021000 | -7.430161000 |
| C | -7.204811000 | -4.394452000 | -9.772382000 |
| H | -8.243852000 | -4.713668000 | -9.734372000 |
| C | -9.359955000 | 0.100246000 | -4.035264000 |
| H | -10.178159000 | 0.806071000 | -3.923591000 |
| C | -6.728759000 | -5.222143000 | -4.090957000 |
| C | -8.094108000 | 0.549570000 | -4.408416000 |
| H | -7.924975000 | 1.607176000 | -4.591360000 |
| C | -8.531276000 | -2.162003000 | -3.983299000 |
| H | -8.709869000 | -3.223282000 | -3.831223000 |
| C | -2.250188000 | -3.670135000 | -11.084319000 |
| H | -2.671430000 | -3.203935000 | -11.979384000 |

| | | | |
|---|---|---|---|
| H | -1.208427000 | -3.343752000 | -11.019210000 |
| H | -2.271186000 | -4.757604000 | -11.211007000 |
| C | -9.573716000 | -1.258722000 | -3.819071000 |
| H | -10.559224000 | -1.619010000 | -3.538489000 |
| C | -2.954667000 | -1.690132000 | -9.720229000 |
| H | -3.502220000 | -1.338159000 | -8.841058000 |
| H | -1.910856000 | -1.370345000 | -9.630874000 |
| H | -3.393531000 | -1.228093000 | -10.611151000 |
| B | -6.302974000 | -4.193628000 | -5.059430000 |
| H | -3.925341000 | -3.792596000 | -1.721772000 |
| H | -2.994017000 | -7.008724000 | -4.429250000 |
| H | -10.047550000 | -5.438987000 | -5.777962000 |
| H | -8.532801000 | -2.030106000 | -7.927129000 |

Coordinates of compound **7-$C_6H_5$** (CPCM(cyclohexane))

| | | | |
|---|---|---|---|
| Ge | 4.645713000 | 4.778932000 | 17.511379000 |
| Ge | 1.352781000 | 5.282906000 | 15.620654000 |
| Cl | 4.249160000 | 5.096576000 | 14.171889000 |
| O | 1.902160000 | 4.807444000 | 18.553717000 |
| O | 3.867913000 | 2.910096000 | 17.194686000 |
| C | 0.643790000 | 5.743800000 | 12.832006000 |
| C | 4.091606000 | 5.131902000 | 19.347525000 |
| C | 6.574493000 | 4.519970000 | 17.460586000 |
| C | 8.588519000 | 3.486331000 | 18.324032000 |
| H | 9.074002000 | 2.826724000 | 19.038053000 |
| C | 4.953883000 | 5.496505000 | 20.385807000 |
| H | 6.028616000 | 5.500545000 | 20.222670000 |
| C | 2.028475000 | 1.071306000 | 15.554325000 |
| C | 3.058688000 | 5.905786000 | 21.832798000 |
| H | 2.681921000 | 6.216891000 | 22.801855000 |
| C | 0.279699000 | 6.491036000 | 11.714465000 |
| H | 0.179915000 | 6.006083000 | 10.747118000 |
| C | 0.775095000 | 6.348611000 | 14.086244000 |
| C | 0.049633000 | 7.858859000 | 11.836488000 |
| H | -0.232594000 | 8.443900000 | 10.965443000 |
| C | 1.480532000 | 0.460081000 | 14.418343000 |
| H | 1.111392000 | 1.080432000 | 13.605787000 |
| C | 7.339790000 | 5.163270000 | 16.481129000 |
| C | -1.138636000 | 6.604742000 | 19.477177000 |
| H | -1.690079000 | 6.937381000 | 20.350620000 |
| C | 7.211564000 | 3.678585000 | 18.381808000 |
| C | 4.436998000 | 5.880545000 | 21.619672000 |
| H | 5.108802000 | 6.171313000 | 22.421726000 |
| C | 8.719362000 | 4.978987000 | 16.432258000 |
| H | 9.306009000 | 5.484264000 | 15.670176000 |
| C | 2.166640000 | 5.537346000 | 20.825446000 |
| C | 0.188976000 | 8.476288000 | 13.077389000 |
| H | 0.017043000 | 9.544816000 | 13.176110000 |
| C | -0.952051000 | 6.480014000 | 17.068250000 |
| H | -1.349397000 | 6.718981000 | 16.085016000 |
| C | -1.641697000 | 6.885402000 | 18.208217000 |
| H | -2.577069000 | 7.428505000 | 18.109535000 |
| C | 0.052916000 | 5.896093000 | 19.631113000 |
| C | 0.710565000 | 5.508649000 | 18.463928000 |
| C | 0.650092000 | 5.464008000 | 20.967679000 |
| C | 9.343034000 | 4.140282000 | 17.351908000 |

| | | | |
|---|---|---|---|
| H | 10.418551000 | 3.991932000 | 17.309378000 |
| C | 2.537868000 | 0.262739000 | 16.579029000 |
| H | 2.961274000 | 0.718587000 | 17.466415000 |
| C | 2.722418000 | 5.160564000 | 19.604543000 |
| C | 2.044886000 | 2.546627000 | 15.638001000 |
| C | 0.552141000 | 7.726191000 | 14.192872000 |
| C | 0.259872000 | 5.792963000 | 17.178897000 |
| C | 3.157421000 | 3.316018000 | 16.207573000 |
| C | 1.026074000 | 3.358786000 | 15.249815000 |
| H | 0.095613000 | 2.920213000 | 14.888095000 |
| C | 1.929858000 | -1.721534000 | 15.340810000 |
| H | 1.893314000 | -2.804042000 | 15.258504000 |
| C | 1.431389000 | -0.923944000 | 14.312995000 |
| H | 1.010919000 | -1.381624000 | 13.422048000 |
| C | 2.480387000 | -1.123055000 | 16.470486000 |
| H | 2.869144000 | -1.738579000 | 17.276852000 |
| C | 0.262200000 | 3.983224000 | 21.210992000 |
| H | -0.827241000 | 3.891351000 | 21.277300000 |
| H | 0.706964000 | 3.629692000 | 22.147657000 |
| H | 0.614759000 | 3.343773000 | 20.396338000 |
| C | 0.136630000 | 6.308851000 | 22.133565000 |
| H | 0.385382000 | 7.367564000 | 22.005364000 |
| H | 0.565347000 | 5.958703000 | 23.076742000 |
| H | -0.948518000 | 6.211759000 | 22.226262000 |
| B | 3.402194000 | 4.848448000 | 15.816570000 |
| H | 6.858452000 | 5.804479000 | 15.746862000 |
| H | 6.631717000 | 3.163624000 | 19.144983000 |
| H | 0.833601000 | 4.677619000 | 12.721421000 |
| H | 0.659175000 | 8.223175000 | 15.155162000 |

Coordinates of compound **6-$CH_3$** (CPCM(cyclohexane))

| | | | |
|---|---|---|---|
| Ge | 4.429742000 | 4.004594000 | 4.716064000 |
| Ge | 7.394427000 | 3.806671000 | 7.059851000 |
| Cl | 9.360341000 | 4.856489000 | 7.151299000 |
| O | 4.632017000 | 3.344997000 | 7.713571000 |
| O | 7.208063000 | 6.520871000 | 4.323751000 |
| C | 3.327856000 | 3.701294000 | 7.419760000 |
| C | 0.752105000 | 4.391207000 | 6.785772000 |
| H | -0.272044000 | 4.652995000 | 6.536557000 |
| C | 5.320842000 | 2.447840000 | 3.956289000 |
| H | 5.008999000 | 1.592174000 | 3.358240000 |
| C | 3.021348000 | 3.916279000 | 6.081271000 |
| C | 2.423867000 | 3.888089000 | 8.467302000 |
| C | 1.698442000 | 4.254398000 | 5.774811000 |
| H | 1.407935000 | 4.440088000 | 4.744040000 |
| C | 7.827771000 | 2.127071000 | 3.793476000 |
| C | 6.485999000 | 4.408467000 | 8.690054000 |
| C | 1.118379000 | 4.233288000 | 8.121638000 |
| H | 0.374604000 | 4.388992000 | 8.896404000 |
| C | 5.161279000 | 4.014292000 | 8.807699000 |
| C | 4.356824000 | 4.266252000 | 9.915428000 |
| C | 4.943660000 | 4.977640000 | 10.961927000 |
| H | 4.366565000 | 5.214737000 | 11.850029000 |
| C | 7.802028000 | 0.796946000 | 3.343118000 |
| H | 6.860870000 | 0.254849000 | 3.323660000 |

| | | | |
|---|---|---|---|
| C | 6.274799000 | 5.389194000 | 10.885488000 |
| H | 6.711480000 | 5.940655000 | 11.712987000 |
| C | 6.599080000 | 2.813392000 | 4.221959000 |
| C | 2.937947000 | 3.704091000 | 9.893122000 |
| C | 7.048060000 | 5.102025000 | 9.762146000 |
| H | 8.082479000 | 5.430810000 | 9.713284000 |
| C | 10.189611000 | 0.836601000 | 2.987198000 |
| H | 11.102305000 | 0.334292000 | 2.679686000 |
| C | 6.969409000 | 5.447841000 | 4.658717000 |
| C | 8.969212000 | 0.161284000 | 2.944400000 |
| H | 8.931738000 | -0.870457000 | 2.605754000 |
| C | 9.062021000 | 2.788278000 | 3.841837000 |
| H | 9.109596000 | 3.810969000 | 4.207680000 |
| C | 2.033859000 | 4.375929000 | 10.926029000 |
| H | 2.429111000 | 4.219858000 | 11.933741000 |
| H | 1.033948000 | 3.933265000 | 10.905444000 |
| H | 1.945704000 | 5.452552000 | 10.746319000 |
| C | 10.231175000 | 2.152642000 | 3.437199000 |
| H | 11.176649000 | 2.685444000 | 3.482737000 |
| C | 3.010703000 | 2.187451000 | 10.201096000 |
| H | 3.653558000 | 1.667541000 | 9.485179000 |
| H | 2.008162000 | 1.748938000 | 10.149981000 |
| H | 3.414800000 | 2.031751000 | 11.207191000 |
| B | 6.554186000 | 4.132534000 | 5.166844000 |
| C | 7.816249000 | 1.906329000 | 7.277845000 |
| H | 6.890740000 | 1.331937000 | 7.168796000 |
| H | 8.531232000 | 1.581367000 | 6.519091000 |
| H | 8.231355000 | 1.743348000 | 8.275990000 |
| C | 3.795013000 | 5.245330000 | 3.328222000 |
| H | 4.590073000 | 5.411813000 | 2.596107000 |
| H | 2.929858000 | 4.821502000 | 2.808520000 |
| H | 3.506011000 | 6.203271000 | 3.770293000 |

Coordinates of compound **7-CH$_3$** (CPCM(cyclohexane))

| | | | |
|---|---|---|---|
| Ge | 4.634682000 | 4.907376000 | 17.447949000 |
| Ge | 1.299017000 | 5.360067000 | 15.586738000 |
| Cl | 4.229245000 | 5.210885000 | 14.157844000 |
| O | 1.880664000 | 4.829875000 | 18.513767000 |
| O | 3.969006000 | 3.006058000 | 17.080474000 |
| C | 4.073194000 | 5.170086000 | 19.298210000 |
| C | 4.937460000 | 5.494592000 | 20.348082000 |
| H | 6.012417000 | 5.506282000 | 20.185066000 |
| C | 2.048185000 | 1.145474000 | 15.611298000 |
| C | 3.048267000 | 5.839948000 | 21.817749000 |
| H | 2.673995000 | 6.114673000 | 22.798732000 |
| C | 1.524316000 | 0.495504000 | 14.486101000 |
| H | 1.162040000 | 1.088733000 | 13.650374000 |
| C | -1.147026000 | 6.616791000 | 19.500728000 |
| H | -1.689565000 | 6.931438000 | 20.386302000 |
| C | 4.425878000 | 5.827761000 | 21.598843000 |
| H | 5.099799000 | 6.088084000 | 22.409587000 |
| C | 2.154243000 | 5.500144000 | 20.802345000 |
| C | -0.972260000 | 6.556693000 | 17.088984000 |
| H | -1.372075000 | 6.833290000 | 16.116542000 |
| C | -1.649837000 | 6.943663000 | 18.242989000 |
| H | -2.574936000 | 7.507445000 | 18.164704000 |
| C | 0.036787000 | 5.889729000 | 19.627626000 |

| | | | |
|---|---|---|---|
| C | 0.684640000 | 5.528443000 | 18.446592000 |
| C | 0.638648000 | 5.416146000 | 20.948048000 |
| C | 2.544272000 | 0.373574000 | 16.670103000 |
| H | 2.946542000 | 0.861456000 | 17.550454000 |
| C | 2.705636000 | 5.168046000 | 19.566056000 |
| C | 2.048636000 | 2.623394000 | 15.654077000 |
| C | 0.226692000 | 5.843655000 | 17.171152000 |
| C | 3.167530000 | 3.416827000 | 16.167393000 |
| C | 1.003354000 | 3.411188000 | 15.285490000 |
| H | 0.071089000 | 2.941592000 | 14.968506000 |
| C | 1.975850000 | -1.653000000 | 15.482572000 |
| H | 1.949595000 | -2.737840000 | 15.433215000 |
| C | 1.488887000 | -0.891906000 | 14.422270000 |
| H | 1.087055000 | -1.380545000 | 13.539156000 |
| C | 2.499973000 | -1.015350000 | 16.603610000 |
| H | 2.877727000 | -1.602627000 | 17.435926000 |
| C | 0.255798000 | 3.927265000 | 21.144999000 |
| H | -0.833272000 | 3.829731000 | 21.209175000 |
| H | 0.702448000 | 3.545526000 | 22.069623000 |
| H | 0.609532000 | 3.315143000 | 20.310159000 |
| C | 0.126285000 | 6.221108000 | 22.142269000 |
| H | 0.371601000 | 7.284192000 | 22.047948000 |
| H | 0.558369000 | 5.841889000 | 23.072606000 |
| H | -0.958416000 | 6.117863000 | 22.234705000 |
| B | 3.340146000 | 4.968250000 | 15.795030000 |
| C | 6.581015000 | 4.779147000 | 17.357924000 |
| H | 6.947576000 | 4.049613000 | 18.086017000 |
| H | 6.882807000 | 4.479813000 | 16.351678000 |
| H | 7.024340000 | 5.755064000 | 17.581589000 |
| C | 0.623315000 | 6.394987000 | 14.062303000 |
| H | 1.234555000 | 6.181037000 | 13.181788000 |
| H | -0.415845000 | 6.129442000 | 13.844461000 |
| H | 0.676876000 | 7.466638000 | 14.274963000 |

Coordinates of compound **6-H** (CPCM(cyclohexane))

| | | | |
|---|---|---|---|
| Ge | -4.155683000 | -3.840669000 | -4.897050000 |
| Ge | -7.223947000 | -3.624661000 | -7.097859000 |
| Cl | -9.146712000 | -4.736897000 | -7.112031000 |
| O | -4.456200000 | -3.158803000 | -7.857310000 |
| O | -7.085949000 | -6.163559000 | -4.192187000 |
| C | -3.159252000 | -3.585811000 | -7.630990000 |
| C | -0.606446000 | -4.447151000 | -7.121780000 |
| H | 0.407472000 | -4.781892000 | -6.923736000 |
| C | -4.943708000 | -2.224893000 | -4.155542000 |
| H | -4.562477000 | -1.360037000 | -3.615077000 |
| C | -2.810261000 | -3.858106000 | -6.314240000 |
| C | -2.306821000 | -3.781638000 | -8.718153000 |
| C | -1.502136000 | -4.287621000 | -6.068856000 |
| H | -1.189100000 | -4.524541000 | -5.054816000 |
| C | -7.426635000 | -1.754808000 | -3.961555000 |
| C | -6.388702000 | -4.113775000 | -8.798558000 |
| C | -1.012195000 | -4.215297000 | -8.435397000 |
| H | -0.308184000 | -4.383722000 | -9.243848000 |
| C | -5.056029000 | -3.760580000 | -8.952875000 |
| C | -4.307597000 | -3.999321000 | -10.102885000 |

| | | | |
|---|---|---|---|
| C | -4.964590000 | -4.638166000 | -11.153847000 |
| H | -4.432597000 | -4.859825000 | -12.073500000 |
| C | -7.313415000 | -0.419661000 | -3.540045000 |
| H | -6.340157000 | 0.062665000 | -3.536775000 |
| C | -6.307650000 | -4.998654000 | -11.041771000 |
| H | -6.799837000 | -5.493354000 | -11.873789000 |
| C | -6.246552000 | -2.529480000 | -4.372912000 |
| C | -2.862024000 | -3.509846000 | -10.113723000 |
| C | -7.021645000 | -4.736728000 | -9.874799000 |
| H | -8.063876000 | -5.032712000 | -9.795681000 |
| C | -9.694480000 | -0.302775000 | -3.160783000 |
| H | -10.570406000 | 0.261234000 | -2.853412000 |
| C | -6.774763000 | -5.148169000 | -4.624176000 |
| C | -8.433910000 | 0.295326000 | -3.143324000 |
| H | -8.327986000 | 1.328634000 | -2.824698000 |
| C | -8.700543000 | -2.337531000 | -3.985799000 |
| H | -8.818705000 | -3.364355000 | -4.323681000 |
| C | -2.032487000 | -4.181617000 | -11.207524000 |
| H | -2.451044000 | -3.957702000 | -12.192804000 |
| H | -1.009446000 | -3.795115000 | -11.202794000 |
| H | -1.998668000 | -5.268611000 | -11.079337000 |
| C | -9.823336000 | -1.621632000 | -3.584242000 |
| H | -10.800525000 | -2.095064000 | -3.609784000 |
| C | -2.865917000 | -1.979446000 | -10.356166000 |
| H | -3.448799000 | -1.457252000 | -9.592311000 |
| H | -1.839748000 | -1.597240000 | -10.332046000 |
| H | -3.303062000 | -1.760611000 | -11.336249000 |
| B | -6.290991000 | -3.900377000 | -5.243689000 |
| H | -3.617036000 | -4.795448000 | -3.813209000 |
| H | -7.663382000 | -2.153039000 | -7.164845000 |

Coordinates of compound **7-H** (CPCM(cyclohexane))

| | | | |
|---|---|---|---|
| Ge | 4.615374000 | 4.900058000 | 17.450781000 |
| Ge | 1.298353000 | 5.346573000 | 15.602008000 |
| Cl | 4.222690000 | 5.214369000 | 14.146114000 |
| O | 1.875514000 | 4.827761000 | 18.516662000 |
| O | 3.976870000 | 3.008619000 | 17.066068000 |
| C | 4.069085000 | 5.160133000 | 19.298197000 |
| C | 4.939519000 | 5.482128000 | 20.344038000 |
| H | 6.013743000 | 5.484947000 | 20.176527000 |
| C | 2.060473000 | 1.138285000 | 15.586582000 |
| C | 3.053336000 | 5.843893000 | 21.814759000 |
| H | 2.682979000 | 6.124036000 | 22.795663000 |
| C | 1.521309000 | 0.495170000 | 14.464733000 |
| H | 1.143281000 | 1.092635000 | 13.639150000 |
| C | -1.144819000 | 6.630854000 | 19.496829000 |
| H | -1.686412000 | 6.952187000 | 20.380556000 |
| C | 4.430703000 | 5.822364000 | 21.593786000 |
| H | 5.107209000 | 6.080483000 | 22.402952000 |
| C | 2.154254000 | 5.506055000 | 20.802849000 |
| C | -0.970963000 | 6.560540000 | 17.084642000 |
| H | -1.366568000 | 6.833309000 | 16.109210000 |
| C | -1.646848000 | 6.953558000 | 18.237420000 |
| H | -2.569584000 | 7.520693000 | 18.156917000 |
| C | 0.036507000 | 5.900445000 | 19.628569000 |

| | | | |
|---|---|---|---|
| C | 0.681925000 | 5.530688000 | 18.449332000 |
| C | 0.638066000 | 5.432577000 | 20.951433000 |
| C | 2.578198000 | 0.361443000 | 16.631188000 |
| H | 2.992377000 | 0.843654000 | 17.509139000 |
| C | 2.701781000 | 5.165854000 | 19.567808000 |
| C | 2.056417000 | 2.615389000 | 15.639722000 |
| C | 0.224233000 | 5.842805000 | 17.173596000 |
| C | 3.172494000 | 3.410799000 | 16.150774000 |
| C | 1.007040000 | 3.403396000 | 15.281586000 |
| H | 0.072622000 | 2.937182000 | 14.968902000 |
| C | 2.000674000 | -1.658314000 | 15.436919000 |
| H | 1.979762000 | -2.742821000 | 15.379131000 |
| C | 1.492228000 | -0.891773000 | 14.390736000 |
| H | 1.078898000 | -1.375626000 | 13.510376000 |
| C | 2.540033000 | -1.027047000 | 16.554283000 |
| H | 2.934999000 | -1.618810000 | 17.375268000 |
| C | 0.246677000 | 3.947499000 | 21.159684000 |
| H | -0.842789000 | 3.857277000 | 21.227140000 |
| H | 0.693052000 | 3.569963000 | 22.086120000 |
| H | 0.594661000 | 3.326926000 | 20.328656000 |
| C | 0.133095000 | 6.249876000 | 22.140408000 |
| H | 0.385433000 | 7.310512000 | 22.037804000 |
| H | 0.564219000 | 5.874479000 | 23.072678000 |
| H | -0.952073000 | 6.154472000 | 22.235085000 |
| B | 3.342293000 | 4.962234000 | 15.774600000 |
| H | 6.151367000 | 4.790684000 | 17.425884000 |
| H | 0.739009000 | 6.161365000 | 14.425172000 |

Optimization of compound **6**.

On the basis of the molecular structure of **6** determined in the solid state the structure was optimized using the program Orca 6.0.1[36-42] along with BP86,[43, 44] Grimme´s dispersion correction and Becke-Johnson damping (D3BJ) with suitable RI approximations (RI/ RIJCOSX).[29] The basis sets employed were def2-TZVP for Ge, B as implemented in ORCA 5.0.3, and def2-SVP on all other elements.[32, 34, 45, 46] For all calculations, tight or very tight convergence criteria were applied for optimization and SCF convergence, respectively. Analyses of the electronic structures were performed using NBO7, plots were generated using ChemCraft.[47, 48]

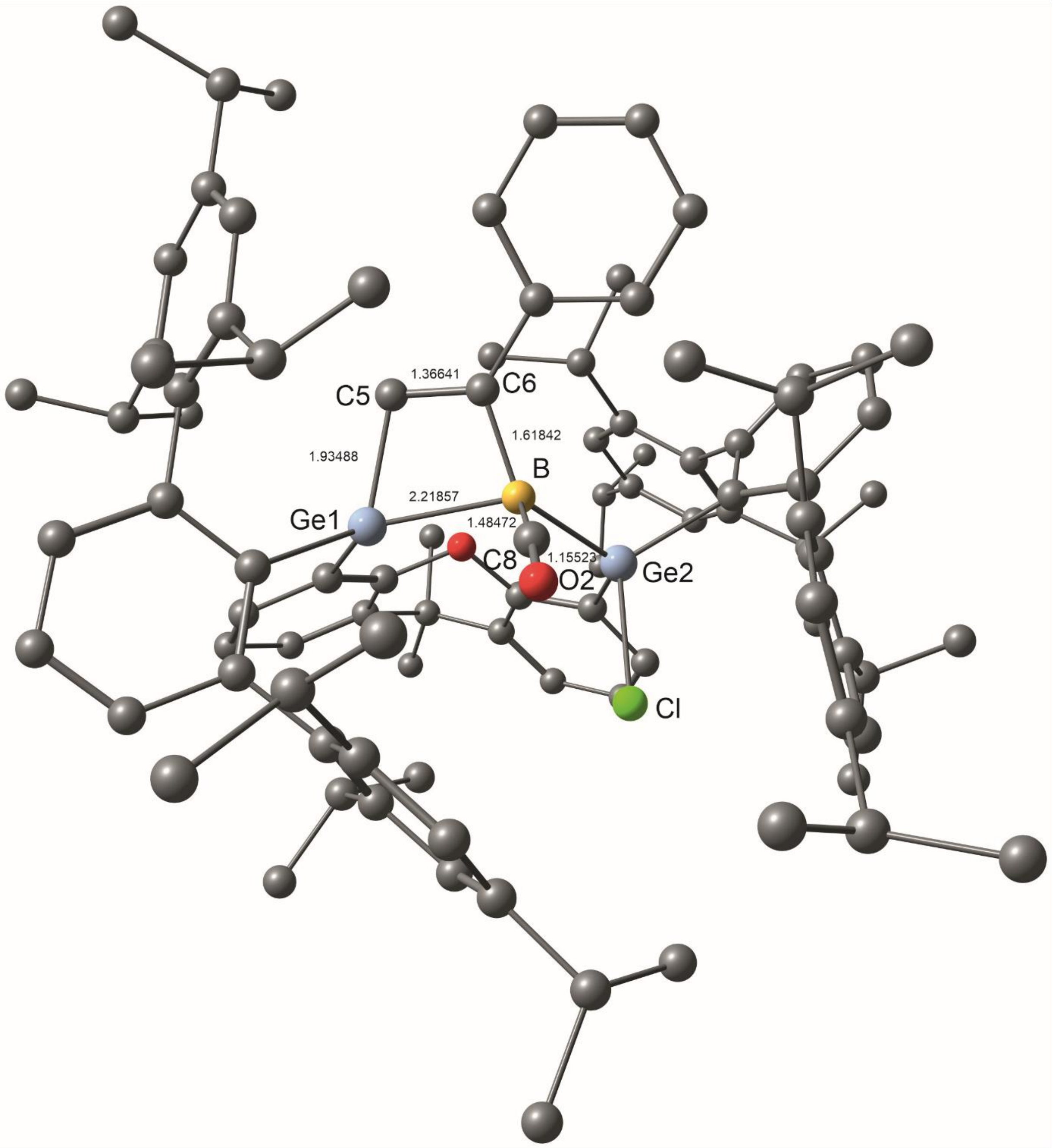


Figure S59. Optimized structure **6**, distances in Å.

218
Coordinates from ORCA-job bp86optfreq_hnumac E -8516.143914952969

| | | | |
|---|---|---|---|
| Ge | 4.59027897589753 | 4.27856063951734 | 4.68932463387493 |
| Ge | 7.52835349483331 | 4.48403013012415 | 7.08443373488437 |
| Cl | 7.55344193608939 | 6.71089010583732 | 7.04908219145154 |
| O | 4.68653230687753 | 3.32507062372266 | 7.54662102327992 |
| O | 7.57216563147691 | 6.20432720096791 | 3.76772086253215 |
| C | 11.64043909484761 | 3.30894791022948 | 6.55041275546109 |
| H | 12.53372389460269 | 3.70791273486342 | 6.04804812554113 |
| C | 3.38419564491771 | 3.73334841336404 | 7.28874678094977 |
| C | 2.42344735035721 | 7.34179453727054 | 2.71964350327397 |
| H | 2.25067893572923 | 8.42121336522914 | 2.84522375058444 |
| C | 0.83189825106437 | 4.62774597853926 | 6.71960135822431 |
| H | -0.18446122756334 | 4.98180309166704 | 6.49152222002975 |
| C | 5.24111603845617 | 2.53664100934332 | 4.15463914315108 |
| H | 4.76017601374577 | 1.65317032204352 | 3.70998852496402 |
| C | 3.12477557164494 | 4.20519242610602 | 6.00088399775872 |
| C | 5.37704416661246 | 9.03157186126998 | 5.84714027328729 |
| H | 5.31850045656856 | 9.32935048563762 | 6.90453718758865 |
| C | 11.65399788856693 | 2.02216708323414 | 7.09551290181639 |
| H | 12.54241077780376 | 1.38075632944176 | 6.99374420262955 |
| C | 2.43744455454891 | 3.73689772872583 | 8.32981410926947 |
| C | 1.87749210753836 | 5.23475294187197 | 1.66066200008188 |
| H | 1.28642155085737 | 4.64740174657011 | 0.94159204834146 |
| C | 1.58066514502657 | 6.58327021551628 | 1.89567168129123 |
| H | 0.72317385432141 | 7.05786845635373 | 1.39487174659573 |
| C | 10.76379850418795 | 7.90143234055399 | 6.40186028141734 |
| H | 10.80351628188881 | 8.76810210530028 | 7.07741303469356 |
| C | 1.81786895443076 | 4.65064699155484 | 5.72260494601807 |
| H | 1.57712085387743 | 5.05533401796602 | 4.72936543832887 |
| C | 6.35296160967273 | 9.60799630515974 | 5.02136877971372 |
| C | 6.39485963476727 | 9.20730883671589 | 3.67668574308267 |
| H | 7.14872946082582 | 9.66310376045173 | 3.01705341923553 |
| C | 5.50710173690193 | 8.25637626111136 | 3.14644467270791 |
| C | 7.61620462718678 | 1.72996463122573 | 4.00542319740961 |
| C | 2.93911154990988 | 4.60607223177426 | 2.34433949989660 |
| C | 3.97391628683533 | 0.53566018618023 | 1.24864626823820 |
| C | 7.29963098211240 | 10.67977492378031 | 5.54152532382948 |
| H | 8.09019913171983 | 10.80939150058265 | 4.77055151599550 |
| C | 10.49312866379828 | 4.12654562183412 | 6.63243841427976 |
| C | 6.47633474822759 | 4.32845974430214 | 8.73539045117682 |
| C | 4.52691876641740 | 7.68200138182622 | 4.00767480400781 |
| C | 1.14504584915611 | 4.19012703265741 | 8.01857475917799 |
| H | 0.37150830012586 | 4.21423315543838 | 8.79902183444569 |
| C | 3.71752948339962 | 5.35111484430993 | 3.27233600249102 |
| C | 10.64214790127354 | 6.61586954243244 | 6.94418225411461 |
| C | 10.98889431767500 | 4.55275650166448 | 3.67663828197033 |
| H | 10.87945170303704 | 3.60290564311797 | 4.22975483617853 |
| C | 3.52446551705866 | 6.75472070228327 | 3.37916378551565 |
| C | 5.17846319263855 | 3.81763327941462 | 8.74915817687630 |
| C | 5.66370861132348 | 0.29675965415265 | 6.77003967158776 |
| H | 5.31155033334482 | -0.62382700278551 | 7.28035552466911 |
| H | 5.59857196370869 | 0.10936052079418 | 5.68027984246823 |
| H | 4.97421257257103 | 1.12612573359620 | 7.01172687037679 |
| C | 4.45789221635255 | 8.08537460916116 | 5.36413805589645 |
| C | 6.44604787552294 | 0.58797107040741 | 9.60240554678430 |
| H | 5.61142254214372 | -0.07344834786221 | 9.32823805026721 |
| C | 4.79146187068764 | 1.62251909041638 | 0.89441773224749 |
| H | 5.71493325737227 | 1.42151032449987 | 0.33217196023539 |
| C | 9.33275877361426 | 3.65377626358853 | 7.31312193313438 |
| C | 10.59680910346187 | 5.50085678723881 | 6.05519929482360 |
| C | 6.56480856274576 | 12.02689206320464 | 5.68521605701115 |
| H | 6.10366711483900 | 12.33810391193965 | 4.72616366122357 |
| H | 7.25942025782385 | 12.82855271971733 | 6.01309243788004 |
| H | 5.75248841076180 | 11.95217241983670 | 6.43837424568926 |
| C | 4.32269810795018 | 3.87525306534585 | 9.86673741251547 |
| C | 4.81463768588763 | 4.51834305234302 | 11.01290910315229 |
| H | 4.17687520828782 | 4.61923217251641 | 11.90177570128231 |
| C | 7.29528074201797 | 0.62286117050624 | 3.18140489316335 |
| H | 6.26563859762727 | 0.50452336305029 | 2.81991434767499 |
| C | 7.30719002281604 | 1.05968435921618 | 8.59651651063202 |
| C | 10.54296583327036 | 1.58359716278760 | 7.82065254368572 |
| H | 10.55726322379204 | 0.60225737438304 | 8.31619671344830 |
| C | 12.01894299787314 | 5.94570609481640 | 8.95031315312027 |
| H | 12.78646149059610 | 6.72093101062173 | 8.74537148216621 |
| H | 12.33686518926251 | 5.01061482642008 | 8.44992031425628 |
| H | 12.00416287972411 | 5.75737216718878 | 10.04417088645951 |
| C | 10.75037218991888 | 5.69985580152387 | 4.65819391999798 |
| C | 3.28593160518897 | 3.19116995081743 | 1.99399235277952 |
| C | 2.81090563437644 | 0.80275571207868 | 1.98701098182860 |
| H | 2.16415687252195 | -0.03350468741870 | 2.29486513718771 |
| C | 6.11184984706475 | 5.05907851031187 | 11.03073703065642 |
| H | 6.47341648494566 | 5.57418197640933 | 11.93311152310133 |
| C | 5.18647781861563 | 1.40123588327984 | 13.00685606527735 |
| H | 6.00946355792959 | 1.82561982504281 | 13.61842601280916 |
| H | 4.44727299303537 | 0.96173544604480 | 13.70782372541918 |
| H | 4.70773306640746 | 2.24416493470904 | 12.47129554692119 |

| | | | |
|---|---|---|---|
| C | 6.61787584049469 | 0.92688730465718 | 10.95329295488845 |
| C | 6.57572892128585 | 2.69613134954325 | 4.40049656643765 |
| C | 7.69467004876418 | 1.76076561044572 | 11.29548182766985 |
| H | 7.84481011884707 | 2.03642358666503 | 12.34907797707810 |
| C | 2.44909996392068 | 2.10948500199313 | 2.36451571524745 |
| C | 5.42696947367939 | 4.07993258972917 | 0.87622830994968 |
| H | 5.40793569596032 | 4.81032334019479 | 1.71122435230317 |
| C | 3.36091354245058 | 7.57405996645091 | 6.28288046052635 |
| H | 2.87975268282749 | 6.72283301948247 | 5.77021884095051 |
| C | 10.80458544312587 | 7.01709351880657 | 4.16423390307978 |
| H | 10.87580481312333 | 7.16715442622144 | 3.07709272001334 |
| C | 2.91758025334380 | 3.27905786291186 | 9.70915488371710 |
| C | 7.98797427052163 | 10.27114748891534 | 6.85294101423790 |
| H | 7.25972181928765 | 10.18207585559750 | 7.68550281644757 |
| H | 8.74182386555710 | 11.02758749637286 | 7.15555742480536 |
| H | 8.49357595249171 | 9.29242888346015 | 6.74690656556960 |
| C | 7.11130548060696 | 0.63653033799322 | 7.14514546520378 |
| H | 7.42031771814108 | 1.49705402097405 | 6.51721256634965 |
| C | 4.48735629482370 | 2.94259858683977 | 1.26588008477578 |
| C | 5.55354296378372 | 7.93419670690269 | 1.65204247325953 |
| H | 5.04641018874263 | 6.95962664421354 | 1.50565177366720 |
| C | 4.35111168398663 | -0.88385477885028 | 0.84814241184771 |
| H | 5.37153442906632 | -0.83137910646737 | 0.40772394506995 |
| C | 9.39800979712893 | 2.39385322214904 | 7.97529094356545 |
| C | 6.93624637601859 | 4.96211721579563 | 9.90261860295907 |
| H | 7.93607203175512 | 5.42035284384010 | 9.91175286898883 |
| C | 9.60591611655257 | -0.14110857753328 | 3.23302344950090 |
| H | 10.37714748747771 | -0.86727625486303 | 2.93440677163730 |
| C | 5.70561833399144 | 0.34153648948188 | 12.02209074858778 |
| H | 4.82803400486811 | -0.09121280812086 | 11.49278335471874 |
| C | 9.54253697271538 | 3.97202451933914 | 11.99753920830617 |
| H | 8.62747853020231 | 4.58100572115751 | 11.88820817276674 |
| H | 10.40004342813214 | 4.65823625335892 | 12.15177136946978 |
| H | 9.43815597364821 | 3.37403728298580 | 12.92672403049176 |
| C | 10.91363965469480 | 9.55367850544890 | 4.48574026851186 |
| H | 10.33931083556125 | 10.19504821028621 | 5.18968742120838 |
| C | 7.25506826460911 | 5.26958539465003 | 4.36793581474727 |
| C | 10.15277035828336 | 7.62531318765629 | 9.26299350618060 |
| H | 10.86760106211497 | 8.47272561731005 | 9.20599833705796 |
| H | 10.05874296930699 | 7.35473486910519 | 10.33473273575127 |
| H | 9.16445796071856 | 7.97481498252981 | 8.90789470777190 |
| C | 10.00362180291561 | 4.49606163165755 | 2.50043458574809 |
| H | 8.98563367939214 | 4.23327182932455 | 2.83753041352203 |
| H | 10.31095664073934 | 3.71289384609295 | 1.77835196376329 |
| H | 9.94383783736773 | 5.45970973960726 | 1.95403727042658 |
| C | 8.59318111278805 | 2.24654471832154 | 10.33351587889839 |
| C | 9.79986304603982 | 3.07892093782244 | 10.77272821151024 |
| H | 10.07306762010456 | 3.74033518080172 | 9.92394814199639 |
| C | 8.27587385064149 | -0.30044215920798 | 2.80162578742944 |
| H | 8.00133711886356 | -1.15078881669396 | 2.15827052705823 |
| C | 1.29598434652027 | 1.50510383931963 | 4.53376404219998 |
| H | 1.25918672332871 | 0.41162642667232 | 4.34694559296490 |
| H | 0.45383987720803 | 1.76297938608277 | 5.20766509035348 |
| H | 2.23623055792045 | 1.72793438815529 | 5.07498209683995 |
| C | 1.20617573205676 | 2.30753756346181 | 3.22253960639108 |
| H | 1.16352055812427 | 3.37907602228040 | 3.49640485188943 |
| C | 8.95379136149990 | 1.87561889220359 | 4.42287934733569 |
| H | 9.21849311992342 | 2.71948460608694 | 5.06920334154262 |
| C | 10.80304170121317 | 8.13001652661360 | 5.01366426278083 |
| C | 8.38371552897228 | 1.90437715370219 | 8.96756118609277 |
| C | 10.63330196505833 | 6.41060937095653 | 8.45959454500321 |
| H | 9.92377693756995 | 5.58326127079564 | 8.66887938879097 |
| C | 6.88785055252127 | 3.64804168171165 | 0.70020250851232 |
| H | 7.24919632428695 | 3.06961886553268 | 1.57305058237377 |
| H | 7.53304938133874 | 4.54209405007675 | 0.58973553825405 |
| H | 7.03664666357191 | 3.02505253088487 | -0.20623338268657 |
| C | 1.97270209532704 | 3.72675477406573 | 10.83097960265081 |
| H | 2.34917343491795 | 3.38563079130389 | 11.81534469421008 |
| H | 0.96824197877320 | 3.27857444701120 | 10.70005779508177 |
| H | 1.86282545394933 | 4.82907685190746 | 10.86148147611551 |
| C | 4.76251311895240 | 8.99008780477120 | 0.85308438895769 |
| H | 3.71384882069765 | 9.06330248225832 | 1.19785676542970 |
| H | 4.74998611735521 | 8.73839493571937 | -0.22794728622614 |
| H | 5.22656728481834 | 9.99208352427480 | 0.96791755266290 |
| C | 9.93895666335676 | 0.95315559186320 | 4.04622824335244 |
| H | 10.97125594590677 | 1.09405746840185 | 4.39898688985011 |
| C | 4.90182724559284 | 4.80834171168153 | -0.37587700584604 |
| H | 5.57225117754792 | 5.64723926195492 | -0.65271706471870 |
| H | 3.88803223741533 | 5.22189238486134 | -0.20795092475912 |
| H | 4.84824901620529 | 4.11166701863131 | -1.23833257045239 |
| C | 3.00716786088179 | 1.72929894975575 | 9.70545215973968 |
| H | 3.69717589117954 | 1.36834068875249 | 8.92054868864875 |
| H | 2.00597777793518 | 1.29188270603512 | 9.51713563505753 |
| H | 3.37776739814336 | 1.36320922642951 | 10.68220467380378 |
| C | 12.44425184219294 | 4.60882083368999 | 3.16766639776514 |
| H | 12.61930450373301 | 5.51269065071481 | 2.54792619551546 |
| H | 12.67652817543769 | 3.71984522211211 | 2.54498672086783 |
| H | 13.16774427929306 | 4.64299914613190 | 4.00755421614228 |
| C | 8.03132436478764 | -0.54343230683891 | 6.77448357468053 |
| H | 9.09948269594809 | -0.29451636741529 | 6.90953905310639 |

| | | | |
|---|---|---|---|
| H | 7.88726289917499 | -0.82337430896193 | 5.71148341599781 |
| H | 7.79881313171492 | -1.42681779467034 | 7.40550664380475 |
| C | 6.97410571071714 | 7.80536520222987 | 1.07675574986477 |
| H | 7.52433720407435 | 8.76828376859182 | 1.11341781424967 |
| H | 6.92448203254914 | 7.50634093466134 | 0.00965647349083 |
| H | 7.57575305752026 | 7.05317146140458 | 1.61844912006524 |
| C | 6.41242060170711 | -0.81191324653327 | 12.76116190658700 |
| H | 6.74932516899695 | -1.59467316874744 | 12.05224850000643 |
| H | 5.73790552171327 | -1.28402511879581 | 13.50591597581477 |
| H | 7.30910004706076 | -0.44103906691064 | 13.30056466133719 |
| C | 4.41210474191925 | -1.83289007706890 | 2.05888078604928 |
| H | 5.10118636683673 | -1.45351181163336 | 2.84011678019703 |
| H | 4.76115297266553 | -2.84072474115362 | 1.75333668510694 |
| H | 3.41351292687235 | -1.95658312858636 | 2.52688014276196 |
| C | 10.30519839365400 | 9.73345677718495 | 3.08817753514754 |
| H | 10.91300350608055 | 9.22938511942948 | 2.30806420157725 |
| H | 10.25487737230699 | 10.80769848965239 | 2.81742899779255 |
| H | 9.28177290131862 | 9.31218661035001 | 3.04016234209284 |
| C | 3.89429523146161 | 7.05905504621474 | 7.62764832814299 |
| H | 4.71567387803239 | 6.33183015187861 | 7.48620578184515 |
| H | 3.08681067424981 | 6.56239000617432 | 8.20137474448722 |
| H | 4.29920369599571 | 7.87960551336665 | 8.25510942830711 |
| C | 3.39979676637392 | -1.42119989825286 | -0.23822922351965 |
| H | 3.70536034409245 | -2.43480835063650 | -0.57147089741403 |
| H | 3.38530177771268 | -0.75467997798781 | -1.12378579659792 |
| H | 2.36051255287920 | -1.49024830047123 | 0.14600106826562 |
| C | 2.26796012608576 | 8.64067018306858 | 6.47770805419760 |
| H | 2.67336965220601 | 9.54269075548407 | 6.98249627668425 |
| H | 1.44000581050966 | 8.24188101735447 | 7.10042271480294 |
| H | 1.84286860604141 | 8.96285581853215 | 5.50545717265285 |
| C | -0.08040935734533 | 1.97980732224740 | 2.44445630970659 |
| H | -0.16728931636688 | 2.59797068118242 | 1.52806540542797 |
| H | -0.97796136850478 | 2.16475976152028 | 3.07035147866747 |
| H | -0.10235798557724 | 0.91491825314685 | 2.13109990828700 |
| C | 12.38082275061769 | 10.02756978356698 | 4.52162614086992 |
| H | 12.80684030892704 | 9.94280135396184 | 5.54147080421455 |
| H | 12.46889640969321 | 11.08544982243295 | 4.19680877249516 |
| H | 13.00562743244774 | 9.40685583506900 | 3.84564553210691 |
| C | 11.01326950566508 | 2.17099103956533 | 11.06381406574258 |
| H | 10.78018205736120 | 1.47348557566509 | 11.89536378051683 |
| H | 11.89366914885728 | 2.77843295638743 | 11.36001719762661 |
| H | 11.29975596413601 | 1.56693554414208 | 10.18409252177597 |
| B | 6.75301474730504 | 4.11610466899003 | 5.15649936332235 |

# References


1. R. H. Kern, M. Schneider, K. Eichele, H. Schubert, H. F. Bettinger and L. Wesemann, *Angew. Chem., Int. Ed.*, 2023, **62**, e202301593.
2. R. K. Harris, E. D. Becker, S. M. C. d. Menezes, R. Goodfellow and P. Granger, *Pure Appl. Chem.*, 2001, **73**, 1795-1818.
3. L. J. Farrugia, *J. Appl. Crystallogr.*, 1997, **30**, 565.
4. L. J. Farrugia, *J. Appl. Crystallogr.*, 1999, **32**, 837-838.
5. L. J. Farrugia, *J. Appl. Crystallogr.*, 2012, **45**, 849-854.
6. C. B. Hübschle, G. M. Sheldrick and B. Dittrich, *J. Appl. Crystallogr.*, 2011, **44**, 1281-1284.
7. G. M. Sheldrick, *Program for crystal-structure refinement. University of Gottingen, Germany.*, 1997.
8. G. M. Sheldrick, *Acta Cryst., Sect. A*, 2008, **64**, 112-122.
9. G. M. Sheldrick, *Journal*, 1996.
10. S. Grimme, A. Hansen, S. Ehlert and J.-M. Mewes, *J. Chem. Phys.*, 2021, **154**, 064103.
11. J. W. Furness, A. D. Kaplan, J. Ning, J. P. Perdew and J. Sun, *The Journal of Physical Chemistry Letters*, 2020, **11**, 8208-8215.
12. J. W. Furness, A. D. Kaplan, J. Ning, J. P. Perdew and J. Sun, *The Journal of Physical Chemistry Letters*, 2020, **11**, 9248-9248.
13. E. Caldeweyher, C. Bannwarth and S. Grimme, *J. Chem. Phys.*, 2017, **147**, 034112.
14. E. Caldeweyher, S. Ehlert, A. Hansen, H. Neugebauer, S. Spicher, C. Bannwarth and S. Grimme, *J. Chem. Phys.*, 2019, **150**, 154122.
15. H. Kruse and S. Grimme, *J. Chem. Phys.*, 2012, **136**, 154101.
16. S. Grimme, *Chem.-Eur. J.*, 2012, **18**, 9955-9964.
17. V. Barone and M. Cossi, *J. Phys. Chem. A*, 1998, **102**, 1995-2001.
18. C. Riplinger and F. Neese, *J. Chem. Phys.*, 2013, **138**, 034106.
19. C. Riplinger, B. Sandhoefer, A. Hansen and F. Neese, *J. Chem. Phys.*, 2013, **139**, 134101.
20. C. Riplinger, P. Pinski, U. Becker, E. F. Valeev and F. Neese, *J. Chem. Phys.*, 2016, **144**, 024109.
21. G. Santra, N. Sylvetsky and J. M. L. Martin, *J. Phys. Chem. A*, 2019, **123**, 5129-5143.
22. S. Grimme, *J. Chem. Phys.*, 2006, **124**.
23. A. D. Becke, *J. Chem. Phys.*, 1993, **98**, 5648-5652.
24. C. Lee, W. Yang and R. G. Parr, *Phys. Rev. B*, 1988, **37**, 785-789.
25. P. J. Stephens, F. J. Devlin, C. F. Chabalowski and M. J. Frisch, *J. Phys. Chem.*, 1994, **98**, 11623-11627.
26. Y. Zhao and D. G. J. T. C. A. Truhlar, *Theor. Chem. Account*, 2008, **120**, 215-241.
27. J.-D. Chai and M. Head-Gordon, *J. Chem. Phys.*, 2008, **128**.
28. N. Mardirossian and M. Head-Gordon, *Phys. Chem. Chem. Phys.*, 2014, **16**, 9904-9924.
29. S. Grimme, S. Ehrlich and L. Goerigk, *J. Comput. Chem.*, 2011, **32**, 1456-1465.
30. W. Hujo and S. Grimme, *J. Chem. Theo. Comp.*, 2011, **7**, 3866-3871.
31. O. A. Vydrov and T. Van Voorhis, *J. Chem. Phys.*, 2010, **133**, 244103.
32. F. Weigend and R. Ahlrichs, *Phys. Chem. Chem. Phys.*, 2005, **7**, 3297-3305.
33. F. Weigend, M. Häser, H. Patzelt and R. Ahlrichs, *Chem. Phys. Lett.*, 1998, **294**, 143-152.
34. F. Weigend, *Phys. Chem. Chem. Phys.*, 2006, **8**, 1057-1065.
35. F. Weigend, *J. Comput. Chem.*, 2008, **29**, 167-175.
36. F. Neese, *Wiley Interdiscip. Rev. Comput. Mol. Sci.*, 2018, **8**, e1327.
37. F. Neese, *Wiley Interdiscip. Rev. Comput. Mol. Sci.*, 2012, **2**, 73-78.
38. F. Neese, *WIREs Comput Mol Sci.*, 2022, **12**, e1606.
39. F. Neese, *J. Comput. Chem.*, 2003, **24**, 1740-1747.
40. D. Bykov, T. Petrenko, R. Izsák, S. Kossmann, U. Becker, E. Valeev and F. Neese, *Mol. Phys.*, 2015, **113**, 1961-1977.
41. F. Neese, *J. Comput. Chem.*, 2023, **44**, 381-396.

42. F. Neese, F. Wennmohs, U. Becker and C. Riplinger, *J. Chem. Phys.*, 2020, **152**, 224108.
43. A. D. Becke, *Phys. Rev. A*, 1988, **38**, 3098-3100.
44. J. P. Perdew and W. Yue, *Phys. Rev. B*, 1986, **33**, 8800-8802.
45. B. Metz, H. Stoll and M. Dolg, *J. Chem. Phys.*, 2000, **113**, 2563-2569.
46. D. Andrae, U. Häussermann, M. Dolg, H. Stoll and H. Preuß, *Theoret. Chim. Acta*, 1990, **77**, 123-141.
47. G. A. Zhurko, *CHEMCRAFT (http://www.chemcraftprog.com)*.
48. E. D. Glendening, J. K. Badenhoop, A. E. Reed, J. E. Carpenter, J. A. Bohmann, C. M. Morales, P. Karafiloglou, C. R. Landis and F. Weinhold, NBO 7.0, 2018.
